# Stabilizing short-lived Schiff base derivatives of 5-aminouracils that activate mucosal-associated invariant T cells

**DOI:** 10.1038/ncomms14599

**Published:** 2017-03-08

**Authors:** Jeffrey Y. W. Mak, Weijun Xu, Robert C. Reid, Alexandra J. Corbett, Bronwyn S. Meehan, Huimeng Wang, Zhenjun Chen, Jamie Rossjohn, James McCluskey, Ligong Liu, David P. Fairlie

**Affiliations:** 1 Division of Chemistry and Structural Biology, Institute for Molecular Bioscience, The University of Queensland, Brisbane, Queensland 4072, Australia; 2 Australian Research Council Centre of Excellence in Advanced Molecular Imaging, The University of Queensland, Brisbane, Queensland 4072, Australia; 3 Department of Microbiology & Immunology, Peter Doherty Institute for Infection and Immunity, University of Melbourne, Parkville, Victoria 3010, Australia; 4 Infection and Immunity Program & Department of Biochemistry and Molecular Biology, Biomedicine Discovery Institute, Monash University, Clayton, Victoria 3800, Australia; 5 Australian Research Council Centre of Excellence in Advanced Molecular Imaging, Monash University, Clayton, Victoria 3800, Australia; 6 Institute of Infection and Immunity, Cardiff University, School of Medicine, Heath Park, Cardiff CF14 4XN, UK

## Abstract

Mucosal-associated invariant T (MAIT) cells are activated by unstable antigens formed by reactions of 5-amino-6-D-ribitylaminouracil (a vitamin B2 biosynthetic intermediate) with glycolysis metabolites such as methylglyoxal. Here we show superior preparations of antigens in dimethylsulfoxide, avoiding their rapid decomposition in water (*t*_1/2_ 1.5 h, 37 °C). Antigen solution structures, MAIT cell activation potencies (EC_50_ 3–500 pM), and chemical stabilities are described. Computer analyses of antigen structures reveal stereochemical and energetic influences on MAIT cell activation, enabling design of a water stable synthetic antigen (EC_50_ 2 nM). Like native antigens, this antigen preparation induces MR1 refolding and upregulates surface expression of human MR1, forms MR1 tetramers that detect MAIT cells in human PBMCs, and stimulates cytokine expression (IFNγ, TNF) by human MAIT cells. These antigens also induce MAIT cell accumulation in mouse lungs after administration with a co-stimulant. These chemical and immunological findings provide new insights into antigen properties and MAIT cell activation.

## Abstract (web summary)

MAIT cells are activated by MR1 restricted antigens derived from riboflavin biosynthesis. Here the authors characterize MAIT cell antigenicity and synthesize a water stable antigen that activates human MAIT cells *in vitro* and mouse MAIT cells *in vivo*.

T cells use their surface T-cell receptors (TCRs) to recognize specific microbial antigens that are captured and presented by antigen-presenting cells (APC). T cells are typically activated by peptides^1^ or lipids^2^,^3^, but mucosal-associated invariant T cells (MAIT cells)^4^ are innate-like T cells in the blood^5^,^6^, liver^7^,^8^,^9^,^10^,^11^,^12^ and gut mucosa^9^,^13^,^14^ that are activated instead through covalent binding to pyrimidine derivatives that feature a chemically unstable α-iminocarbonyl Schiff base component (R^1^CO–CR^2^=NH–)^15^,^16^. These antigens are formed from 5-amino-6-D-ribitylaminouracil (**1**, 5-A-RU), a key intermediate in the biosynthesis of riboflavin (vitamin B2) by many bacteria and fungi^15^,^17^. During microbial riboflavin biosynthesis, this intermediate undergoes enzyme-catalyzed condensation with 3,4-dihydroxy-2-butanone-4-phosphate (**2**), followed by rapid cyclization to form 6,7-dimethyl-8-(D-ribityl)lumazine (**4a**), which subsequently dismutates to give riboflavin (Fig. 1a). However, **1** can also react through non-enzymatic Schiff base condensation with α-dicarbonyl compounds (**5a-c**) (Fig. 1a), such as glyoxal (**5b**) and methylglyoxal (pyruvaldehyde, **5c**)^18^,^19^, derived from mammalian glycolysis or bacterial metabolism to give the adducts 5-(1-methyl-2-oxopropylideneamino)-6-D-ribitylaminouracil (5-MOP-RU, **3a**; a riboflavin biosynthetic intermediate), 5-(2-oxoethylideneamino)-6-D-ribitylaminouracil (5-OE-RU, **3b**), and 5-(2-oxopropylideneamino)-6-D-ribitylaminouracil (5-OP-RU, **3c**).

These α-iminocarbonyl adducts **3a-c** are very unstable chemical entities, susceptible to hydrolysis or intramolecular cyclization to give bicyclic lumazines **4a-c**. Nevertheless, **3b** and **3c** persist long enough to be captured and stabilized through a covalent bond with Lys43 of the major histocompatibility complex (MHC) class I-related protein (MR1)^15^. This creates a second Schiff base and anchors **3b-c** to MR1 for subsequent presentation to the TCR of MAIT cells (Fig. 1b). The formation of this covalent bond between the antigen and the antigen-presenting protein is a striking and novel way to stabilize a T-cell antigen. Furthermore, although a Schiff base ligand-protein covalent bond is not without precedence in nature (for example, pyridoxal phosphate, fructose 1,6-bisphosphate, retinal)^20^, the linkage between the uracil and MR1 involves two consecutive Schiff bases, creating a 1,2-diimino moiety (–NH=CR^1^–CR^2^=N–; Fig. 1c) that is unprecedented in biological chemistry. Crystal structures of the MR1-antigen-TCR ternary complexes reveal that the antigen is almost encircled by MR1, with multiple hydrogen bonds from the uracil and ribityl components to MR1 and TCR residues (Fig. 1c)^15^,^21^,^22^,^23^,^24^. Two tyrosine residues from MR1 (Y7, Y62) are positioned for *π*-interactions with the pyrimidine ring. The novelty and potential immunological importance of these structures demands an understanding of the synthesis, structure, and chemical properties of **3a-c**, as well as the identification of stereochemical requirements for antigen potency and stability and for MR1-dependent MAIT cell activation, all of which are described here.

## Results

### Synthesis of unstable MAIT cell antigens

**Figure 1:**
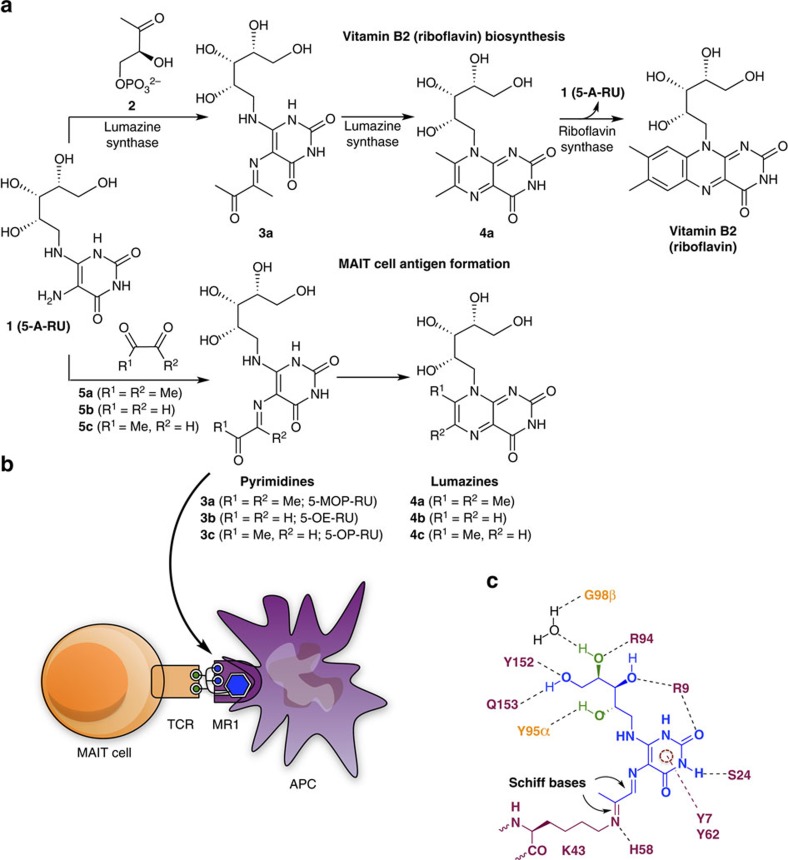
Glycolysis metabolites react with a vitamin B2 biosynthetic intermediate to create MAIT cell antigens. (**a**) Enzymatic biosynthesis of vitamin B2 (top), and formation of antigens from biosynthetic intermediate **1** by condensation with glycolysis metabolites **5a-c** (bottom). (**b**) Complex of antigen (blue) sandwiched between MR1 (purple) on the surface of an antigen-presenting cell APC and T-cell receptor protein TCR (orange) on the surface of a MAIT cell. (**c**) Interactions observed in a ternary crystal structure of a human MAIT TCR-**3c**-MR1 complex^15^, showing antigen (blue)···MR1 (purple) and antigen (green)···TCR (orange) contacts, with an extended conjugated system involved in *π*-interactions (dashed circle).

The condensation of uracil derivative **1** with α-dicarbonyls **5** in water is known^25^,^26^,^27^,^28^,^29^,^30^,^31^ to produce lumazines (Fig. 1a), but the anticipated putative imine intermediates **3a-c** had not been isolated nor characterized prior to our work. These fleeting intermediates cannot be efficiently synthesized in water for biological applications for three reasons. First, the condensation of diamine **1** with unsymmetrical α-dicarbonyls **5** generates a mixture of inseparable regioisomers. Second, formation of imines (as in **3a-c**) and their hydrolyses are reversible in water. Third, **3a-c** undergo rapid intramolecular condensation between amine and carbonyl groups to form bicyclic lumazines **4a-d** (Fig. 1a), a process likely to be driven thermodynamically by aromatization.

**Figure 2:**
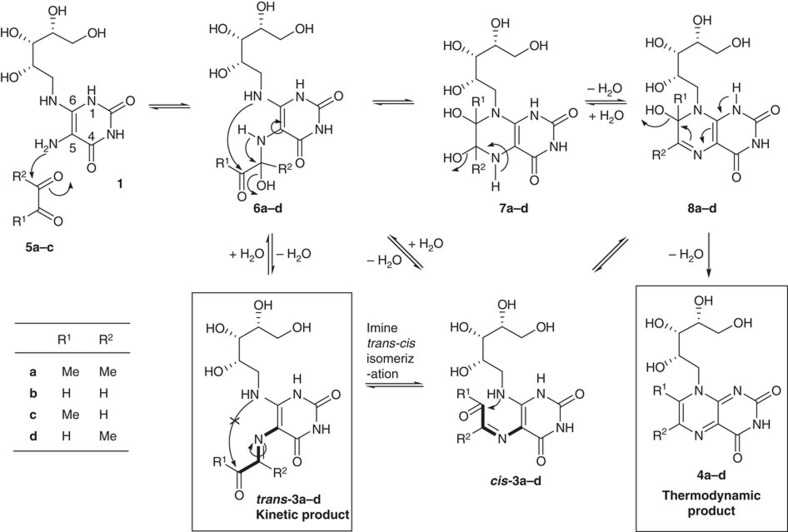
Proposed mechanism for reaction between 1 and 5 to produce uracil and lumazine derivatives. These reactions are reversible in water, but dehydrative cyclization drives formation of thermodynamically more stable aromatic lumazine derivatives **4**. Polar aprotic solvents may facilitate kinetic control of these equilibria and enable *trans*-**3** to persist in solution.

These limitations were evident when the reactions between **1** and **5a-c** were monitored in water (Fig. 2). For example, the reaction with **5c** was monitored at different pH (pH 7.4, PBS; pH 5.4, 20 mM NH_4_OAc buffer, 150 mM NaCl) and temperature (37 and 15 °C) using analytical LCMS (Supplementary Fig. 1). Regioisomers can form depending on whether the C-5 amine of **1** condenses with the more reactive aldehyde or the ketone of **5c** to give **4c** or **4d**, respectively (see Supplementary Figs 22–30 for NMR spectra). The rate of formation of imines **3c-d** and their subsequent cyclization to lumazines **4c-d** were highly dependent on reaction conditions, with **4c** forming preferentially over **4d** with increasing acidity or temperature (Supplementary Fig. 2). The concentration of **3c** typically peaked at approximately 5–10 min (Supplementary Fig. 1). Compound **1** reacted even faster with **5b** than **5c**, with the concentration of **3b** peaking at 1 min before completely cyclizing within 30 min (pH 7.4, PBS; 15 °C) to **4b** (Supplementary Fig. 3).

We discovered that the unstable intermediates **3a-c** could be isolated as kinetic products by exploiting key stereochemical properties, as revealed by the reaction mechanism between **1** and **5** (Fig. 2). Diamine **1** should react with dicarbonyl **5** to preferentially form hemiaminal **6**, as the C-5 amine is more nucleophilic than the C-6 amine at the β-position of a vinylogous amide (Supplementary Fig. 2). Elimination of water from **6** gives *trans*-**3**, in which the amine and carbonyl groups are held apart by the *trans*-configuration to prevent cyclization, unlike *cis*-**3**, which is preorganized for cyclzation. If the reaction of **1** and **5** was carried out in water, the reaction proceeds under thermodynamic control, with *trans*-**3** rehydrating to **6** followed by bond rotation and cyclization via **7** (or *cis*-**3**) and **8** to give lumazine **4** (Fig. 2). However, the reaction between **1** and **5** in a non-aqueous solvent should proceed under kinetic control. In this scenario, *trans*-**3** should form faster than *cis*-**3** from **6** for steric reasons (Supplementary Fig. 4). Once formed, *trans*-**3** is unlikely to isomerize to *cis*-**3** as this transformation has a large and prohibitive activation energy barrier^32^, estimated by *ab initio* gas phase density functional theory (DFT) calculations to be 20–28 kcal mol^−1^ (Supplementary Fig. 5). Thus, if the formation of *trans*-**3** from **6** was also faster than the formation of **7**, then *trans*-**3** should accumulate as the kinetic product (Fig. 2).

**Figure 3:**
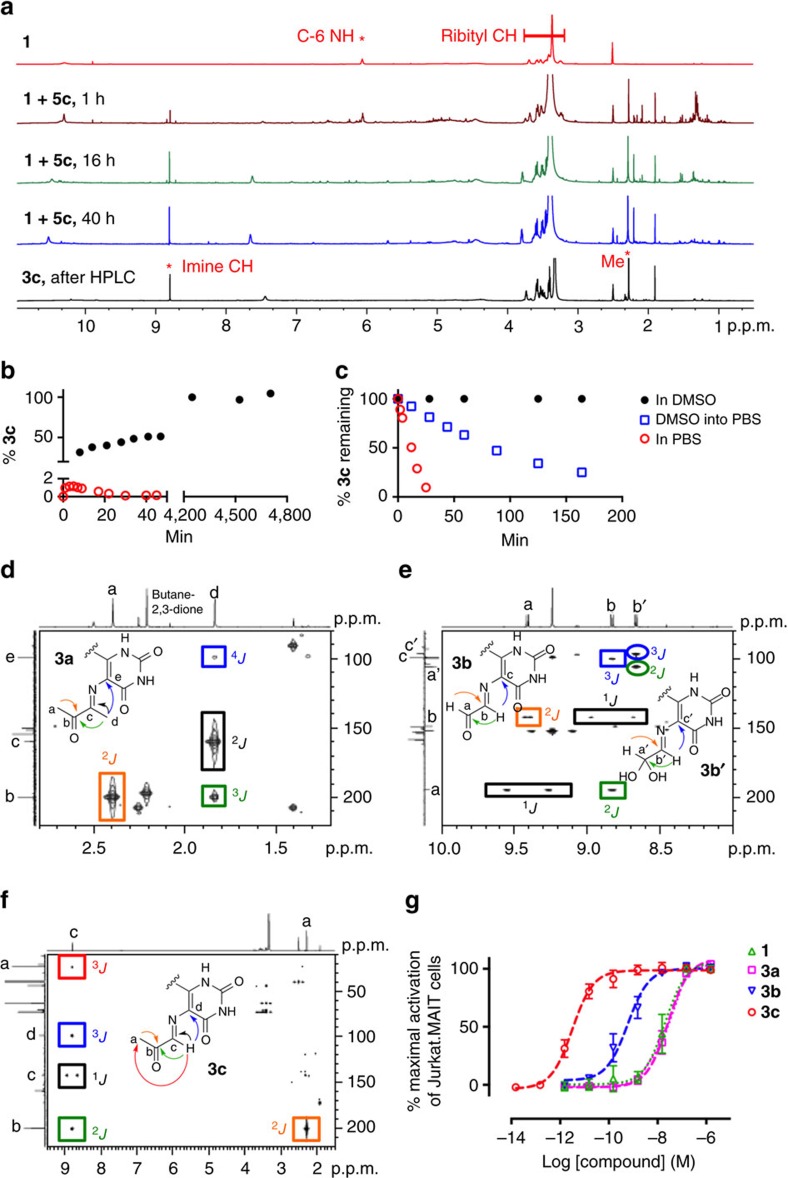
Formation and solution structures of uracil-based antigens that activate MAIT cells. (**a**) Exclusive formation of 5-OP-RU **3c** from **1** and **5c** in DMSO-*d*_6_, as monitored by ^1^H NMR spectroscopy, with the characteristic signals of **1** and 5-OP-RU **3c** as indicated. (**b**) Formation of 5-OP-RU **3c** in DMSO (black) versus PBS buffer (red). In PBS buffer (red, 2.62 mM of **1**, 7.86 mM of **5c**, pH 7.4, 37 °C), **3c** reached a maximum concentration corresponding to only 1.1% conversion of **1** at 5 min. In DMSO (black, 31.8 mM **1** and 34.5 mM **5c**, room temperature), 5-OP-RU **3c** reached 100% conversion after 2 days and then plateaued. (**c**) Stability of 5-OP-RU **3c** in DMSO (black), PBS buffer (red) and after the DMSO solution was diluted into PBS buffer (blue). Data for PBS buffer were extracted from **b** (red) at the time the maximum 5-OP-RU **3c** concentration was reached (that is, 5 min after mixing). (**d**–**f**) HMBC NMR spectra of **3a-c** (DMSO-*d*_6_), respectively. Square boxes indicate correlations for the aldehyde form of **3b**, and circles for its hydrate form **3b′**. Arrows and boxes indicate key ^1^H–^13^C long-range correlations that unambiguously characterize the compounds. *J* refers to heteronuclear coupling through 1, 2, 3 or 4 bonds. (**g**) MAIT cell activation. Data represent mean±s.e.m. (*n*=3). The α-dicarbonyls **5a-c**, lumazines **4a-b** and the solvent DMSO-*d*_6_ were inactive (up to 15 μM), while **4c** and **4d** had only very weak activity at the highest concentrations tested (EC_50_>100 nM).

The choice of solvent can significantly alter the outcome of a chemical reaction and dictate whether it is kinetically or thermodynamically controlled^33^,^34^,^35^. In order to obtain the kinetic product *trans*-**3**, the condensation of **5c** with model compound 5-amino-6-methylaminouracil (an analogue of **1**) was investigated by NMR spectroscopy in a polar aprotic solvent (nitromethane-*d*_3_, DMF-*d*_7_ or DMSO-*d*_6_). However, imine formation without simultaneous cyclization was only observed in DMSO-*d*_6_ (Fig. 3a), consistent with it having the largest dielectric constant and an ability to accept hydrogen bond donors. These properties respectively accelerate the cationic E1 dehydration over the cyclization reaction, and suppress imine rehydration through solvation of liberated water molecules by DMSO (Supplementary Fig. 6). The reactions between **1** and **5a-c** in anhydrous DMSO-*d*_6_ were studied by ^1^H and ^13^C NMR spectroscopy (Fig. 3). Formation of **3a-c** was observed clearly within 1 h (for example, Fig. 3b). The reaction of **1** with the unsymmetrical **5c** gave the regioisomer **3c** exclusively, consistent with the greater reactivity of the formyl than the acetyl group in **5c** (Fig. 3a).

Imines **3a-c** were much more stable in DMSO than in water (Fig. 3c and Supplementary Fig. 9), remaining essentially unchanged (>90%) after 2 days in DMSO-*d*_6_ at 22 °C. Thus, by using a common aprotic solvent, the outcome of the reaction was completely diverted to a single kinetic end point without the use of protecting groups, overcoming the substantial challenge posed by the instability of **3a-c** in water where they convert rapidly to lumazines **4a-c** (ref. 36). After extensive optimization of conditions, imine **3c** could even be purified by rpHPLC by minimizing its exposure to water and acid (Fig. 3a and Methods). However, **3a** and **3b** were much less stable in aqueous media and could not be further purified.

Imines **3a-c** were characterized in DMSO-*d*_6_ by 1D and 2D NMR and HRMS (Fig. 3d–f; Supplementary Information). The imines **3a** and **3c** were single, stable species in DMSO. The reaction between **1** and **5b** gave an inseparable mixture of imine **3b**, its formyl hydrate (*gem* diol) and the symmetrical *bis*-imine adduct (as characterized by the imine singlet at *δ* 9.24 p.p.m. and ESI-MS *m/z*=575 [M+H]^+^) in a ratio of 1:1:1.3. Imine **3b** showed characteristic coupling (^3^*J*_HH_=8.0 Hz) between the formyl (9.42 p.p.m.) and imine (8.82 p.p.m.) protons. Meanwhile, its hydrate showed a doublet (^3^*J*_HH_=6.7 Hz) with a chemical shift (δ 5.40 p.p.m.) typical for an acetal proton. All three imines **3a-c** were single geometric isomers, unambiguously characterized by Heteronuclear Multiple Bond Correlation (HMBC) NMR spectra, indicating key connectivities within and between the α-iminocarbonyl unit and the uracil ring (Fig. 3d–f). In **3b** and **3c**, the imine protons (δ 8.81 and 8.70 p.p.m., respectively) showed ^*3*^*J*_HC_ correlations to the ring C-5, with the methyl signal at *δ* 1.82 p.p.m. making the analogous ^4^*J*_HC_ correlation for imine **3a**. The imine protons of **3b** and **3c** also showed ^2^*J*_CH_ correlations to carbonyl carbons (*δ* 194.5 and 200.2 p.p.m., respectively), while the methyl proton signal at *δ* 1.85 p.p.m. of **3a** showed a ^*3*^*J*_HC_ correlation with a carbonyl carbon at *δ* 199.7 p.p.m., proving that cyclization of **3a-c** had not occurred.

The chemical stabilities of **3a-c** were determined in various aqueous buffers (Supplementary Fig. 7). In PBS buffer (pH 8.0, 15 °C), the half-life of **3a** could not be measured as it had completely degraded in <5 min, while **3b** decayed exponentially with a half-life of 18 min. The homologue **3c** was more stable with a half-life of 14 h, consistent with a slower attack on the more hindered carbonyl of **6c** than **6b**. The half-life of **3c** decreased substantially with increasing acidity and temperature. At 15 °C and 0.065 mM, its half-life decreased to 12 h at pH 6.8 (MilliQ water) to 44 min at pH 5.4. It decayed linearly with time at pH 6.8 and pH 8.0, but exponentially at pH 5.4. Under physiological conditions (PBS, pH 7.4, 37 °C), its half-life was 88 min. In aqueous media, **3c** degraded to give exclusively lumazine **4c** without forming isomer **4d** at pH 6.8–8.0.

Next, we evaluated how the 5-OP-RU **3c** formation and degradation kinetics impacted on its preparation and potential use as an immunological reagent. In our previous studies, 5-OP-RU **3c** was prepared by mixing 5-A-RU **1** with methylglyoxal **5c** in aqueous media and then used in immunological studies *in vitro*. However, when 5-A-RU **1** and methylglyoxal **5c** are mixed in PBS buffer (pH 7.4 and 37 °C), 5-OP-RU **3c** reaches a maximum conversion of only 1.1%, before very rapidly degrading with a half-life of 12 min, indicating that the purity of the desired compound generated in the mixture was extremely low (Fig. 3b). In contrast, when 5-A-RU **1** and methylglyoxal **5c** were combined under argon in DMSO-*d*_6_, 5-OP-RU **3c** was formed as the sole product and still intact after 72 h (Fig. 3a,b). Prepared in this way, 5-OP-RU **3c** was stable in DMSO solution (>95%) for days at room temperature. When this DMSO solution of 5-OP-RU **3c** was diluted into aqueous buffer (PBS, pH 7.4, 37 °C) its half-life also increased more than sevenfold (half-life=88 min) (Fig. 3c). Thus, pre-forming 5-OP-RU **3c** in DMSO-*d*_6_ as a kinetic product and then diluting into aqueous buffer yielded a product of substantially higher purity, in greater quantity and with much higher stability than in our previously reported aqueous preparations.

### Comparison of MAIT cell activation by 3a-c

MAIT activation was evaluated using a well established reporter assay^17^ by co-culturing two cell lines (human MAIT TCR-transduced Jurkat T cells and MR1-overexpressed C1R cells, Fig. 3g). Imine **3c** (effector concentration for half-maximum response (EC_50_) 1.6 pM) was the most potent antigen. Although the high-resolution ternary MR1-antigen-TCR crystal structures of **3b-c** were nearly identical^15^, **3b** (EC_50_ 510 pM) was 2 orders of magnitude less potent than **3c**, consistent with its reduced chemical stability in water. Imine **3a** might also be expected to act as an antigen for the MAIT cell mediated detection of microbial infections due to its structural homology to **3b-c** and its role as a putative intermediate in bacterial riboflavin biosynthesis. However, consistent with the inability of mixtures of **1** and **5a** to refold MR1 (ref. 15), imine **3a** (EC_50_ 24 nM) was found to be significantly less active than **3b-c** (Fig. 3g), a result attributable to its extreme instability in water.

### Conformational analysis of 3a-c

**Figure 4:**
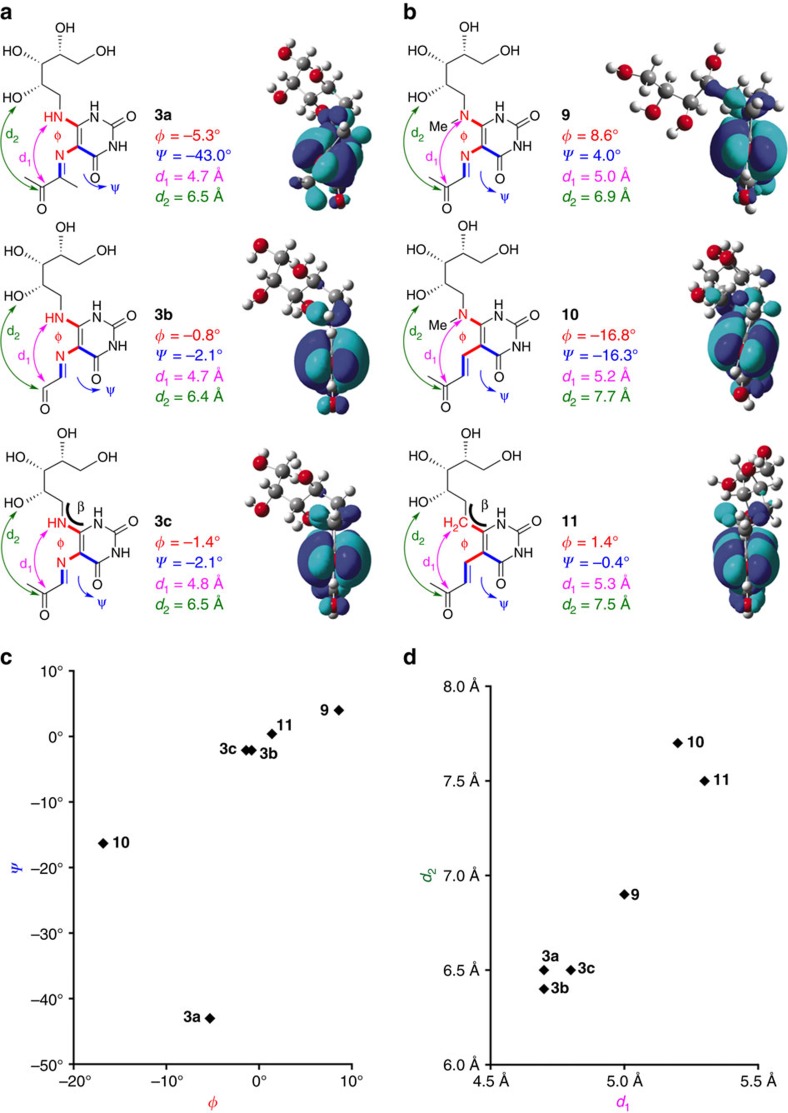
DFT optimized conformations of 3a-c and analogues 9–11. (**a**) Optimized conformers of **3a**–**c** and (**b**) optimized conformers of **9**–**11**, which are analogues of **3c**. Dihedral angles *φ* (red) and *ψ* (blue) define the angles formed by 5,6-substituents centred at uracil C5-C6 and 5-substituent to uracil C5-C4, respectively. In order to visualize the twisting of these substituents, the LUMOs (with orbital phases in cyan and blue) are shown for clarity. The incorporation of a methyl group to **3c** (that is, **3a**, **9** or **10**) resulted in a twisting of the C-5 substituent relative to the ring and an inversion of the LUMO phase at the carbonyl carbon (**3a** and **10**). These changes likely impact the ability of the ligands to form non-covalent interactions with MR1 and MAIT TCR (Fig. 1), as well as their reactivity towards the formation of Schiff base with K43 of MR1. (**c**,**d**) Plots illustrating the structural similarity of the compounds based on the dihedral angles *φ* (red) and *ψ* (blue), and the distances *d*_1_ (purple) and *d*_2_ (green).

To better understand the relative potencies and stabilities of **3a-c**, which have almost identical chemical structures, *ab initio* DFT calculations (at the level of B3LYP/6-311+g(d,p)) were used to predict their optimized structures (Fig. 4a). Compounds **3b-c** showed little deviation from conformations that we observed in crystal structures, with the C-6 nitrogens, uracil rings and iminocarbonyl moieties notably coplanar. This implied that the solution conformations of **3b-c** were likely to be close to their optimal MR1-binding conformation, consistent with MR1 requiring **3b-c** in order to fold into a biologically functional ternary structure, a process that caused little conformational perturbation to the antigens, according to these calculations.

Unexpectedly, **3a** had a significantly different modelled conformation to **3b-c** despite their structural similarity. Due to a steric clash between the imine methyl and the C-4 uracil carbonyl, the iminocarbonyl and the ribityl moieties were twisted out of the uracil ring conjugation plane by −43.0° (*ψ*) and −5.3° (*φ*), respectively. Further, as the imine was twisted out of the plane of ring conjugation, the imine hydration of **3a** (to give **6a**, Fig. 2a) would not disrupt conjugation and was likely to incur a lower energetic penalty than **3b-c**, leading to increased susceptibility to cyclization and imine hydrolysis. The significantly different electronic structures (based on LUMOs) of **3a** and **3b-c** were consistent with a loss of conjugation in **3a** (Supplementary Fig. 8). Together, the inability to adopt the flat conformation needed for binding and the increased imine hydration susceptibility rationalizes the greatly reduced antigenic activity of **3a** relative to **3b-c** and represents a crucial distinction between the productive riboflavin biosynthetic intermediate **3a** and off-pathway by-products of the much more potent antigens **3b** and **3c**. The apparent MAIT activity of **3a** should therefore be cautiously interpreted, but may be attributable to facile hydrolysis to **1**. Under the cell assay conditions, compound **1** can subsequently react with endogenous **5b-c** to give trace **3b-c**. Moreover, the potency of **1**, which can neither refold nor form a Schiff base with MR1, was dependent on the concentration of dicarbonyl **5b-c,** suggesting that its apparent EC_50_ 18 nM was also due to its ability to reform trace amounts of **3b-c**.

### Design and synthesis of a stable T-cell antigen

Having synthesized the unstable but potent antigens **3b-c**, we sought to create a potent antigen that was chemically stable. As the energy-minimized conformations of **3b-c** were similar to those found in their ternary TCR-antigen-MR1 crystal structures^15^ (Fig. 4a), we hypothesized that MAIT cell activation may be related to conformational similarity of an antigen to **3c** in the crystal structure. Thus, analogue **9** was designed from **3c**, in which the nucleophilic C-6 amine was *N*-methylated in order to retard cyclization (Fig. 4b). Then, **10** was designed from **9** by replacing the hydrolytically susceptible imine with an olefin (Fig. 4b). These changes were not expected to affect MR1 binding directly as neither the C-6 nor C-5 nitrogens of **3c** made direct contact with the MR1 protein in the crystal structure^15^ (Fig. 1c). The conformational agreement of **9** and **10** to **3c** was assessed by calculating their energy minimized structures. The iminocarbonyl and the ribityl moieties of **9** were twisted slightly (*φ*=8.6° and *ψ*=4.0°) due to the additional steric bulk at the C-6 amine (Fig. 4b, c). Consistent with this, the distances between the carbonyl carbon and the C-6 amine or the 2′-hydroxyl (d_1_ and d_2_, respectively) were slightly elongated compared to **3c** (Fig. 4b, d). The olefin and ribityl moieties of **10** were significantly more twisted (*φ*=−16.8° and *ψ*=−16.3°) with d_1_ and d_2_ further elongated. Thus, replacing the *N*-methylamine of **10** with the smaller methylene group gave a third analogue (**11**) (Fig. 4b), which was expected to have even greater stability due to the removal of a nucleophile. The energy minimized **11** showed greater conformational similarity to **3c**, with the enone, uracil ring and the C-6 carbon essentially coplanar (Fig. 4b, c). However, the ribityl chain was more distorted, possibly due to a change in hybridization (N-*sp*^2^ to C-*sp*^3^) causing a bond angle decrease (*β*=123.6 to 112.8°) at the ribityl-uracil linkage site.

**Figure 5:**
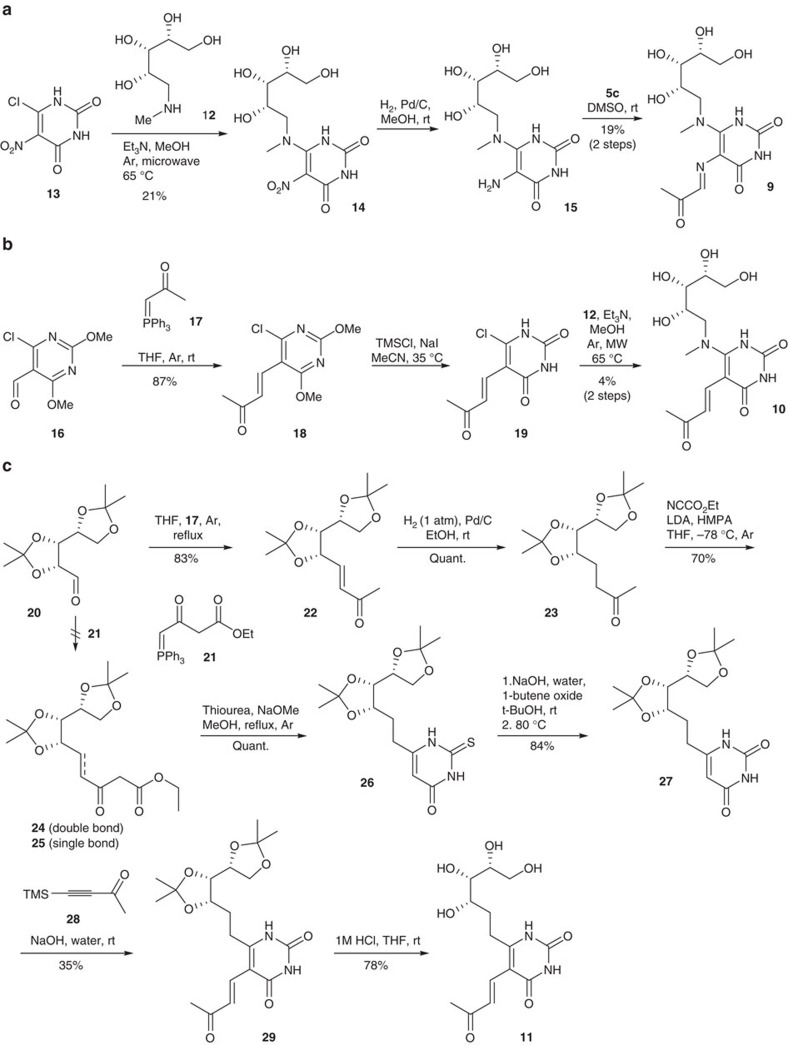
Chemical syntheses of uracil-based analogues of 3c as possible antigens. (**a**) Three step synthesis of **9** from 5-chloro-6-nitrouracil (**13**) and *N*-methylribitylamine (**12**). (**b**) Three step synthesis of analogue **10** from aldehyde **16** via successive Wittig, nucleophilic demethylation and Michael-type substitution reactions. (**c**) Seven step synthesis of **11** from the protected ribose-derived aldehyde **20**. Successive Wittig and hydrogenation reactions on **20**, followed by elaboration with Mander's reagent gave β-ketoester **25**. Subsequent dehydrative cyclization with thiourea, base mediated desulfurization, ring alkylation and acid mediated global deprotection gave analogue **11**.

To assess their antigenic potencies and stabilities, **9–11** were synthesized (Fig. 5). Compound **9** was synthesized via the addition of *N*-methylribitylamine **12** to chloronitrouracil **13** to give **14**, followed by nitro reduction (to give **15**) then condensation with methylglyoxal (Fig. 5a). Compound **10** was synthesized by first introducing the enone substituent via a Wittig olefination on **16** (prepared via an *ortho*-lithiation/formylation sequence), followed by an aza-Michael-type chloride displacement by *N*-methylribitylamine **12** at the doubly activated C-6 position (Fig. 5b). The synthesis of **11** (Fig. 5c) was challenging, as there was almost no precedence for the synthesis of delicately functionalized 5,6-dialkyl uracils. Our initial approach of C-6 functionalization by directed *ortho*-lithiation^37^,^38^ was unsuccessful, presumably due to chelation effects from the substrate's polyoxygenated ribityl chain. Subsequently, we envisaged a construction of the uracil ring from the corresponding ribityl β-ketoester **25**, a key retrosynthetic decision that allowed ribose-derived aldehyde **20** (ref. 39) to be elaborated with a stabilized ylide **21** without α-epimerization. In the forward synthesis, aldehyde **20** was alkylated with ylide **17** under neutral conditions to give **22** (83%). Contrary to the reported reactivity of ylide **21** under neutral^40^,^41^ or basic^42^ conditions, we found that **20** reacted with **21** to give only trace amounts of **24** or led to significant α-epimerization under various conditions. Hydrogenation of **22** gave **23** quantitatively and then α-carboxylation with Mander's reagent^43^ gave β-ketoester **25** (70%). Condensation with thiourea gave thiouracil **26** (quant.)^44^, which was desulfurized unconventionally^45^ under basic conditions with 1-butene oxide to yield **27** (84%), preserving the acetonide protecting groups. After investigating reaction conditions on the model compound 6-methyluracil, the methyl vinyl ketone moiety of **29** was installed in one step with alkyne **28** (35%). Finally, deprotection of **29** with 1 M HCl in THF gave **11** (78%). The structures of **9**, **10** and **11** and their synthetic precursors were unambiguously confirmed by HRMS and NMR (Supplementary Methods and Supplementary Figs 31–58).

### Stability and MAIT cell activation of synthetic analogues

**Figure 6:**
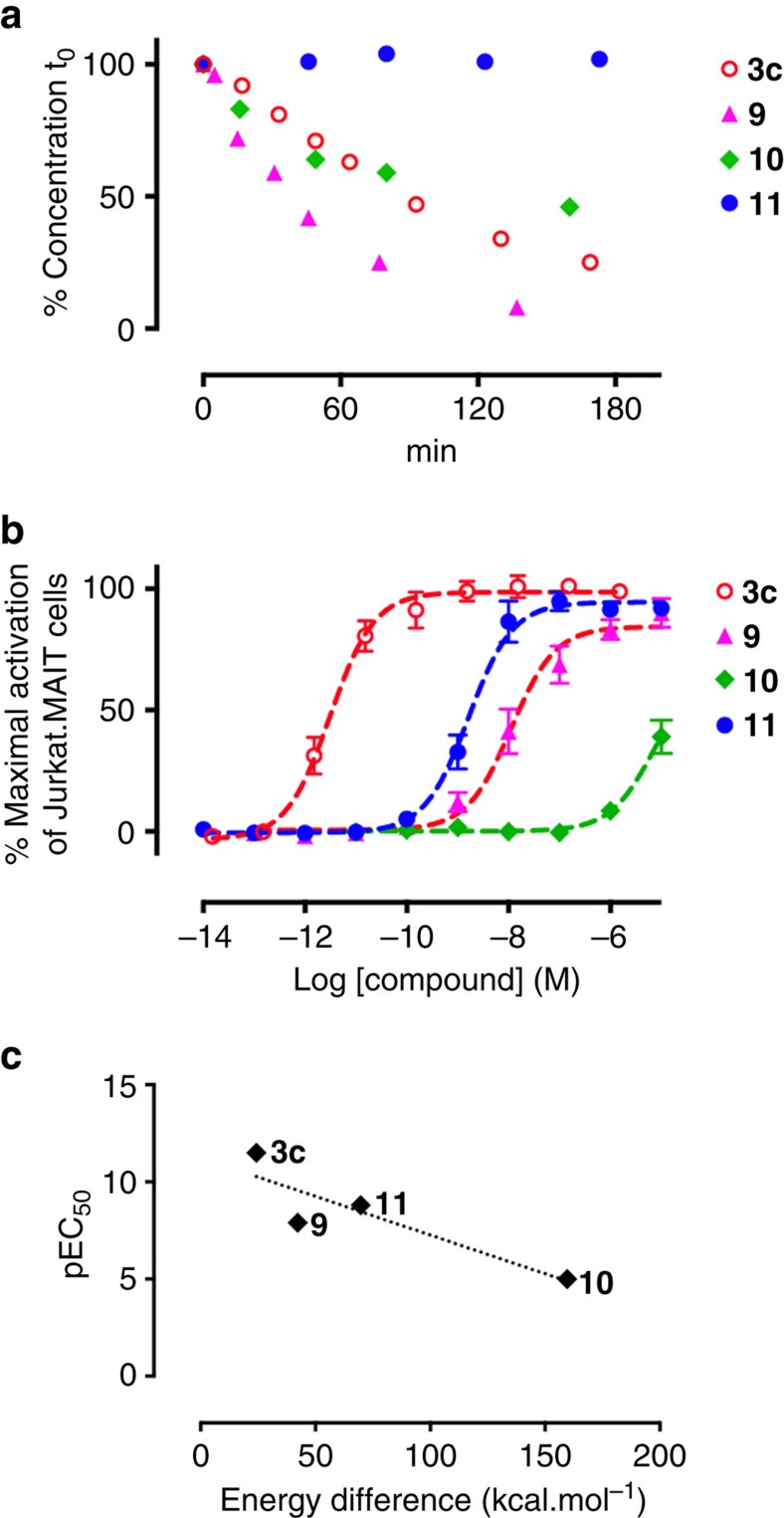
Stability and MAIT cell activation of 9, 10 and 11. (**a**) The aqueous stability of **3c** and **9**–**11** measured by LCMS as a percentage of initial concentration (0.065 mM) over time (37 °C, PBS buffer pH 7.4). (**b**) MAIT cell activation. Data represent mean±s.e.m. (*n*=3). (**c**) Plot of antigen potency (pEC_50_) versus calculated energy difference between the energy minimized conformers and postulated bioactive conformers of **3c** and **9**–**11**. The biologically active conformers of **9**–**11** were created by making the necessary atom connectivity changes to the antigen conformer present in the MR1-**3c**-TCR crystal structure.

The chemical stability and antigenic activities of these analogues were assessed. Despite the additional bulk at the nucleophilic C-6 nitrogen, **9** was less stable than **3c** (Fig. 6a), cyclizing readily to the corresponding quaternary ammonium species. However, as in **3a**, the reduced stability of **9** was consistent with twisting of the iminocarbonyl moiety out of the conjugation plane (*φ*=8.6° and *ψ*=4.0°), likely leading to an increased susceptibility to imine hydration. Although the iminocarbonyl group of enone **10** was even more twisted, it was more stable than **9** as the alkene was more resistant to hydration. Nonetheless, it still degraded to give the corresponding cyclic quaternary ammonium species. Finally, **11** was completely water stable with no degradation observed over 5 days (PBS buffer, pH=7.4, 37 °C).

The antigenic activity of **9** (EC_50_=14±14 nM) was reduced by four orders of magnitude compared to its parent imine **3c**, while the more stable enone analogues **10** and **11** exhibited disparate potencies of >10 μM and 1.6 nM, respectively (Fig. 6b). These relative potencies were consistent with the hypothesis that a better conformational match to **3c**, especially with respect to the dihedral angle *ψ*, would result in greater antigenic potency (Fig. 4c). To support this idea further, the overall conformational distortion of the ligands was quantitatively evaluated, reasoning that the energies expended by **9**–**11** to adopt the ideal bioactive conformation would be related to their antigenic activities. Thus, **9**–**11** were modelled by making the necessary atom connectivity changes to the **3c** conformer bound in the MR1-**3c**-TCR crystal structure. The energies of these ‘bioactive' conformers and the energy-minimized conformers were then calculated using Gaussian. Their differences inversely correlated with their activity (pEC_50_), supporting the conformational matching hypothesis (Fig. 6c). Together, **9**–**11** provide an important base for the delineation of further structure-activity relationships to optimize these antigens and to clarify the molecular determinants of MR1 binding versus MAIT cell activation.

### Functional profiling of analogue 11

**Figure 7:**
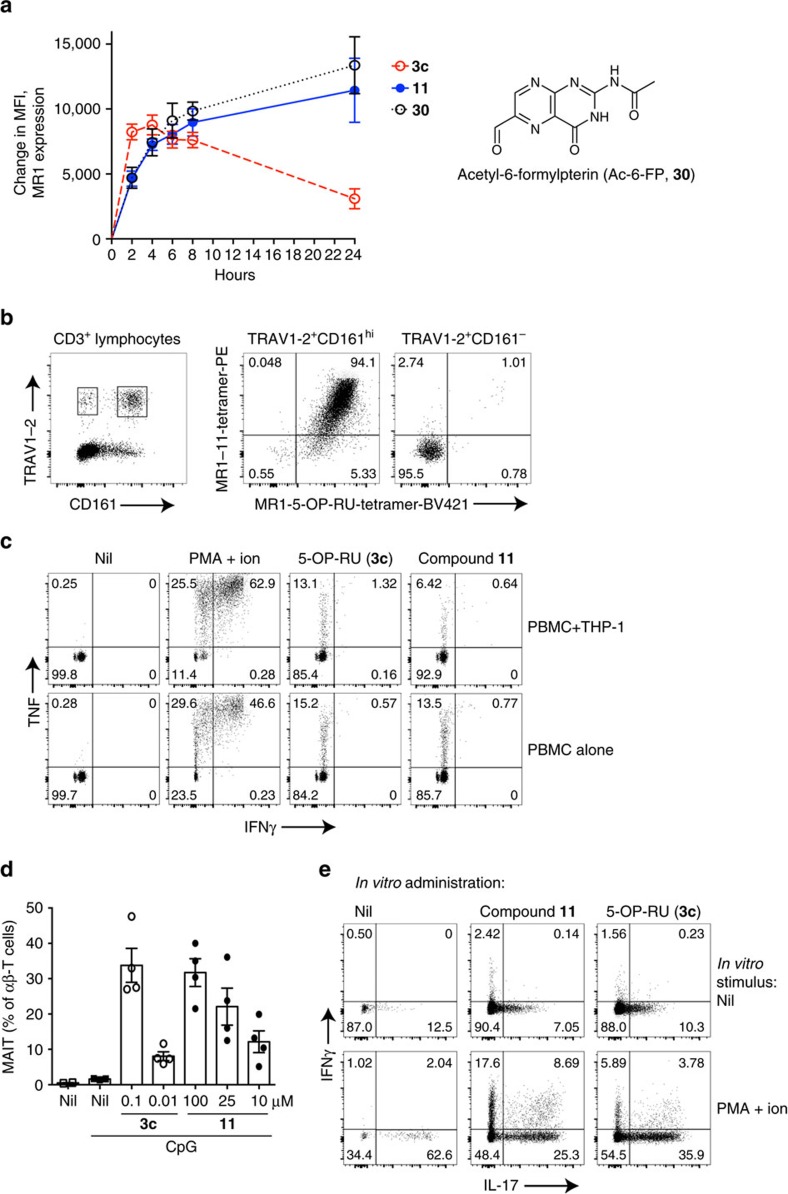
Analogue 11 is functionally similar to 5-OP-RU (3c). (**a**) Upregulation of surface expression of MR1 on C1R.MR1 cells at indicated time points with 10 μM 5-OP-RU **3c**, compound **11** or Ac-6-FP (**30**, structure shown at right). Mean±s.e.m. from three independent experiments (for analogues **9** and **10**, and concentration-response curves of 5-OP-RU **3c** and **11** at defined time points see Supplementary Fig. 9). (**b**) Co-staining of human PBMCs with antibodies to CD3, CD161 and TCR TRAV1–2. Gated CD3^+^ lymphocytes are shown from one representative donor from four. Subsequently gated MAIT (TRAV1-2^+^CD161^hi^CD3^+^) cells and conventional T cells expressing TRAV1–2 (TRAV1-2^+^CD161^hi^CD3^+^) were co-stained with MR1 tetramers from **11** and 5-OP-RU **3c**. See Supplementary Fig. 10 for gating strategy. (**c**) Cytokine profiles after activation of human PBMCs with 5-OP-RU (**3c**, 1.28 nM) and **11** (100 μM). See Supplementary Fig. 11 for gating strategy. (**d**) MAIT cells (TCRβ^+^, MR1-**3c** Tet^+^) as a percentage of αβ-T cells (TCRβ^+^) isolated from the lungs of mice intranasally inoculated with CpG plus 5-OP-RU **3c** or **11** at indicated doses. Day 7 data are shown (Mean±s.e.m.). 4 mice per group. See Supplementary Fig. 12 for gating strategy. (**e**) Cytokine production by MAIT cells harvested from lungs (day 7) of mice inoculated with CpG (day 0) plus 5-OP-RU **3c** (1 μM) or **11** (100 μM) four times on day 0, 1, 2 and 4, detected by intracellular cytokine staining either with or without further stimulation with PMA+ionomycin. One representative mouse (from 4) per group is shown. Experiments were performed twice. See Supplementary Fig. 12 for gating strategy.

To demonstrate the utility of analogue **11** in MAIT cell-related immunological studies, we performed *in vitro* and *in vivo* functional assays. We have previously shown upregulation of MR1 surface expression for **3c** and also for the MAIT-non-stimulatory MR1 ligand acetyl-6-formylpterin (Ac-6-FP, **30**)^21^,^46^. Here we incubated C1R.MR1 cells with a range of concentrations of **3c**, **9**–**11** or **30** over a 24 h time course and detected MR1 expression by staining with the monoclonal antibody 26.5 (**3c**, **11** and **30** shown in Fig. 7a; all compounds and dose titration of **3c** and **11** shown in Supplementary Fig. 9). Compounds **3c** and **11** showed comparable early upregulation of MR1, but the upregulation caused by **3c** peaked at 4 h then reduced, consistent with turnover of MR1 in the cells^46^ and instability of **3c** in aqueous solution. In contrast, MR1 surface expression continued to increase over 24 h in the presence of **11** to a similar level as induced by **30** (ref. 46), consistent with **11** being chemically more stable in water than **3c**.

Next, we examined whether **11** was capable of refolding MR1 for the generation of tetramer reagents. Indeed, human MR1 was readily refolded in the presence of **11** (not shown) and tetramers were generated in parallel to **3c** (that is, MR1-5-OP-RU tetramers^15^,^23^). When used to detect cells within human peripheral blood mononuclear cells (PBMCs) from four healthy blood donors, >94% of TCR TRAV1-2^+^CD161^hi^CD3^+^ cells (MAIT cells) co-stained with both tetramers, but no distinct staining was observed for conventional T cells expressing TRAV1-2 (Fig. 7b).

To assess the ability of **11** to activate MAIT cells, human PBMCs were cultured alone, or with THP-1 cells, in the presence of the compounds, and cytokine production assessed by intracellular cytokine staining. Similar to **3c**, **11** stimulated the production of interferon-γ (IFNγ) and tumour necrosis factor (TNF) by human MAIT cells (Fig. 7c), indicating that it is functionally comparable to **3c**.

Finally, we utilized our established mouse model^47^ to examine the MAIT cell response to analogue **11** *in vivo*. In this model, we previously showed that accumulation of MAIT cells occurs in the lungs after intranasal administration of **3c** and a co-stimulant, such as Toll-like receptor (TLR) agonists. Thus, we used TLR agonist CpG oligodeoxynucleotides with either **3c** or **11** at a range of doses. Both compounds were capable of causing MAIT cell accumulation in the lungs (Fig. 7d), albeit with different efficiencies. Thus, **11** has the same functional profile as **3c** in both *in vitro* and *in vivo* assays examined to date.

## Discussion

MAIT cells are an abundant population of innate-like T cells recognized relatively recently and considered to be involved in many immunological and pathological processes^9^,^48^,^49^,^50^. The identity of the natural antigens was elusive until recently, when we discovered that they were uracil-based imines **3**, formed by a Schiff base condensation between bacterial riboflavin biosynthetic intermediate **1** and the α-dicarbonyl glycolysis metabolites **5** (ref. 15). Unfortunately, antigens **3** are very unstable in water, necessitating the development of a practical synthesis in order to more effectively investigate their immunology and pharmacology. We found that although compounds **3a-c** form only transiently and in small quantities during the reaction between **1** and **5** in water, they were formed exclusively in high yield as long-lived kinetically stable products in DMSO. Importantly, when **3** was isolated from DMSO, it degraded to **4** much more slowly in water than from mixtures of **1** and **5** in water under the same conditions, suggesting a mechanism involving direct conversion of **6** to **4** with very little formation of the immunologically active **3** in water. Taken together, the superior preparation of 5-OP-RU **3c** in DMSO under kinetic control can dramatically improve its utility as an immunological reagent compared to our previously reported aqueous-based preparation (Fig. 3b, c). In addition, this study rationalizes mechanistically and demonstrates experimentally the different aqueous stabilities and potencies (EC_50_ 510 and 1.6 pM, **3b** and **3c** respectively) of these unstable α-iminocarbonyl compounds as MAIT cell antigens. Compounds **9–11** were synthesized as analogues of **3c** in attempts to improve antigen stability, with **11** shown to be the most potent analogue that was completely stable in water. Despite the chemical and structural similarities of **3c** and **11**, their different potencies suggest a very specific conformational requirement for antigen recognition of the MR1-TCR complex. Nevertheless, since **11** shows a similar functional profile to **3c** *in vitro* and *in vivo*, is indefinitely stable in water and easier to handle, it is a potentially useful new reagent for the study of MAIT cells. Furthermore, this study has provided conformational insights for rational design of new MAIT cell antigens and lays the foundation for a better understanding of MAIT cell recognition and activation in immunological responses.

## Methods

General experimental methods for NMR, HPLC and LCMS for monitoring kinetics of formation and stability in aqueous media, and MAIT cell activation assay were conducted as described previously^15^,^17^.

### Materials

Glyoxal, methylglyoxal (both as 40% solutions in water), and butane-2,3-dione (neat) were purchased from Sigma-Aldrich. 5-Amino-6-D-ribitylaminouracil degrades oxidatively in air^31^,^51^, so it was freshly generated under argon in the dark by reduction with sodium dithionite (3 eq) of the corresponding 5-nitroso-6-D-ribitylaminouracil precursor, prepared following a literature procedure^26^ with modifications (Supplementary Information). Methylglyoxal (40% solution in water) degrades with prolonged storage; to minimize this, new commercial samples were aliquoted and stored at −80 °C.

### Synthesis and characterization of α-carbonyl imines 3a-c

A solution of freshly prepared amine **1** was adjusted to pH 7.0 using 1 M NaOH and lyophilized. The powdered **1** (70 mg, containing sodium sulfate from its preparation) was suspended in DMSO-*d*_6_ (1 g) under argon, and then mixed at rt for 1 min. The mixture was filtered through a HPLC syringe filter to remove salts, degassed *in vacuo*, purged with argon, and transferred to an NMR tube prefilled with argon. The concentration was determined by PULCON NMR spectroscopy (typically 50 mM, NMR spectra in Supplementary Data 1)^52^. Glyoxal (2.0 equivalents), methylglyoxal (1.1 equivalent) or butane-2,3-dione (2.0 equivalents) was added, and the reaction monitored by ^1^H NMR. Within 1 h, the reaction was observed clearly and the various unstable α-carbonyl imines **3a** and **3b** were characterized by 1D and 2D NMR (Supplementary Figs 15–21). In the case of compound **3c**, the reaction was allowed to stand at room temperature for 48–72 h to reach completion. The solvent was removed *in vacuo* at room temperature (4 h) with magnetic stirring. The residue was dissolved in 5 ml of 20 mM pH 5.4 aqueous NH_4_OAc and then immediately purified using a Shimadzu preparative HPLC system equipped with a Phenomenex Luna 10 micron C18 250 × 21.2 mm column (P/No 00G-4253-PO-AX) and a SPD-M20A diode array detector (monitored at wavelength λ365 nm). Flow rate was 20 ml min^−1^ with linear gradient: 100% solvent A to 100% solvent B over 30 min where solvent A was 20 mM ammonium acetate pH 5.4 in H_2_O and solvent B was 20 mM ammonium acetate pH 5.4 in MeCN-H_2_O (80:20, v/v). Immediately after the collection of the product peak (9.1 min), the pH of the fraction was adjusted accurately to 8.3 with 0.05% w/w ammonia aqueous solution. The solution was frozen immediately (dry ice/acetone bath) and lyophilized in an acid-free environment. The resultant light purple material was redissolved in milliQ water, then immediately frozen and re-lyophilized. The product was obtained as a yellow powder (31% yield; NMR spectra in Supplementary Figs 20–21).

5-(1-Methyl-2-oxopropylideneamino)-6-(1-D-ribitylamino)uracil (**3a**). ^1^H NMR (600 MHz, DMSO-*d*_6_, key signals only): *δ* 1.83 (s, 3H, N=CMe), 2.40 (s, 3H, MeCO); ^13^C NMR (150 MHz, DMSO-*d*_6_): *δ* 17.2 (N=C*Me*), 24.9 (*Me*CO), 159.8 (N=C), 199.7 (C=O); HRMS (*m*/*z*): [M]^−^ calcd. for C_13_H_19_N_4_O_7_, 343.1259; found, 343.1250.

5-(2-Oxoethylideneamino)-6-(1-D-ribitylaminouracil (**3b**): ^1^H NMR (600 MHz, DMSO-*d*_6_, key signals only): *δ* 8.83 (d, *J*=8.0 Hz, 1H, N=CH), 9.41 (d, *J*=8.1 Hz, 1H, CHO); ^13^C NMR (150 MHz, DMSO-*d*_6_): *δ* 142.0 (N=C), 194.5 (CH=O); HRMS (*m*/*z*): [M]^−^ calcd. for C_11_H_15_N_4_O_7_, 315.0946; found, 315.0944.

5-(2-Oxopropylideneamino)-6-(1-D-ribitylamino)uracil **3c**: ^1^H NMR (600 MHz, DMSO-*d*_6_): *δ* 2.28 (s, 3H, Me), 3.39–3.43 (m, 2H), 3.47–3.51 (m, 1H), 3.52–3.56 (m, 1H), 3.56–3.61 (m, 2H), 3.73 (m, 1H), 7.43 (br s, 1H), 8.80 (s, 1H, N=CH); ^13^C NMR (150 MHz, DMSO-*d*_6_): *δ* 23.5 (Me), 44.1, 63.1, 70.7, 72,8, 72.9, 98.5 (C=N–*C*), 142.0 (br, N=C), 152.1 (br), 157.6 (br), 159.1, 200.2 (C=O); HRMS (*m/z*): [M]^−^ calcd. for C_12_H_17_N_4_O_7_, 329.1103; found, 329.1116.

### Chemical characterization data of compound 11

^1^H NMR (600 MHz, 10% D_2_O/H_2_O): *δ* 1.75–1.81 (m, 1H), 1.92–1.98 (m, 1H), 2.35 (s, 3H), 2.79–2.91 (m, 2H), 3.57–3.61 (m, 2H), 3.66–3.69 (m, 1H), 3.74–3.77 (m, 2H), 7.21 (d, *J*=16.0 Hz, 1H), 7.52 (d, *J*=16.0 Hz, 1H); ^13^C NMR (150 MHz, 10% D_2_O/H_2_O): *δ* 27.0, 27.2, 30.1, 62.9, 70.7, 72.4, 74.3, 105.9, 127.8, 136.4, 151.9, 161.3, 165.0, 205.2; HRMS (*m/z*): [M]^+^ calcd. for C_14_H_21_N_2_O_7_, 329.1343; found, 329.1348.

### Stability of compounds **3a-c** and 9–11 in aqueous media

Stock solutions of either crude (**3a**, **3b** and **9**) or purified compounds (**3c, 10** and **11**) in DMSO-*d*_6_, with a known concentration calibrated using the PULCON NMR technique^52^, were dissolved in aqueous buffers, such as TBS buffer (10 mM Tris, 150 mM NaCl, pH 8.0), PBS (pH 7.4), MilliQ water (pH 6.8), or aqueous ammonium acetate buffer (20 mM, pH 5.4, 150 mM NaCl). Consumption of **3** was monitored by LCMS. The degradation curves and half-lives under various conditions were summarized in Fig. 6a and in Supplementary Fig. 7.

### MAIT reporter cell activation assay

MAIT reporter cell activation assays and MR1 upregulation assays were performed by slight modifications to reported methods^17^. Briefly, Jurkat cells transduced with genes encoding a MAIT TCR comprising the TRAV1-2-TRAJ33 invariant α chain, and a TRBV6-1 β chain, were tested for activation by co-incubation with compounds and C1R antigen-presenting cells expressing MR1 (CIR.MR1) for 16 h or over a time course as indicated. Cells were subsequently stained with PE-Cy7-conjugated anti-CD3 (UCHT1, eBioscience, 1:300), and APC-conjugated anti-CD69 (BD, 1:25) antibodies as well as biotinylated anti-MR1 mAb 26.5, a gift from Dr Ted Hansen, Washington University School of Medicine, St Louis, MO^53^, followed by Streptavidin-PE (BD, 1:1,000), before analysis by on a FACS CantoII (BD) flow cytometer. Activation of Jurkat.MAIT was measured by an increase in surface CD69 expression. MR1 expression was detected on gated C1R.MR1 cells in the same assay.

### Generation of MR1–11 and MR1-5-OP-RU tetramers

MR1 refolding was performed by slight modifications to reported methods^17^. Human MR1-5-OP-RU **3c**, human MR1–11 tetramers, and mouse MR1-5-OP-RU tetramers were prepared by known methods^15^. Genes encoding soluble human MR1 or human β2m were expressed for 4 h in BL21 *E. coli* following induction with 1 mM isopropyl β-D-1-thiogalactopyranoside. *E. coli* were pelleted and resuspended in a buffer containing 50 mM Tris, 25% (w/v) sucrose, 1 mM EDTA, 10 mM DTT pH 8.0. Inclusion body protein was then extracted by lysis of bacteria in a buffer containing 50 mM Tris pH 8.0, 1% (w/v) Triton X-100, 1% (w/v) sodium deoxycholate, 100 mM NaCl, 10 mM DTT, 5 mM MgCl_2_ and 1 mg DNaseI per litre of starting culture; and subsequent steps involved homogenization with a polytron homogenizer, centrifugation, and washing inclusion body protein sequentially with firstly a buffer containing 50 mM Tris pH 8.0, 0.5% Triton X-100, 100 mM NaCl, 1 mM EDTA, 1 mM DTT, and secondly a buffer containing 50 mM Tris pH 8.0, 1 mM EDTA, 1 mM DTT. Inclusion body protein was then resuspended in a buffer containing 20 mM Tris pH 8.0, 8 M urea, 0.5 mM EDTA, 1 mM DTT and following centrifugation the supernatant containing solubilized, denatured inclusion body protein was collected and stored at −80 °C.

C-terminal cysteine-tagged MR1 and β2m were refolded with ligand essentially as described^15^. Briefly, in order to generate MR1-5-OP-RU and MR1–**11**, 28 mg of MR1 and 14 mg of β2m inclusion body proteins were refolded with 5-A-RU (5.25 μmol) and methylglyoxal (1.77 mmol), or compound **11** (1.92 μmol), respectively. Refolded MR1-Ag was then purified by sequential DEAE (GE Healthcare) anion exchange, S75 16/60 (GE Healthcare) gel filtration, and MonoQ (GE Healthcare) anion exchange chromatography. Cysteine-tagged-MR1-5-OP-RU or MR1–**11** were then reduced with 5 mM DTT for 20 min prior to buffer exchange into PBS using a PD-10 column (GE Healthcare), and biotinylated with Maleimide-PEG2 biotin (Thermoscientific) with a 30:1 molar ratio of biotin:protein at 4 °C for 16 h in the dark. Biotinylated MR1 was subjected to S200 10/300 GL (GE Healthcare) chromatography to remove excess biotin. Biotinylated, MR1-5-OP-RU or MR1–11 monomers were tetramerized with streptavidin conjugated to either PE (SA-PE) or Brilliant Violet 421 (SA-BV) (BD Pharmingen).

### Tetramer staining of human PBMCs

Healthy donor blood packs were obtained through the Australian Red Cross Blood Service after approval from the Melbourne University Human Research Ethics Committee and PBMCs were prepared by separation on Ficoll–Paque Premium (GE Healthcare). Cells were then washed twice prior to resuspension in 10% DMSO in FCS. Prior to use, PBMCs were stored in liquid nitrogen. Approximately 2 × 10^6^ cells were stained with human MR1-5-OP-RU tetramer (BV421), human MR1–**11** tetramer (PE) and monoclonal antibodies to CD3 (AlexaFluor700, OKT3, eBioscience, 1:50), CD161 (PE-Cy7, HP-3G10, Biolegend, 1:50) and TRAV1–2 (APC, clone 3C10, Biolegend, 1:50) for 30 min on ice and fixed in 1% paraformaldehyde before acquisition of data on a BD LSR-Fortessa. Data were analyzed using FlowJo software (Tree Star).

### *In vitro* PBMC stimulation assay

Stimulation of PBMCs was performed essentially as previously described^17^. Briefly, 5 × 10^5^ PBMCs were cultured, alone or with 1 × 10^5^ THP-1 cells, overnight with or without compounds **3c** or **11** at a range of doses or with PMA and ionomycin (5 ng ml^−1^ and 1 μg ml^−1^ final concentrations, respectively) as a positive control. BD GolgiStop (1:1,000 dilution) was added after the first hour of stimulation. Cells were stained for surface markers using human MR1-5-OP-RU tetramer (PE, 1:300), and monoclonal antibodies to CD3 (PE-CF594, UCHT1, BD Horizon, 1:50), CD161 (PE-Cy7, HP-3G10, Biolegend, 1:50) for 30 min on ice and fixed in 1% paraformaldehyde before cytokine production by MAIT cells was detected by intracellular cytokine staining with monoclonal antibodies to TNF (APC, MAb11, eBioscience, 1:50) and IFNγ (AlexaFluor700, B27, BD 1:50) in the presence of 0.3% saponin. Data was acquired on a BD LSR-Fortessa and analysed using FlowJo software (Tree Star).

### *In vivo* assay

Mice were bred and housed at the Biological Research Facility of the Peter Doherty Institute (Melbourne, Victoria, Australia). Male adult (6–10 weeks) C57BL/6 mice were used for all experiments, after approval by the University of Melbourne Animal Ethics Committee. CpG 1688 sequence: 5′-T*C*C*A*T*G*A*C*G*T*T*C*C*T*G*A*T*G*C*T-3′ (*phosphorothioate linkage) nonmethylated cytosine-guanosine oligonucleotides was purchased from Geneworks (Australia). Mice were inoculated with 20 μg CpG once per nares, plus **3c** or **11** at indicated doses and MAIT cells were detected from lungs at day 7 as previously described^47^. Cytokine production by isolated MAIT cells was detected by intracellular cytokine staining^47^. Briefly, mice were sacrificed by administration of CO_2_ and lungs taken following heart perfusion with 10 ml cold RPMI. To prepare single cell suspensions, lungs were finely chopped with a scalpel blade and treated with 3 mg ml^−1^ collagenase III (Worthington), 5 μg ml^−1^ DNAse and 2% FCS in RPMI for 90 min at 37 °C with gentle shaking. Cells were then filtered (70 μM) and washed with PBS/2% FCS. RBCs were lysed with hypotonic buffer TAC (Tris-based Amino Chloride) for 5 min at 37 °C. Approximately 1.5 × 10^6^ cells were filtered (40 μm) and used for flow cytometric analysis.

Ab against CD19 (1D3, PerCP-Cy 5.5, 1:200), CD45.2 (104, FITC, 1:200), IFNγ (XMG1.2, PE-Cy7, 1:400), TCRβ (H57–597, APC or FITC, 1:200) and IL-17A (TC11-18H10, PE, 1:200) were purchased from BD. To block non-specific staining, cells were incubated with MR1-6FP tetramer and anti-Fc receptor (2.4G2) for 15 min at room temperature, then incubated at room temperature with antibody/tetramer cocktails in PBS/2% FCS. 7-AAD (5 μl per sample) was added for the last 10 min. Cells were fixed with 1% PFA prior to analysis on LSR Fortessa (BD Bioscience) flow cytometer. Data analysis was performed with FlowJo software. For intracellular cytokine staining (ICS), Golgi plug (BD Biosciences) was used during all processing steps. Cells stimulated with PMA/Ionomycin (20 ng ml^−1^, 1 μg ml^−1^ respectively) for 3 h at 37 °C were included as positive controls. FVD (eBioscience) were added for 30 min at 4 °C before surface staining. Surface staining was performed at 37 °C and cells stained for intracellular cytokines using the BD Fixation/Permeabilisation kit or transcription factors using the transcription buffer staining set (eBioscience) according to the manufacturers' instructions.

### Data availability

The authors declare that the data supporting the findings of this study are available within the paper and its Supplementary Information files. FIDs of the NMR spectra are available from the authors upon request.

## Additional information

**How to cite this article:** Mak, J. Y. W. *et al*. Stabilizing short-lived Schiff base derivatives of 5-aminouracils that activate mucosal-associated invariant T cells. *Nat. Commun.* **8,** 14599 doi: 10.1038/ncomms14599 (2017).

**Publisher's note:** Springer Nature remains neutral with regard to jurisdictional claims in published maps and institutional affiliations.

## Supplementary Material

Supplementary Information Supplementary Figures, Supplementary Methods and Supplementary References
