# Supplementary material for: Stabilizing short-lived Schiff base derivatives of 5-aminouracils that activate mucosal-associated invariant T cells
Source: Nat Commun. 2017 Mar 8;8:14599. doi: 10.1038/ncomms14599 (PMC5344979; doi:10.1038/ncomms14599)
Supplement: Supplementary Information — Supplementary Figures, Supplementary Methods and Supplementary References [file ncomms14599-s1.pdf]

Supplementary Methods

General. Anhydrous tetrahydrofuran was dispensed from an Innovative Technology PureSolv solvent purification system. Anhydrous methanol was prepared by refluxing the methanol with magnesium (with catalytic iodine), and then distilling the resultant methanol/magnesium methoxide mixture under argon. Anhydrous acetonitrile was prepared by refluxing and then distilling from calcium hydride under argon. Anhydrous hexamethylphosphoramide was prepared by vacuum distillation from calcium hydride. Lithium diisopropylamide solution was purchased and then titrated with *N*-benzylbenzamide[1] before use. All other chemicals were used as purchased without further purification. HPLC and LCMS mobile phases were defined as follows (unless specifically defined): solvent A was 20 mM ammonium acetate in MilliQ H_2O, and solvent B was 20 mM ammonium acetate in MeCN–H_2O (80:20, v/v). Analytical HPLC was performed using an Agilent 1200 Series with a diode-array detector on a Phenomenex Luna 5 μm, C18(2) 250 × 4.60 mm column. The flow rate was 1 mL/min with a linear gradient: isocratic 100 % solvent A over 20 min, then 0 % to 5 % solvent B gradient over 10 min, isocratic 5% solvent B over 20 min, 5% to 100% solvent B gradient over 5 min, and then a further 5 min at isocratic 100% solvent B. LCMS analysis (15 min run) was carried out on a Shimadzu LCMS-2020 system equipped with a Phenomenex Luna 3 micron C18(2) 150 × 2.00 mm column (P/No 00F-4251-BO) and a SPD-M20A diode array detector. The temperatures for the sample and column chambers were 15 °C and 40 °C, respectively. The flow rate was 0.4 mL/min with a linear gradient: isocratic 100 % solvent A over 5 min, then 0 % to 50% solvent B gradient over 5 min, and then a regeneration phase of a 100 % to 0 % solvent B gradient over 1 min and then isocratic 100 % solvent A over 4 min. Electrospray ionisation high-resolution mass spectra (ESI-HRMS) measurements were obtained on a Bruker micrOTOF mass spectrometer equipped with an Agilent 1100 Series LC/MSD mass detector in either negative or positive ion mode by direct infusion in water at 100 μL/h using sodium formate clusters as an internal calibrant. ^{1}H and ^{13}C NMR spectra were recorded on Bruker Avance 600 spectrometers at 298 K in the solvents indicated and referenced to tetramethylsilane or residual solvent signals in the deuterated solvents (^{1}H: δ 4.79 for D_2O, $^{1}H/^{13}C$: δ 0.00/77.0 for $CDCl_3$ [containing tetramethylsilane] δ 2.50/39.5 for DMSO-d_6 and δ 3.31/49.0 for CD_3OD). Proton and carbon assignments were determined by various 1D and 2D NMR experiments (JMOD, gCOSY, gHSQC, gHMBC, NOESY).

Synthesis of 5-Amino-6-D-ribitylaminouracil (1)

The nitroso precursor 5-nitroso-6-D-ribitylaminouracil was prepared following a literature procedure[2] with modifications. A mixture of D-(–)-ribose (5.00 g, 33.3 mmol) and benzylamine (3.64 mL, 33.3 mmol, 1 eq) in anhydrous methanol (100 mL) was stirred with freshly activated

molecular sieves 4 Å (0.5 g, 3.2 mm pellets) at room temperature under nitrogen overnight. The mixture was transferred into a Parr hydrogenation vessel. The mixture was degassed with nitrogen, platinum oxide (0.2 g) was added, and the mixture shaken under hydrogen (≤ 15 psi) for 7 h. For larger scale reactions, stirring the above mixture in a round bottom flask under hydrogen (1 atm) gave improved yields. ESI-MS indicated the complete conversion to *N*-benzyl-1-deoxy-1-D-ribtylamine (*m/z* 242, $M+H^+$). The mixture was filtered through a celite pad and the solid rinsed with methanol (5 mL). The combined filtrate and rinse fractions were transferred into a Parr hydrogenation vessel and degassed with nitrogen. To this mixture was added 10% Pd/C (0.5 g), and then shaken under hydrogen (≤ 10 psi) at room temperature overnight. The mixture was filtered through a celite pad and the solid rinsed with methanol (5 mL). The combined filtrate and rinse fractions were concentrated by rotary evaporation and then pumped to dryness to give crude 1-deoxy-1-D-ribtylamine as light brownish syrup. ESI-MS: 152 ($M+H^+$).

To a solution of the above syrup in water (28 mL) in a round-bottom flask was added 6-chlorouracil (4.07 g, 27.8 mmol, 0.83 eq). Nitrogen was bubbled into the mixture with stiring at 100 ºC (external oil bath temperature) for 5 minutes. The flask was sealed with a septa under nitrogen and the mixture stirred at 100 ºC while *N,N*-diisopropylethylamine (4.84 mL, 27.8 mmol) was added slowly using a syringe pump over 1 h. After addition, the mixture was bubbled with nitrogen at 100 ºC for 1 h and then pumped to dryness by rotary evaporation. The residue (light brownish syrup) was heated at 110 ºC (bath temperature) under high vacuum for 2 h, cooled to room temperature under nitrogen to give crude 6-D-ribitylaminouracil as light brownish gum. ESI-MS: 262 ($M+H^+$).

The above gum was dissolved in water (160 mL) and sodium nitrite (5.75 g, 83.3 mmol, 3 eq) was added in one portion. The mixture was stirred at room temperature (pH = 5.53, pH meter) while pH was adjusted by dropwise addition of acetic acid, during which a cloud of orange gas started to evolve (pH = 4.60). The mixture was stirred and the pH maintained at 4.60 for 2.5 h, checking the pH every 30 minutes. The mixture was then poured into 0.1 M ammonia solution (800 mL). The resultant red solution was loaded onto an anionic exchange column chromatography (Dowex 1 × 8 200–400 mesh, formate form). The column was washed (by gravity) with DI-water (2 L) and gradient formic acid buffers (0.01 M, 1 L; 0.02 M, 2 L; 0.03 M, 1 L; 0.05 M, 1 L). A pink-red band started to elute with 0.03 M formic acid and fully eluted with the 0.05 M formic acid buffer. The column was regenerated by washing sequentially with 0.1 M formic acid buffer (1 L), 1 M ammonium formate (pH = 6.78, 1 L) and DI-water (3 L). The pink-red fractions (ESMS: 291, $M+H^+$) were combined and then evaporated to a small volume (~35 mL). After standing at room temperature overnight, the pink-red crystals were filtered, rinsed with water (5 mL) and then dried

under high vacuum overnight (3.28 g). The filtrate was lyophilised to give a second crop of the product as a red powder (0.57 g, overall yield 3.85 g, 40% from ribose). Alternatively, the combined eluted fractions could be lyophilised directly to give spectroscopically identical material. To the best of our knowledge, shown below is the first NMR characterisation of this known compound.

^{1}H NMR (600 MHz, DMSO-d_6–D_2O 10:1): 3.36–3.40 (m, 2H, ribityl-3'-CH and 1 × H of ribityl-5'-CH_2), 3.44 (dd, J = 14.2, 7.2 Hz, 1H, 1 × H of ribityl-1'-CH_2), 3.47 (td, J = 6.4, 3.3 Hz, 1H, ribityl-2'-CH), 3.56 (dd, J = 11.2, 3.1 Hz, 1H, 1 × H of ribityl-5'-CH_2), 3.59 (dd, J = 14.3, 3.2 Hz, 1H, 1 × H of ribityl-1'-CH_2), 3.76–3.80 (m, 1H, ribityl-4'-CH); ^{13}C NMR (150 MHz, DMSO-d_6): 161.0 (C), 149.6 (C), 146.9 (C), 137.9 (C), 72.7 (CH), 72.6 (CH), 69.0 (CH), 63.1 (CH_2), 43.6 (CH_2); ESI-MS (m/z): 291 [M+H$^+$].

5-Amino-6-D-ribitylaminouracil (**1**) was prepared by modifications to a literature procedure.[2] The nitroso precursor (558 mg, 1.92 mmol, 1 eq) was dissolved in degassed MilliQ water (15 mL) at 80 °C under argon. To the red solution was added sodium dithionite powder in portions (total 1.00 g, 5.77 mmol, 3.0 eq) under argon. The colour changed instantly to pale-yellow. After stirring at 80 ºC for 5 min, the solution was cooled under argon in an ice-water bath. An aliquot was taken and the pH adjusted as required using 1 M HCl or NaOH aqueous solutions (see NMR spectra in appendix), then diluted with MilliQ water to make 50 mM stock solutions for kinetic studies, synthesis of lumazines, or stored in 1.5 mL aliquots at –20 °C. For kinetic studies by LCMS in various aqueous buffers and biological studies, 50 mM pH 7.0 solutions were used. For the synthesis of α-carbonyl imines **3a–c** in DMSO, a pH 7.0 stock solution was lyophilised to powder. Note that the solutions contain sodium sulfate (150 mM, i.e. 3 eq), derived from the oxidative degradation of sodium dithionite. This can be removed by dissolving the material in DMSO-d_6 followed by filtration.

To the best of our knowledge, shown below is the first NMR characterisation of this known compound. In particular, its ^{1}H NMR spectrum was measured in water at varying pH to facilitate the study of its reaction with α,β-dicarbonyls by NMR spectroscopy (Appendix). A representative water suppression Watergate NMR spectrum[3] is given below.

^{1}H NMR (600 MHz, H_2O:D_2O, 9:1, v/v, pH = 4.8, residue water signal at 4.79 ppm): δ 3.50 (dd, J = 14.8, 7.4 Hz, 1H, 1 × H of ribityl-1'-CH_2), 3.62 (dd, J = 14.8, 2.8 Hz, 1H, 1 × H of ribityl-1'-CH_2), 3.67 (dd, J = 11.8, 6.7 Hz, 1H, 1 × H of ribityl-5'-CH_2), 3.72 (m, 1H, ribityl-3'-CH), 3.80 (dd, J =

11.8, 3.1 Hz, 1H, 1 × H of ribityl-5'-CH_2), 3.83 (m, 1H, ribityl-4'-CH), 3.96 (ddd, *J* = 7.4, 6.2, 2.8 Hz, 1H, ribityl-2'-CH). ^{1}H NMR (600 MHz, DMSO-d_6): δ 3.21–3.27 (m, 1H, 1 × H of ribityl-1'-CH_2), 3.35–3.48 (m, 3H, ribityl-3' or 4'-CH, 1 × H of ribityl-1'-CH_2 and 1 × H of ribityl-5'-CH_2), 3.52 (br s, 1H, ribityl-3' or 4'-CH), 3.58 (d, *J* = 10.2 Hz, 1H, 1 × H of ribityl-5'-CH_2), 3.69 (br s, 1H, ribityl-2'-CH); ^{13}C NMR (150 MHz, DMSO-d_6,): δ 44.4 (CH_2, ribityl-1'-CH_2), 63.1 (CH_2, ribityl-5'-CH_2), 71.4 (CH, ribityl-2'-CH), 72.6 (CH) and 72.8 (CH, ribityl-3' and 4'-CH), 94.3 (C, 5), 147.2 (C), 149.6 (C), 161.1 (C); ESI-MS (m/z): 277 [M+H$^+$].

NMR monitoring of the reaction between 5-A-RU and α,β-carbonyls 5a-c to form lumazines. As there were few proton signals that could be used to monitor the formation of the intermediates and the cyclised lumazines, the mixed NMR solvent system H_2O: D_2O (9:1, v/v) was used to minimise the disappearance of exchangeable protons such as NH and 7-methyl in lumazines,[4,5] using the watergate NMR technique to suppress the water peak.[3] All solvents were purged with argon and the NMR tubes prefilled with argon. Stock solutions of **1** (50 mM containing 150 mM Na_2SO_4, 30 μL, 1.5 μmol) at various pH (4.8, 5.8, 6.8, and 8.0) and solutions of α,β-dicarbonyls butane-2,3-dione (**5a**), glyoxal (**5b**), and methylglyoxal (**5c**) (50 mM solution) were prepared.

The ^{1}H NMR spectrum of each starting material was measured by using 540 μL of the aqueous stock and 60 μL of D_2O (total volume 600 uL, concentration 45 mM). Aliquots (300 μL each) of the amine **1** and the α,β-dicarbonyl solutions were mixed in a new NMR tube (final concentration: 22.5 mM, molar ratio of 1:1). The mixture was vortexed, and the ^{1}H NMR spectra at the 5 min time point was recorded, before storing at 4 °C or room temperature in the dark. The reaction was then monitored by NMR spectroscopy until completion at half-hour then 1 h intervals for the first 6 h, and then every 24 hours for 1–7 days.

Aliquots (300 μL each) of amine **1** and the α,β-dicarbonyl solutions were mixed and sealed in a glass vial (final concentration: 22.5 mM, molar ratio of 1:1), and then heated with stirring in the dark at 80 °C for 30 min, monitoring the reaction progress by NMR. By this method, the optimal conditions for lumazine formation were determined for compounds 6,7-dimethyl-8-(1′-D-ribityl)lumazine (**4a**, rt, 7 days or 80 ºC, 30 min), 8-(1′-D-ribityl)lumazine (**4b**, 4 ºC, 4–7 days), and 7-dimethyl-8-(1′-D-ribityl)lumazine (**4c**, 4 ºC, 1 day).

Preparative scale synthesis of the lumazines was carried out on a 0.2 mmol scale with 3 equivalents of the α,β-dicarbonyls under the optimised conditions. The optimal conditions for formation of the

6-dimethyl-8-(1'-D-ribityl)lumazine (**4d**) regioisomer was to carry out the reaction in TBS buffer at higher dilution (2.6 mM rather than 22.5 mM, 4 ºC, 1 day). The crude product was diluted with aqueous 20 mM ammonium acetate buffer (pH 5.4), and then purified using preparative rpHPLC with a linear gradient: isocratic 100 % solvent A over 20 min, then 0 % to 5 % solvent B gradient over 10 min, 5 % to 100 % solvent B gradient over 20 min and then a further 10 min at isocratic 100% solvent B. The pure product fractions were combined and lyophilised. The resultant powder was re-dissolved in MilliQ water and re-lyophilised to remove residual ammonium acetate to give the product as a yellow or green powder. Compounds were fully characterised by 1D and 2D NMR spectroscopy. (Supplementary Figures)

8-(1'-D-Ribityl)lumazine (**4b**). ^{1}H NMR (600 MHz, D_2O–CD_3OD 9:1), δ 3.69 (dd, J = 11.9, 6.8 Hz, 1H, 1 × H of ribityl-5′-CH_2), 3.79 (dd, J = 6.6, 5.2 Hz, 1H, ribityl-3′-CH), 3.82 (dd, J = 11.9, 3.1 Hz, 1H, 1 × H of ribityl-5′-CH_2), 3.91 (td, J = 6.8, 3.1 Hz, 1H, ribityl-4′-CH), 4.24–4.31 (m, 2H, ribityl-2′-CH and 1 × H of ribityl-1′-CH_2), 5.10 (d, J = 11.3 Hz, 1H, 1 × H of ribityl-1′-CH_2), 8.36 (d, J = 3.8 Hz, 1H, H-6), 8.43 (d, J = 3.8 Hz, 1H, H-7); ^{13}C NMR (150 MHz, D_2O–CD_3OD 9:1): δ 57.2 (CH_2, ribityl-1′-CH_2), 63.5 (CH_2, ribityl-5′-CH_2), 69.3 (CH, ribityl-2′-CH), 72.9 (CH, ribityl-4′-CH), 74.1 (CH, ribityl-3′-CH), 133.9 (CH, C-6), 138.0 (C, C-4a), 140.5 (CH, C-7), 152.5 (C, C-8a), 159.0 (C, C-2), 163.3 (C, C-4); HRMS (m/z): $[M]^+$ calcd. for $C_{11}H_{15}N_4O_6$, 299.0986; found, 299.0983; HPLC t_R = 7.8 min.

7-Methyl-8-(1'-D-ribityl)lumazine (**4c**). ^{1}H NMR (600 MHz, D_2O–CD_3OD 9:1): δ 2.90 (s, 3H, 7-CH_3), 3.73 (dd, J = 11.9, 6.7 Hz, 1H, 1 × H of ribityl-5′-CH_2), 3.87 (dd, J = 11.9, 2.9 Hz, 1H, 1 × H of ribityl-5′-CH_2), 3.89 (dd, J = 7.1, 5.0 Hz, 1H, ribityl-3′-CH), 3.95 (td, J = 6.9, 3.0 Hz, 1H, ribityl-4′-CH), 4.46 (ddd, J = 9.5, 4.8, 2.5 Hz, 1H, ribityl-2'-CH), 4.62 (dd, J = 13.2, 10.3, 1H, 1 × H of ribityl-1′-CH_2), 4.97 (dd, J = 13.2, 1.9 Hz, 1H, 1 × H of ribityl-1′-CH_2), 8.36 (s, 1H, H-6); ^{13}C NMR (150 MHz, D_2O–CD_3OD 9:1): δ 24.2 (CH_3, 7-CH_3), 52.2 (CH_2, ribityl-1′-CH_2), 63.6 (CH_2, ribityl-5′-CH_2), 69.7 (CH, ribityl-2′-CH), 73.0 (CH, ribityl-4′-CH), 74.4 (CH, ribityl-3′-CH), 134.3 (C, C-4a), 136.6 (CH, C-6), 152.7 (C, C-8a), 153.5 (C, C-7), 159.0 (C, C-2), 163.8 (C, C-4); HRMS (m/z): $[M]^+$ calcd. for $C_{12}H_{17}N_4O_6$, 313.1143; found, 313.1140; HPLC t_R = 15.0 min.

6-Methyl-8-(1'-D-ribityl)lumazine (**4d**). ^{1}H NMR (600 MHz, D_2O–CD_3OD 9:1), δ 2.61 (s, 3H, 7-CH_3), 3.69 (dd, J = 12.1, 6.9 Hz, 1H, 1 × H of ribityl-5′-CH_2), 3.78 (t, J = 5.5 Hz, 1H, ribityl-3′-CH), 3.82 (dd, J = 12.0, 2.3 Hz, 1H, 1 × H of ribityl-5′-CH_2), 3.91 (td, J = 7.1, 2.5 Hz, 1H, ribityl-4′-CH), 4.23–4.31 (m, 2H, ribityl-2′-CH and 1 × H of ribityl-1′-CH_2), 5.07 (d, J = 11.8 Hz, 1H, 1 ×

H of ribityl-1′-CH_2), 8.38 (s, 1H, H-7); ^{13}C NMR (150 MHz, D_2O–CD_3OD 9:1): δ 20.4 (CH_3, 6-CH_3), 57.3 (CH_2, ribityl-1′-CH_2), 63.5 (CH_2, ribityl-5′-CH_2), 69.5 (CH, ribityl-2′-CH), 72.9 (CH, ribityl-4′-CH), 74.0 (CH, ribityl-3′-CH), 136.4 (C, C-4a), 139.7 (CH, C-7), 144.7 (C, C-6), 150.7 (C, C-8a), 158.9 (C, C-2), 163.3 (C, C-4); HRMS (m/z): $[M]^+$ calcd. for $C_{12}H_{17}N_4O_6$, 313.1143; found, 313.1146; HPLC t_R = 16.2 min.

Kinetic studies of reaction between 1 and α,β-dicarbonyls 5b or 5c

A solution of freshly formed amine **1** was chilled, adjusted to pH 7.0 with 1 M sodium hydroxide solution, diluted with MilliQ water to make 26 mM stock solutions (containing 78 mM of Na_2SO_4) and stored in 1.5 mL aliquots at –20 ºC for later use. This stock was further diluted to the required concentrations with MilliQ water for lower starting concentrations. A chilled (0 °C) 2 mL glass HPLC vial was purged with argon, to which was added 211 μL of aqueous buffer (PBS, TBS: 10 mM Tris, 150 mM NaCl, pH 8.0; MilliQ water, pH 6.8; or 20 mM ammonium acetate, pH 5.4), and chilled solutions of **1** (26 mM, 25 μL, 0.65 μmol) and pyruvaldehyde **5c** (136 mM, 14.4 μL, 1.96 μmol, 3 eq). The total volume was 250 μL and the final concentration of amine **1** was 2.6 mM. To make reaction solutions of other starting concentrations, for example, to achieve 5-A-RU final concentrations of 0.5 mM or 0.1 mM, while maintaining the same molar ratio to pyruvaldehyde, more diluted solutions of 5-A-RU (5 mM or 1 mM) and methylglyoxal (26.1 mM or 5.22 mM) were prepared and used instead. The solution was vortexed for 5s and incubated at the designated temperatures (0, 15 or 37 ºC). At each time point, 5 μL of the reaction mixture was analysed using LCMS. As a single LCMS run took 20 min, several repeats were carried out to collect data for a full curve covering shorter time points. Similarly, the reaction of **1** and glyoxal **5b** was also carried out. Quantification was based on comparing the UV absorbance peak area at 365 nm against that from a solution of lumazine **4b** of known concentration (as determined by standard PULCON NMR). The ratio of regioisomers (**4c** and **4d**) was calculated by comparison of the UV absorbance peak area at 365 nm and was consistent to that determined by NMR.

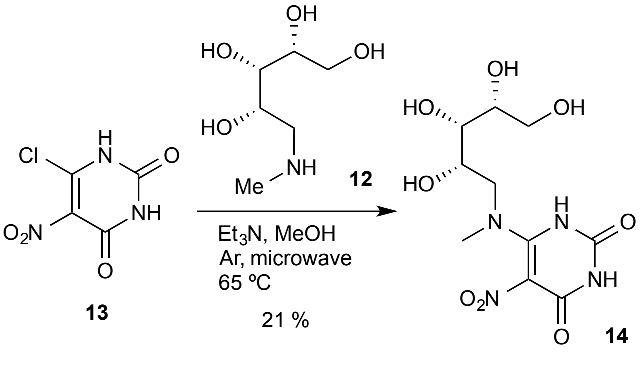

Synthesis of 14

To a solution of 5-chloro-6-nitrouracil[6] (**13**, 561 mg, 2.93 mmol) and *N*-methylribitylamine[7] (**12**, 387 mg, 2.34 mmol) in methanol (5.85 mL) was added triethylamine (0.98 mL, 7.02 mmol) at room temperature. Upon consumption of the *N*-methylribitylamine (**12**) as indicated by mass spectrometry, the solvent was removed, dissolved in 7 % ammonia solution, and loaded onto a DOWEX 1×8 200-400 mesh CI resin, which had been previously washed with methanol, 4 column volumes each of 1 M (pH 6.55) ammonium formate and water. The resin was washed with 4 column volumes each of water, 7 % ammonia solution, water, 0.01 M formic acid, 0.05 M formic acid, and then 0.1 M formic acid, at which point the yellow product eluted as indicated by LCMS. The product fractions were lyophilised to give **14** as a yellow powder (160 mg, 0.500 mmol, 21 %).

^{1}H NMR (600 MHz, DMSO-d_6): δ 2.81 (s, 3H), 3.37 – 3.59 (m, 6H), 4.02 (br d, 1H, J = 8.0 Hz), 4.52 (br s, 1H), 4.9 (br s, 1H), 5.09 (br s, 1H), 6.24 (br s, 1H); ^{13}C NMR (150 MHz, DMSO-d_6): δ 38.8, 55.0, 63.2, 69.1, 72.5, 73.0, 112.1, 148.2, 153.4, 157.3; HRMS (m/z): $[M]^+$ calcd. for $C_{10}H_{17}N_4O_8$, 321.1041; found, 321.1040.

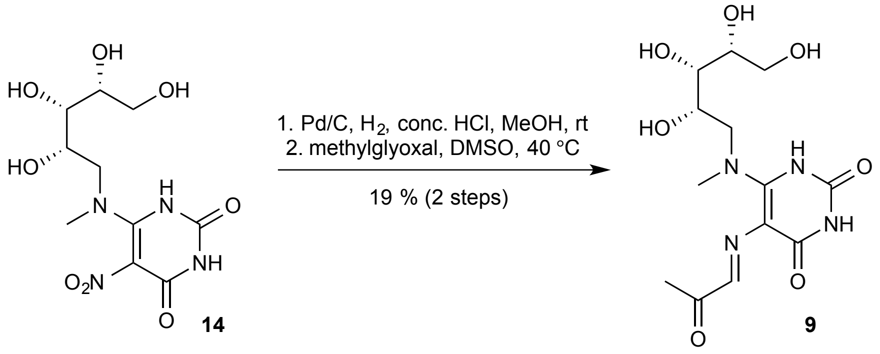

Synthesis of 9

A mixture of **14** (formate salt, 45mg, 0.123 mmol), concentrated hydrochloric acid (32 %, 0.075 mL) and palladium on charcoal (10 %, 18 mg) in methanol (1.5 mL) was stirred at room temperature for 18 hours under hydrogen (1 atm). When starting material was consumed as indicated by mass spectrometry, the reaction was filtered through cotton wool, eluting with degassed methanol into a flask at 0 °C under argon. After removing the solvent *in vacuo*, degassed water (3 mL) was added under argon. Sodium hydroxide solution (1 M) was added to pH 7, and then the mixture was lyophilised. DMSO-d_6 (1 g) was added, filtered and then transferred to an NMR tube, all under an argon atmosphere. After measuring the concentration of the solution by NMR, methylglyoxal (40 % w/w, 2 eq.) was added, mixed, and allowed to stand at room temperature for 1 hr to give **9** (7.9 mg, 0.023 mmol, 19 % over 2 steps) as a 38.6 mM solution in 0.60 mL DMSO-d_6.

^{1}H NMR (600 MHz, DMSO-d_6): δ 2.23 (s, 3H), 3.34 (s, 3H), 3.37 – 3.70 (m, 6H), 4.04, (br d, J = 8.7 Hz, 1H), 8.85 (s, 1H); ^{13}C NMR (150 MHz, DMSO-d_6): δ 24.4, 42.3, 56.4, 63.3, 69.5, 72.8, 73.1, 101.4, 144.8, 148.9, 155.4, 160.8, 200.7; HRMS (m/z): $[M]^+$ calcd. for $C_{13}H_{20}N_4NaO_7$, 367.1224; found, 367.1227.

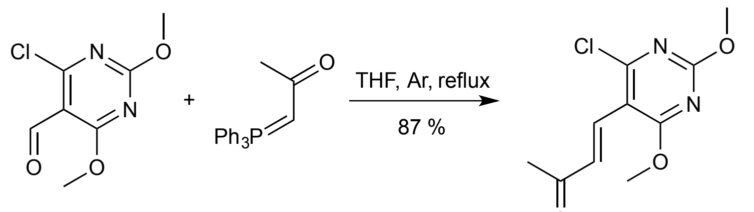

Synthesis of 17

To a mixture of aldehyde[8] **16** (400 mg, 1.97 mmol) and ylide[9] **17** (817 mg, 2.57 mmol) under argon was added tetrahydrofuran (anhydrous, 10 mL) at room temperature. The reaction was refluxed for 1.5 hours and then stirred at room temperature for 16 hours. The solvent was removed *in vacuo* and the residue was purified chromatographically (silica gel, 10 to 40 % ethyl acetate/petroleum spirit) to give **18** (415 mg, 1.71 mmol, 87 %). ^{1}H NMR (600 MHz, $CDCl_3$): δ 2.38 (s, 3H), 4.05 (s, 3H), 4.12 (s, 3H), 7.05 (d, J = 16.3 Hz, 1H), 7.68 (d, J = 16.5 Hz, 1H); ^{13}C NMR (150 MHz, $CDCl_3$): δ 27.5, 55.2, 55.6, 107.8, 131.2, 132.6, 162.5, 163.1, 170.0, 198.8; HRMS (m/z): $[M]^+$ calcd. for $C_{10}H_{12}ClN_2O_3$, 243.0531; found, 243.0535.

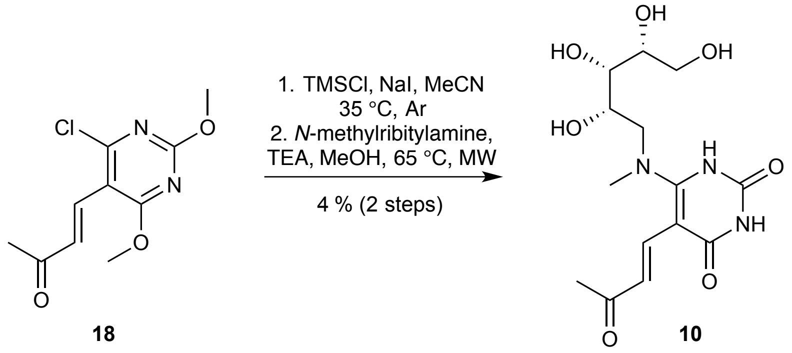

Synthesis of 10

After drying under vacuum, to a mixture of **18** (203 mg, 0.837 mmol), sodium iodide (424, 2.83 mmol) under argon was added acetonitrile (anhydrous, 2 mL) and trimethylsilyl chloride (anhydrous, 319 mg, 2.51 mmol) successively at room temperature. After heating at 35 °C for 3 hrs, LCMS indicated that 10 % of the starting material remained. Trimethylsilyl chloride (anhydrous, 107 mg, 0.84 mmol) and sodium iodide (125 mg, 0.84 mmol) were added. After heating 35 °C for 1 hr, the reaction was complete. After adding water (4 mL) and acetonitrile (2 mL) successively, the mixture was purified by HPLC with a gradient of 100 % solvent A to 7 % solvent B in solvent A

over 10 minutes, then isocratic 100 % B. For only this deprotection reaction, solvents A and B are respectively defined as 0.1 % trifluoroacetic acid in water and 10 % v/v water in acetonitrile with 0.1 % trifluoroacetic acid. After collecting the product peaks, the acetonitrile was removed by rotary evaporation, before lyophilising to give a brown powder consisting of a mixture of the chloro- and iodouracil (100 mg).

A solution of this halouracil mixture (62 mg), amine **12** (79 mg, 0.48 mmol), triethylamine (0.399 g, 3.94 mmol) in methanol (1.7 mL) was heated in a microwave reactor at 65 °C for 1 hour. A further aliquot of triethylamine (181 mg, 1.97 mmol) was added, and the reaction heated in the microwave reactor at 65 °C until the product and the halouracil peak was approximately 1:1 based on UV absorption at 230 nm. The reaction mixture was concentrated *in vacuo*, and then purified on HPLC (0 to 100 solvent B over 120 minutes), collecting the peak at 16-17 minutes. The pH was adjusted to 8.8 with 2 % ammonia solution then lyophilised. After dissolving in water, this process was repeated again to give the product **10** as a brown powder (7 mg, 0.020 mmol, 4 % over 2 steps).

^{1}H NMR (600 MHz, DMSO-d_6): δ 2.15 (s, 3H), 3.04 (s, 3H), 3.37 - 3.60 (m, 5H), 3.98 (m, 1H), 6.82 (d, J = 15.6 Hz, 1H), 7.38 (d, J = 15.6 Hz, 1H); ^{13}C NMR (150 MHz, DMSO-d_6): δ 27.2, 41.0, 55.2, 63.3, 68.6, 72.6, 73.3, 88.1, 120.2, 138.5, 150.8, 163.9, 170.1, 197.7; HRMS (m/z): $[M]^+$ calcd. for $C_{14}H_{21}N_3NaO_7^+$ $[M + Na]^+$: 366.1272; found, 366.1268.

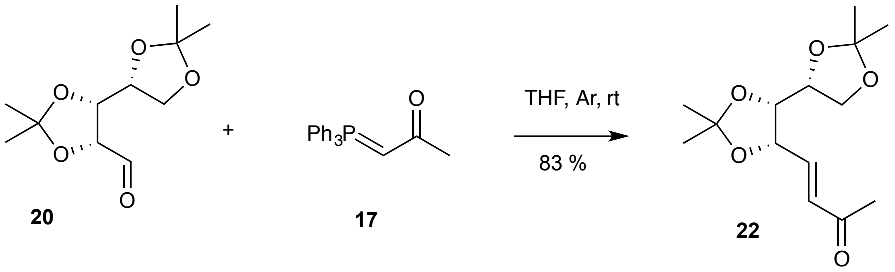

Synthesis of 22

A mixture of aldehyde **20**[10] (952 mg, 4.14 mmol) and ylide **17** (prepared from bromoacetone[9]) (1.95 g, 6.12 mmol) was briefly dried at room temperature under high vacuum, before filling the reaction vessel with argon. Tetrahydrofuran (anhydrous, 20 mL) was added, and the resulting solution was stirred at 45 °C for 17 hours, at which time the reaction was complete, as indicated by NMR. After removing the solvent *in vacuo*, the residue was purified by chromatography (silica gel, 2 – 20 % ethyl acetate/petroleum spirit) to give **22** as a colourless oil (930 mg, 83 %, ~10:1 *trans*/*cis*). NMR spectral data of the *trans* isomer below:

^{1}H NMR (600 MHz, $CDCl_3$): δ 1.30 (s, 3H), 1.39 (s, 3H), 1.41 (s, 3H), 1.48 (s, 3H), 2.30 (s, 3H),

3.89 – 3.95 (m, 2H), 4.08 (d, J = 7.6 Hz, 1H), 4.15 (d, J = 7.6 Hz, 1H), 4.88 (m, 1H), 6.37 (d, J = 16.1 Hz, 1H), 6.89 (dd, J = 16.1, 4.3 Hz, 1H); ^{13}C NMR (150 MHz, $CDCl_3$): δ 25.2, 25.3, 26.8, 27.0, 27.5, 67.7, 73.9, 76.6, 79.0, 109.67, 109.74, 131.0, 142.1, 198.0; HRMS (m/z): $[M]^+$ calcd. for $C_{14}H_{22}NaO_5$, 293.1359; found, 293.1361.

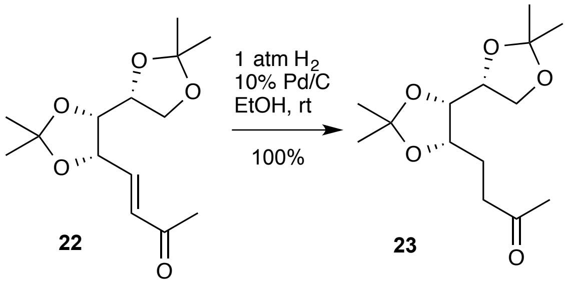

Synthesis of 24

A filtered solution of **22** (930 mg, 3.44 mmol) in ethanol (0.015 M) was hydrogenated with the H-cube Pro flow reactor (5% Pd/C 70 mm catalyst cartridge) with a flow rate of 2 mL/min, under 1 atm of hydrogen at room temperature. After a single pass, NMR indicated complete conversion to the desired product. The product solution was evaporated *in vacuo* to give **23** as a colourless oil (935 mg, quant.).

^{1}H NMR (600 MHz, $CDCl_3$): δ 1.31 (s, 3H), 1.34 (s, 3H), 1.38 (s, 3H), 1.40 (s, 3H), 1.81 (m, 1H), 2.01 (m, 1H), 2.17 (s, 3H), 2.62 (m, 2H), 3.90 – 3.97 (m, 2H), 4.08 – 4.11 (m, 2H), 4.12 – 4.16 (m, 1H); ^{13}C NMR (150 MHz, $CDCl_3$): δ 23.7, 25.4, 25.5, 26.7, 28.1, 30.0, 40.3, 67.9, 73.3, 76.9, 78.6, 108.2, 109.6, 208.4; HRMS (m/z): $[M]^+$ calcd. for $C_{14}H_{24}NaO_5$, 295.1516; found, 295.1519.

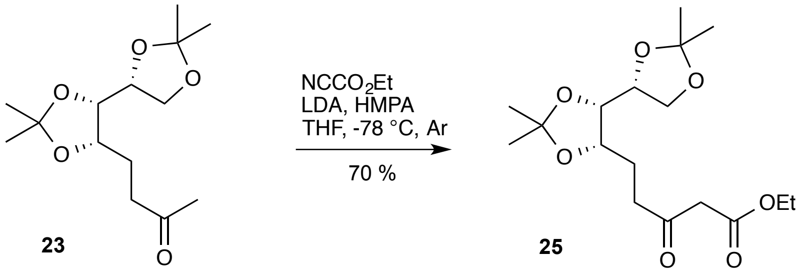

Synthesis of 25

Adapting the procedure by Mander[11], ketone **23** (946 mg, 3.48 mmol) was dried *in vacuo* at 40 °C to constant mass, and then dissolved in tetrahydrofuran (anhydrous, 3.5 mL). This solution was added dropwise to a solution of freshly titrated lithium diisopropylamide (1.71 M, 2.44 mL, 4.17 mmol) in tetrahydrofuran (anhydrous, 8.7 mL) under argon at -78 °C. After stirring at the same temperature for 15 mins, the mixture was stirred in a 0 °C bath for 1 hour. After cooling again to -78 °C, hexamethylphosphoramide (distilled from calcium hydride, 623 mg, 3.48 mmol) and ethyl

cyanoformate (413 mg, 4.17 mmol) were successively added dropwise. The reaction was warmed to -10 °C over 3 hours, then quenched with water. The mixture was extracted with diethyl ether (×3), and then the combined extracts were washed with brine, dried over magnesium sulphate and concentrated *in vacuo*. After chromatography (silica gel, 5 to 10 % ethyl acetate/petroleum spirit, **23** and **25** overlap significantly on TLC), **25** was obtained as a colourless oil (728 mg, 2.11 mmol, 61 % yield), of which ~5% exists as the enol form.

^{1}H NMR (600 MHz, $CDCl_3$): δ 1.29 (t, *J* = 7.3 Hz, 3H), 1.31 (s, 3H), 1.33 (s, 3H), 1.38 (s, 3H), 1.39 (s, 3H), 1.81 – 1.88 (m, 1H), 2.02 – 2.08 (m, 1H), 2.66 – 2.71 (m, 1H), 2.75 – 2.80 (m, 1H), 3.47 (s, 2H), 3.89 – 3.96 (m, 2H), 4.07 – 4.17 (m, 3H), 4.20 (t, 2H, 7.1 Hz); ^{13}C NMR (150 MHz, $CDCl_3$): δ 14.1, 23.6, 25.4, 25.5, 26.7, 28.1, 39.7, 49.4, 61.3, 67.9, 73.3, 76.7, 78.6, 108.3, 109.6, 167.1, 202.4; HRMS (m/z): $[M]^+$ calcd. for $C_{17}H_{28}NaO_7$, 367.1727; found, 367.1728.

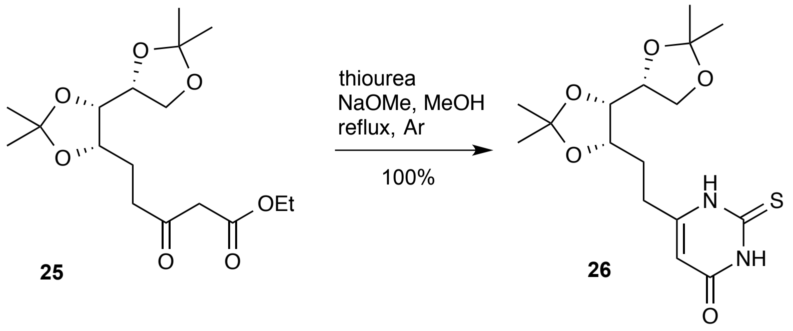

Synthesis of 26

Adapting a literature procedure[12], to thiourea (149 mg, 1.96 mmol) was added a solution of freshly prepared sodium methoxide in anhydrous methanol (2.1 M, 1.39 mL) and a solution of β–keto ester **25** (478 mg, 1.39 mmol) in anhydrous methanol (1.9 mL) successively under argon. After refluxing for 15 hours, TLC indicated complete consumption of starting material. After removing the solvent *in vacuo*, the mixture was partitioned between ethyl acetate and saturated ammonium chloride solution then extracted with ethyl acetate (×2). The combined extracts were washed with brine, dried over magnesium sulfate then evaporated *in vacuo* to give **26** as a light-brown oil (510 mg, quant.) that was used without further purification.

^{1}H NMR (600 MHz, $CDCl_3$): δ 1.38 (s, 3H), 1.43 (s, 3H), 1.45 (s, 3H), 1.48 (s, 3H), 1.96 (m, 1H), 2.13 (m, 1H), 2.58 (m, 2H), 3.99 (m, 1H), 4.02 (m, 1H), 4.10 – 4.16 (m, 2H) 4.24 (m, 1H), 5.77 (s, 1H), 9.87 (br s, 1H), 10.26 (br s, 1H); ^{13}C NMR (150 MHz, $CDCl_3$): δ 25.3, 25.4, 26.8, 27.0, 28.2, 29.8, 67.6, 73.2, 76.6, 78.4, 103.8, 108.9, 110.4, 155.5, 161.4, 175.8; HRMS (m/z): $[M]^+$ calcd. for $C_{16}H_{25}N_2O_5S$, 357.1479; found, 357.1478.

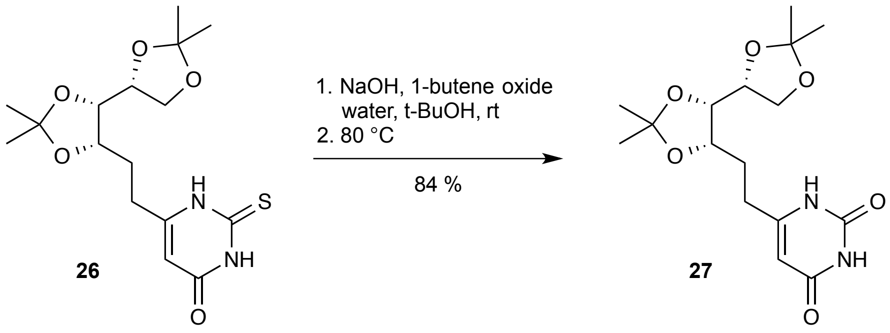

Synthesis of 27

Adapting a literature procedure[13], to a solution of thiouracil **26** (354 mg, 0.99 mmol) in *tert*-butanol and water (1:1, 4.4 mL) was added a solution of sodium hydroxide (1.25 M, 956 uL) and then 1-butene oxide (86 mg, 1.19 mmol). After stirring at room temperature for 20 hours, LCMS indicated complete consumption of starting material. The mixture was heated to 98 °C for 24 hours, at which time LCMS indicated complete conversion to the desired product. The mixture was neutralised with saturated ammonium chloride solution and extracted with ethyl acetate (×3). The combined extracts were washed with brine, dried over magnesium sulfate and concentrated *in vacuo*. The crude product was purified chromatographically (silica gel, 75 % to 100 % ethyl acetate/petroleum spirit) to give an oil that formed a white semi-solid upon standing (282 mg, 0.83 mmol, 84 %).

^{1}H NMR (600 MHz, $CDCl_3$): δ 1.34, (s, 3H), 1.37 (s, 3H), 1.41 (s, 3H), 1.42 (s, 3H), 1.91 – 1.98 (m, 1H), 2.02 – 2.08 (m, 1H), 2.50 – 2.5 (m, 1H), 2.61 – 2.66 (m, 1H), 3.97 (dd, *J* = 8.2, 4.9 Hz, 1H), 4.01 (dd, *J* = 9.0, 5.7 Hz, 1H), 4.10 – 4.16 (m, 2H), 4.22 (m, 1H), 5.58 (s, 1H), 9.97 (br s, 1H), 10.30 (br s, 1H); ^{13}C NMR (150 MHz, $CDCl_3$): δ 25.38, 25.44, 26.7, 27.0, 28.0, 29.7, 67.8, 73.3, 76.4, 78.5, 99.6, 108.6, 110.0, 152.5, 156.0, 164.9; HRMS (m/z): $[M]^+$ calcd. for $C_{16}H_{25}N_2O_6$, 341.1707; found, 341.1708.

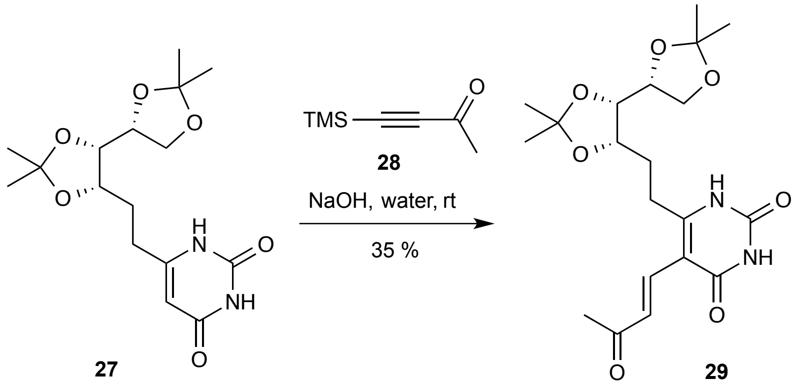

Synthesis of 29

To a solution of **27** (282 mg, 0.83 mmol) in a sodium hydroxide solution (1.25 M, 1.32 mL) was

added ketone **28** (1.16 g, 8.30 mmol). After stirring at room temperature for 22 hours, the mixture was neutralised with saturated ammonium chloride solution and extracted with ethyl acetate (×3). The combined extracts were washed with brine, dried over magnesium sulfate and concentrated *in vacuo*. The crude product was eluted through a silica gel pad with ethyl acetate, purified on reverse phase HPLC (35% to 50 % solvent B over 20 minutes) and then lyophilised to give a white powder (120 mg 0.29 mmol, 35 %).

^{1}H NMR (600 MHz, $CDCl_3$): δ 1.36 (s, 3H), 1.43 (s, 3H), 1.45 (s, 3H), 1.47 (s, 3H), 2.06 (dd, *J* = 14.7, 7.3 Hz, 2H), 2.32 (s, 3H), 2.65 (dt, *J* = 14.5, 7.2 Hz, 1H), 2.96 (dt, *J* = 14.2, 7.7 Hz, 1H) 4.00 – 4.06 (m, 2H), 4.13 – 4.18 (m, 2H), 4.25 (m, 1H), 7.38 (d, *J* = 15.5 Hz, 1H), 7.51 (d, *J* = 15.4 Hz, 1H); ^{13}C NMR (150 MHz, $CDCl_3$): δ 25.3, 25.4, 26.7, 26.9, 27.4, 28.1, 29.9, 67.7, 73.3, 76.0, 78.4, 106.1, 108.8, 110.7, 128.2, 131.1, 149.1, 156.5, 162.1, 198.7; HRMS (m/z): $[M]^+$ calcd. for $C_{20}H_{29}N_2O_7$, 409.1969; found, 409.1966.

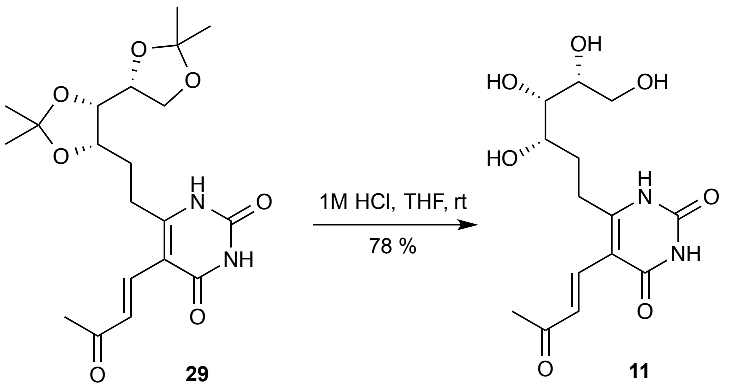

Synthesis of 11

To a solution of **29** (7.5 mg, 0.018 mmol) in tetrahydrofuran (3.75 mL) was added a hydrochloric acid solution (1M, 3.75 mL). After stirring the mixture for 23 hours, LCMS indicated complete conversion to the desired product. Ammonia solution (3.5 % w/w, 2 mL) was added to the mixture, concentrated *in vacuo* to 1 – 2 mL, then purified by reverse phase HPLC (0 % to 50 % B over 30 minutes). The product is unstable without an aqueous environment (e.g. in DMSO or neat). Therefore, the product HPLC fraction was concentrated at 40 °C *in vacuo* to 1 – 2 mL. Water (~ 2 mL) was added, then concentrated at 40 °C *in vacuo*. This was repeated two more times to remove traces of acetonitrile. After concentrating the solution to < 0.5 mL, water and D_2O were added to prepare a 0.6 mL 10% D_2O/H_2O NMR sample containing 4.7 mg of **11** (0.014 mmol, 78 %), as measured by the PULCON technique[14].

^{1}H NMR (600 MHz, 10% D_2O/H_2O): δ 1.75 – 1.81 (m, 1H), 1.92 – 1.98 (m, 1H), 2.35 (s, 3H), 2.79 – 2.91 (m, 2H), 3.57 – 3.61 (m, 2H), 3.66 – 3.69 (m, 1H), 3.74 – 3.77 (m, 2H), 7.21 (d, *J* = 16.0 Hz,

1H), 7.52 (d, *J* = 16.0 Hz, 1H); ^{13}C NMR (150 MHz, 10% D_2O/H_2O): δ 27.0, 27.2, 30.1, 62.9, 70.7, 72.4, 74.3, 105.9, 127.8, 136.4, 151.9, 161.3, 165.0, 205.2; HRMS (m/z): $[M]^+$ calcd. for $C_{14}H_{21}N_2O_7$, 329.1343; found, 329.1348.

Computational methods

DFT Calculations for direct imine inversion activation barrier: All *ab initio* DFT calculations were performed using Gaussian 09 Rev A.02[15] using the B3LYP functional and the 6-311g(2d,2p) basis set. The geometries of *trans*-**3a-d** and *cis*-**3a-d** were optimised and the transition states were revealed using the QST3 method. Energy minima were confirmed by the absence of negative frequencies whereas transitions states had one negative frequency only. The intrinsic reaction coordinate from the transition states to the lower energy (*trans*/*cis*) structures was analysed in both forward and reverse directions to confirm that the transition states were on a path between the corresponding minima. The activation energy delta G was calculated as the difference between the sum of electronic and thermal free energies for the transition states and *trans*-**3a-d**.

DFT Calculations for Ligand Structural Minimisation: The 3D coordinates of **3c** deposited in the PDB (PDB code: 4NQC) were taken as initial structure for DFT minimisation. This structure also represented the bioactive conformation of the ligand. As the hydrogen atoms were not resolved in the crystal structure, the “Ligand Preparation” module from Maestro (version 9.4, Schrödinger, LLC, New York, NY, 2014) was used to model hydrogen atoms onto the ligand. Before quantum chemical calculations, a molecular mechanic minimisation was performed with the UFF force field available in Gaussian 09 software[15]. Further Quantum Mechanical calculations were performed at the level of B3LYP/6-311+g(d,p). Molecular structures of all other ligands were constructed based on the crystal structure of **3c** respectively and QM optimised accordingly. All calculations were performed in a vacuum.

MAIT reporter cell activation assay

Conducted as described previously[16]. Jurkat cells transduced with genes encoding a MAIT TCR comprising the TRAV1-2-TRAJ33 invariant α chain and a TRBV6-1 β chain were co-incubated with C1R antigen presenting cells expressing MR1 (CIR.MR1) in the presence of graded amounts of ligands for 16 h. Cells were subsequently stained with PE-Cy7-conjugated anti-CD3 (eBioscience), and APC-conjugated anti-CD69 (BD Biosciences) antibodies, followed by Streptavidin-PE (BD Biosciences), before analysis by flow cytometry. Activation of Jurkat.MAIT cells was measured by an increase in surface CD69 expression.

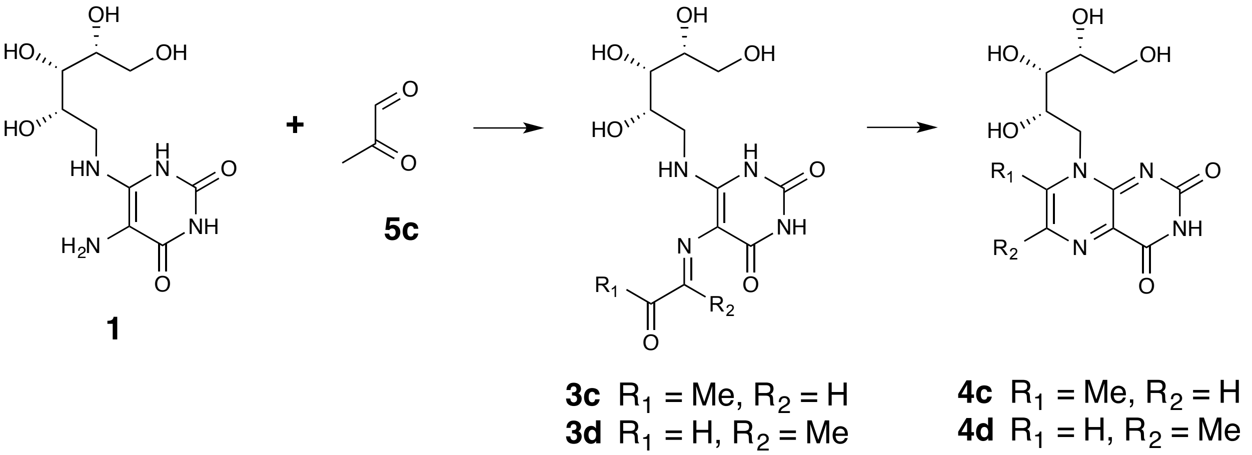

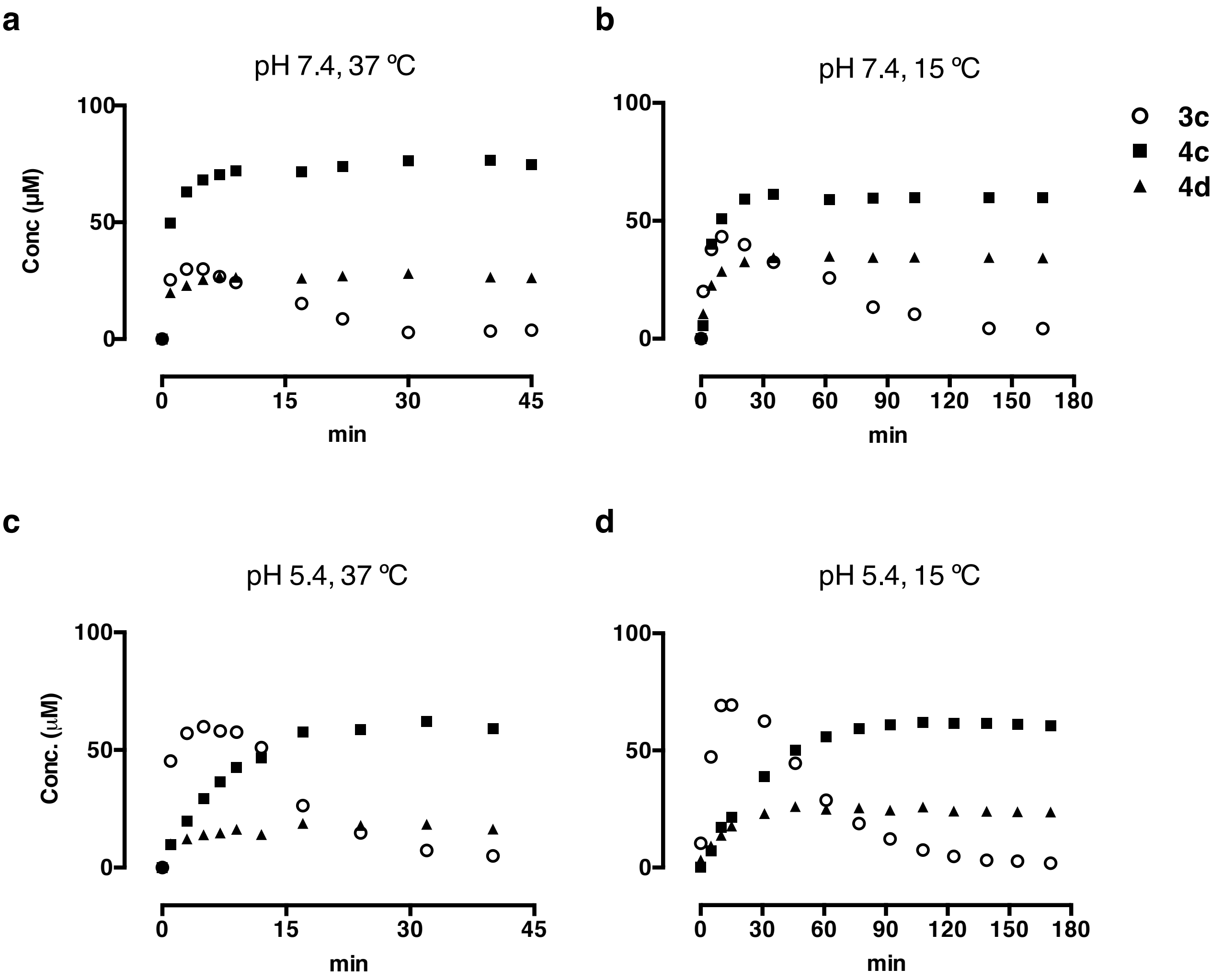

Supplementary Figure 1. Temperature and pH dependence of reactions between 1 and 5c in aqueous buffers. (a-b) PBS buffer (pH 7.4); **(c-d)** NH_4OAc buffer (20 mM and 150 mM of NaCl). The starting concentrations of **1** and **5c** were 2.6 mM and 7.8 mM, respectively. The ratio of cyclised products (i.e. **4c/4d)** was constant in all four cases: 2.8:1 (panel a), 1.8:1 (panel b), and 2.5:1 (panels c and d).

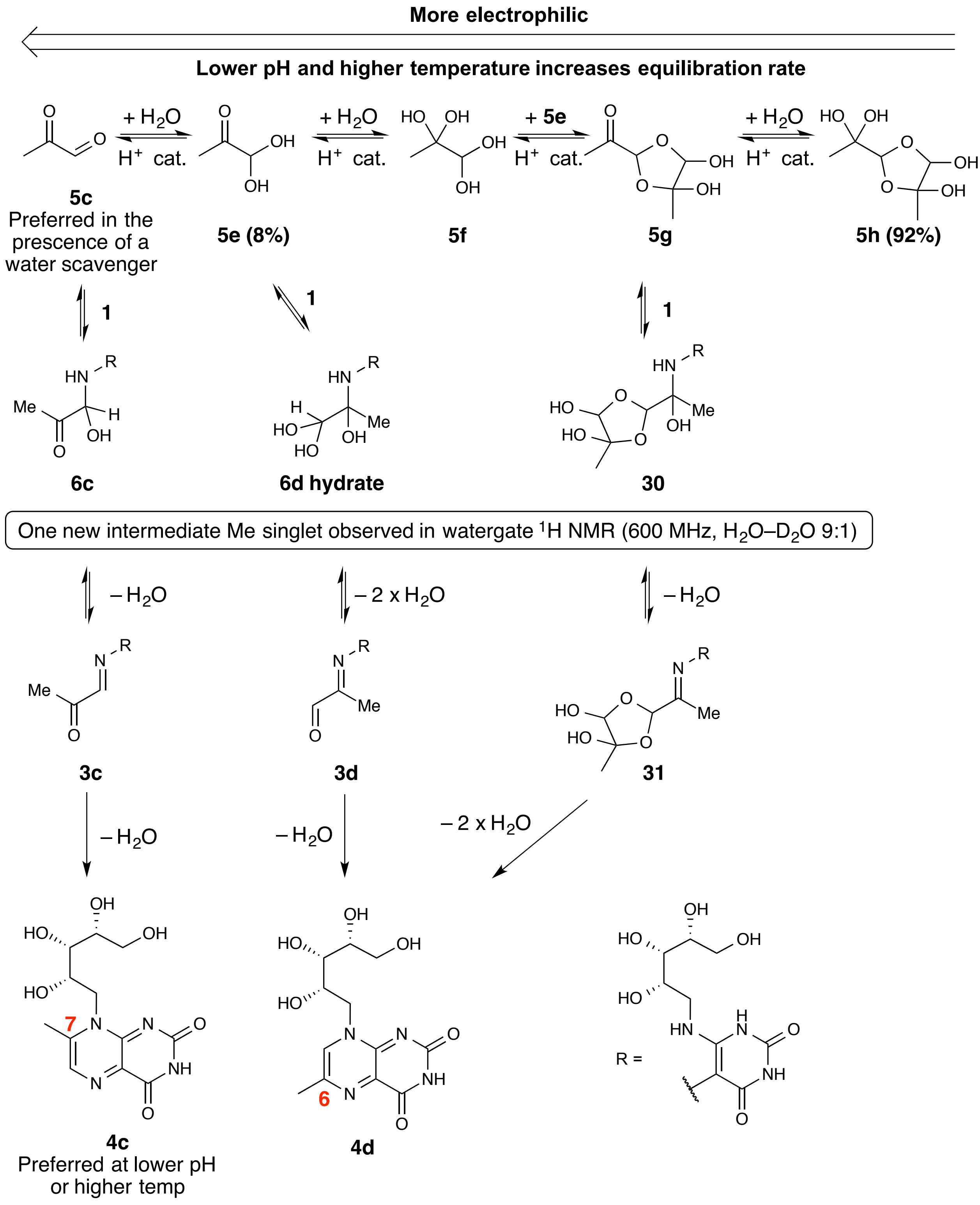

Supplementary Figure 2. Proposed mechanism explaining regioselectivity in the reaction between 1 and 5c. Pyruvaldehyde (**5c**) predominantly exists as the dimeric acetal hydrate form (**5h**, 92%) at high concentrations in water, with only a trace amount of the monomeric hydrate form (**5e**, 8%), as determined by NMR spectroscopy. When the acidity or temperature of the medium is increased, the equilibration between the more electrophilic monomeric and the less electrophilic dimeric (protected) species is faster. If a non-aqueous medium is used, the equilibrium shifts from the dimeric (**5h**, **5g**) to the monomeric forms (**5e**, **5c**), thus leading to the regioselective production **3c** and then **4c**.

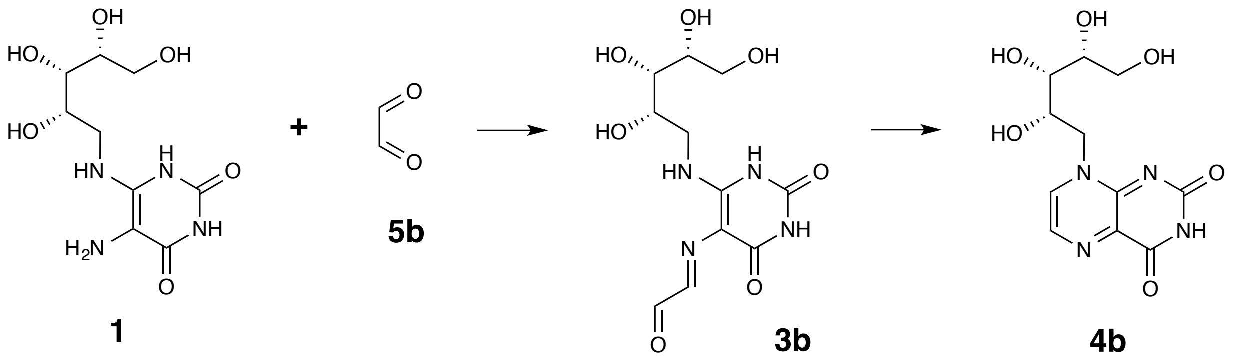

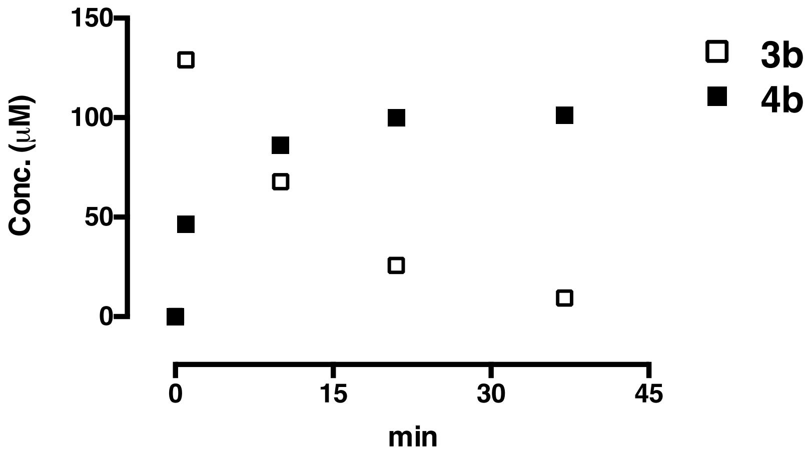

Supplementary Figure 3. Reaction profile between 1 and 5b in PBS buffer at pH 7.4 and 15 ºC. The starting concentrations of **1** and **5b** were 2.6 mM and 7.8 mM, respectively. The maximum concentration of **3b** was already observed at the first practical time point (1 min). The reaction of **1** and **5b** in NH_4OAc buffer (20 mM and 150 mM of NaCl, pH5.4, 15 ºC) was even faster.

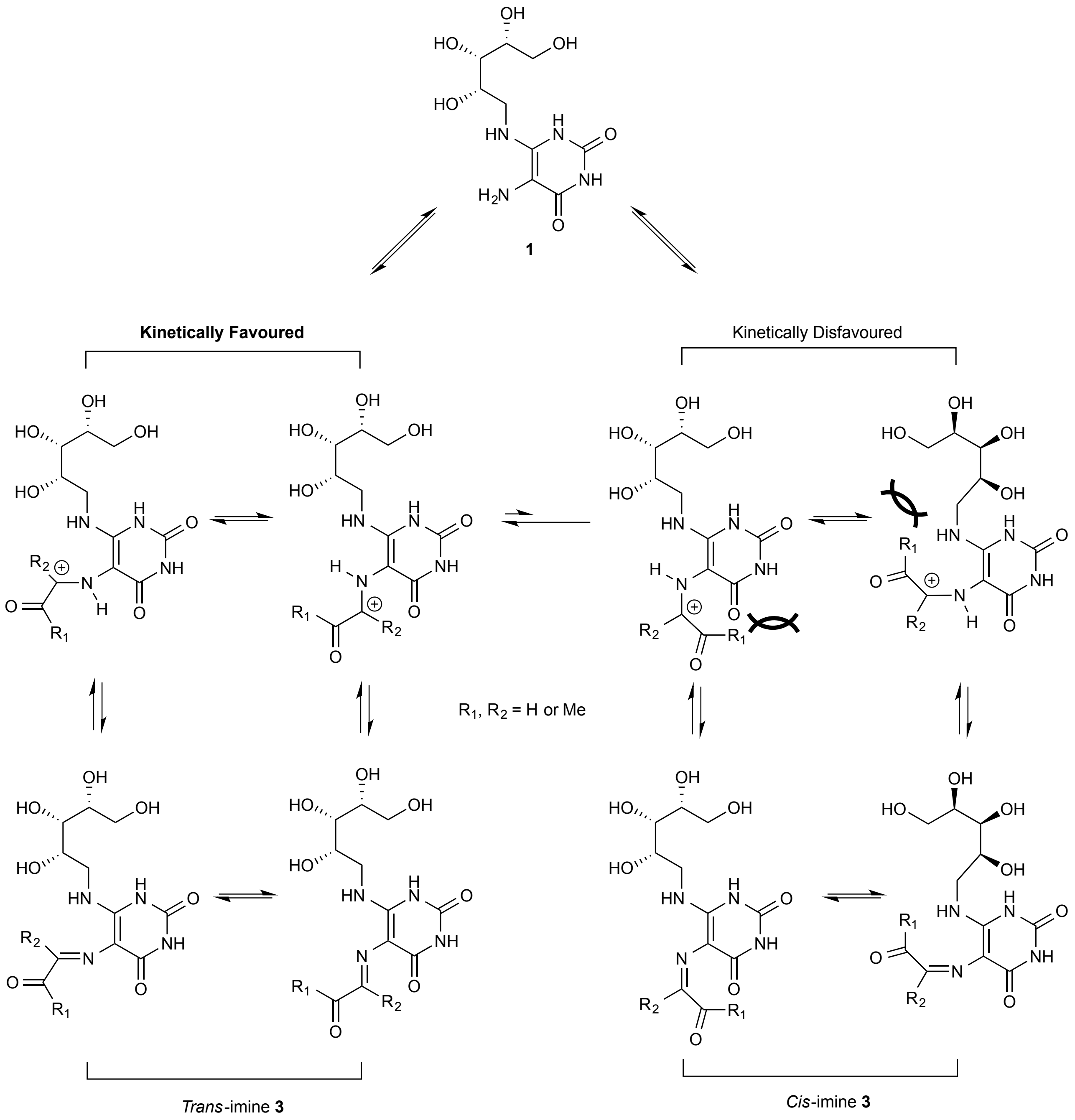

Supplementary Figure 4. *Trans*-imine is selectively formed over *cis*-imine when under kinetic control. In polar solvents (such as DMSO or water) under neutral or slightly acidic conditions, imine formation from hemiaminals typically proceed via a carbocation intermediate. The carbocation conformers that would lead to the *cis*-imine are sterically disfavoured. Thus, the *trans*-imine is formed selectively as its formation proceeds via lower energy carbocation conformers.

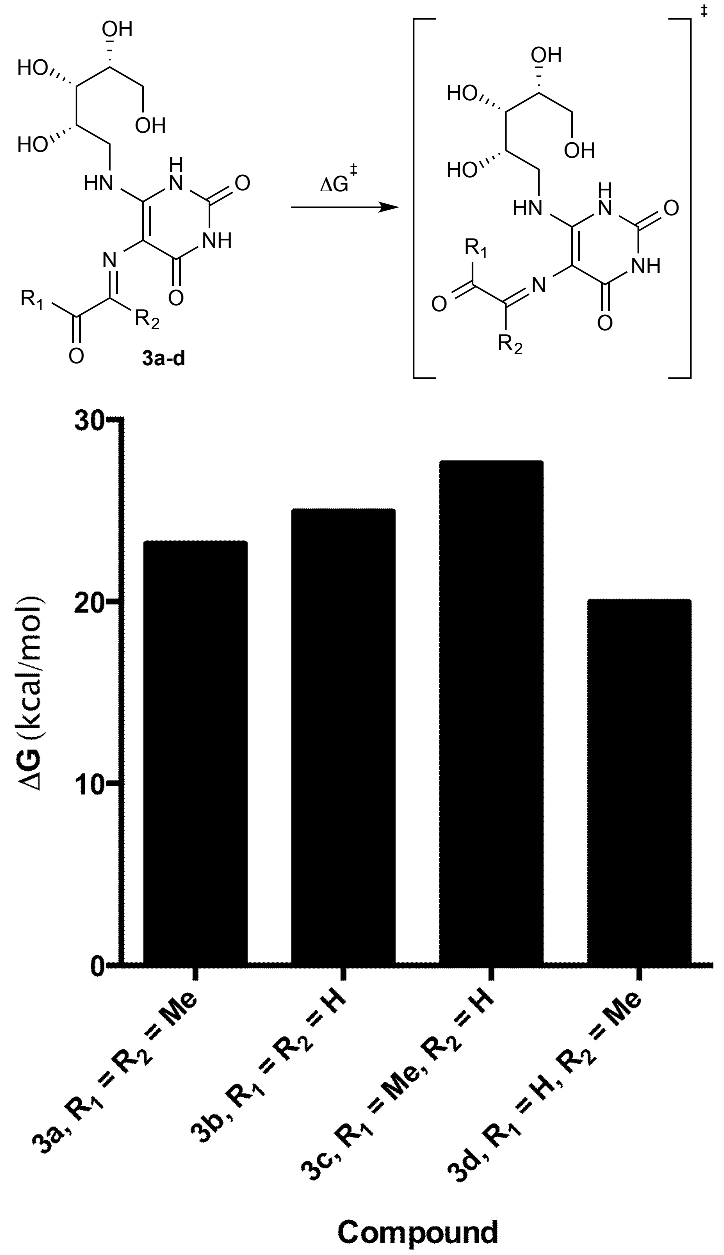

Supplementary Figure 5. Calculated activation energies of *trans*-3a-d *in vacuo* for direct inversion isomerisation. The energy of each conformer was calculated based on *ab initio* DFT method at the B3LYP/6-311++g(2d,2p) level of theory.

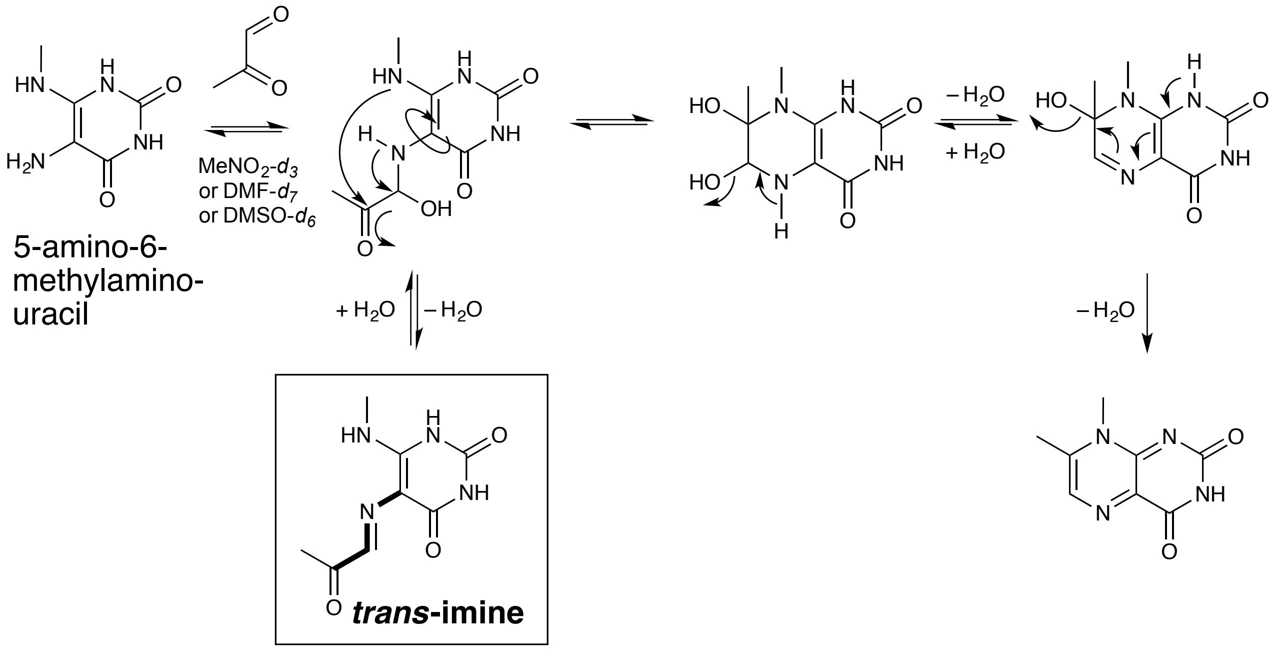

Supplementary Figure 6. Condensation of 3c with 5-amino-6-methylaminouracil in three aprotic polar solvents. Using 5-amino-6-methylaminouracil as a model compound, exclusive imine formation was only observed in DMSO-d_6, the solvent with the largest dielectric constant and the ability to effectively solvate water molecules. These properties respectively accelerate the imine forming, cationic E1 dehydration process over the cyclisation reaction, and suppress imine rehydration through solvation of liberated water molecules.

a

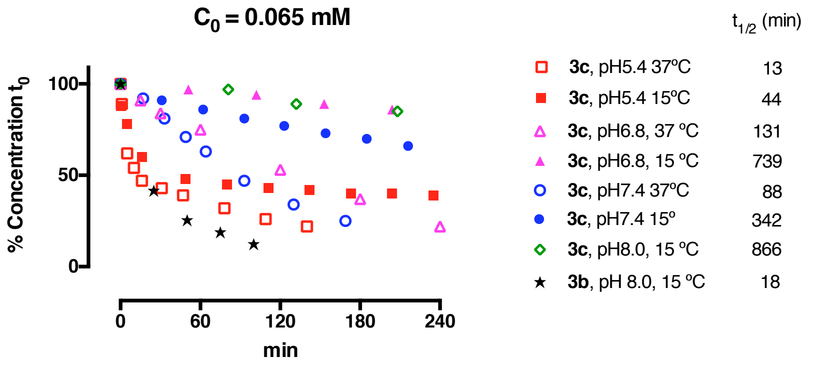

b

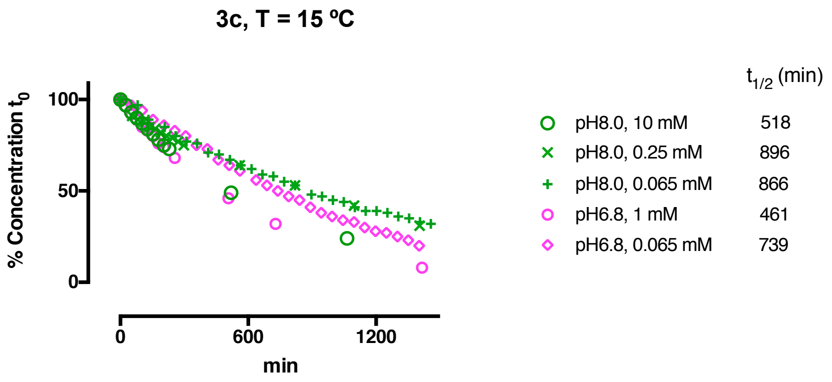

c

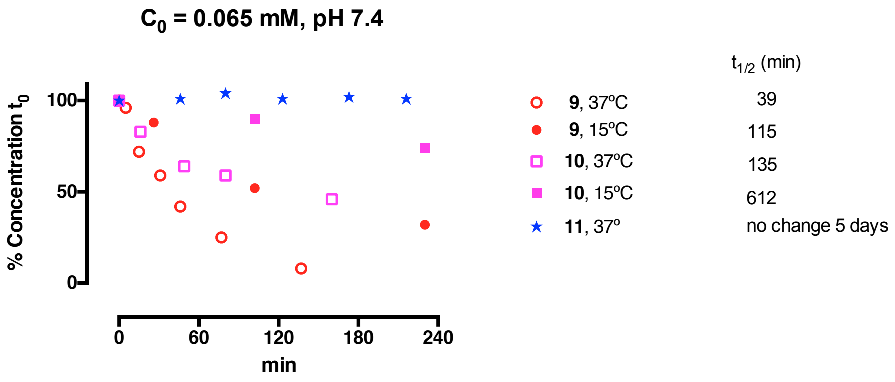

Supplementary Figure 7. Stability in aqueous media. TBS buffer (10 mM Tris, 150 mM NaCl, pH 8.0), PBS (pH 7.4), MilliQ water (pH 6.8), or ammonium acetate buffer (20 mM, 150 mM NaCl, pH 5.4). **(a)** Degradation of **3c** and **3b** at various pH and temperatures with a starting concentration of 0.065 mM; **(b)** Degradation of **3c** at various pH and starting concentrations; **(c)** Degradation of compounds **9-11** at various temperatures at pH 7.4 with a starting concentration of 0.065 mM. The initial concentration was quantified by measuring the LCMS UV chromatogram absorbance peak area at 365 nm. Data shown as % of initial concentration. Imine **3b** was significantly less stable, decaying exponentially with a half-life of 18 minutes (pH 8.0, 15 °C). The half-life of **3a** was unquantifiable, as it had completely degraded to the **4a** in less than 5 minutes under the same conditions. Stability of **3c** at pH 6.8-8.0 was relatively independent of its concentration at the range of 0.065 – 0.25 mM.

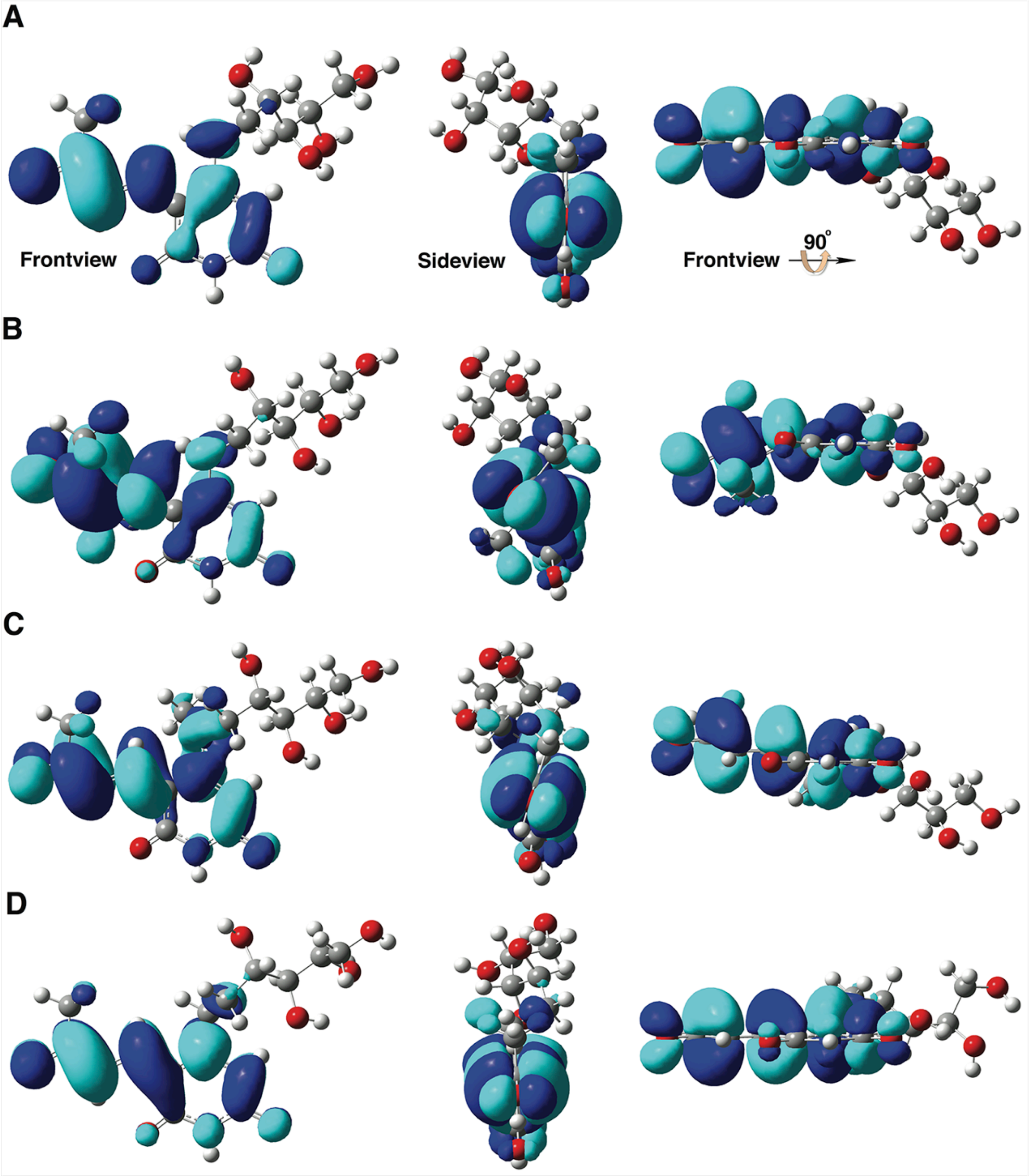

Supplementary Figure 8. LUMO densities of compounds (a) 3c, (b) 3a, (c) 10, and (d) 11. LUMO mainly centered exclusively on non-sugar part of the molecules. Orbital phases are shown as cyan and blue. The molecular orbitals of **11** were very similar to **3c**, with the exception that the LUMO was biased towards the α-carbon atom of enone of **11**. The orbital phasic pattern of **11** was also similar to **3c**, but significantly different to **3a** and **10**. Similar trends were observed with the HOMO. These subtle differences between the ligands may affect their ability to form pi-pi interactions, and their reactivity with Lys43 of MR1 to form a Schiff base.

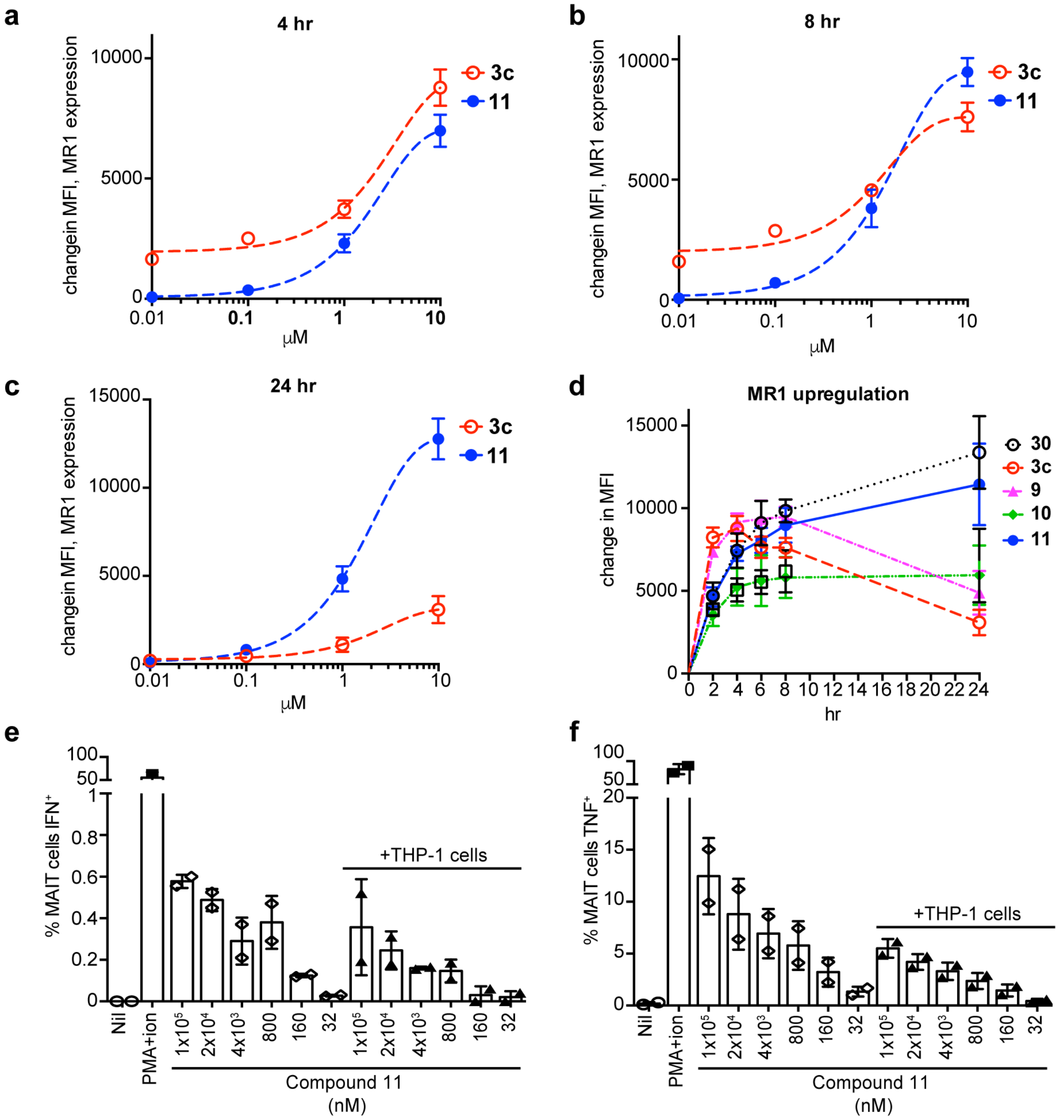

Supplementary Figure 9. Analogue 11 is functionally similar to 5-OP-RU (3c). (a-d) Dose-response of surface MR1 expression up-regulation caused by 5-OP-RU (**3c**) and compound **11** at indicated time points. MR1 was detected on C1R.MR1 cells at indicated time points after addition of indicated doses (**a-c**), or 10 μM 5-OP-RU (**3c**), compounds **9** – **11** or Ac-6-FP (**30**) (**d**) and incubation at 37 °C. Mean +/- SEM from 3 independent experiments. (**e-f**) Cytokine profiles after activation of human PBMCs with **11** at various concentrations with or without THP-1 cells. Mean +/- SD from two donors. Experiment performed twice. See Supplementary Fig. 11 for gating strategy.

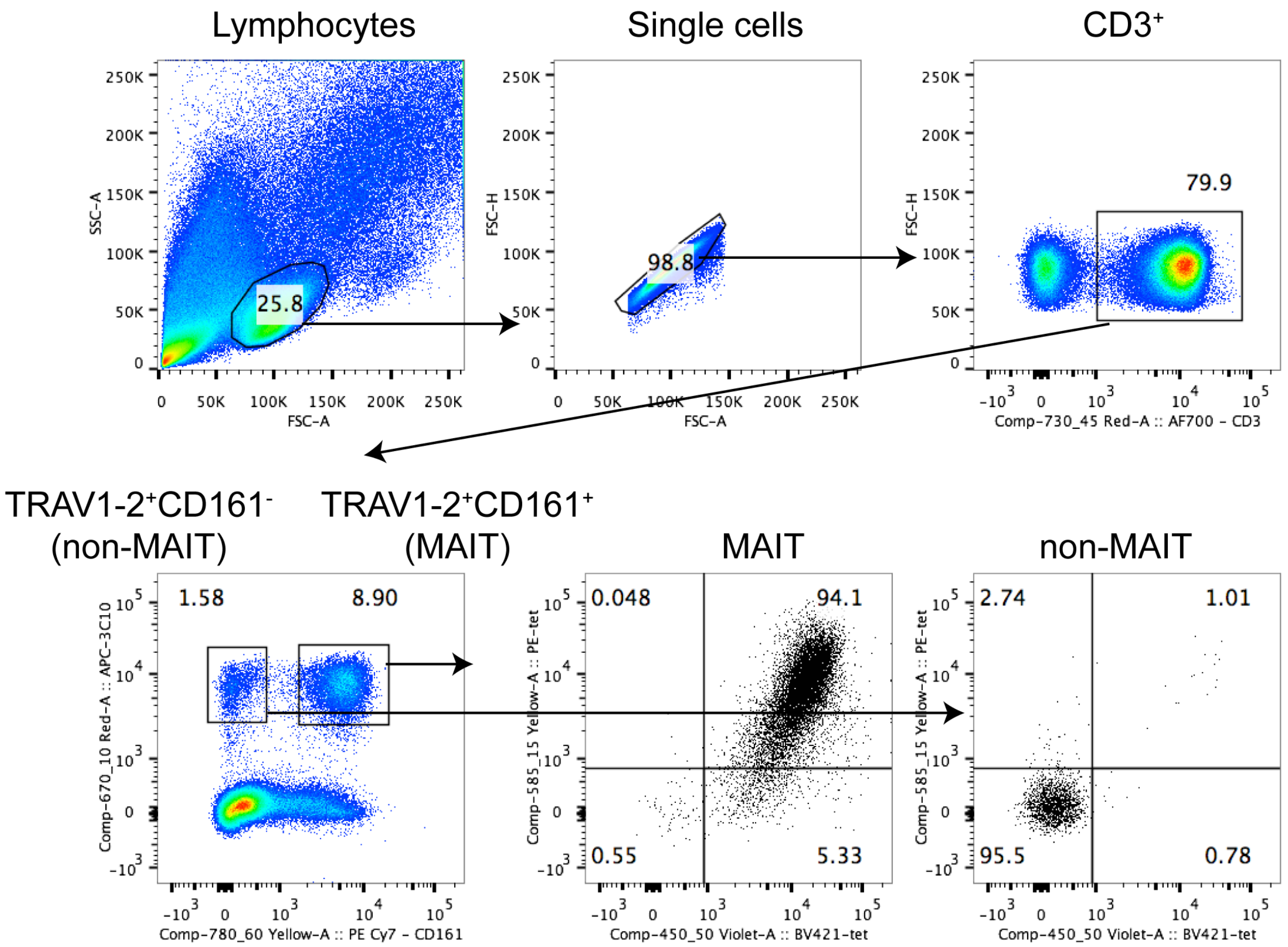

Supplementary Figure 10. Gating strategy for human PBMC experiments shown in Fig.7b.

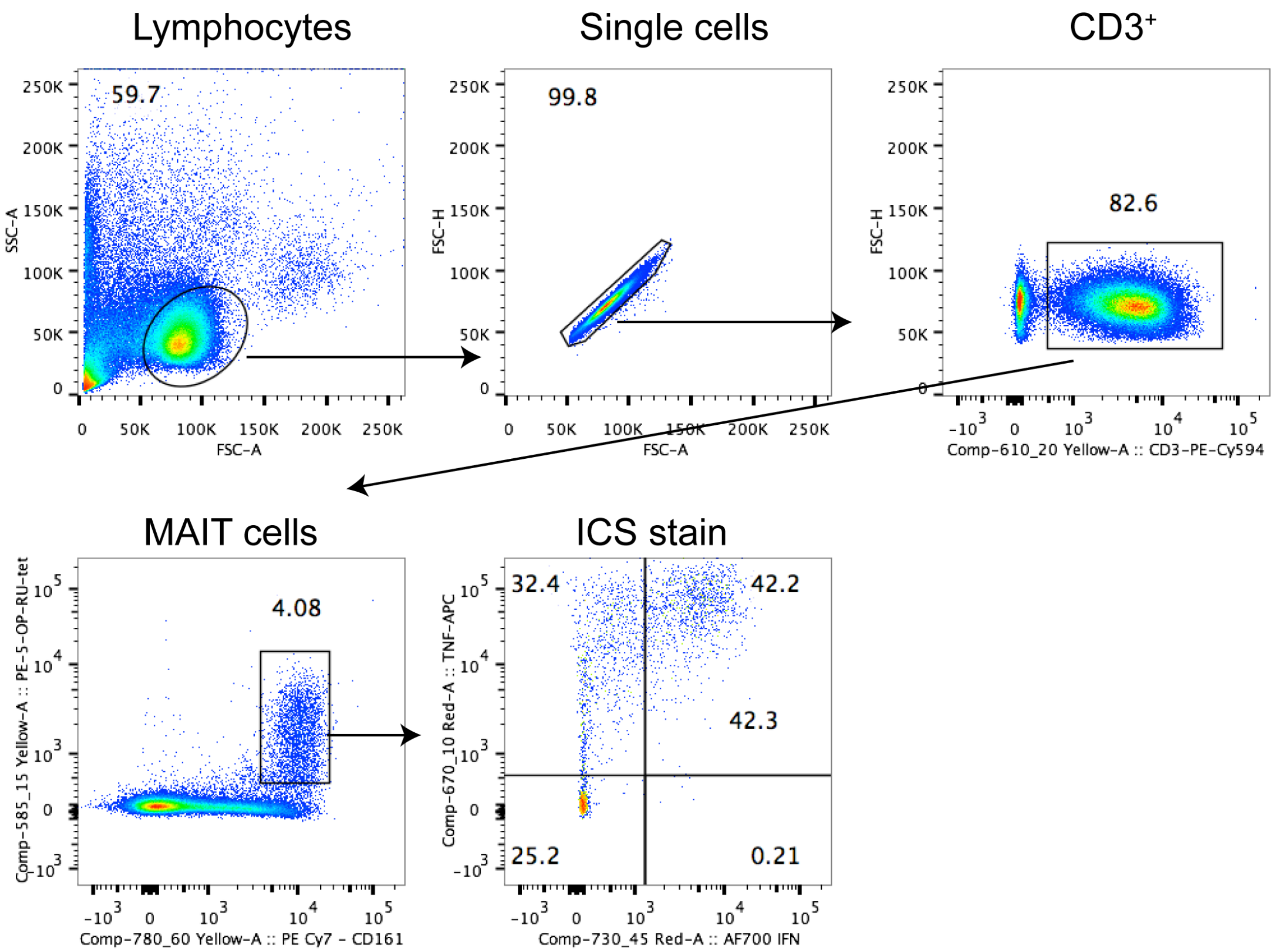

Supplementary Figure 11. Gating strategy for human PBMC stimulation experiments shown in Fig.7c and Supplementary Fig. 9e-f.

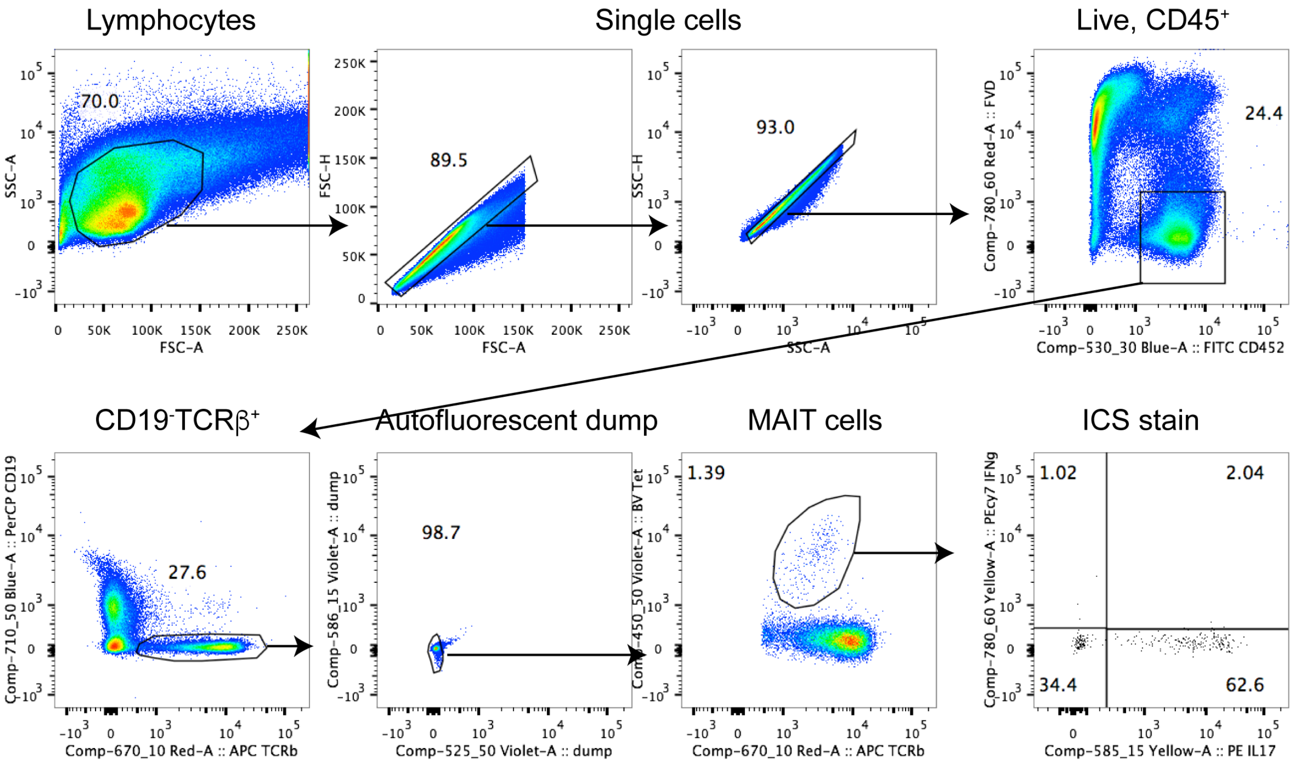

Supplementary Figure 12. Gating strategy for mouse experiments shown in Fig.7d-e.

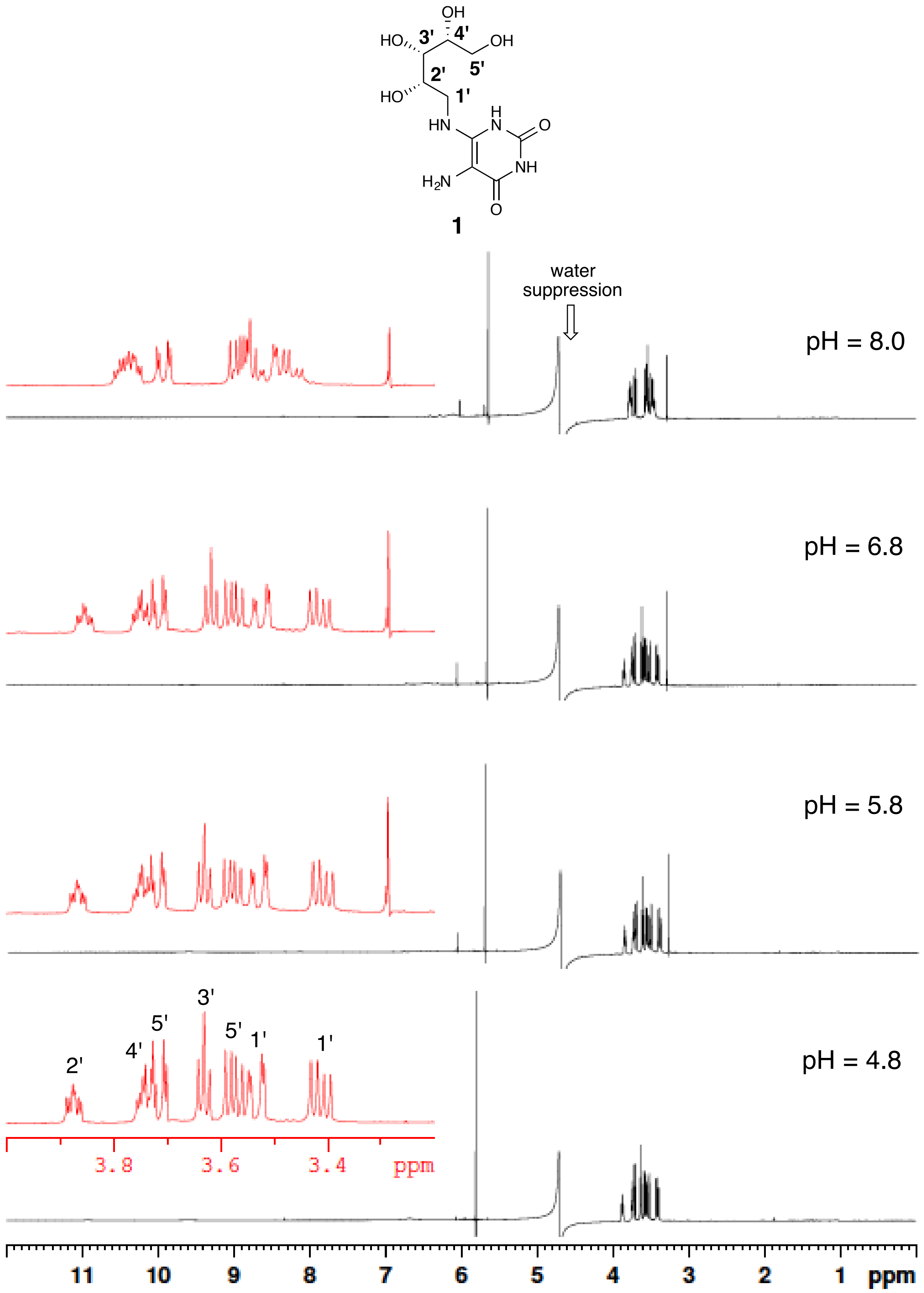

Supplementary Figure 13. Watergate ^{1}H NMR of **1** in 9:1 H_2O–D_2O at various pH.

a

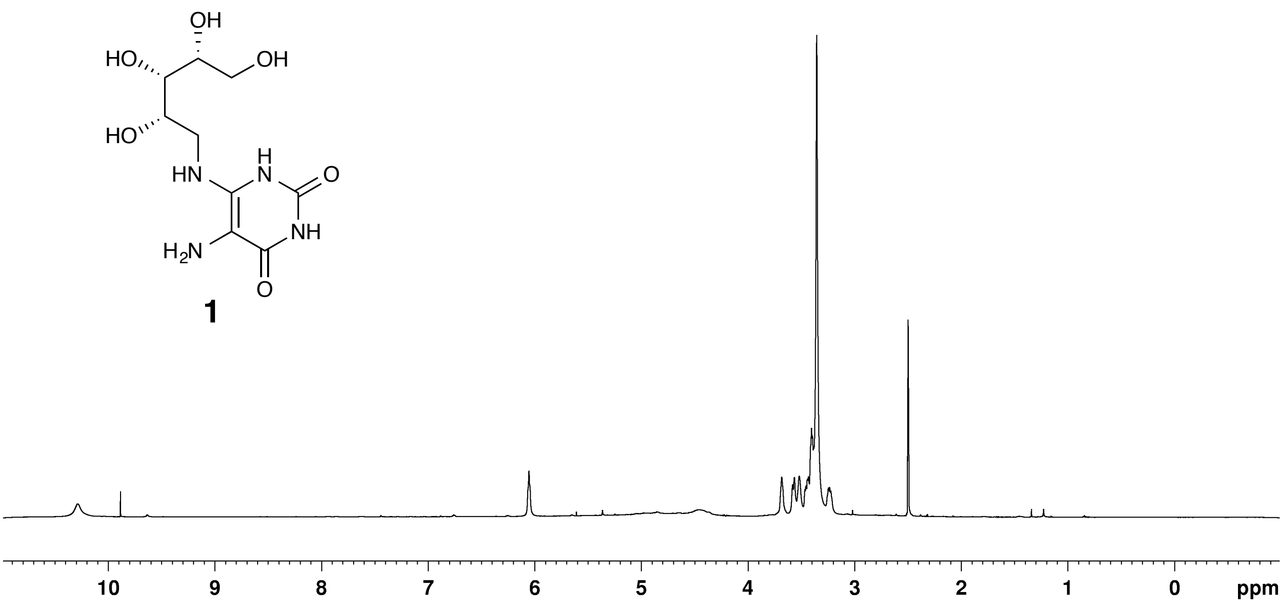

b

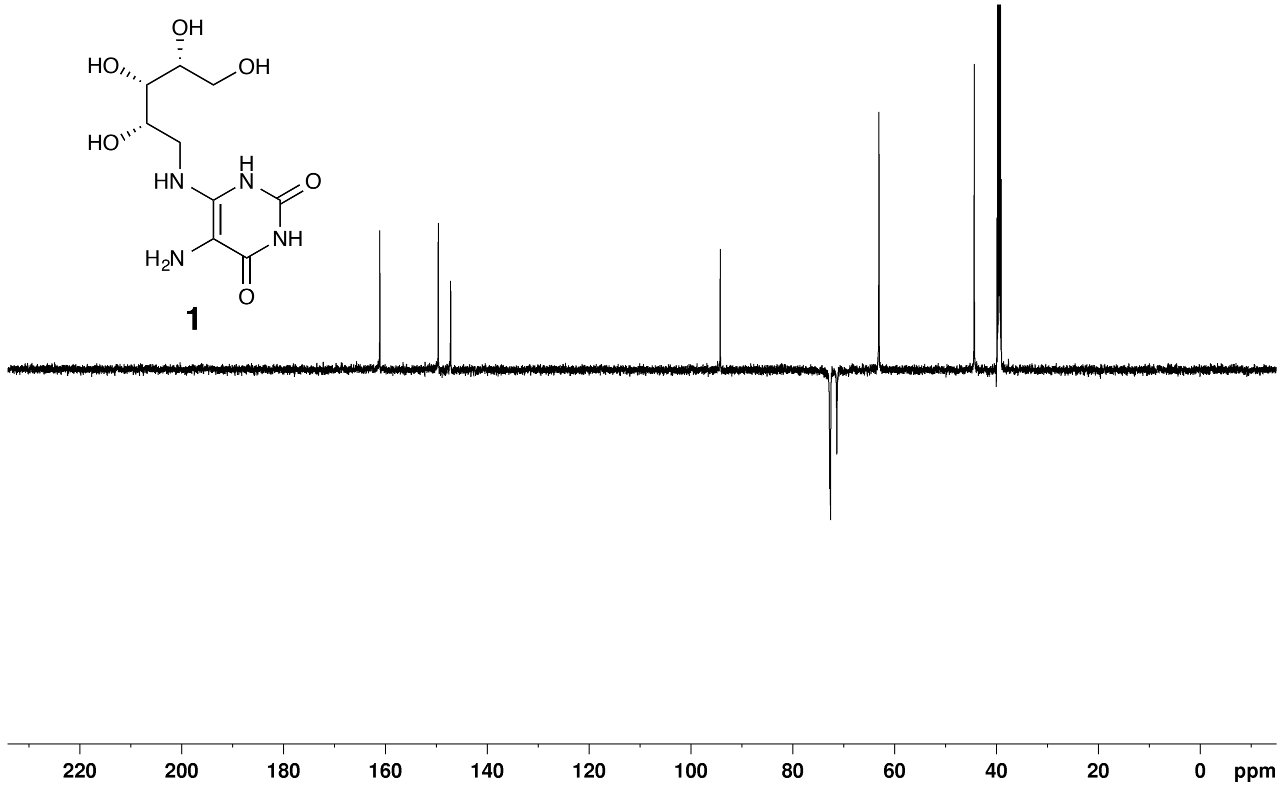

Supplementary Figure 14. (a) ^{1}H and (b) ^{13}C NMR spectrum of 1 in DMSO-d_6.

a

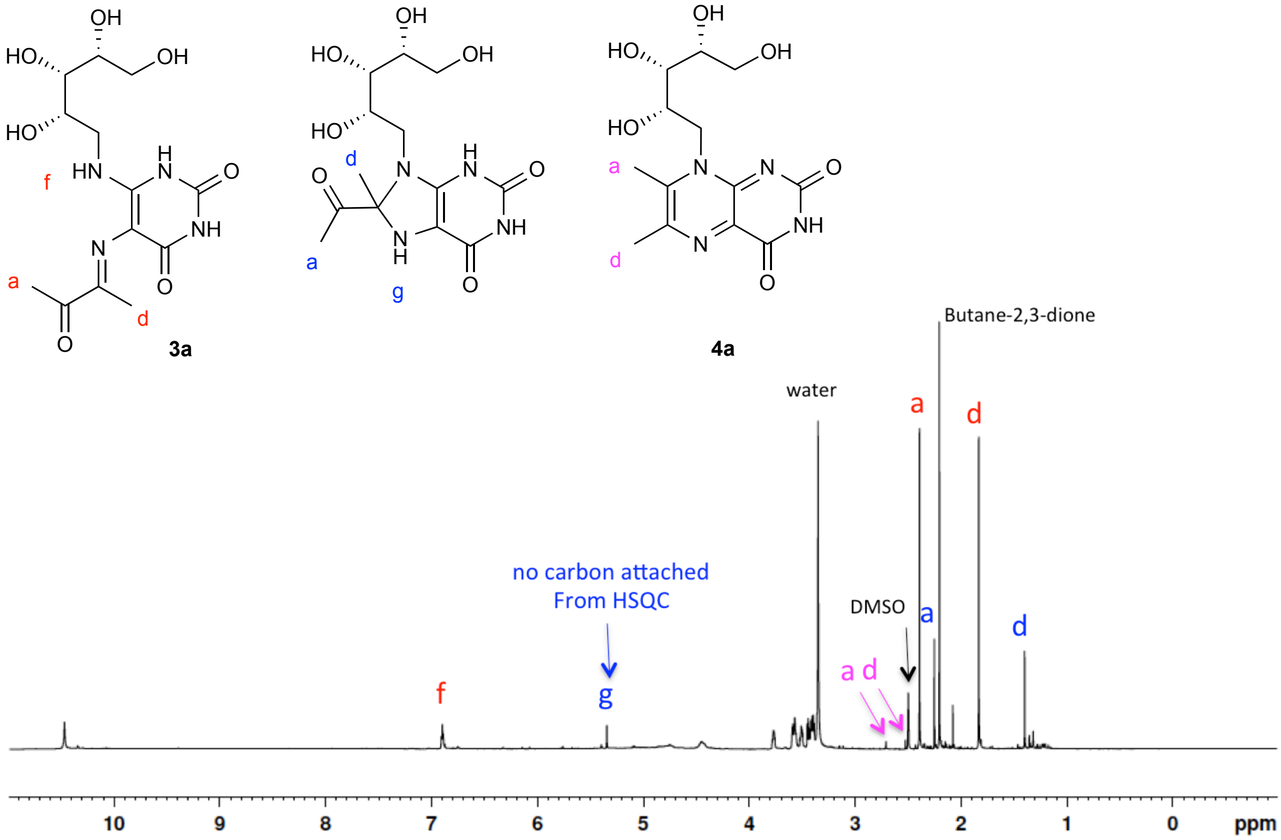

b

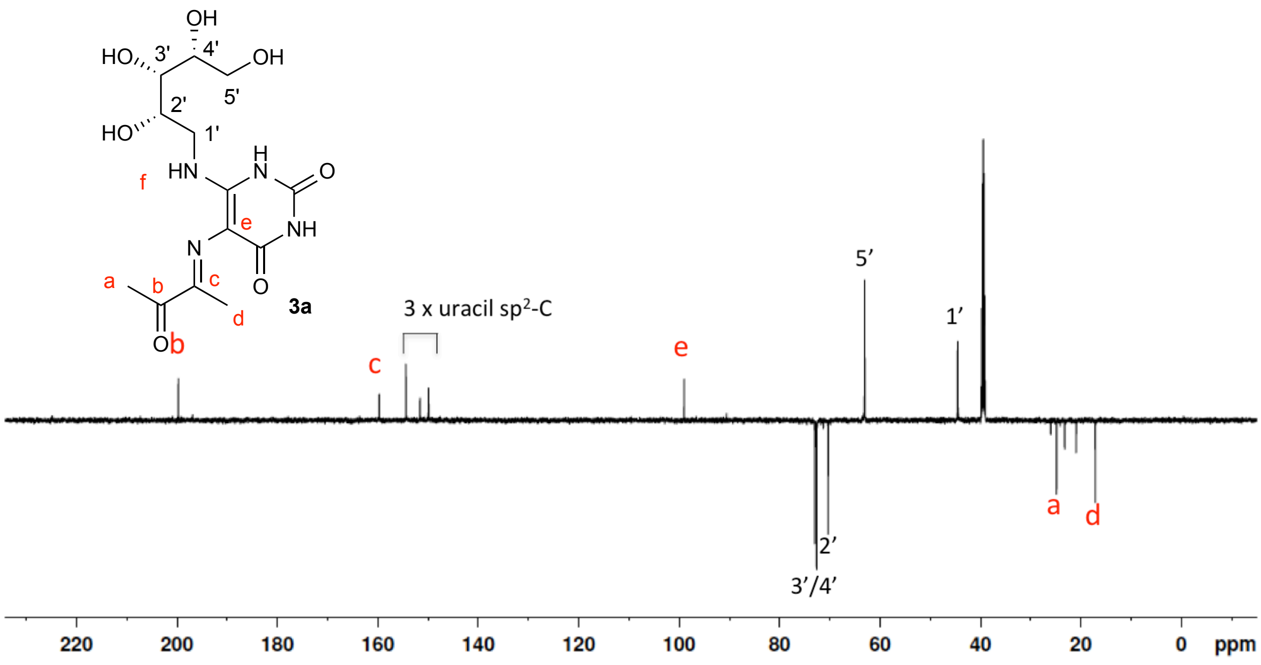

Supplementary Figure 15. (a) ^{1}H and (b) ^{13}C NMR spectrum of 3a in DMSO-d_6.

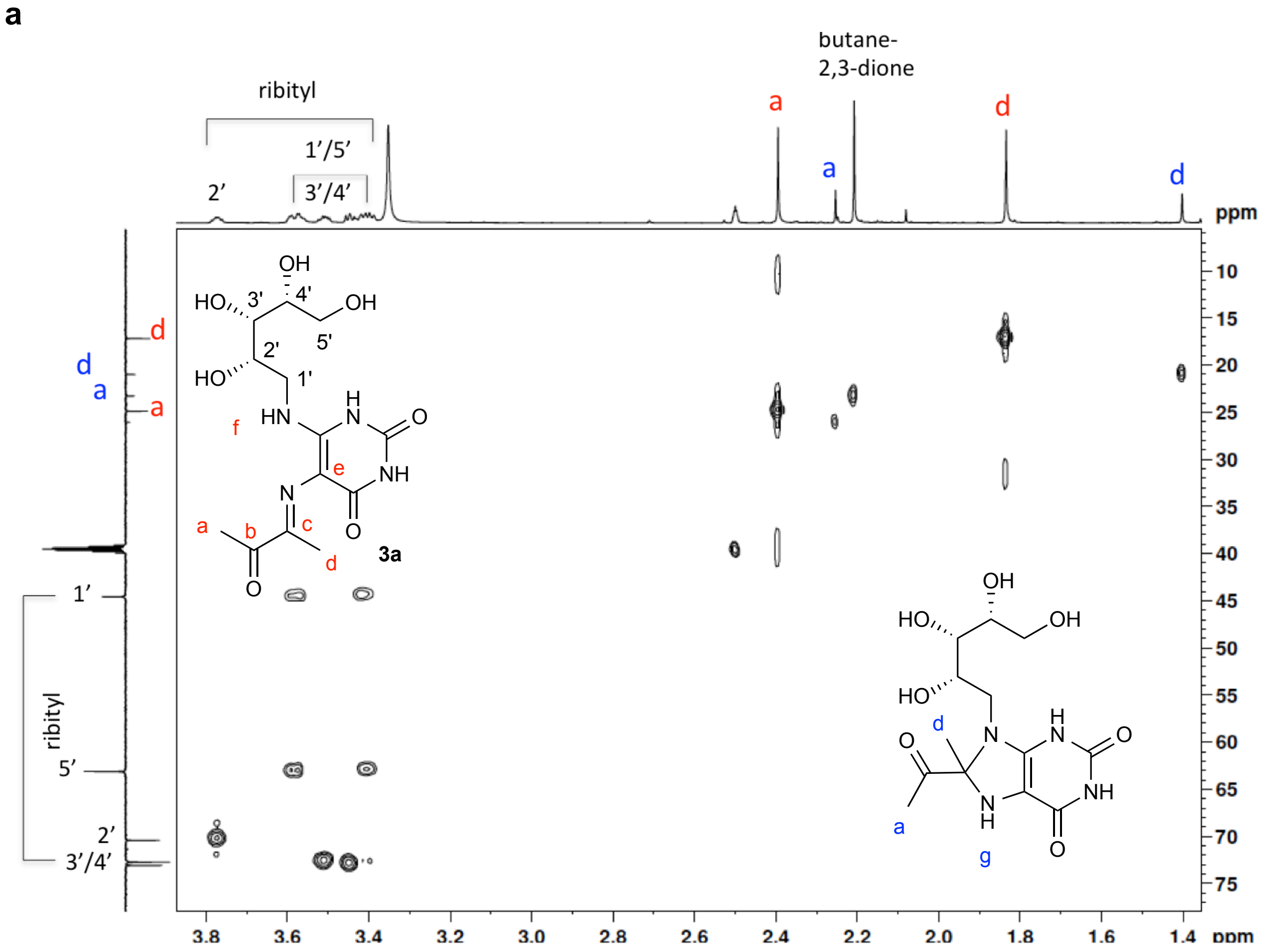

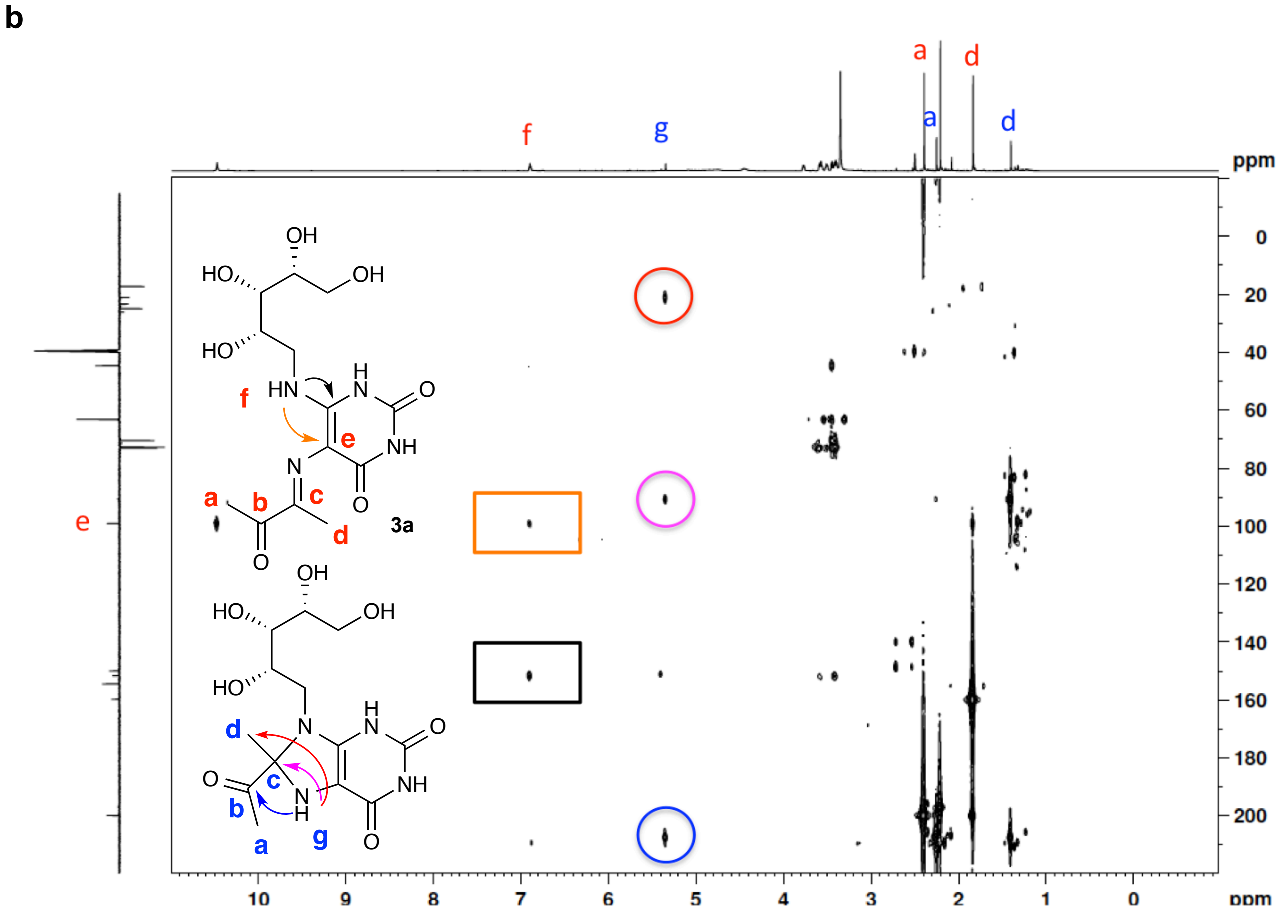

Supplementary Figure 16. (a) HSQC and (b) HMBC NMR spectrum of 3a in DMSO-d_6.

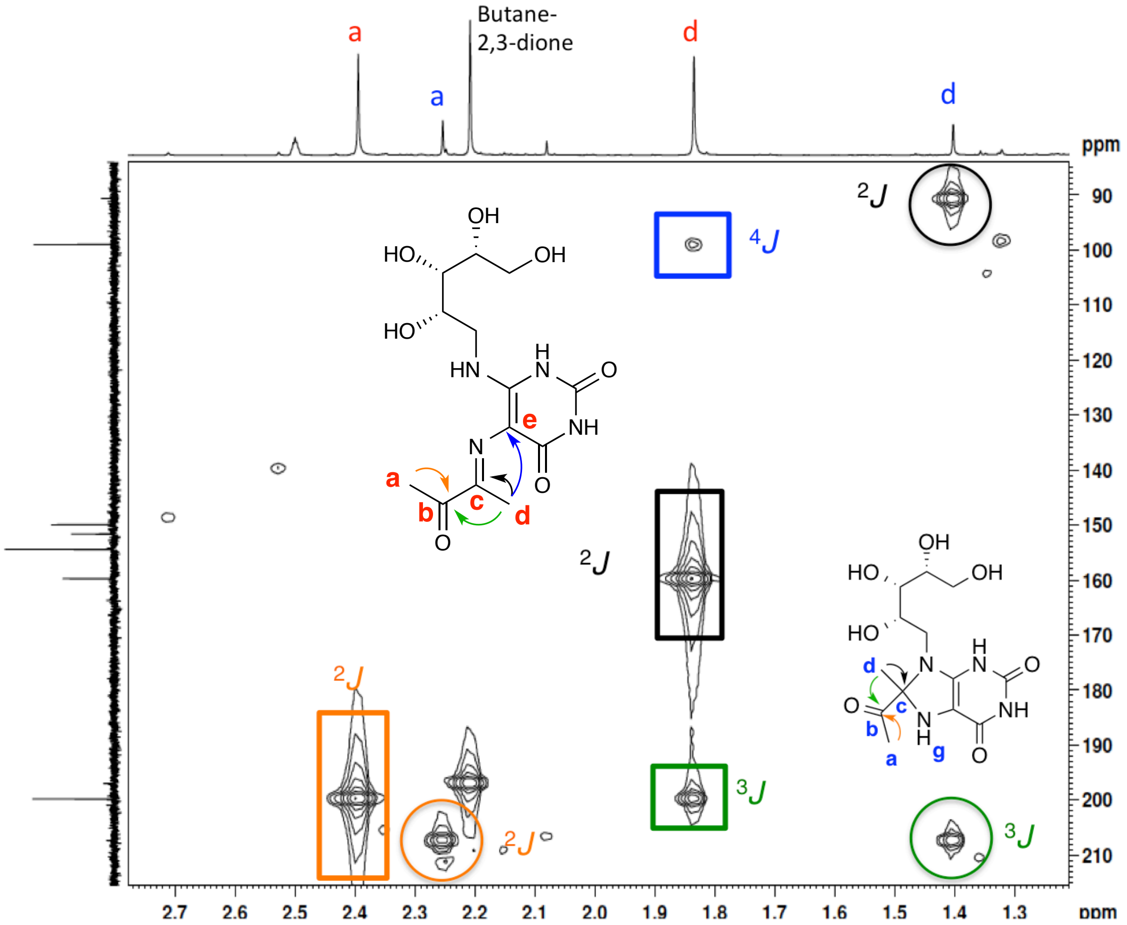

Supplementary Figure 17. Zoomed HMBC NMR spectrum of 3a in DMSO-d_6.

a

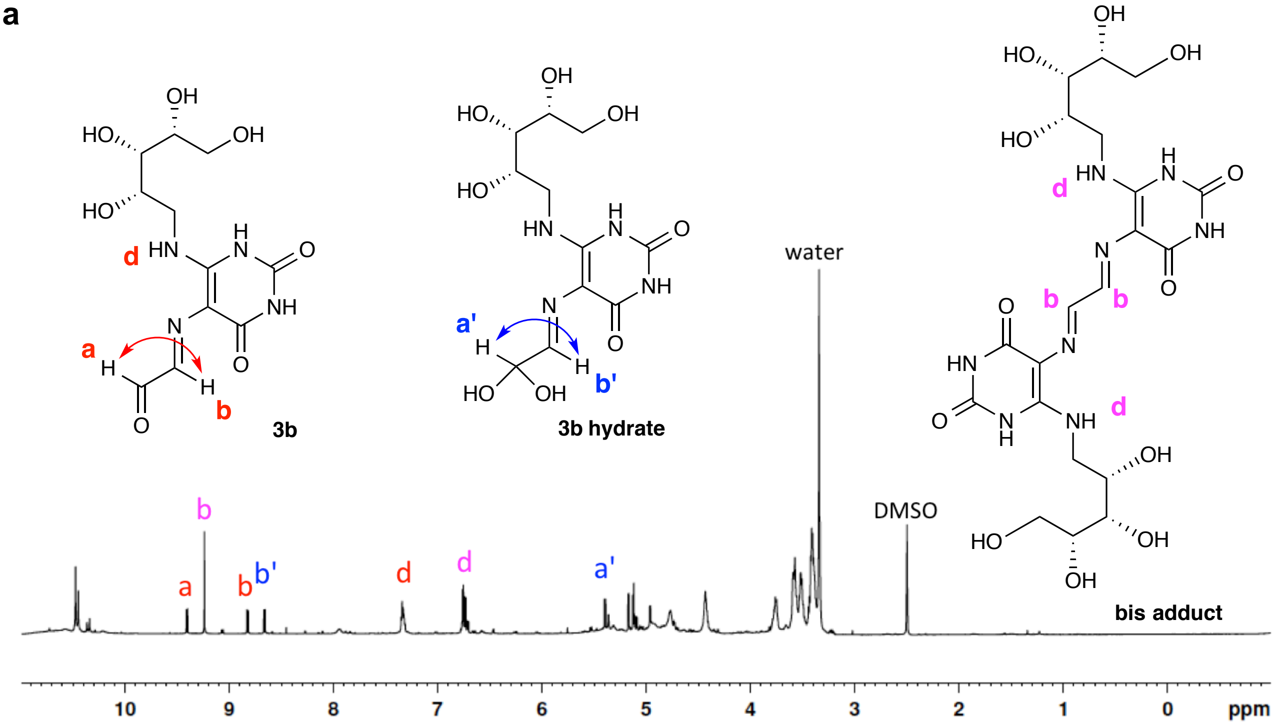

b

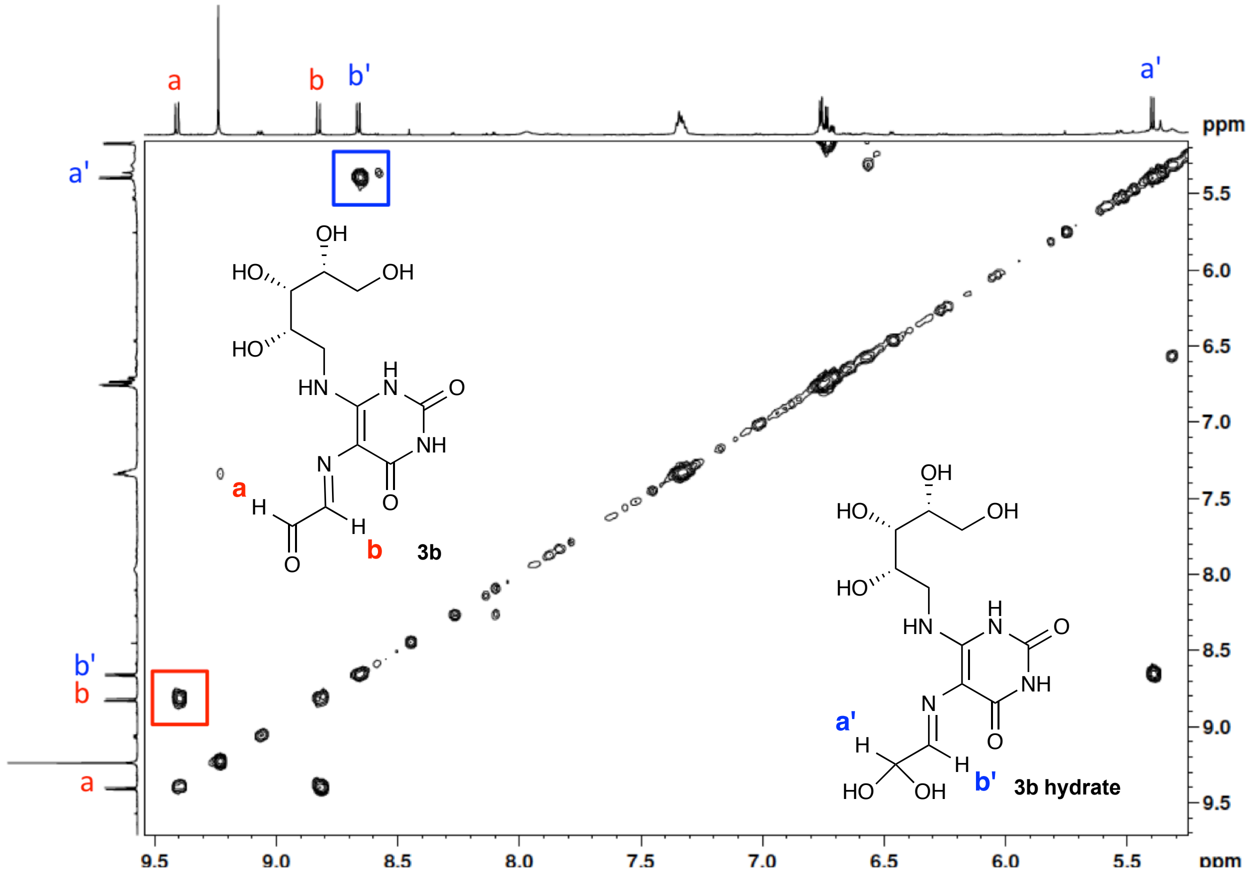

Supplementary Figure 18. (a) ^{1}H and (b) COSY NMR spectrum of 3b in DMSO-d_6.

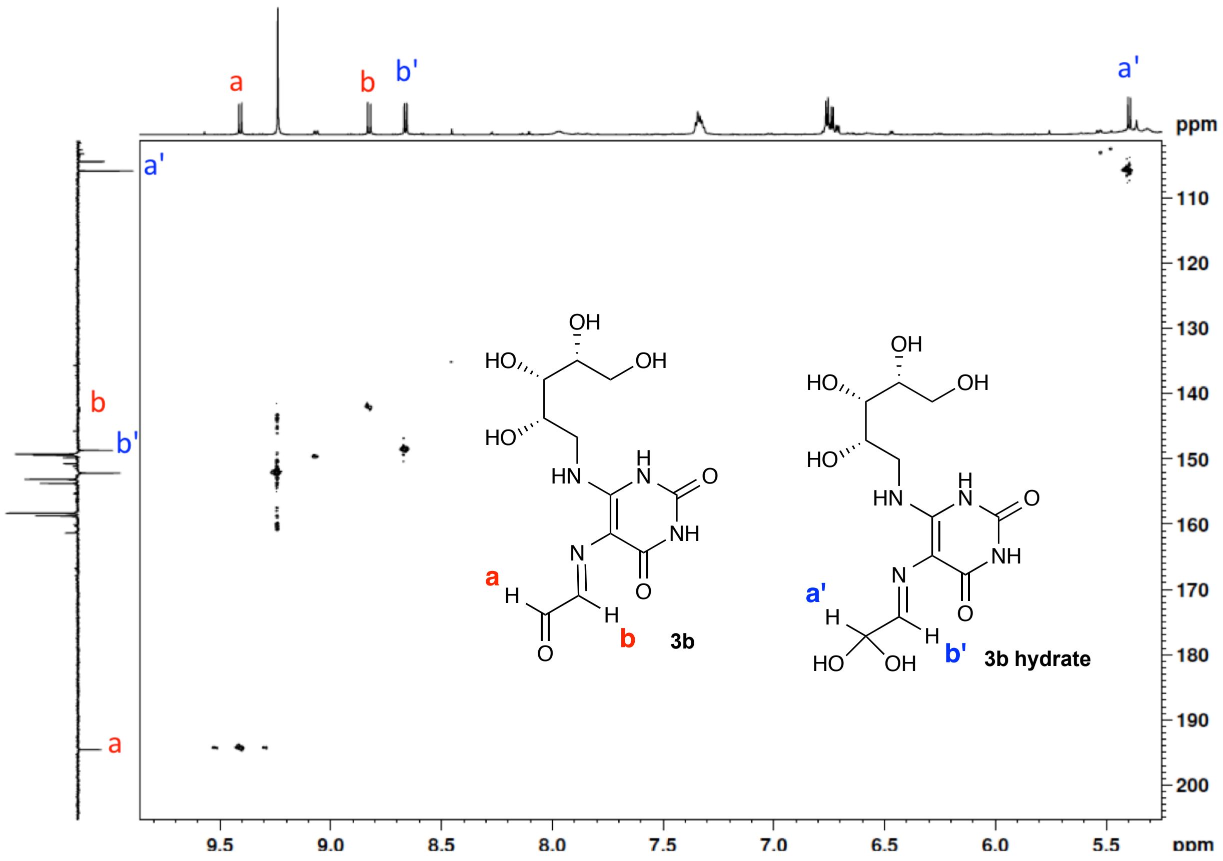

Supplementary Figure 19. HSQC NMR spectrum of 3b in DMSO-d_6.

a

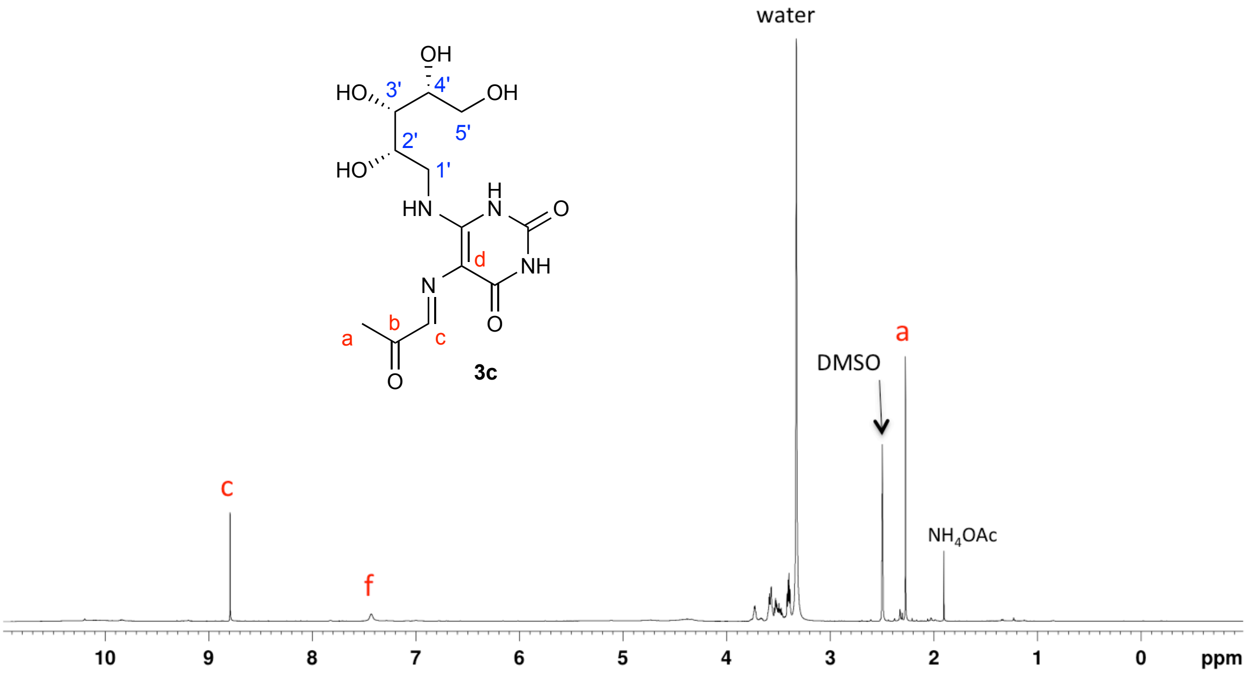

b

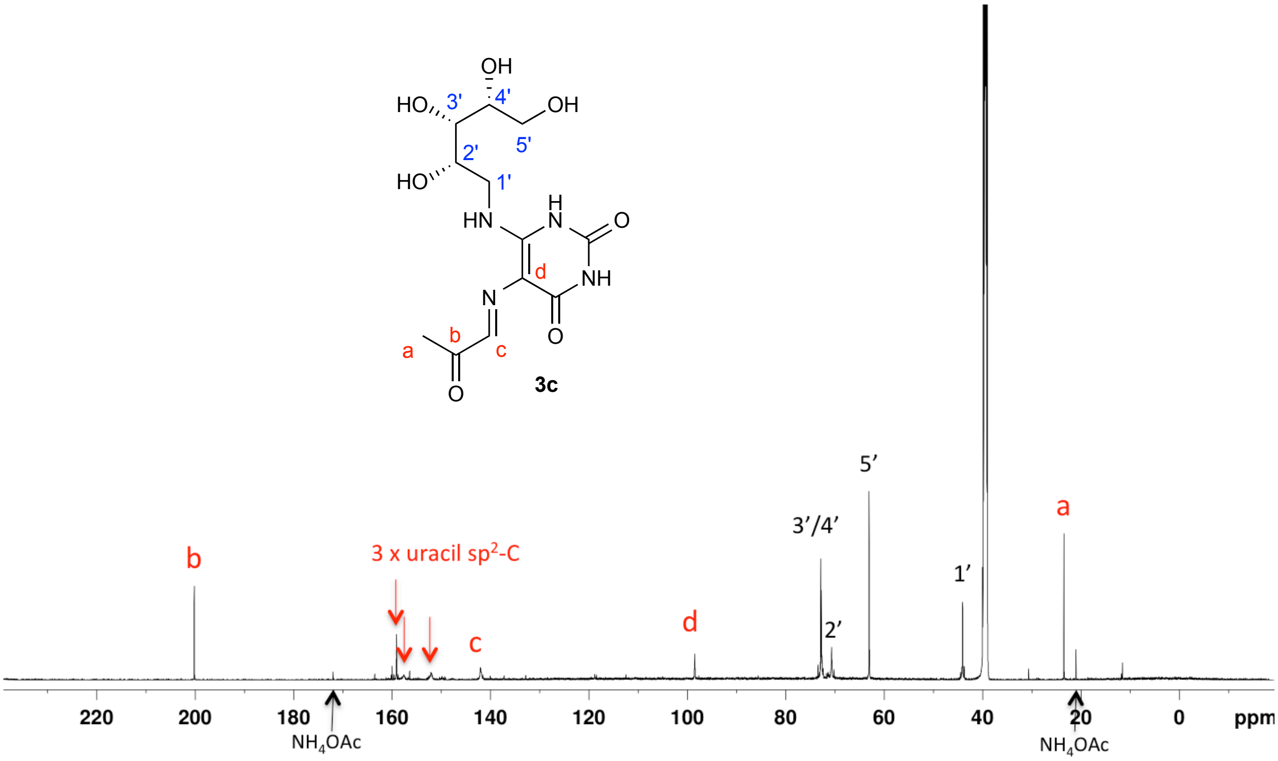

Supplementary Figure 20. Proton and carbon NMR spectra of 3c in DMSO-*d*$_6$. (a) ^{1}H NMR spectrum showing proton resonances for uracil derivative (red labels). **(b)** ^{13}**C** NMR spectrum showing ^{13}C resonances for ribityl (black labels) and uracil derivative (red labels). ^{13}C signals for the uracil ring can be broad when the spectrum is measured under NMR conditions optimised for lumazines. This could be due to a much longer relaxation time of the ring nitrogens, or signal averaging caused by slow tautomerisation between uracil form and 2,4-dihydroxypyrimidine form.

a

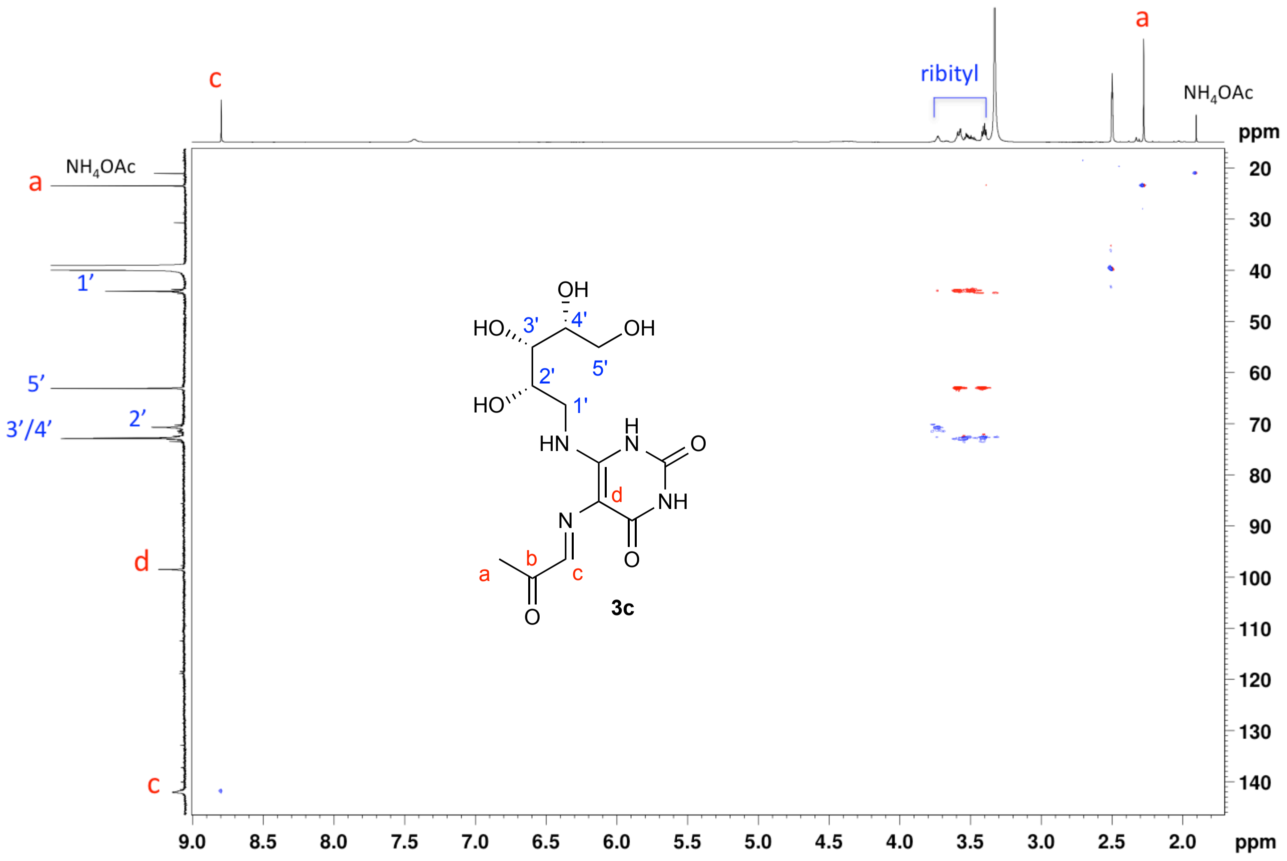

b

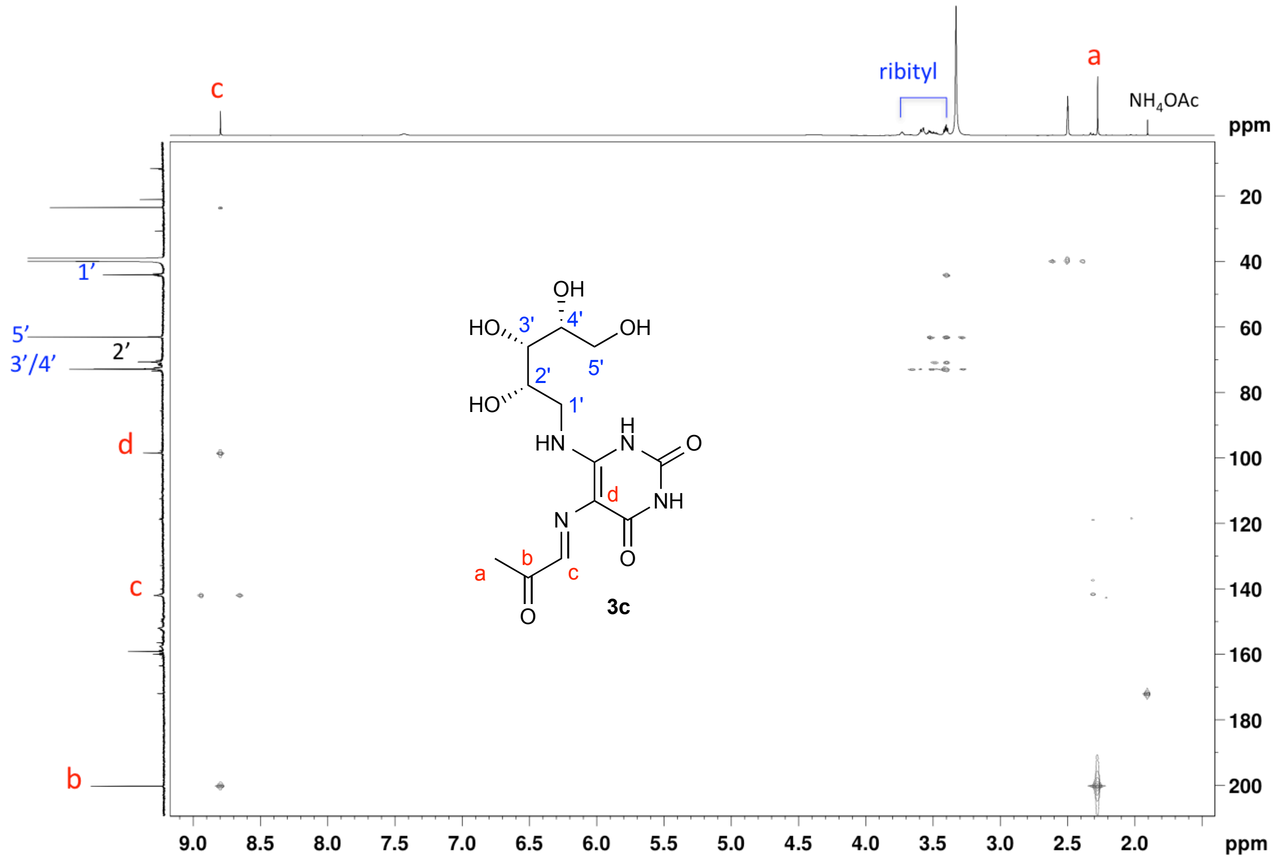

Supplementary Figure 21. (a) HSQC and (b) HMBC NMR spectrum of 3c in DMSO-d_6.

a

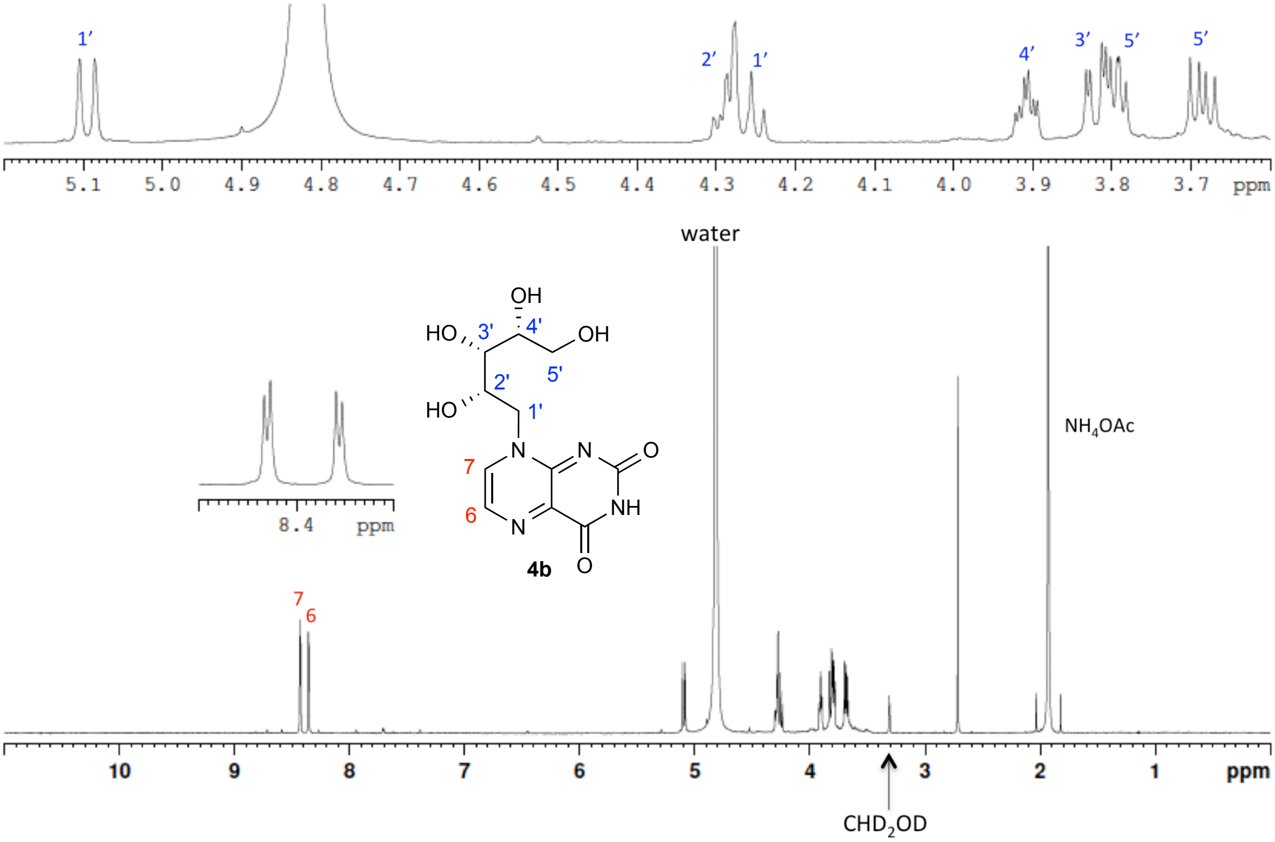

b

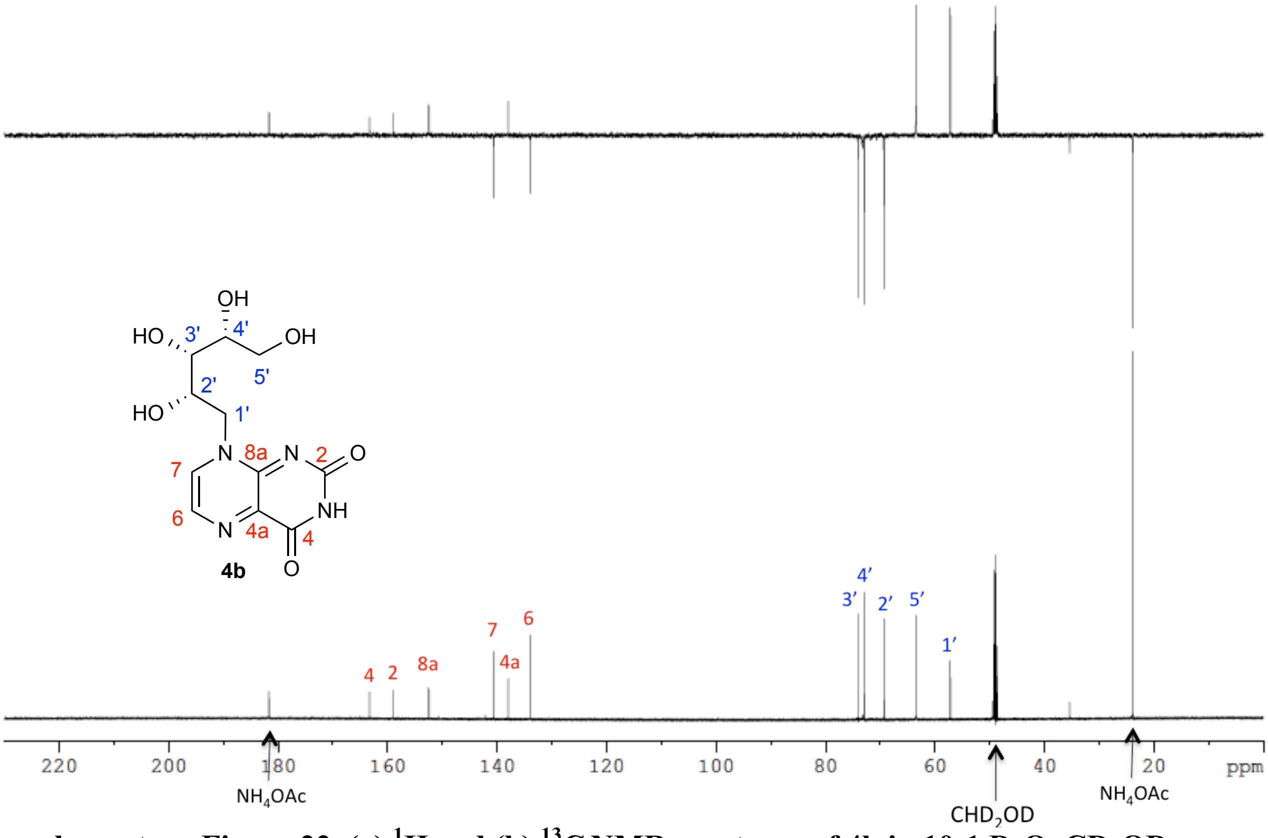

Supplementary Figure 22. (a) ^{1}H and (b) ^{13}C NMR spectrum of 4b in 10:1 D_2O–CD_3OD.

a

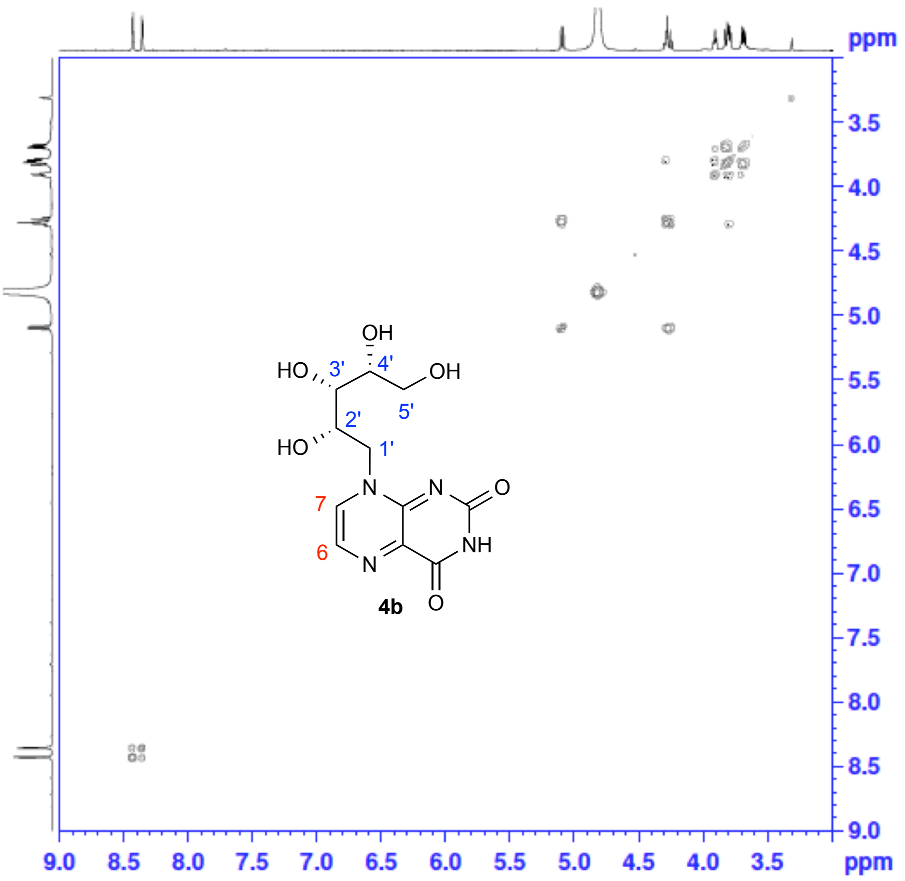

b

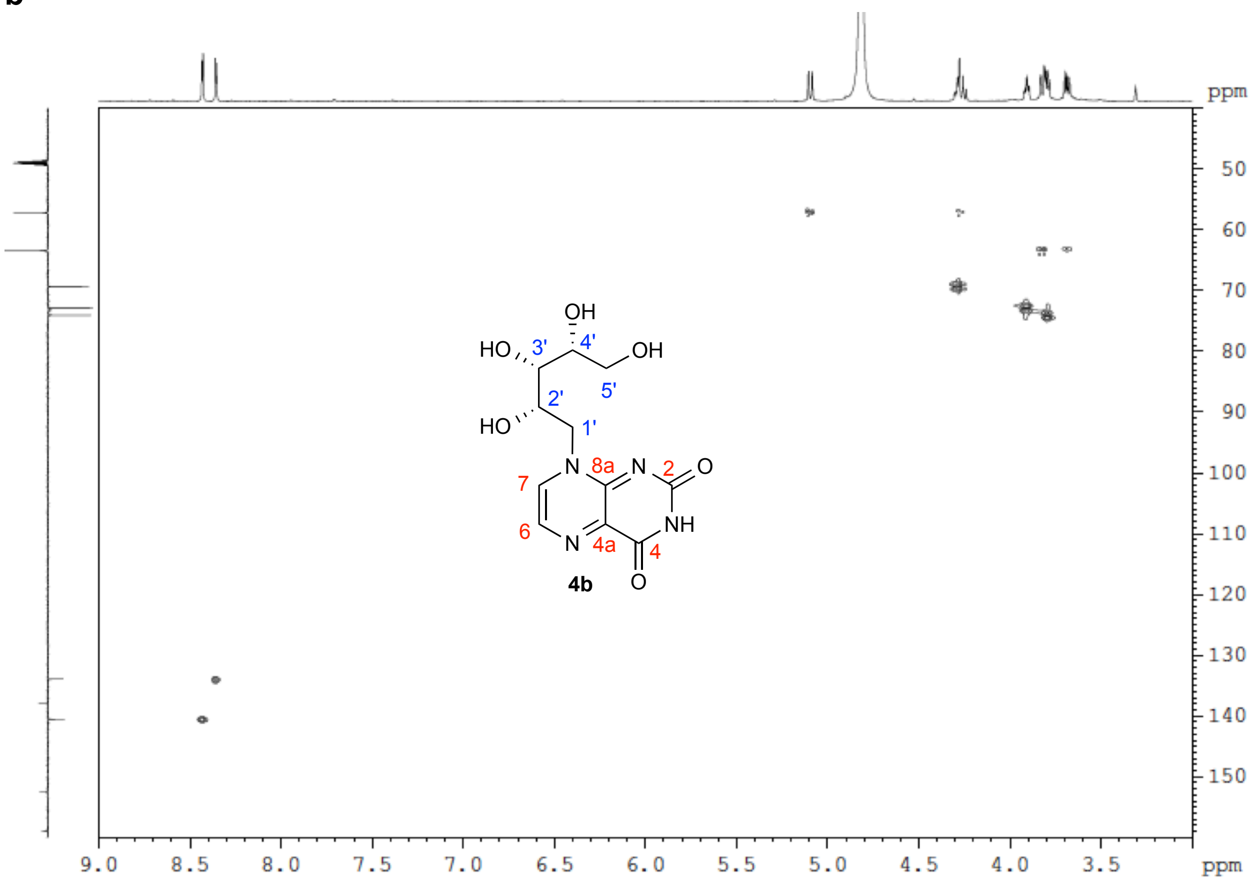

Supplementary Figure 23. (a) COSY and (b) HSQC NMR spectrum of 4b in 10:1 D_2O–CD_3OD.

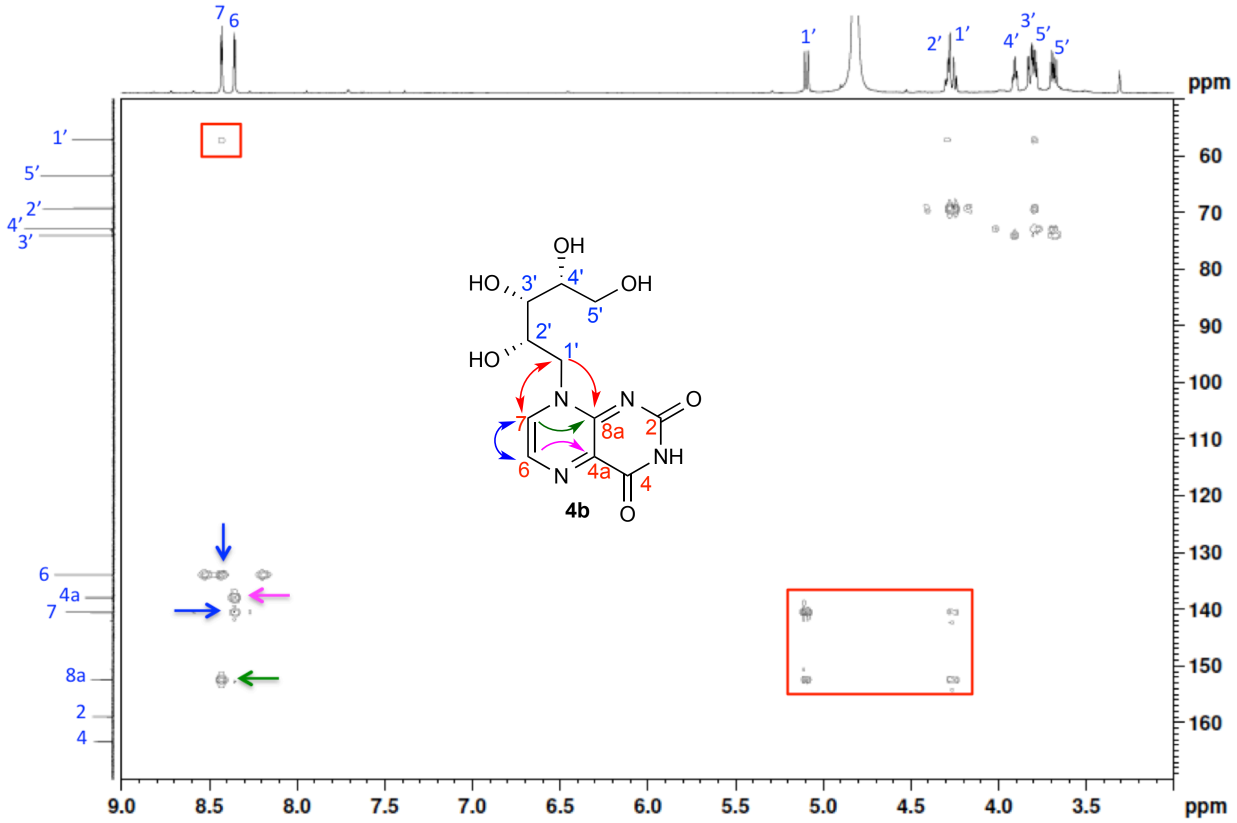

Supplementary Figure 24. HMBC NMR spectrum of 4b in 10:1 D_2O–CD_3OD.

a

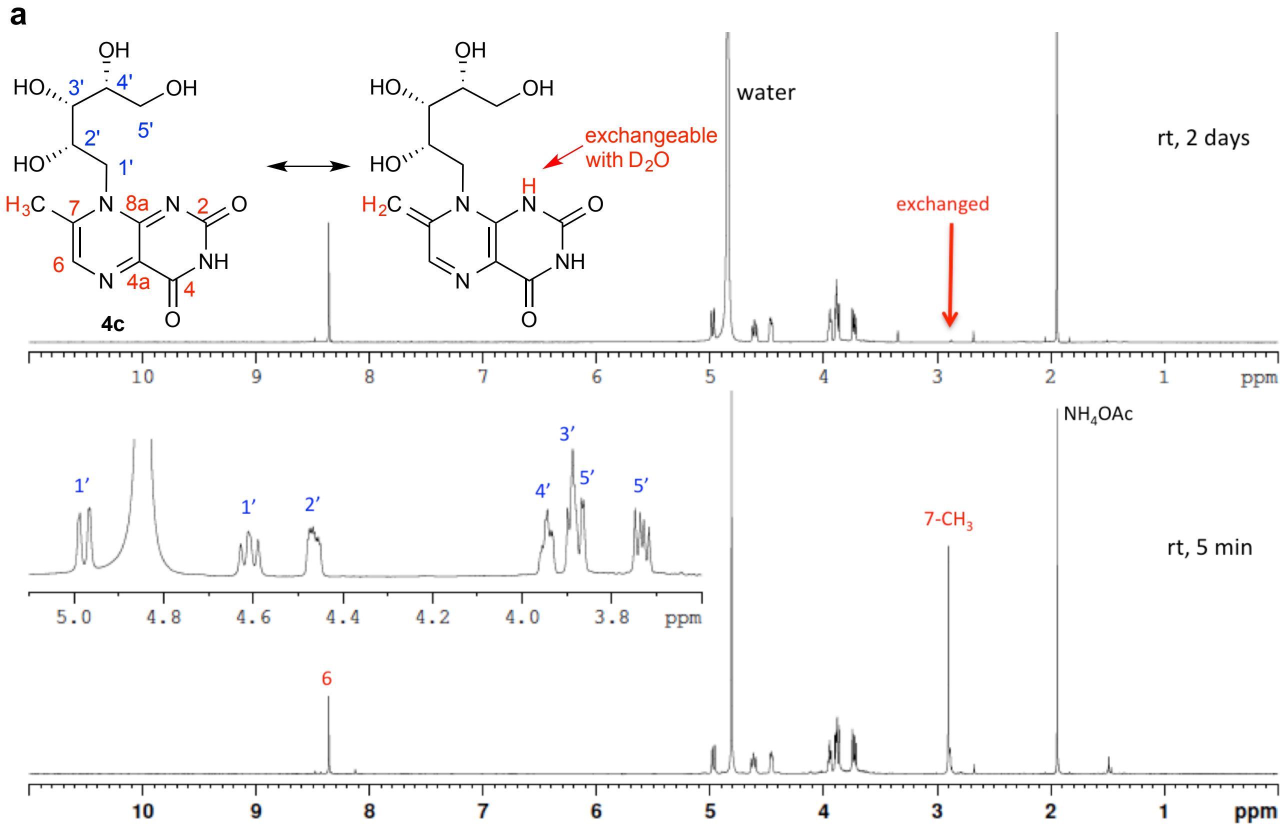

b

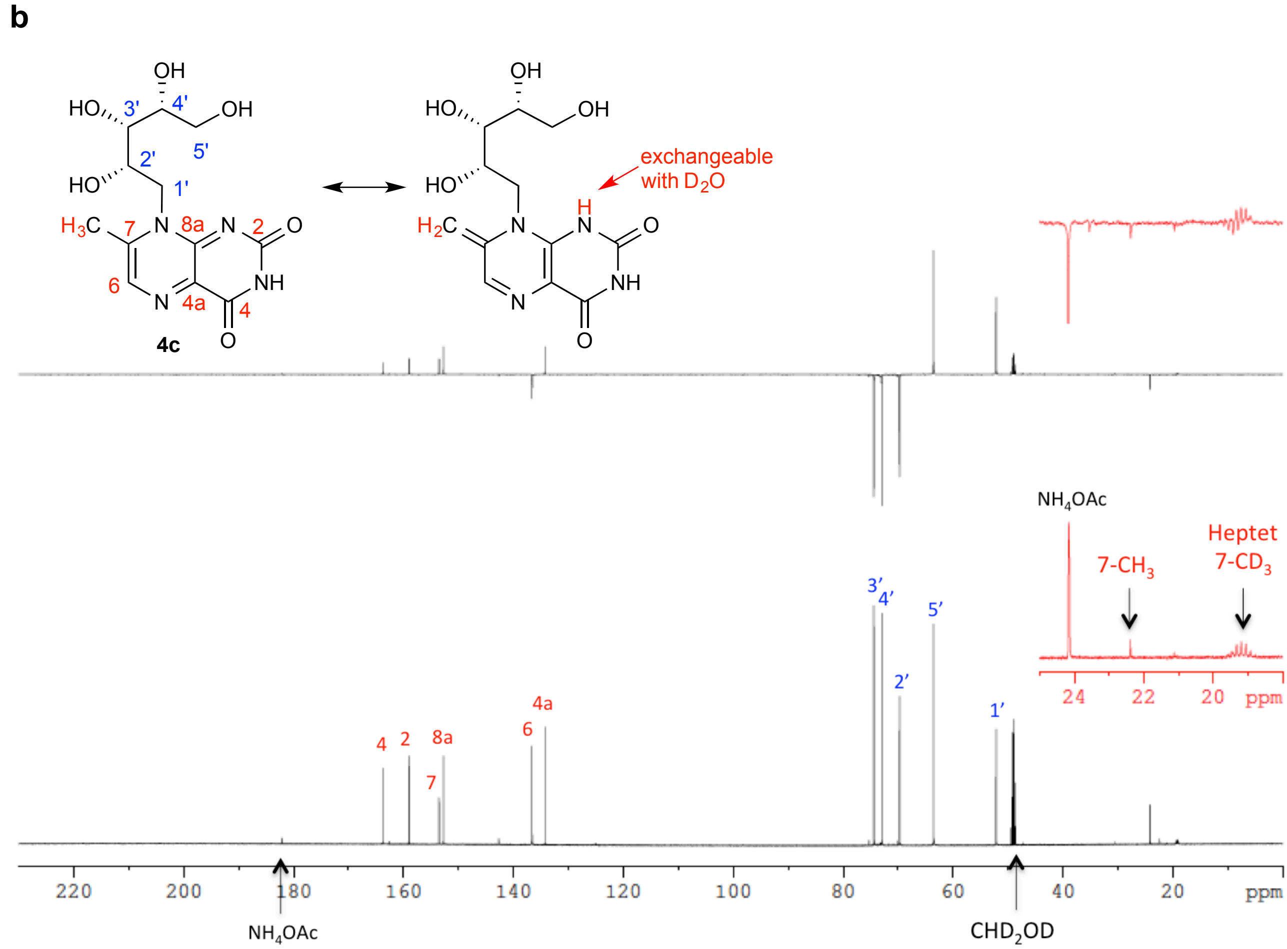

Supplementary Figure 25. (a) ^{1}H and (b) ^{13}C NMR spectrum of 4b in 10:1 D_2O–CD_3OD.

a

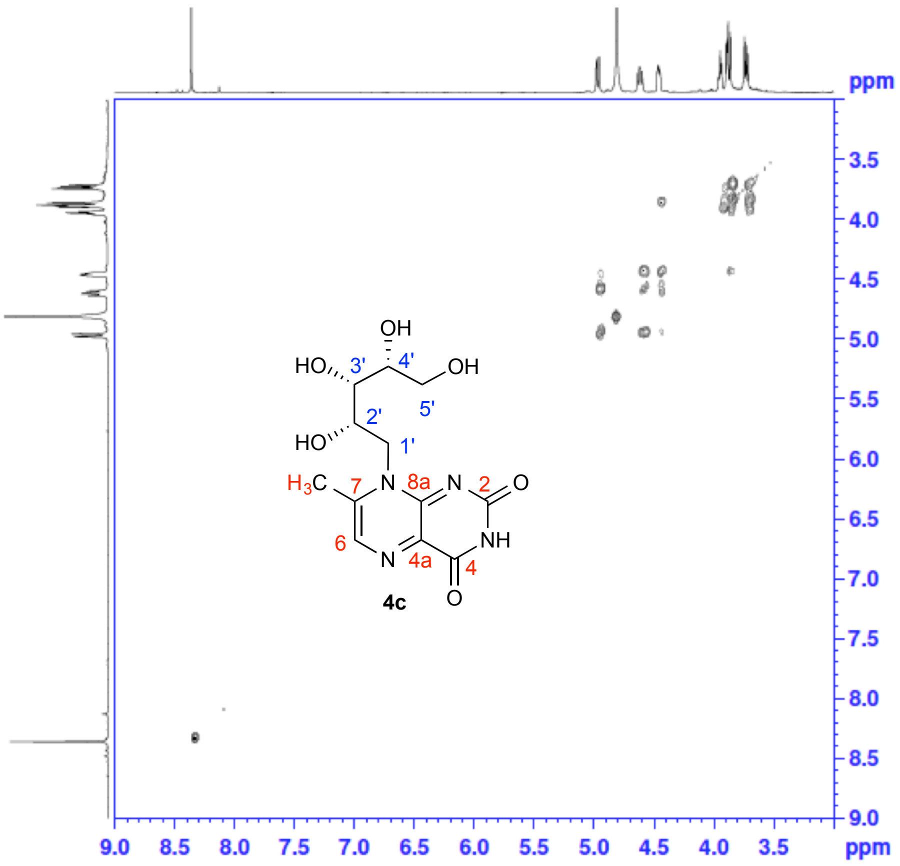

b

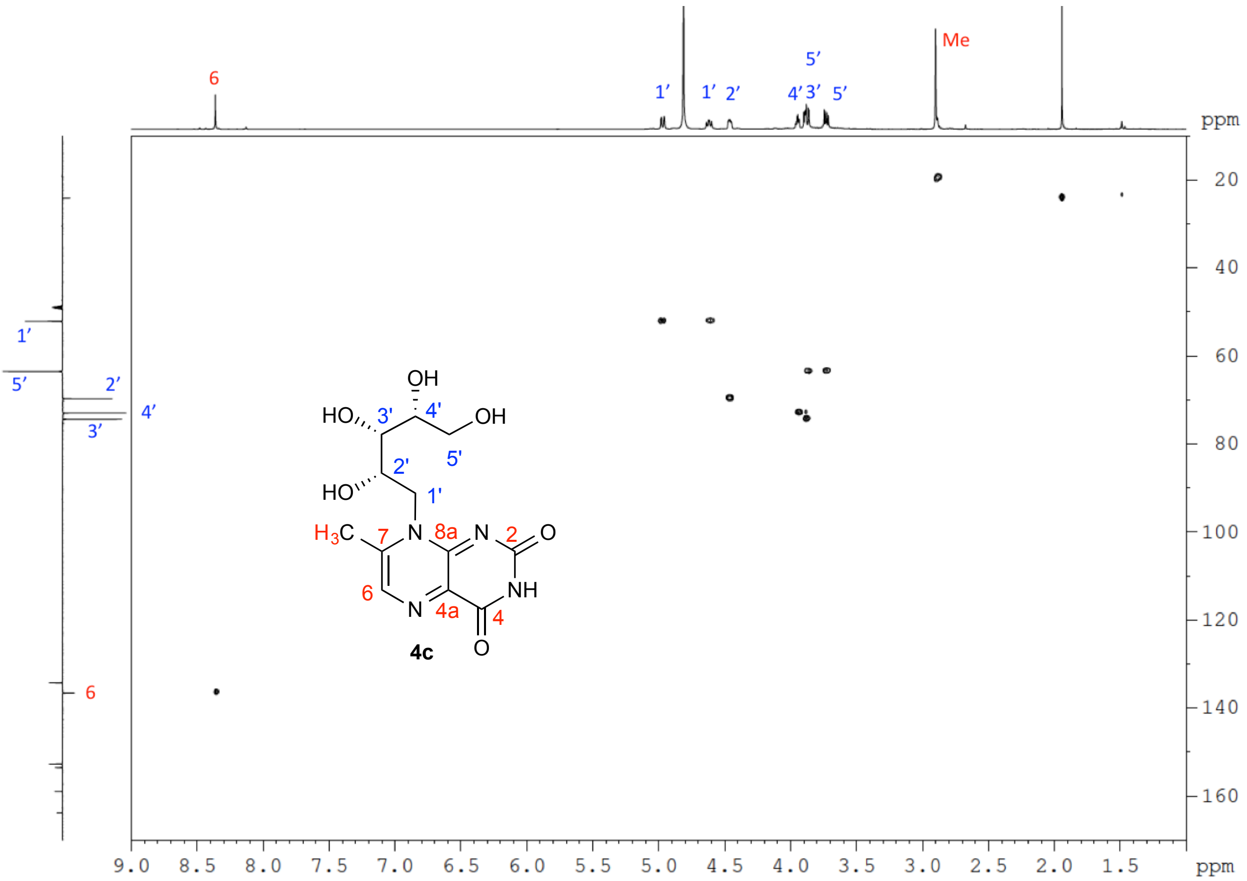

Supplementary Figure 26. (a) COSY and (b) HSQC NMR spectrum of 4c in 10:1 D_2O–CD_3OD.

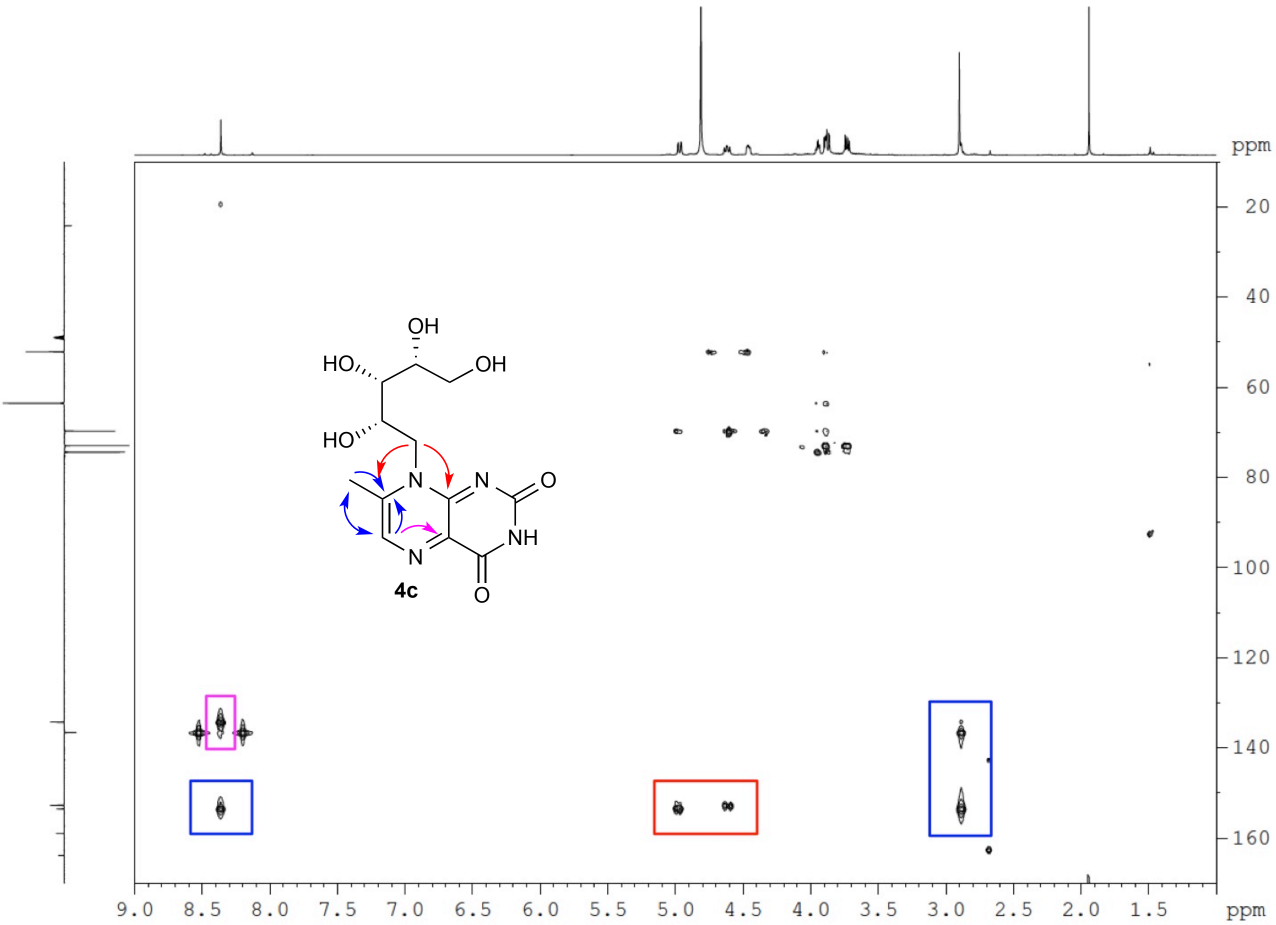

Supplementary Figure 27. HMBC NMR spectrum of 4c in 10:1 D_2O–CD_3OD.

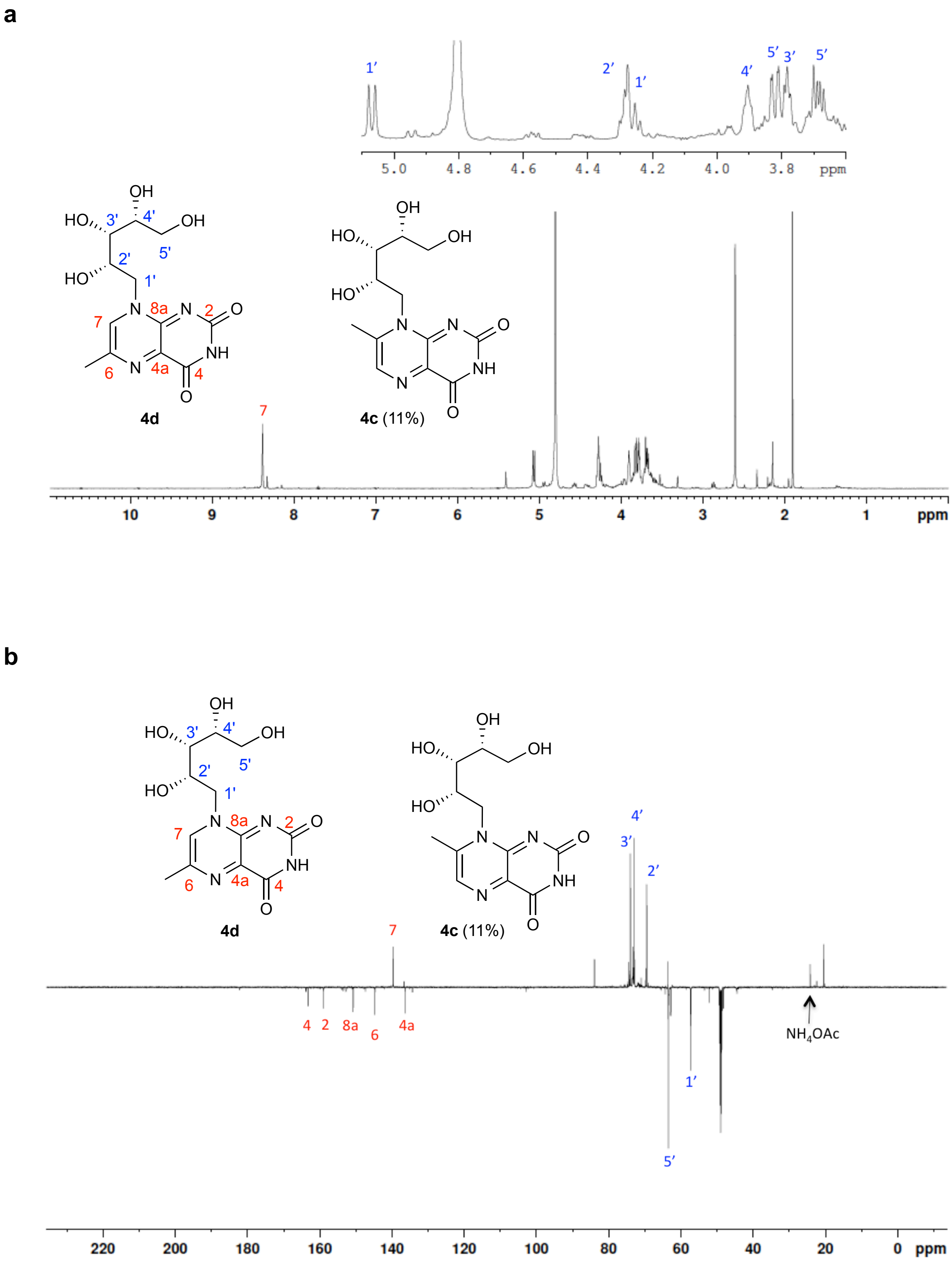

Supplementary Figure 28. (a) ^{1}H and (b) ^{13}C NMR spectrum of 4d in 10:1 D_2O–CD_3OD.

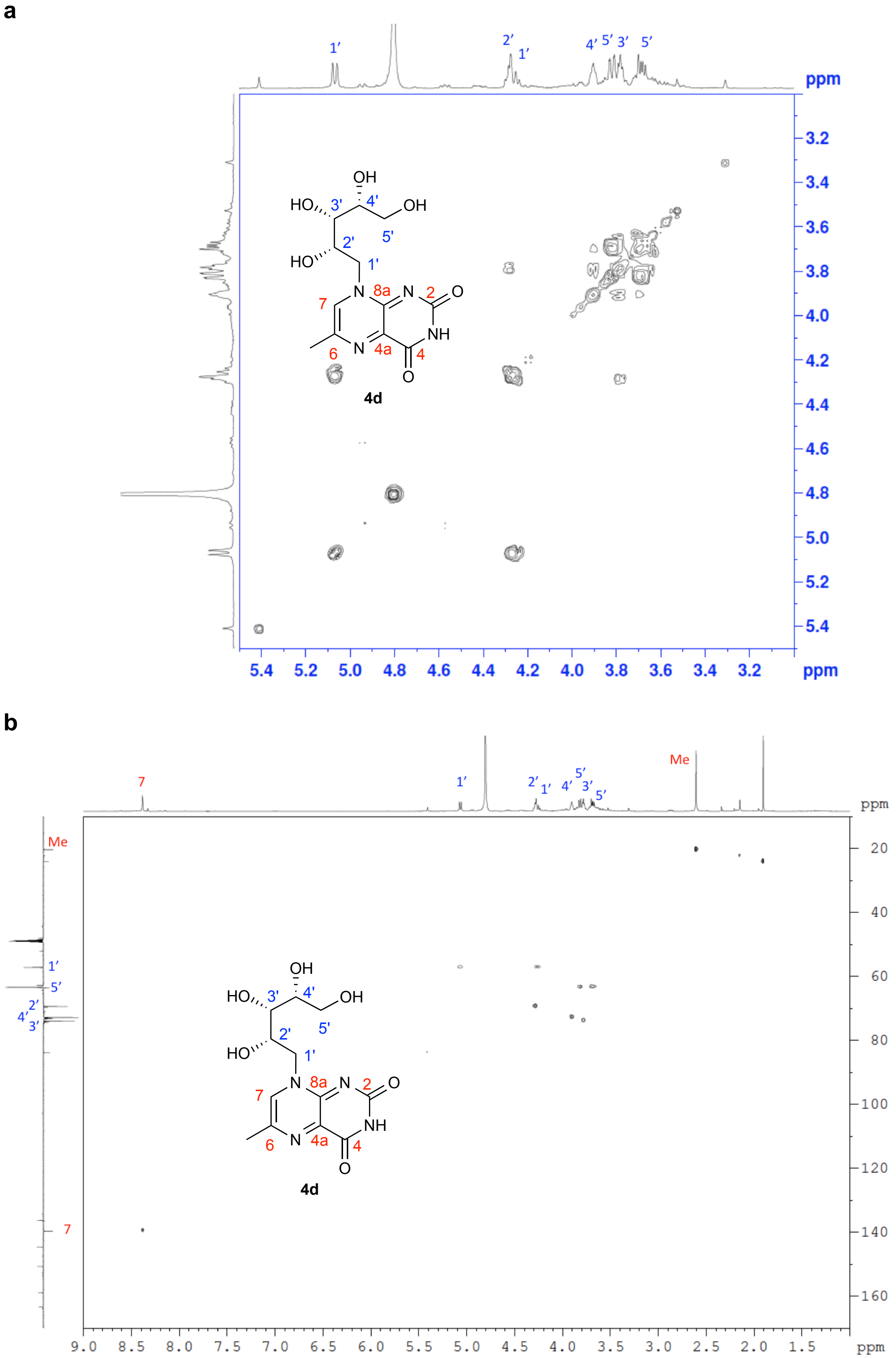

Supplementary Figure 29. (a) COSY and (b) HSQC NMR spectrum of 4d in 10:1 D_2O–CD_3OD.

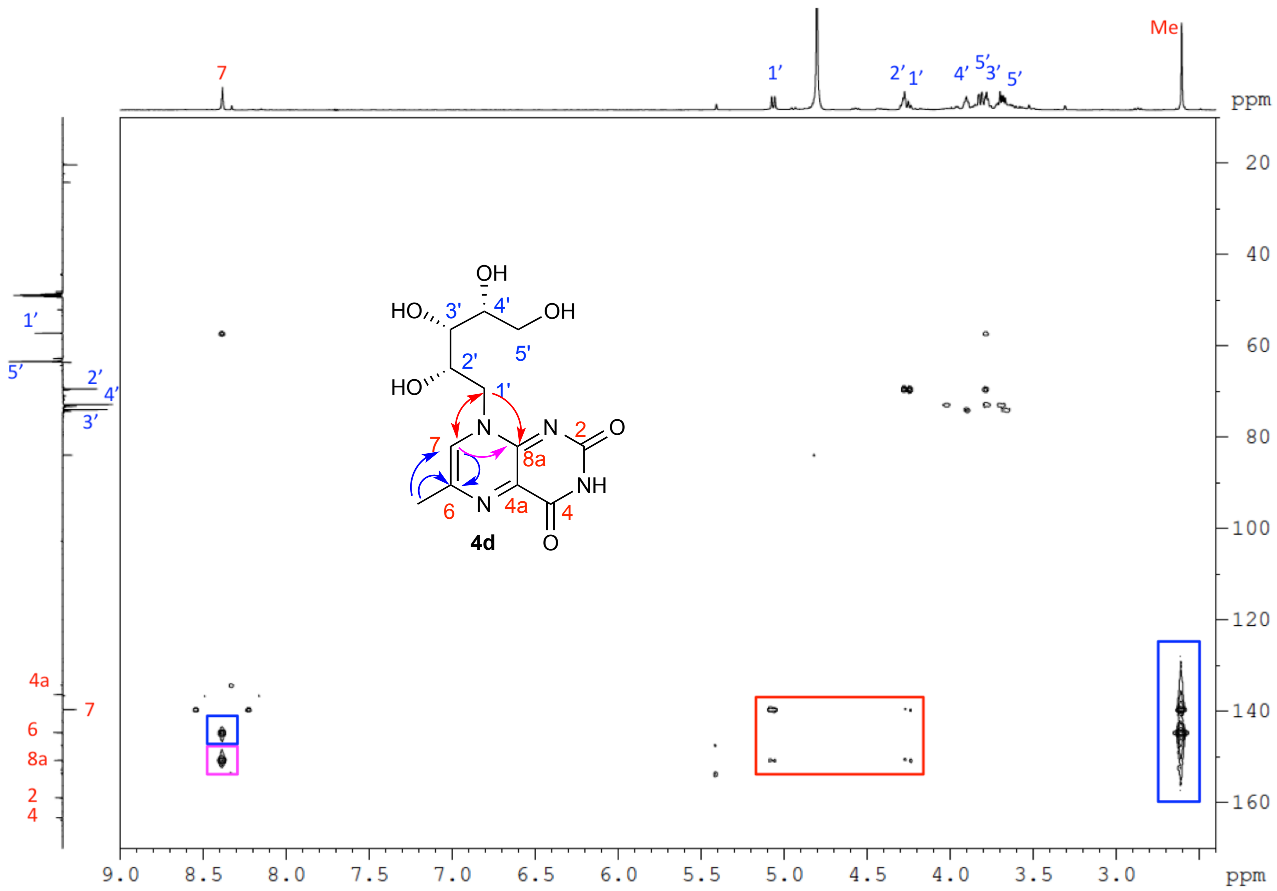

Supplementary Figure 30. HMBC NMR spectrum of 4d in 10:1 D_2O–CD_3OD.

a

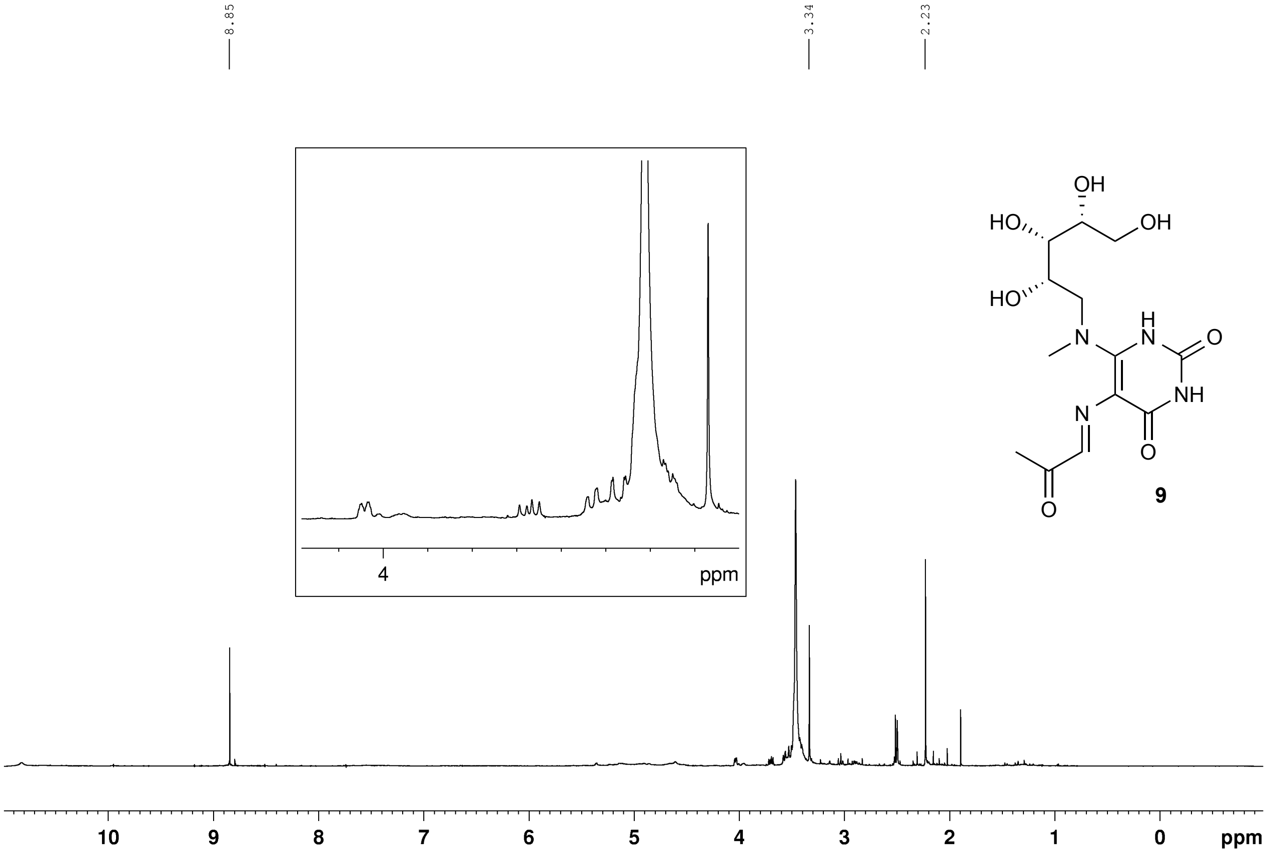

b

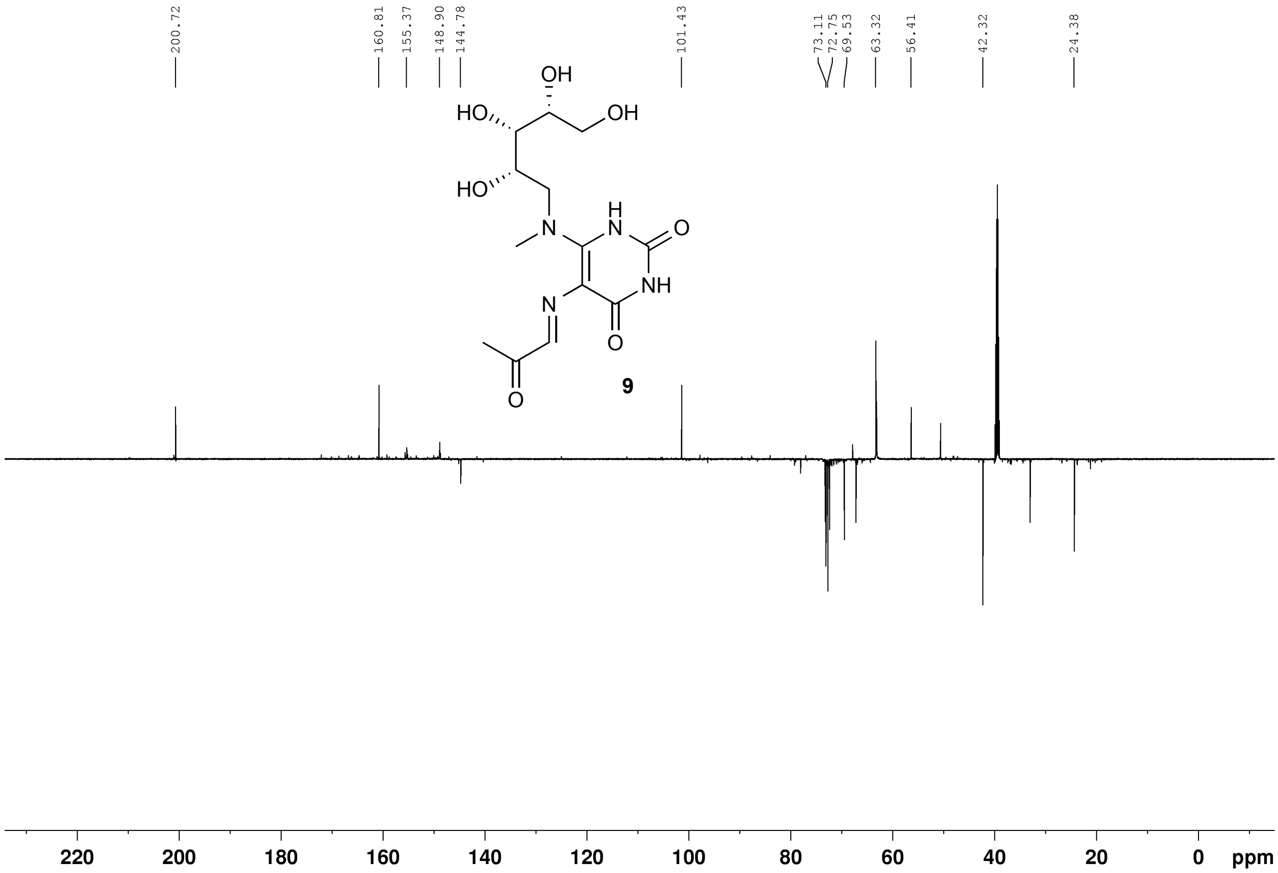

Supplementary Figure 31. (a) ^{1}H and (b) ^{13}C NMR spectrum of 9 in DMSO-d_6.

a

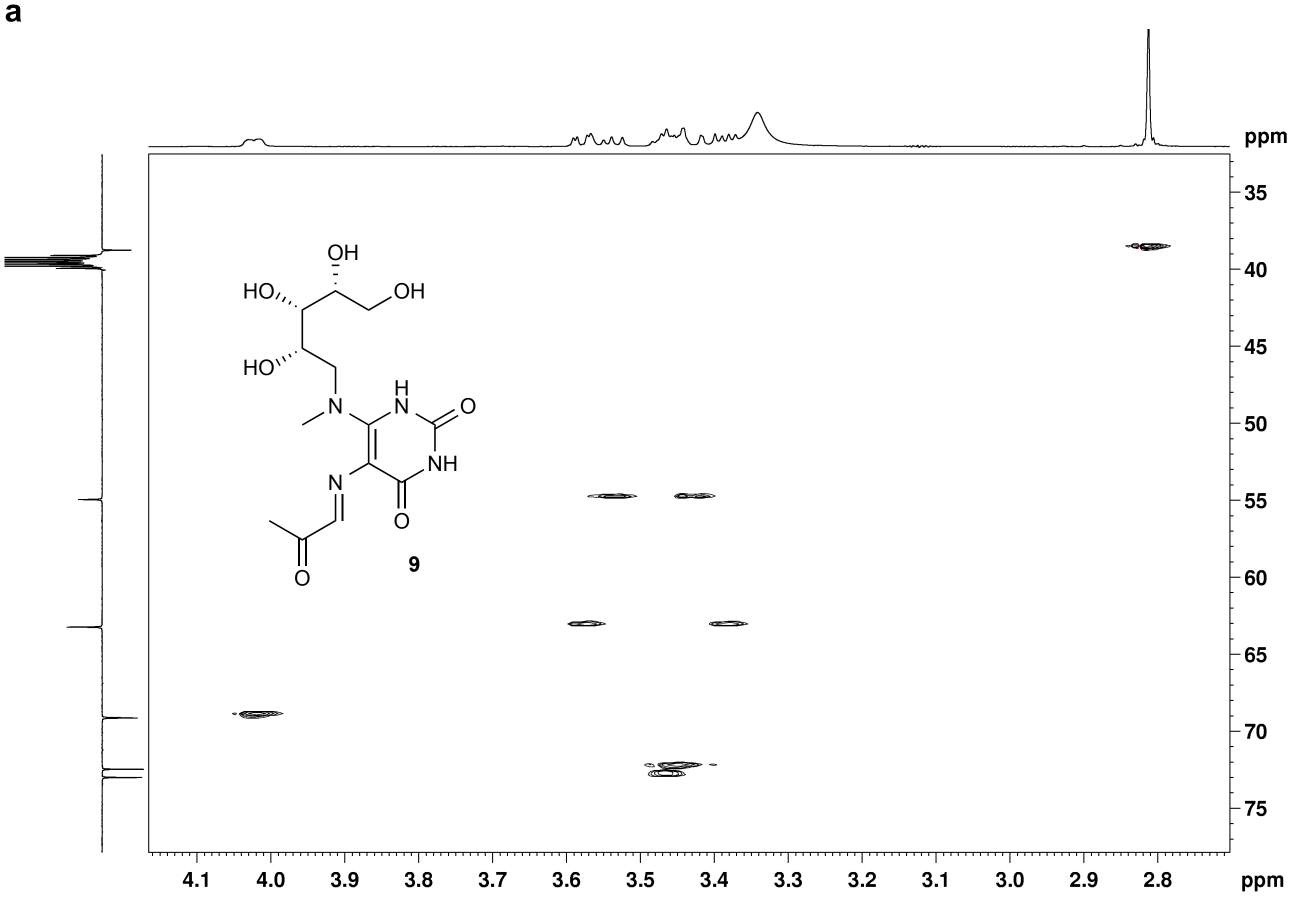

b

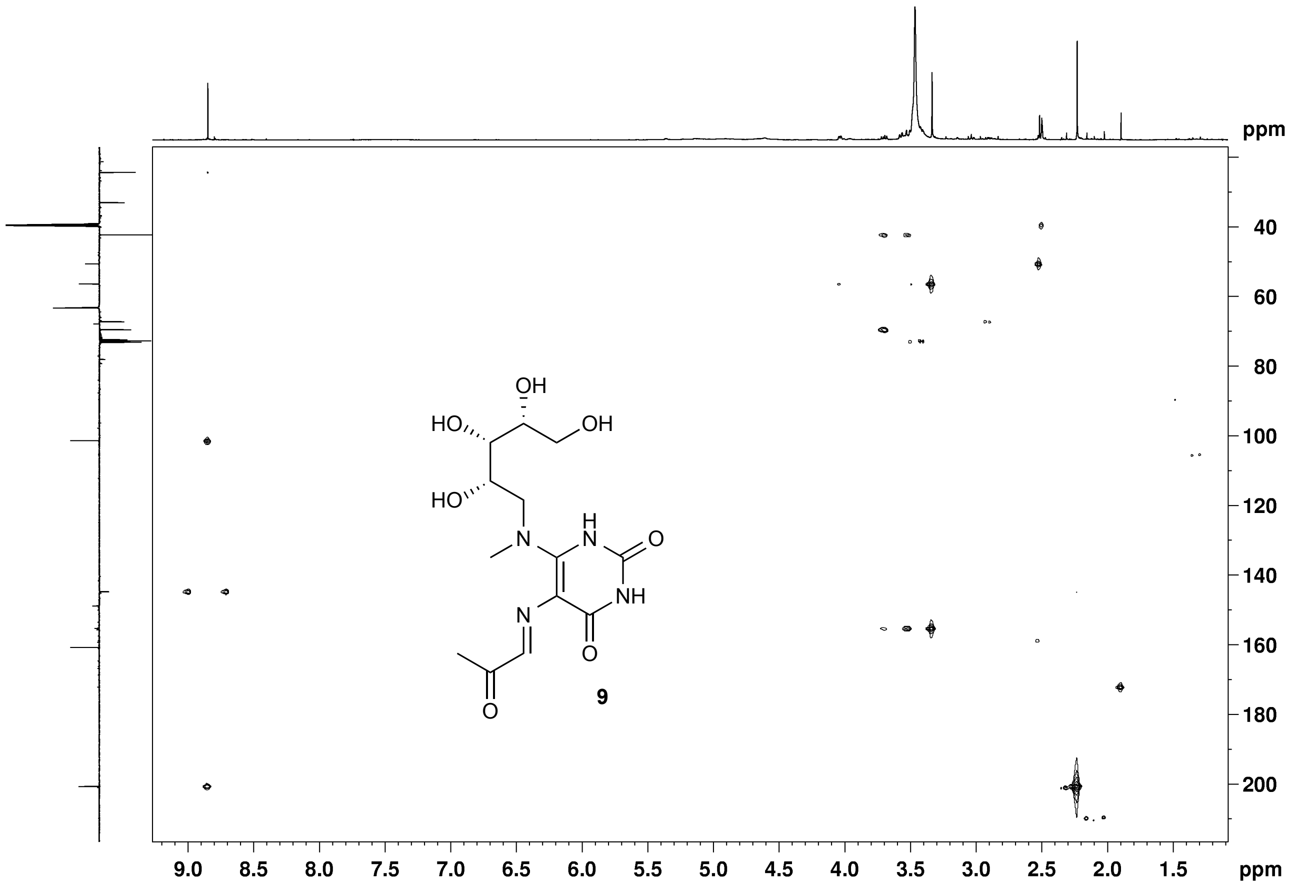

Supplementary Figure 32. (a) HSQC and (b) HMBC NMR spectrum of 9 in DMSO-d_6.

a

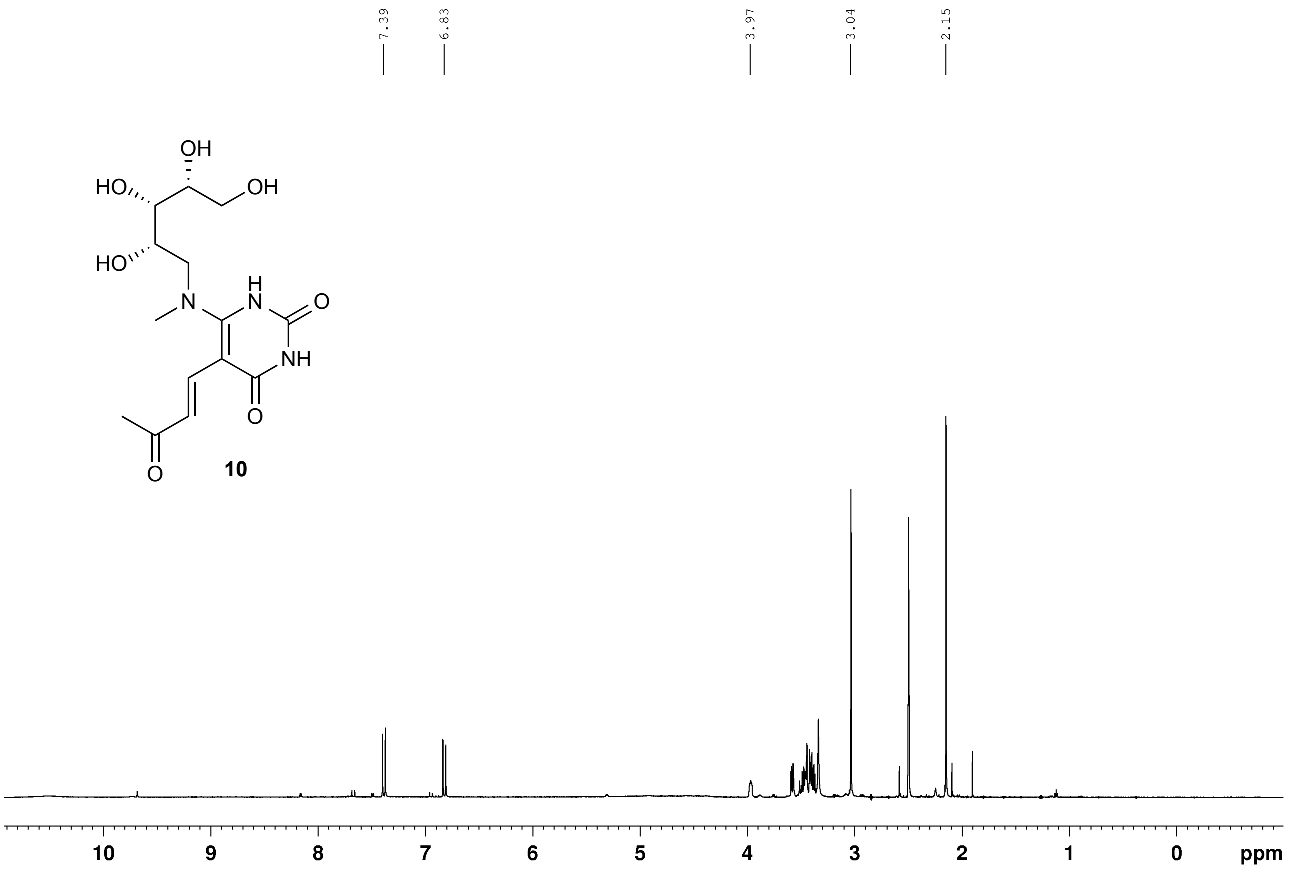

b

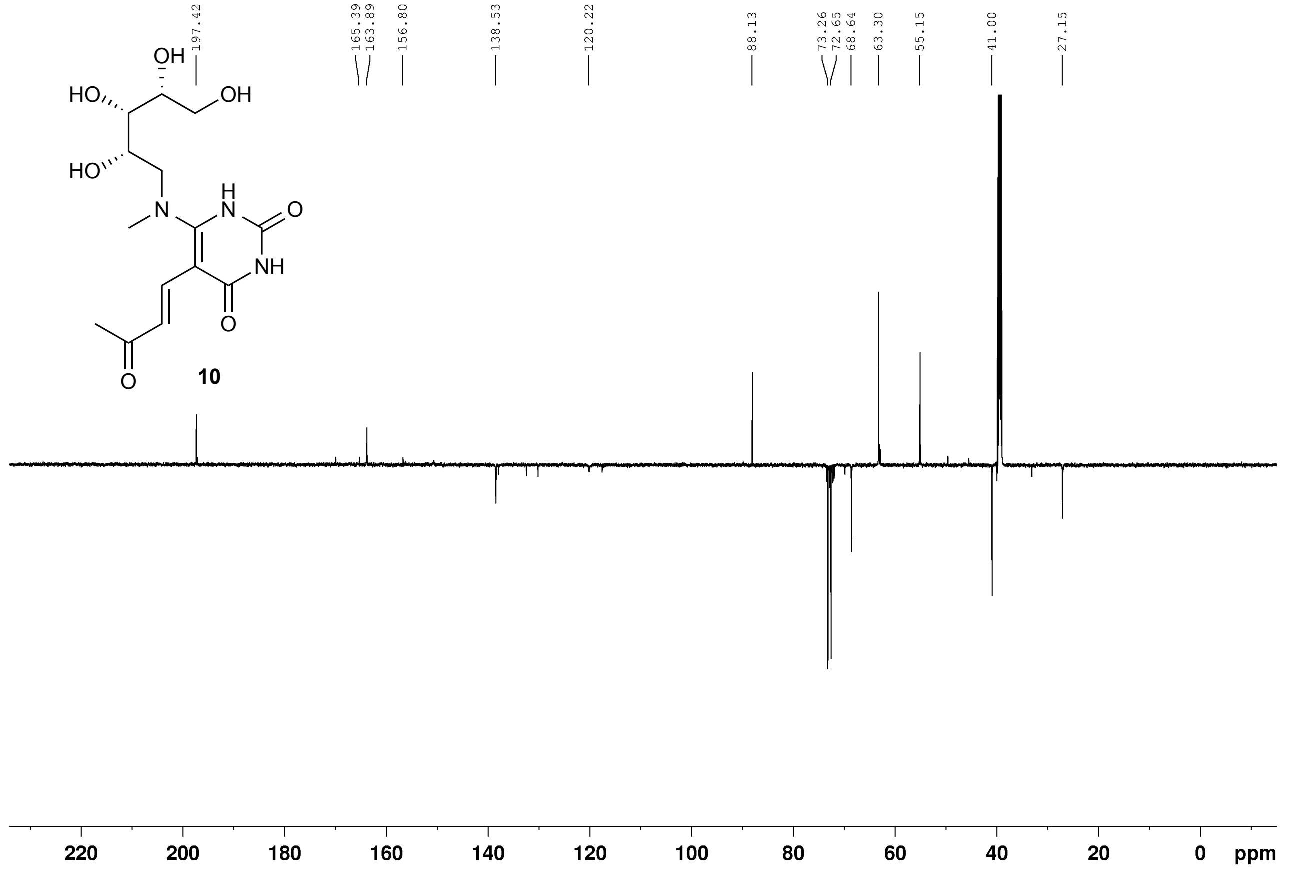

Supplementary Figure 33. (a) ^{1}H and (b) ^{13}C NMR spectrum of 10 in DMSO-d_6.

a

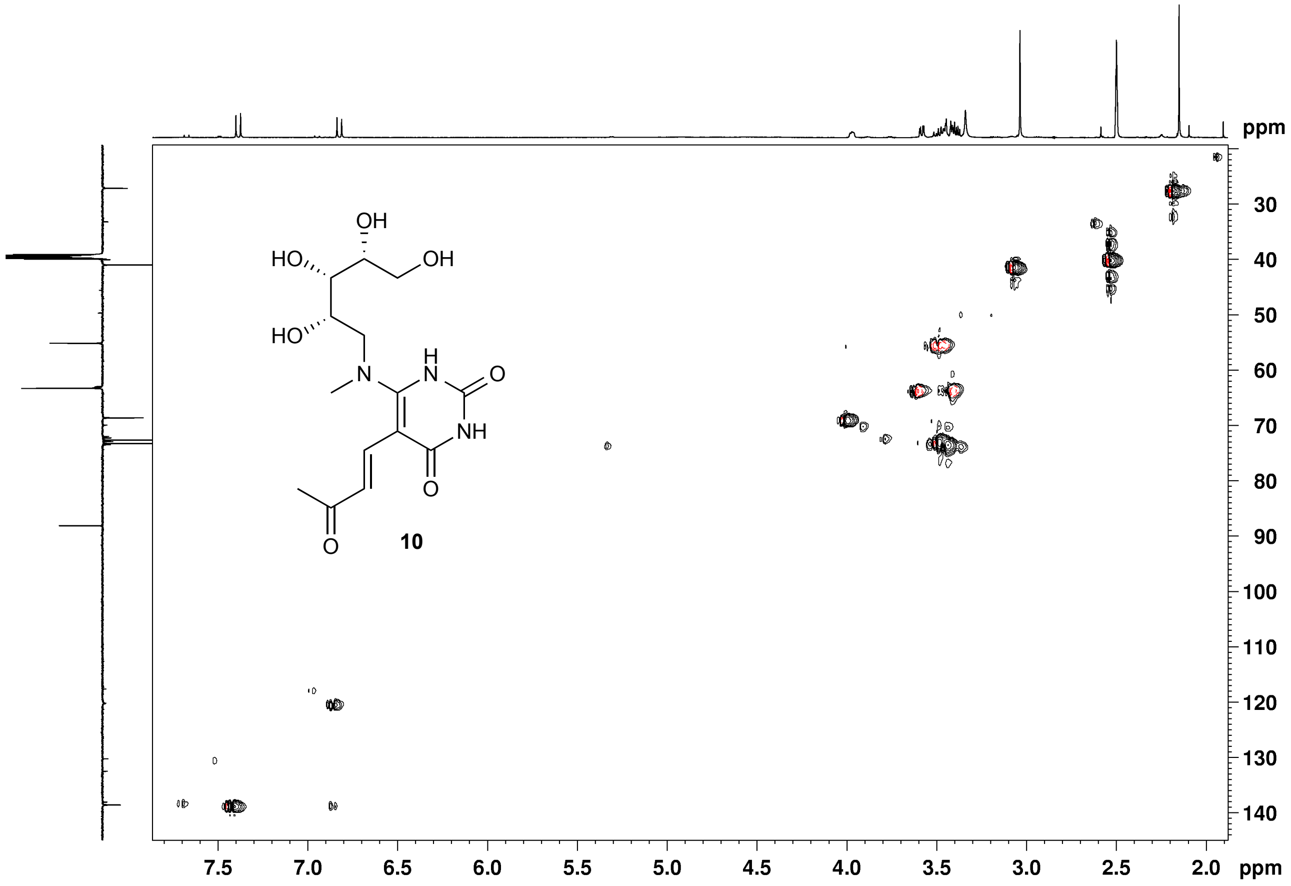

b

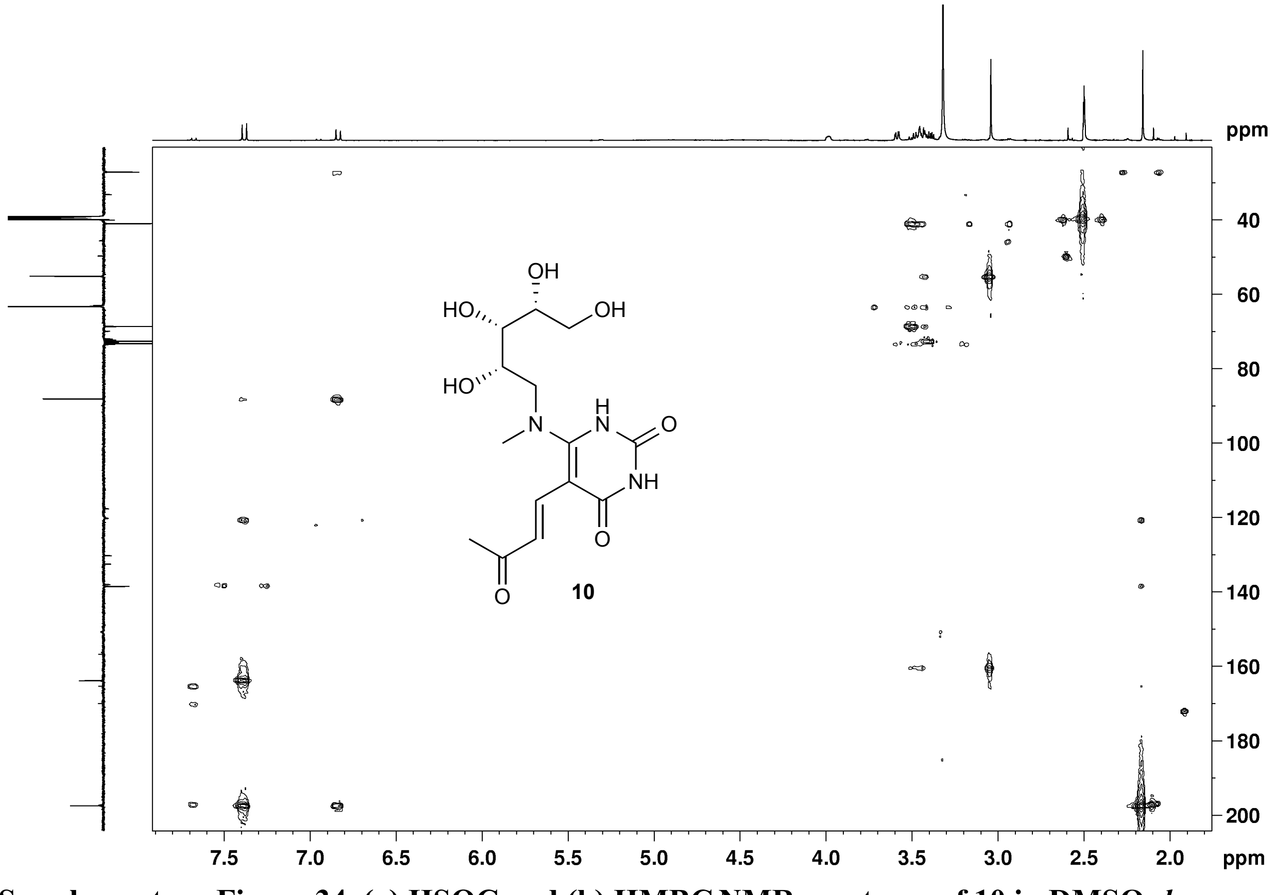

Supplementary Figure 34. (a) HSQC and (b) HMBC NMR spectrum of 10 in DMSO-d_6.

a

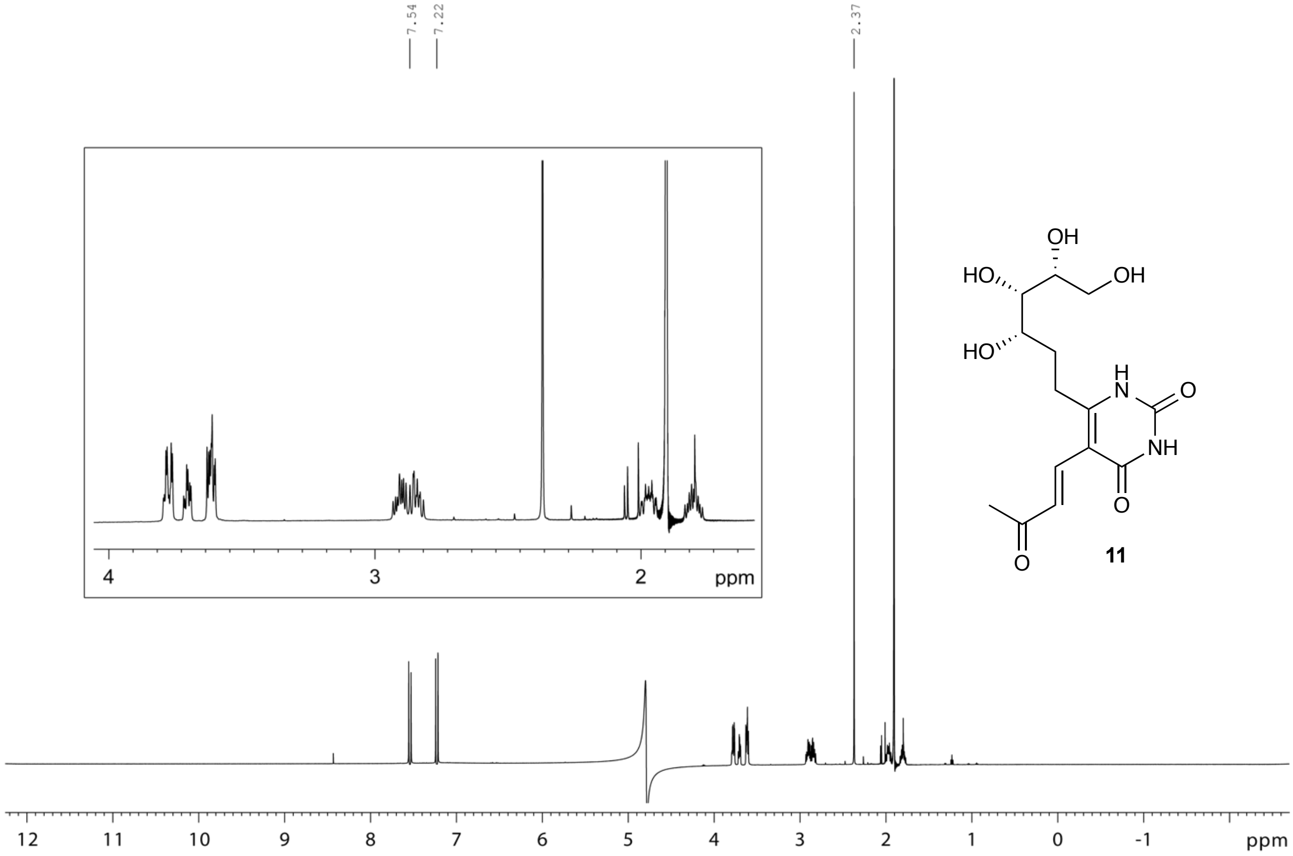

b

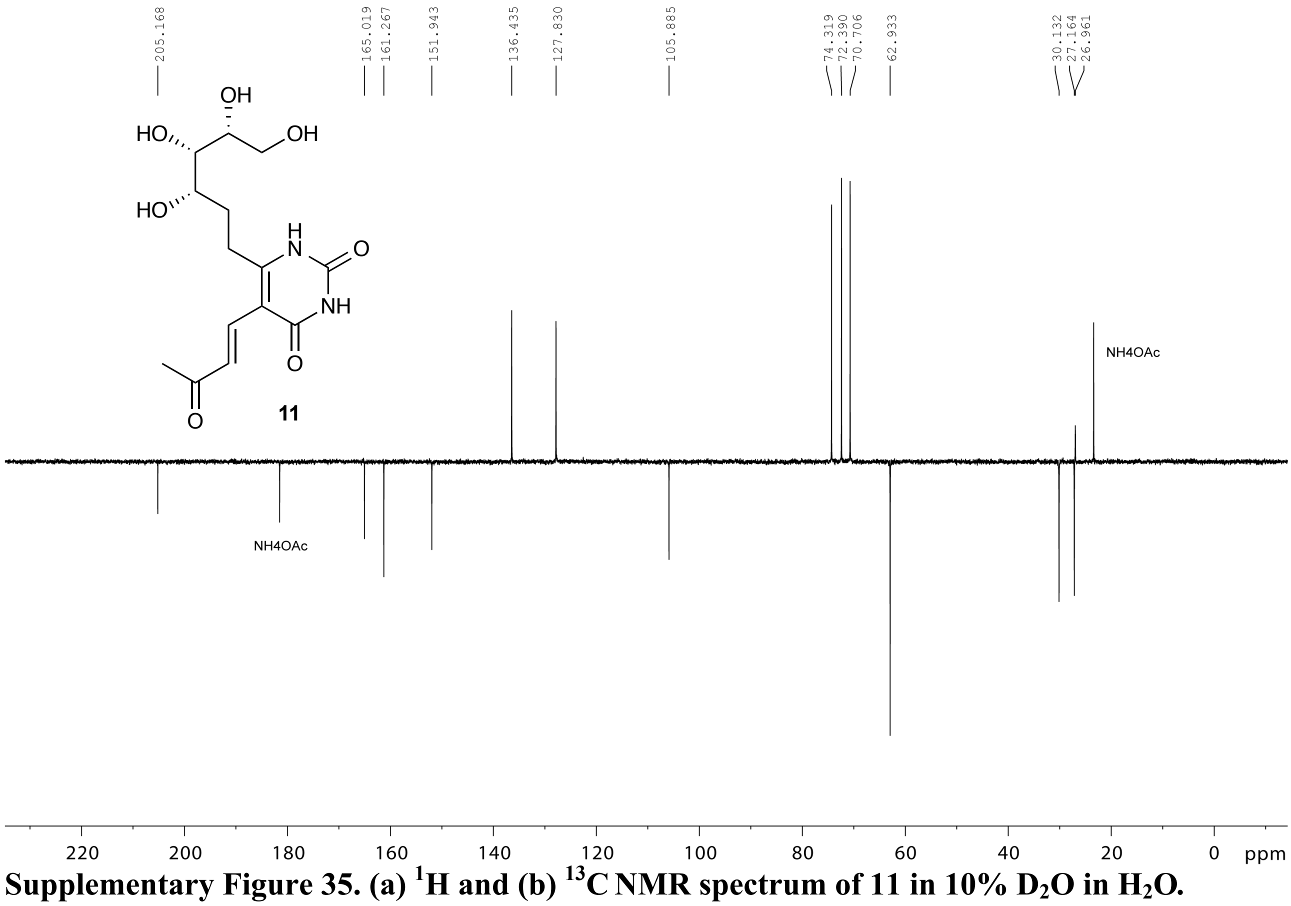

Supplementary Figure 35. (a) ^{1}H and (b) ^{13}C NMR spectrum of 11 in 10% D_2O in H_2O.

a

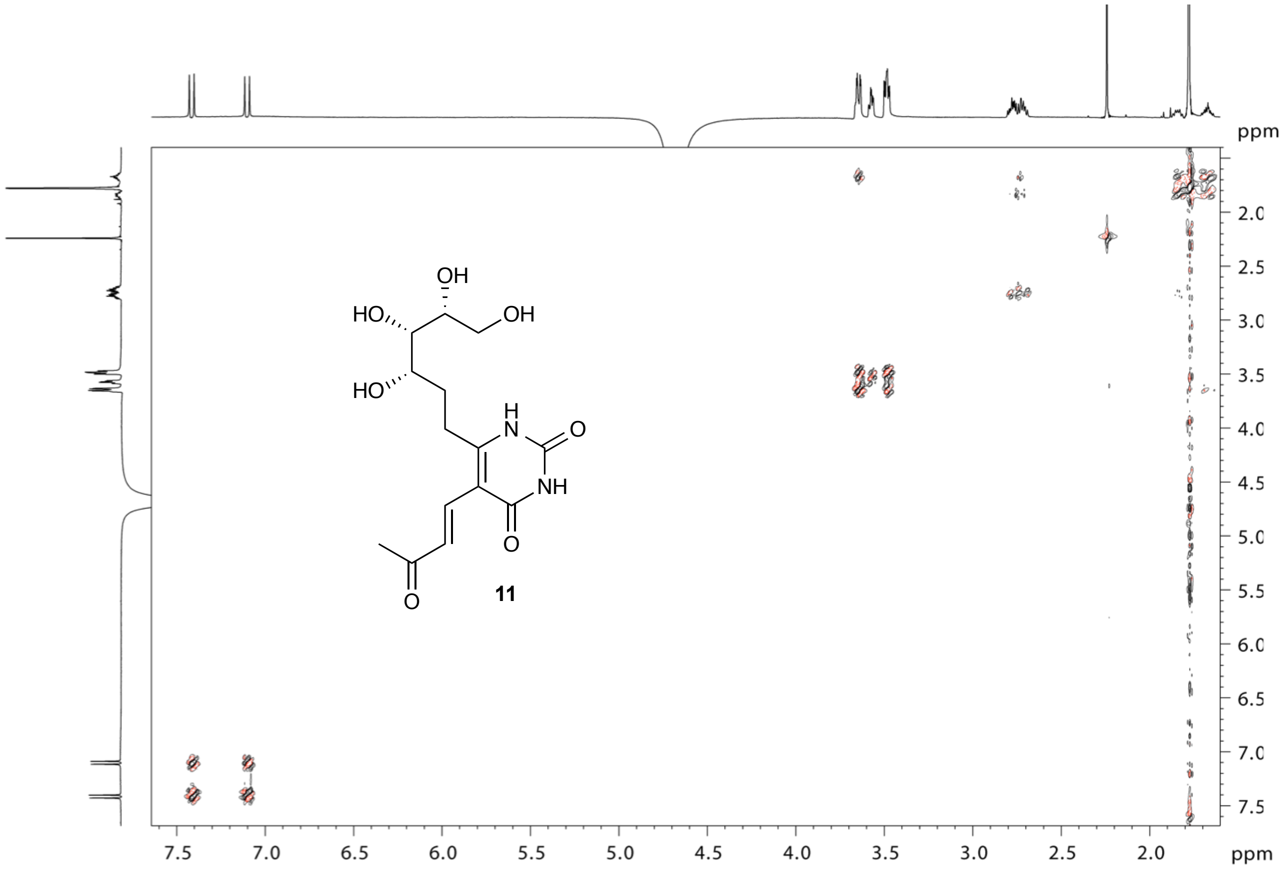

b

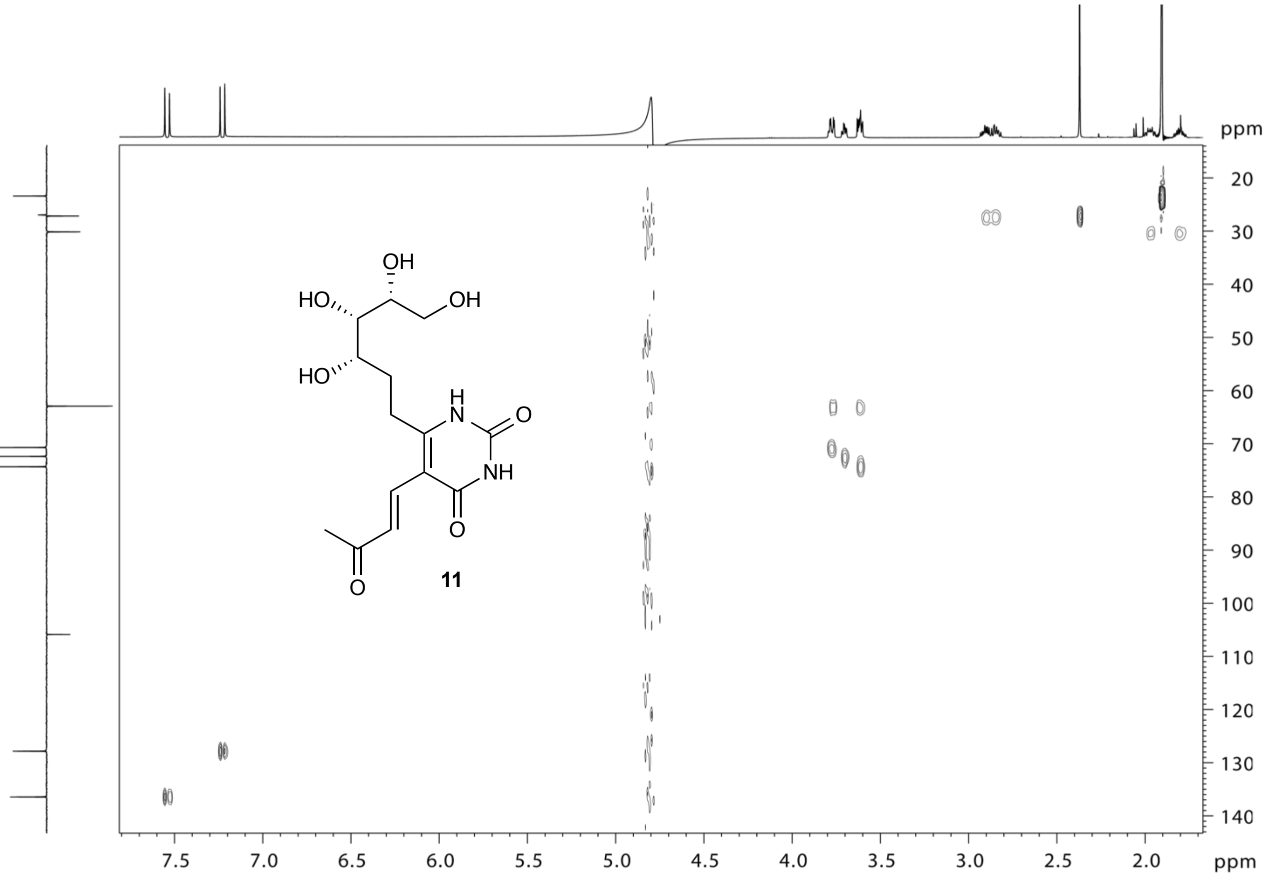

Supplementary Figure 36. (a) COSY and (b) HSQC NMR spectrum of 11 in 10% D_2O in H_2O.

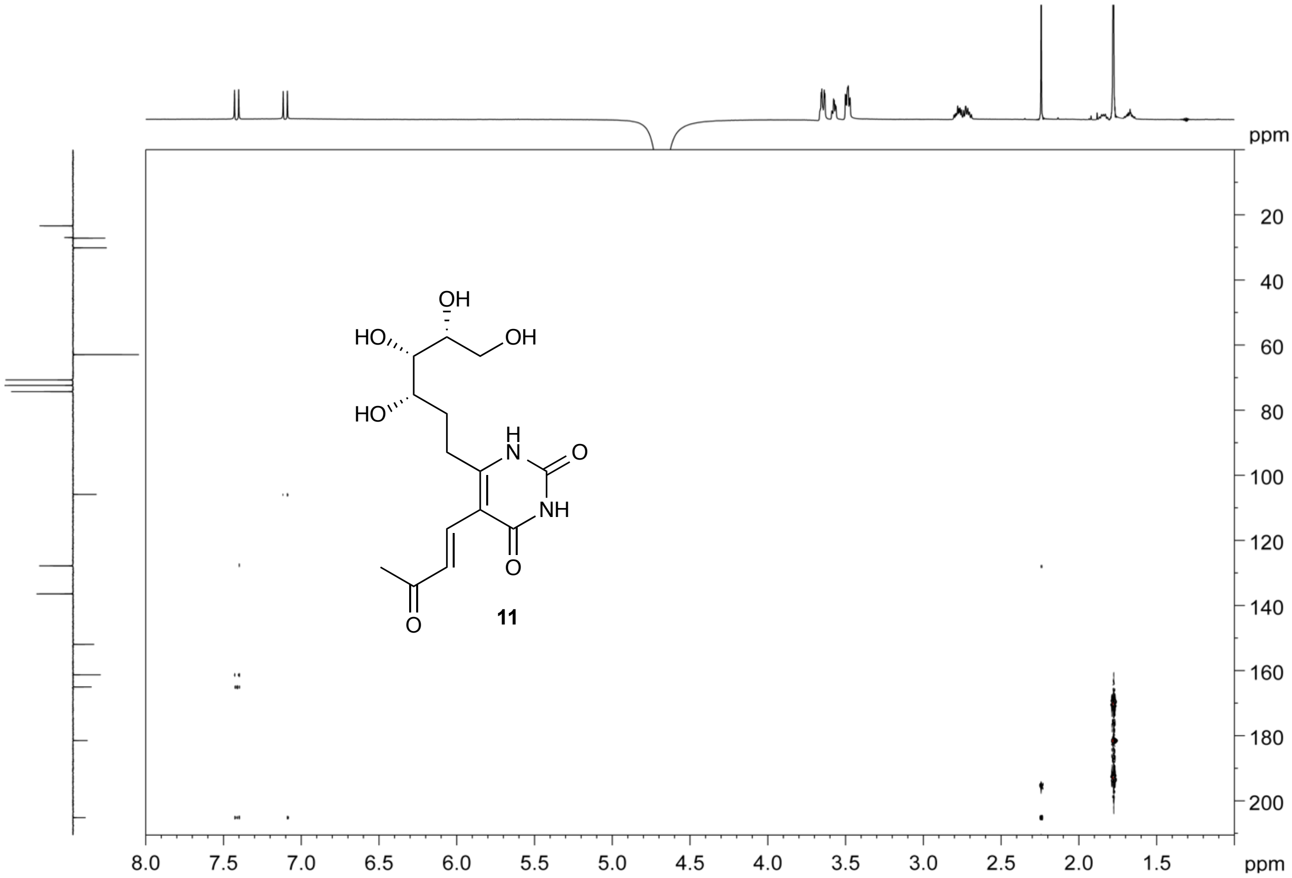

Supplementary Figure 37. HMBC NMR spectrum of 11 in 10% D_2O in H_2O.

a

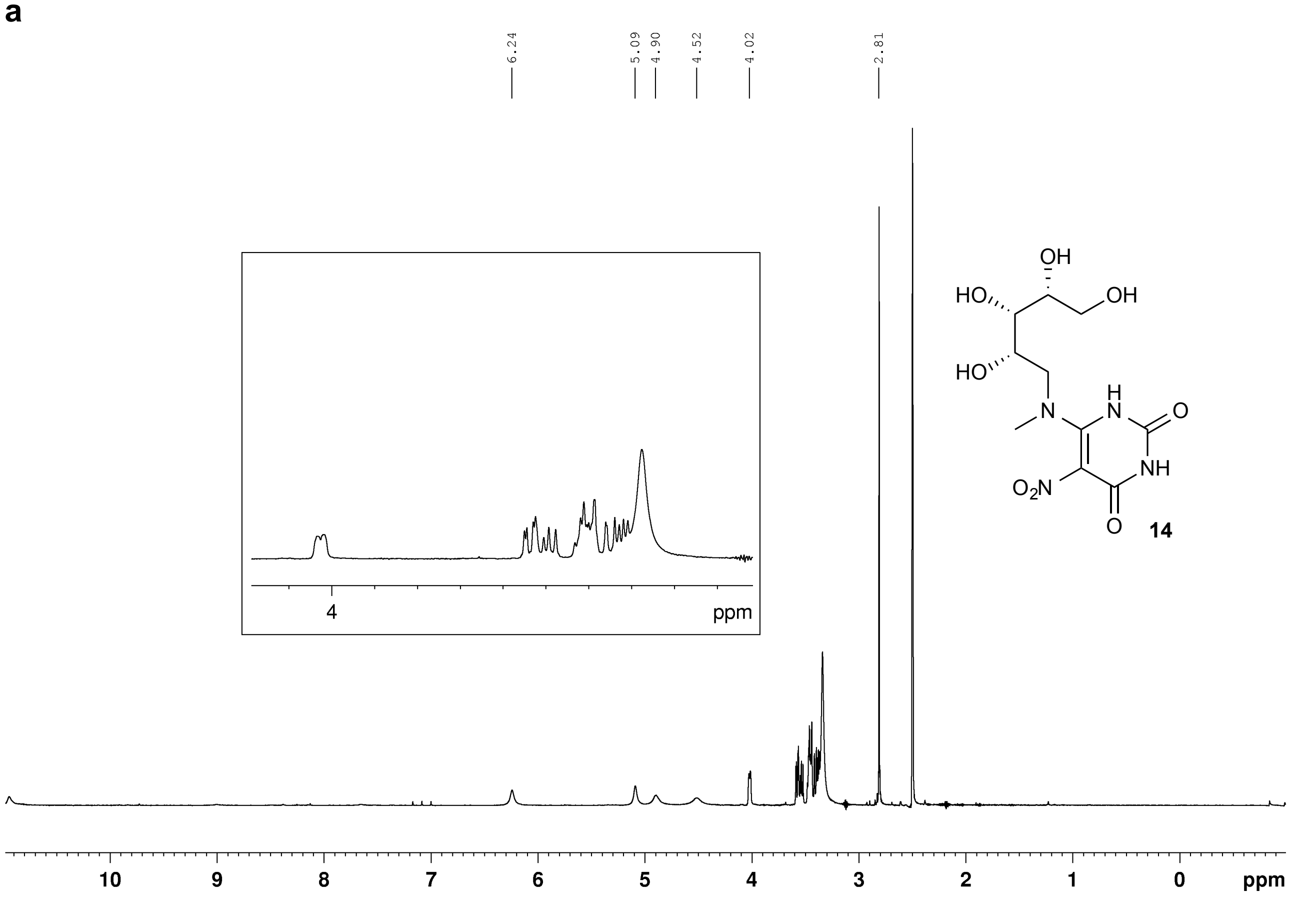

b

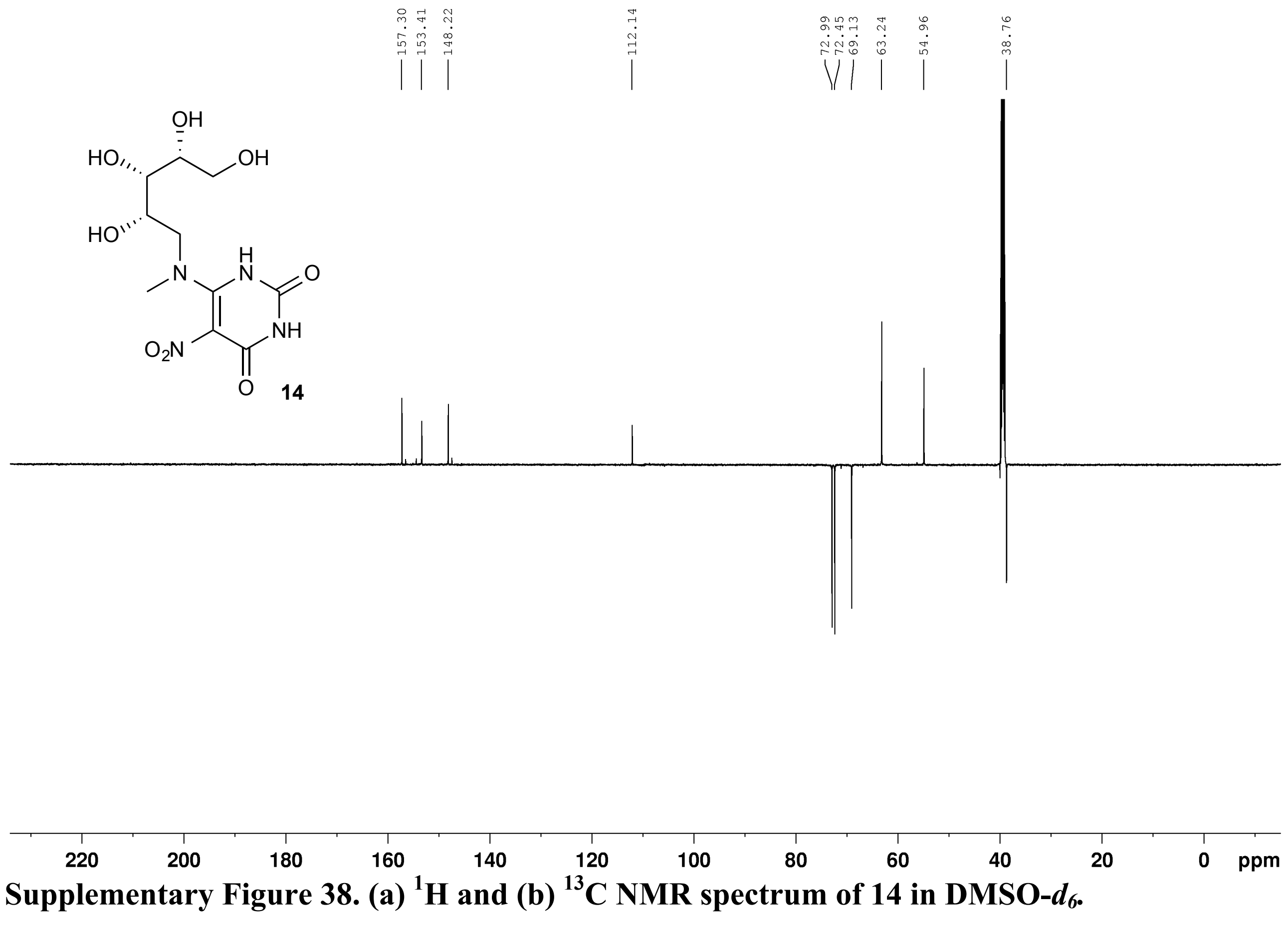

Supplementary Figure 38. (a) ^{1}H and (b) ^{13}C NMR spectrum of 14 in DMSO-d_6.

a

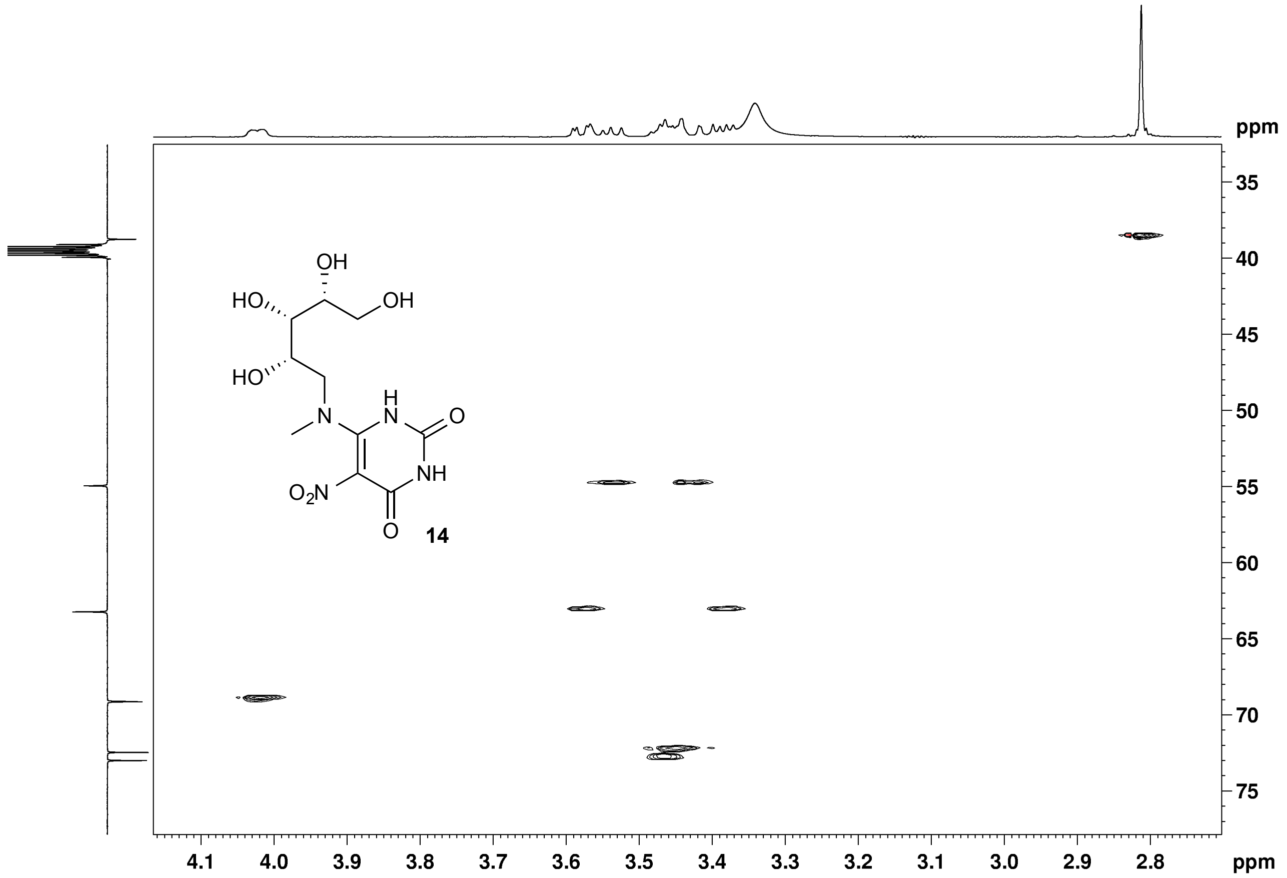

b

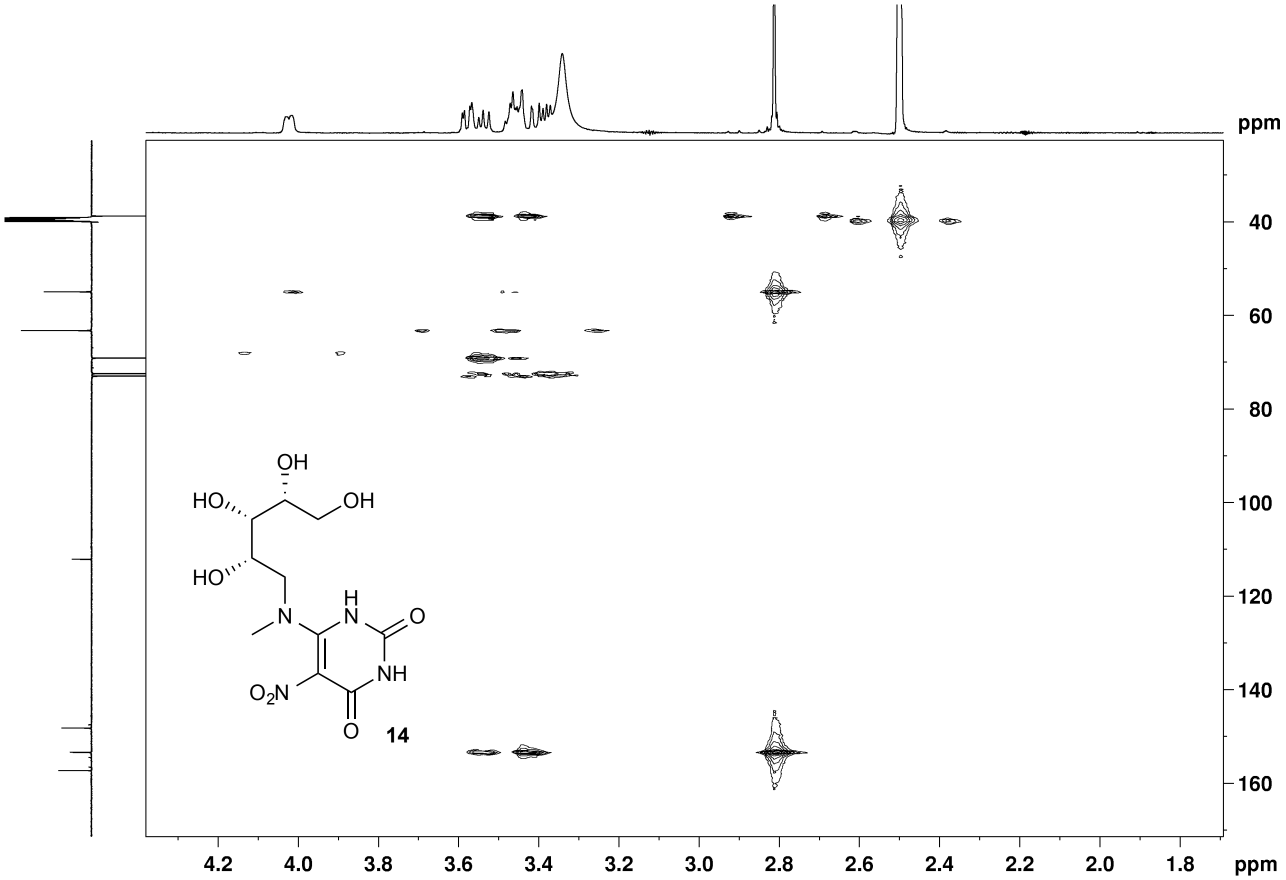

Supplementary Figure 39. (a) COSY and (b) HSQC NMR spectrum of 14 in DMSO-d_6.

a

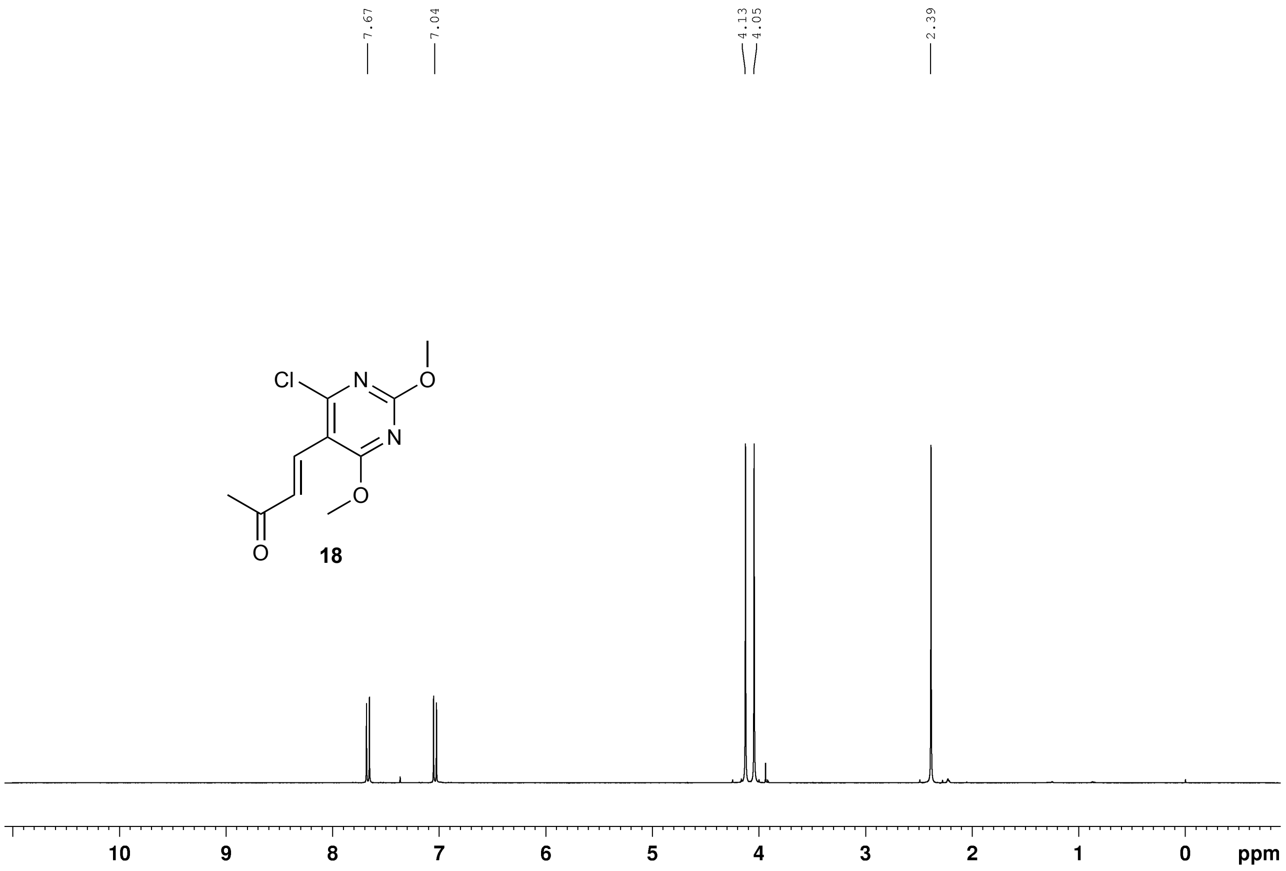

b

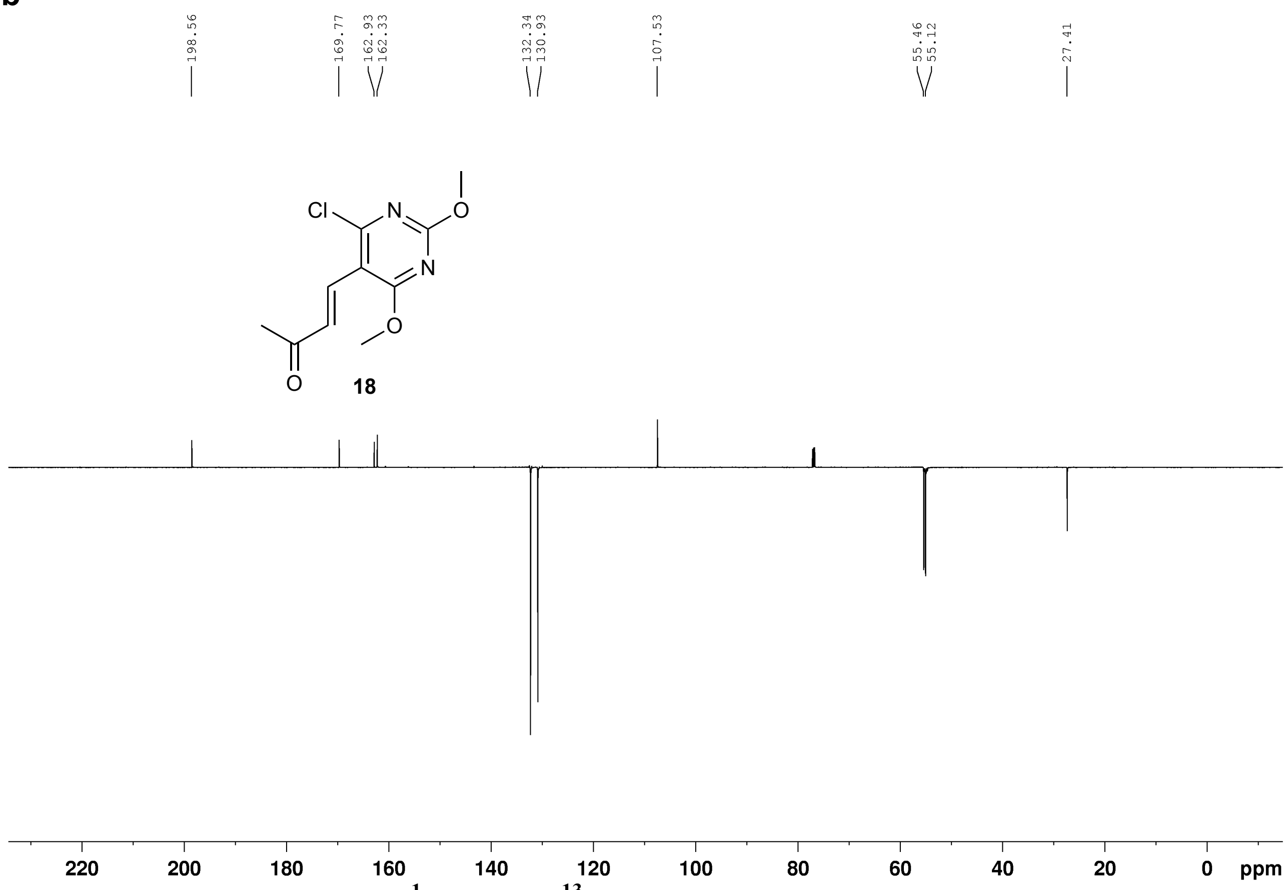

Supplementary Figure 40. (a) ^{1}H and (b) ^{13}C NMR spectrum of 18 in $CDCl_3$.

a

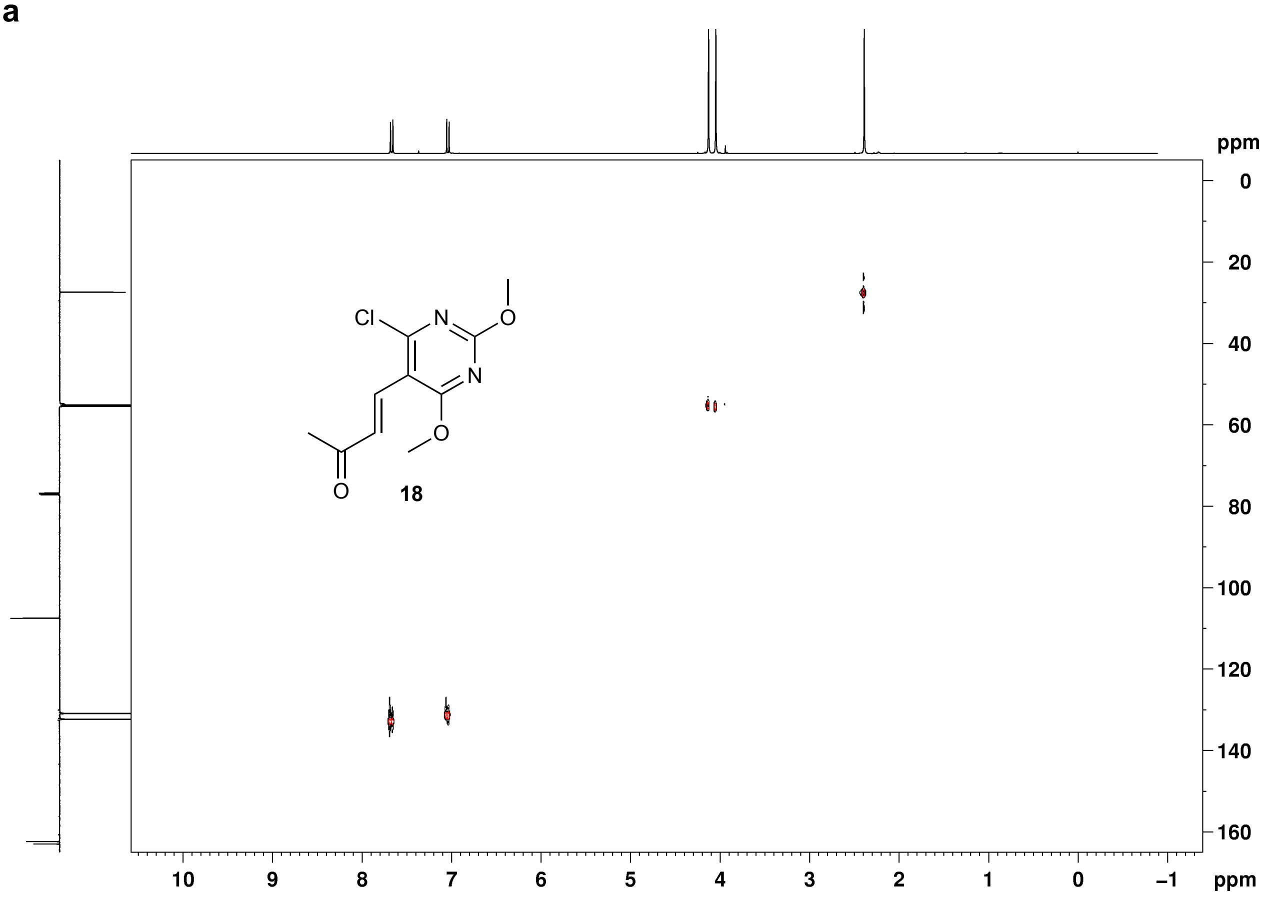

b

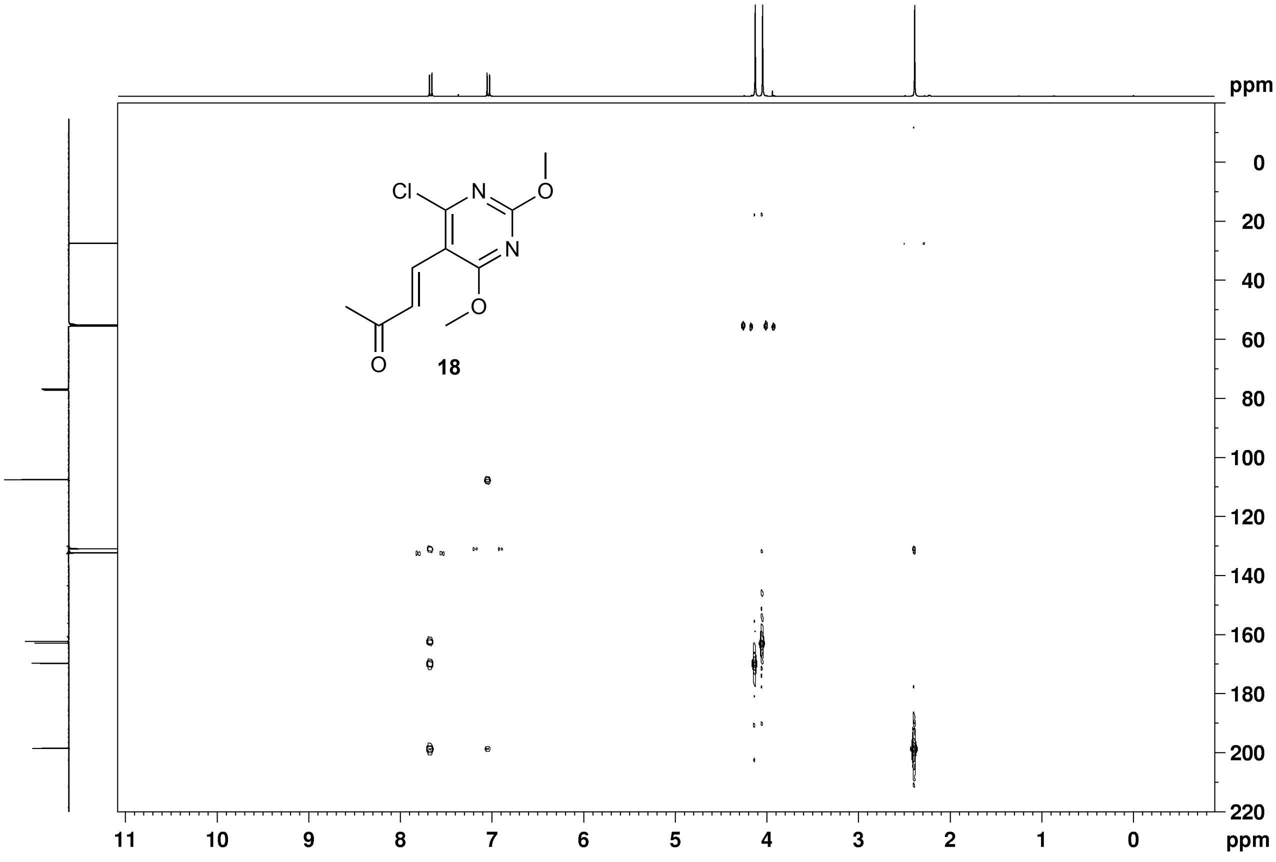

Supplementary Figure 41. (a) HSQC and (b) HMBC NMR spectrum of 18 in $CDCl_3$.

a

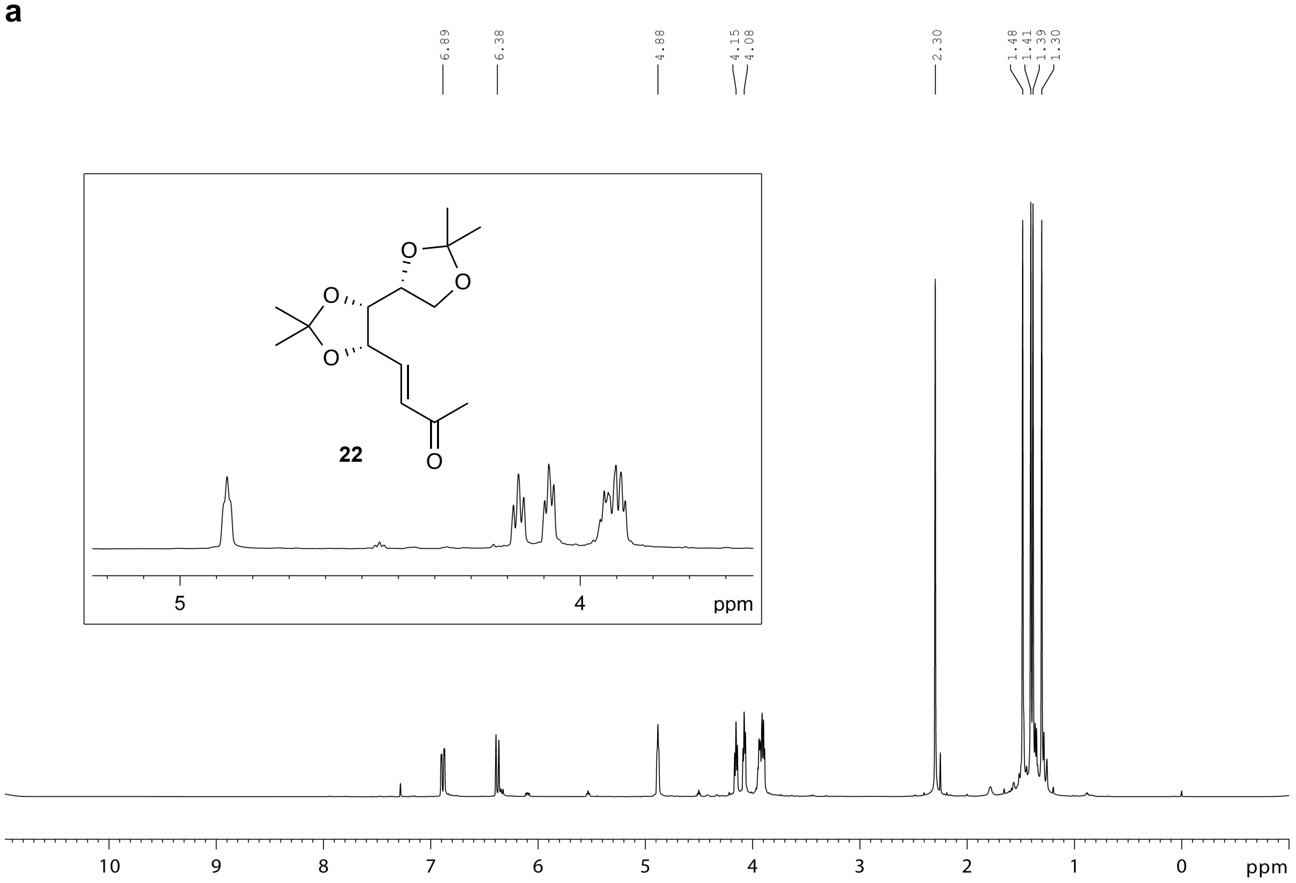

b

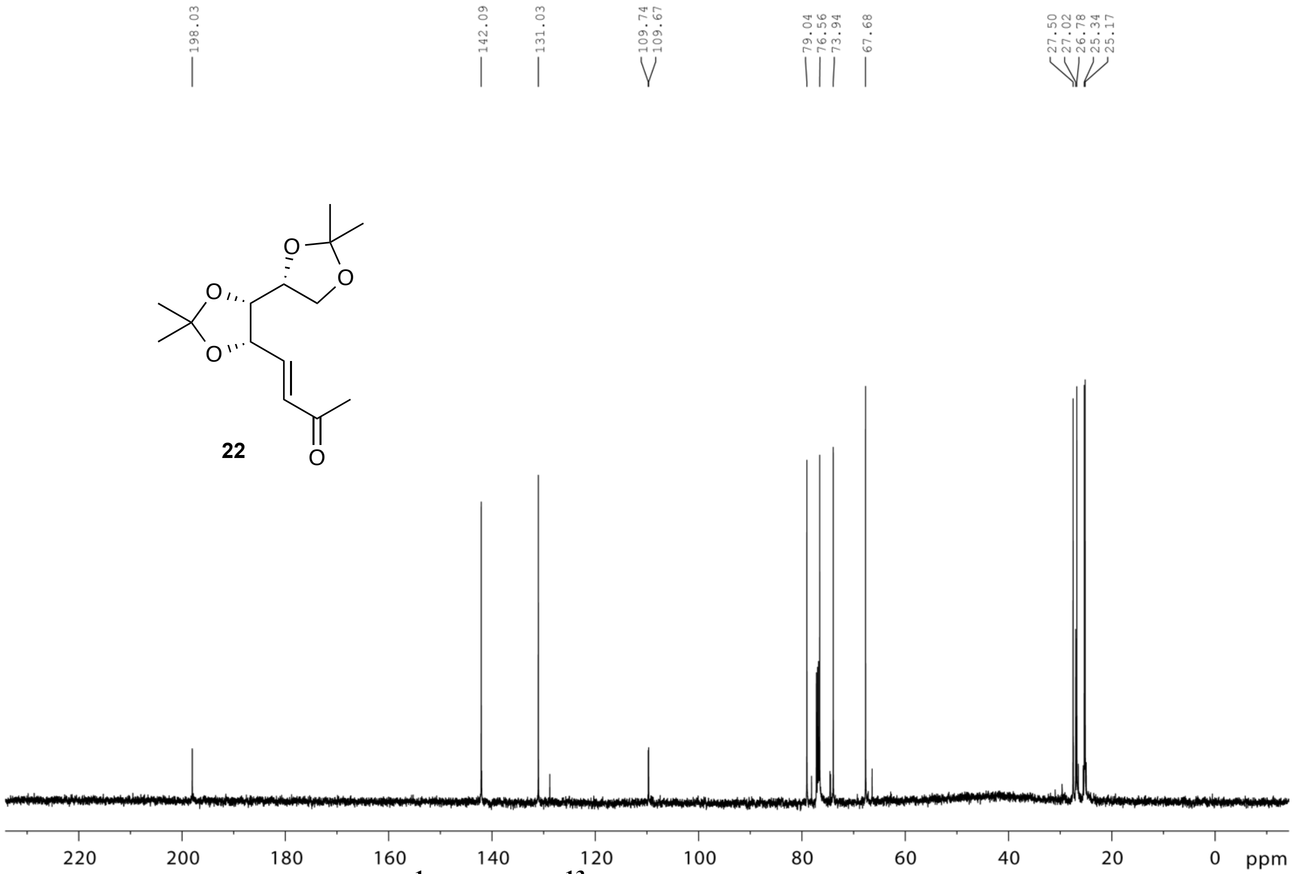

Supplementary Figure 42. (a) ^{1}H and (b) ^{13}C NMR spectrum of 22 in $CDCl_3$.

a

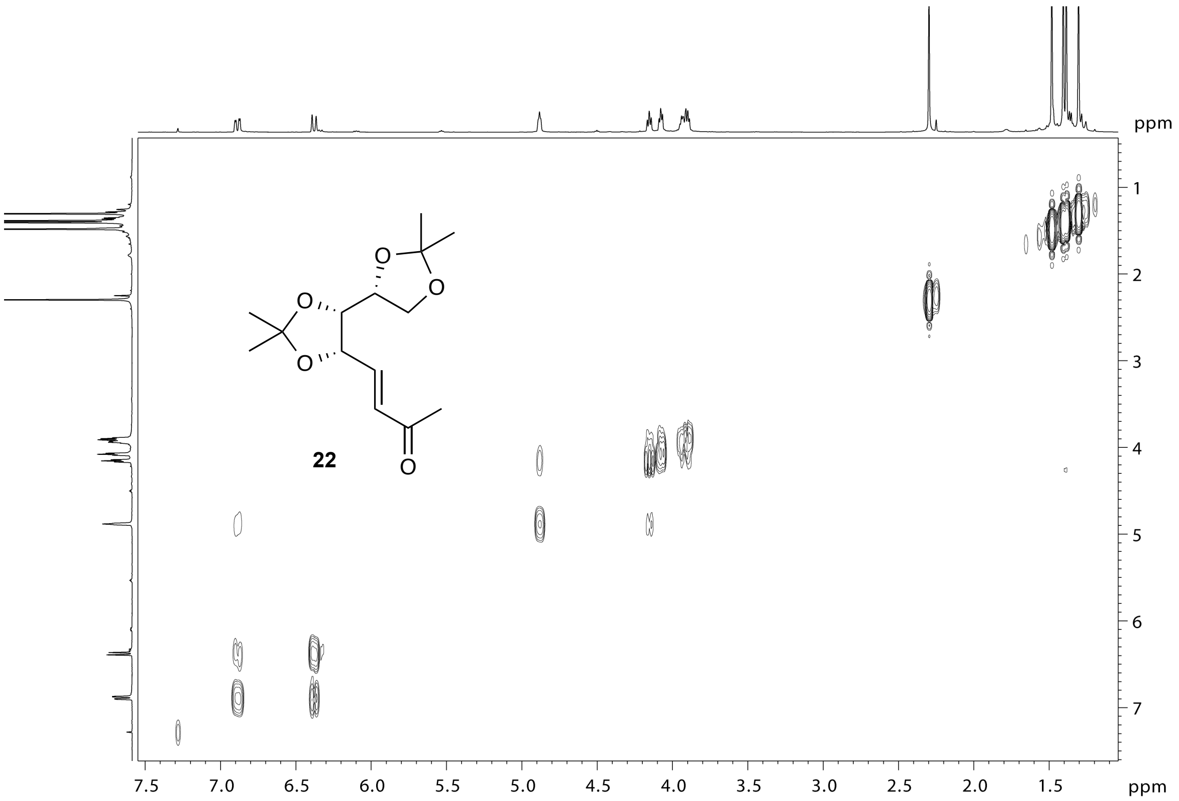

b

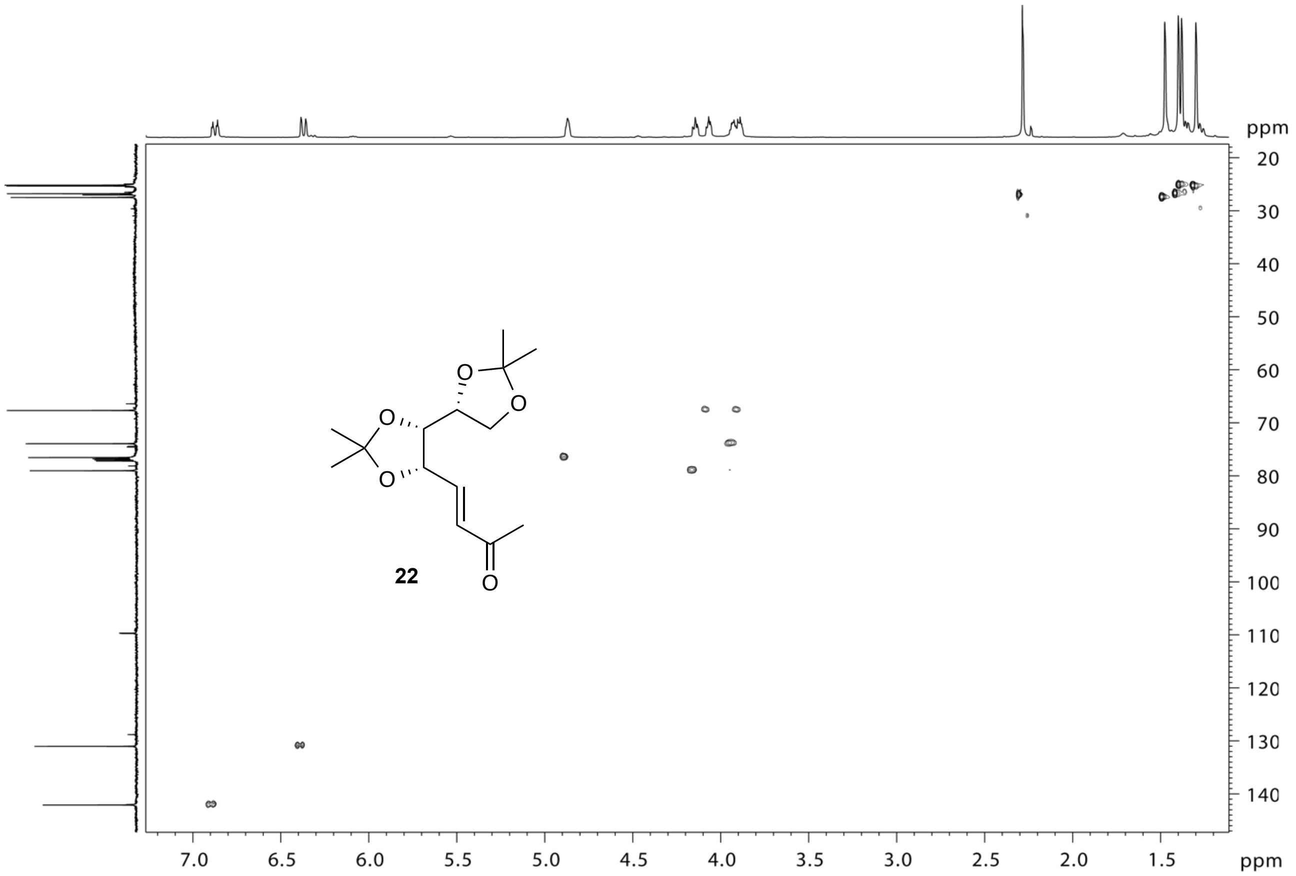

Supplementary Figure 43. (a) COSY and (b) HSQC NMR spectrum of 22 in $CDCl_3$.

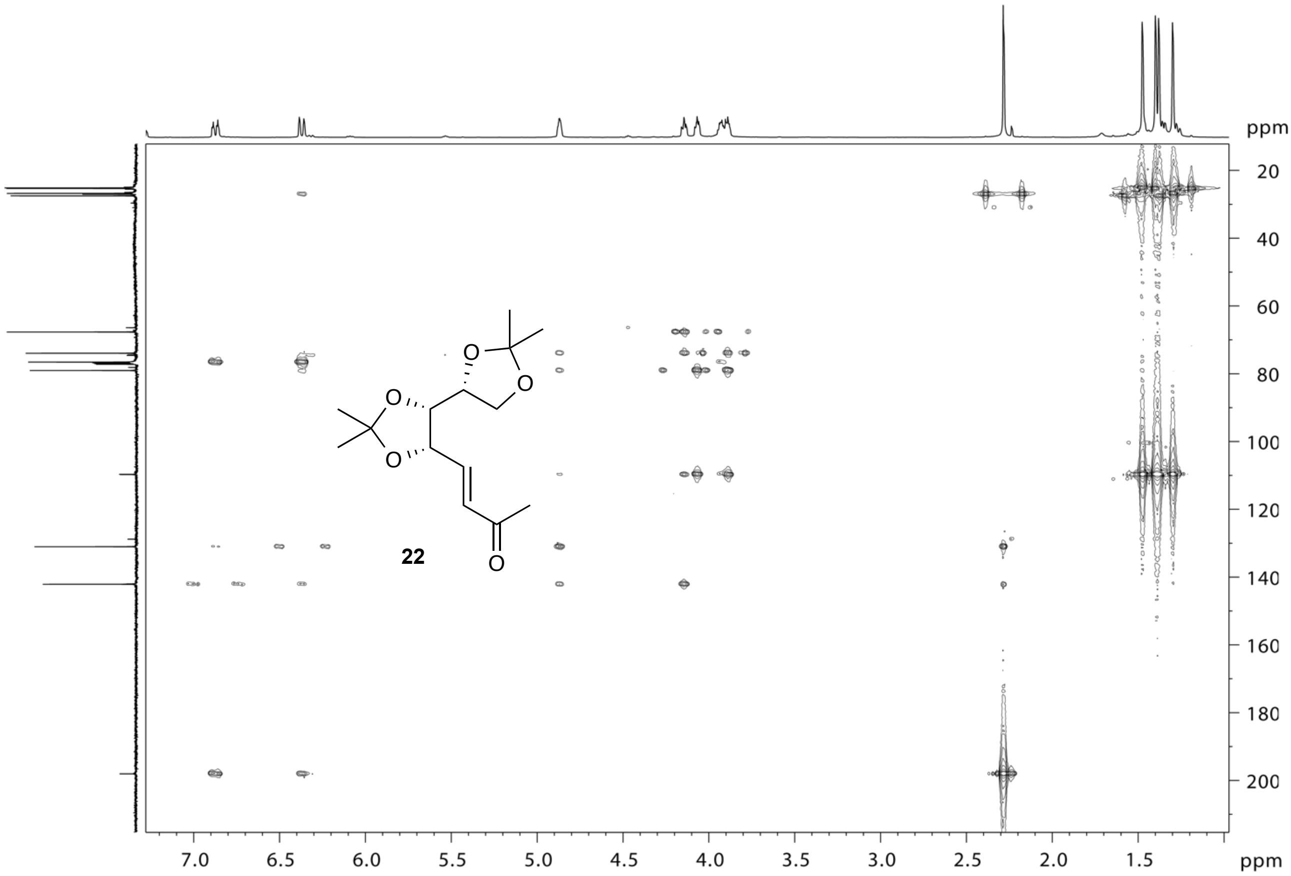

Supplementary Figure 44. HMBC NMR spectrum of 22 in $CDCl_3$.

a

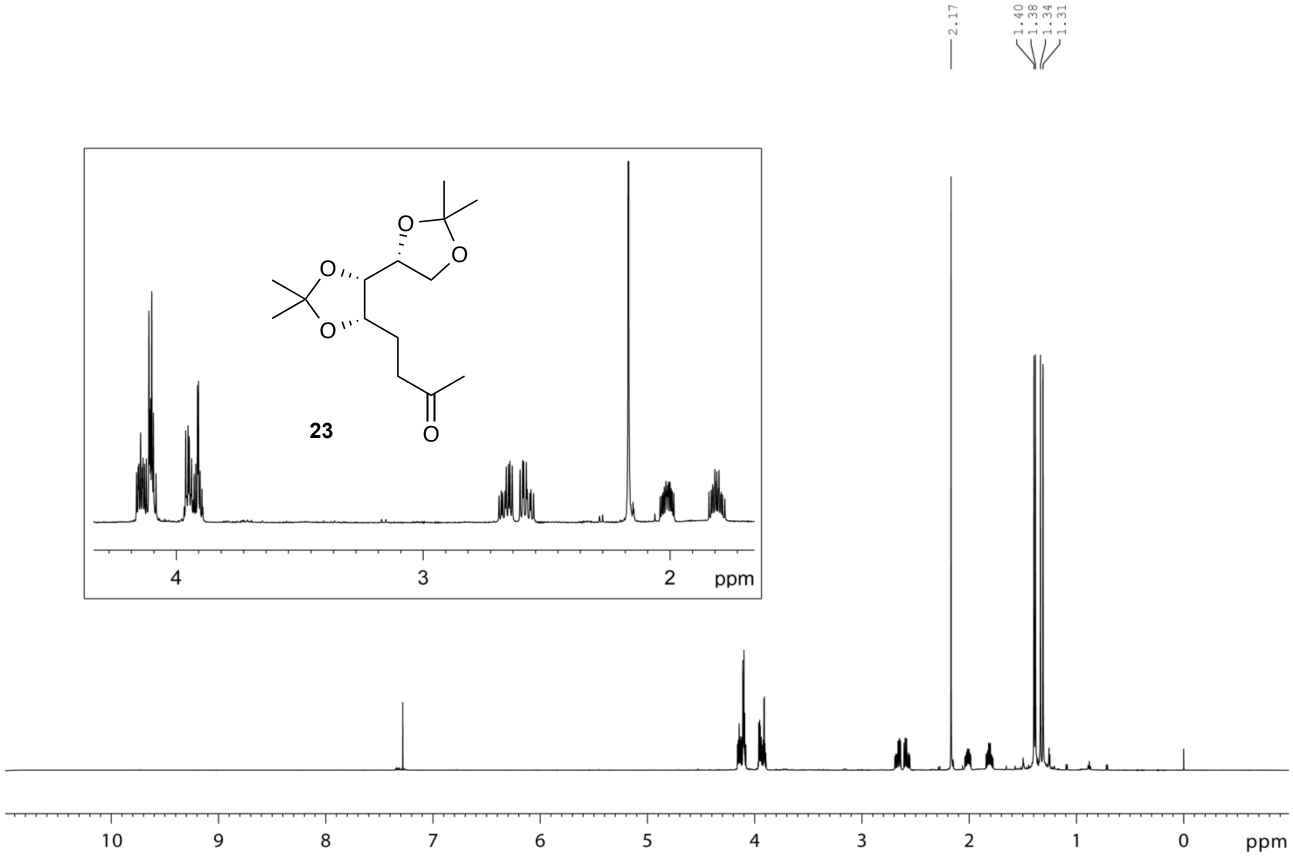

b

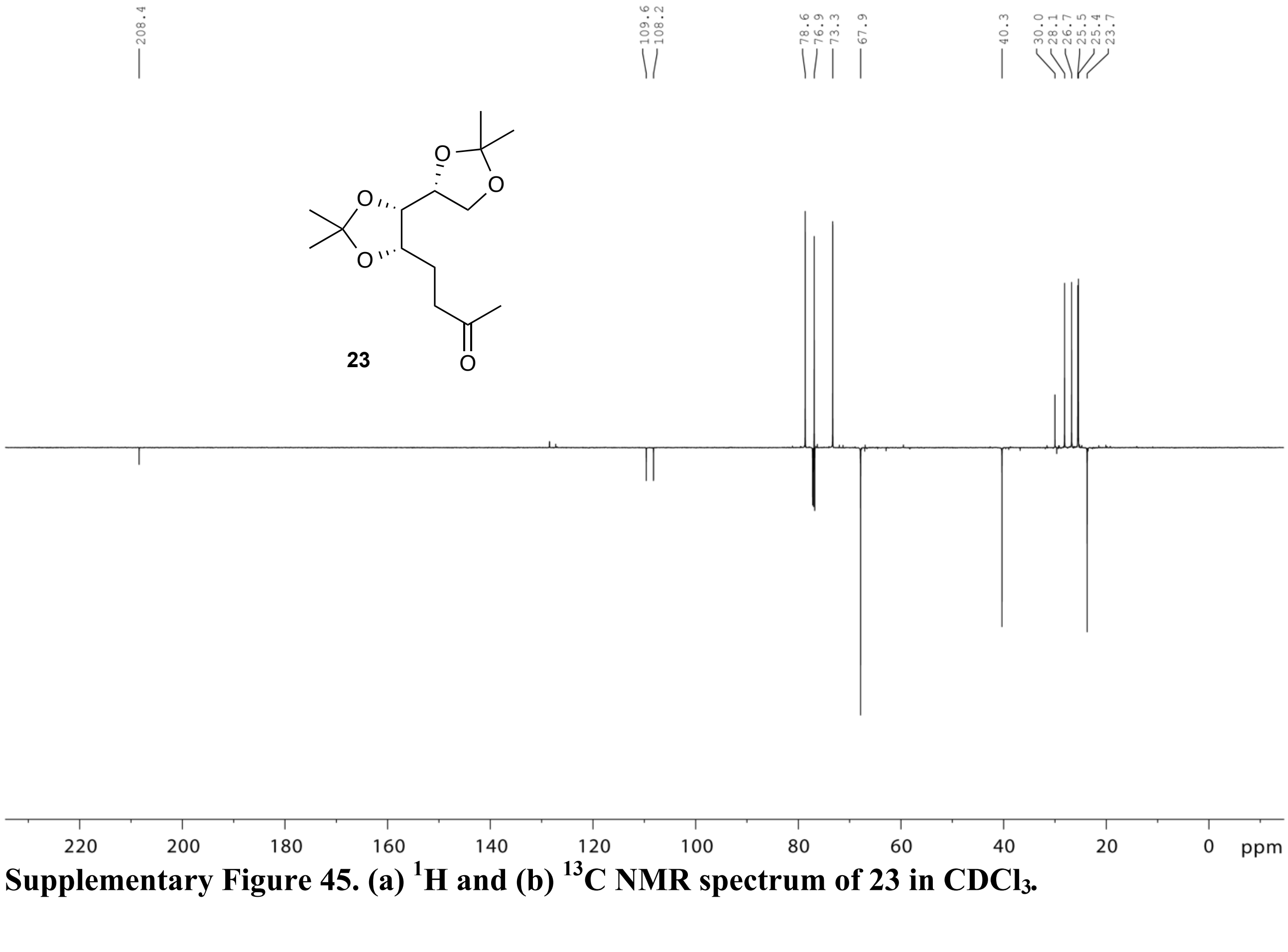

Supplementary Figure 45. (a) ^{1}H and (b) ^{13}C NMR spectrum of 23 in $CDCl_3$.

a

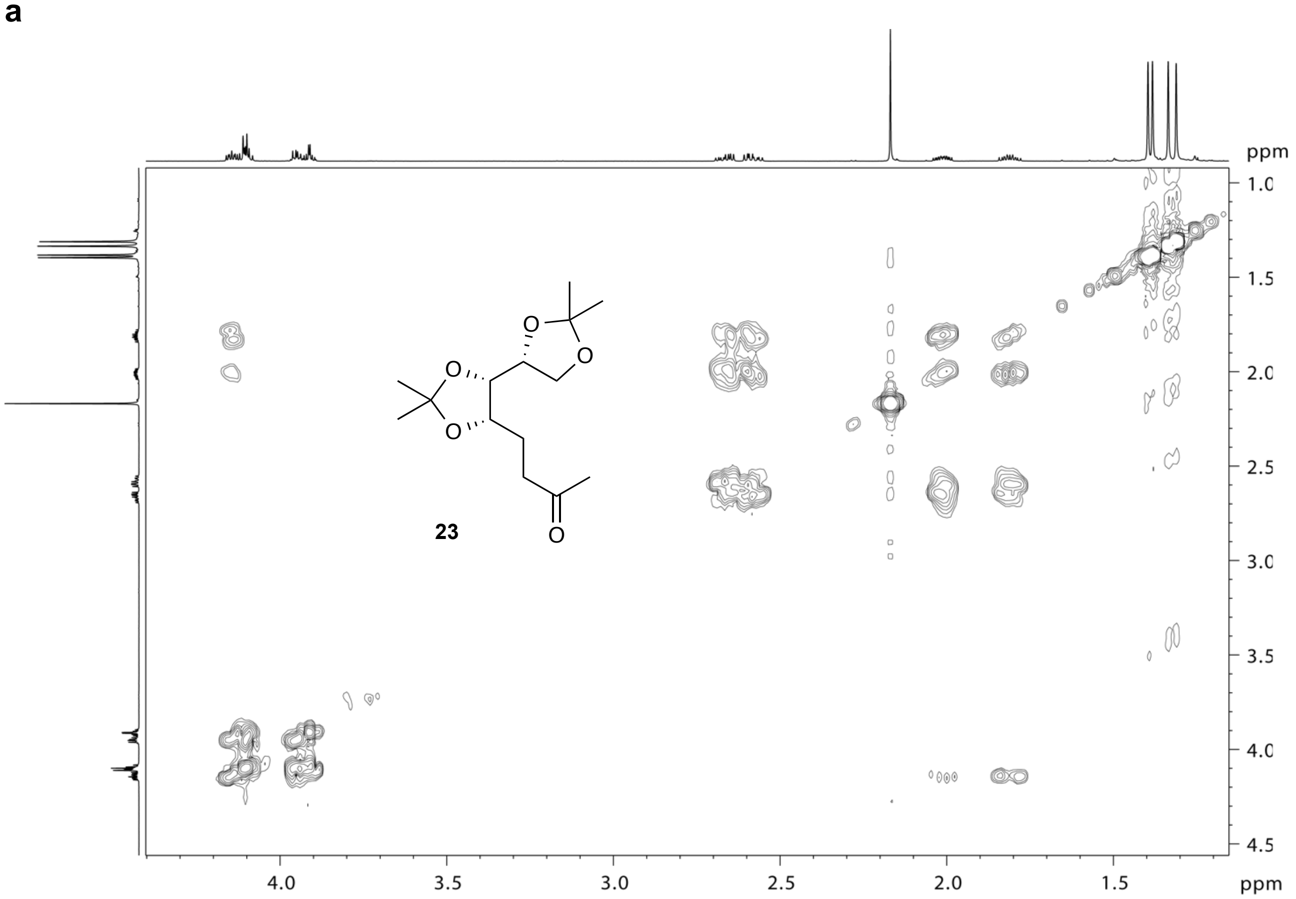

b

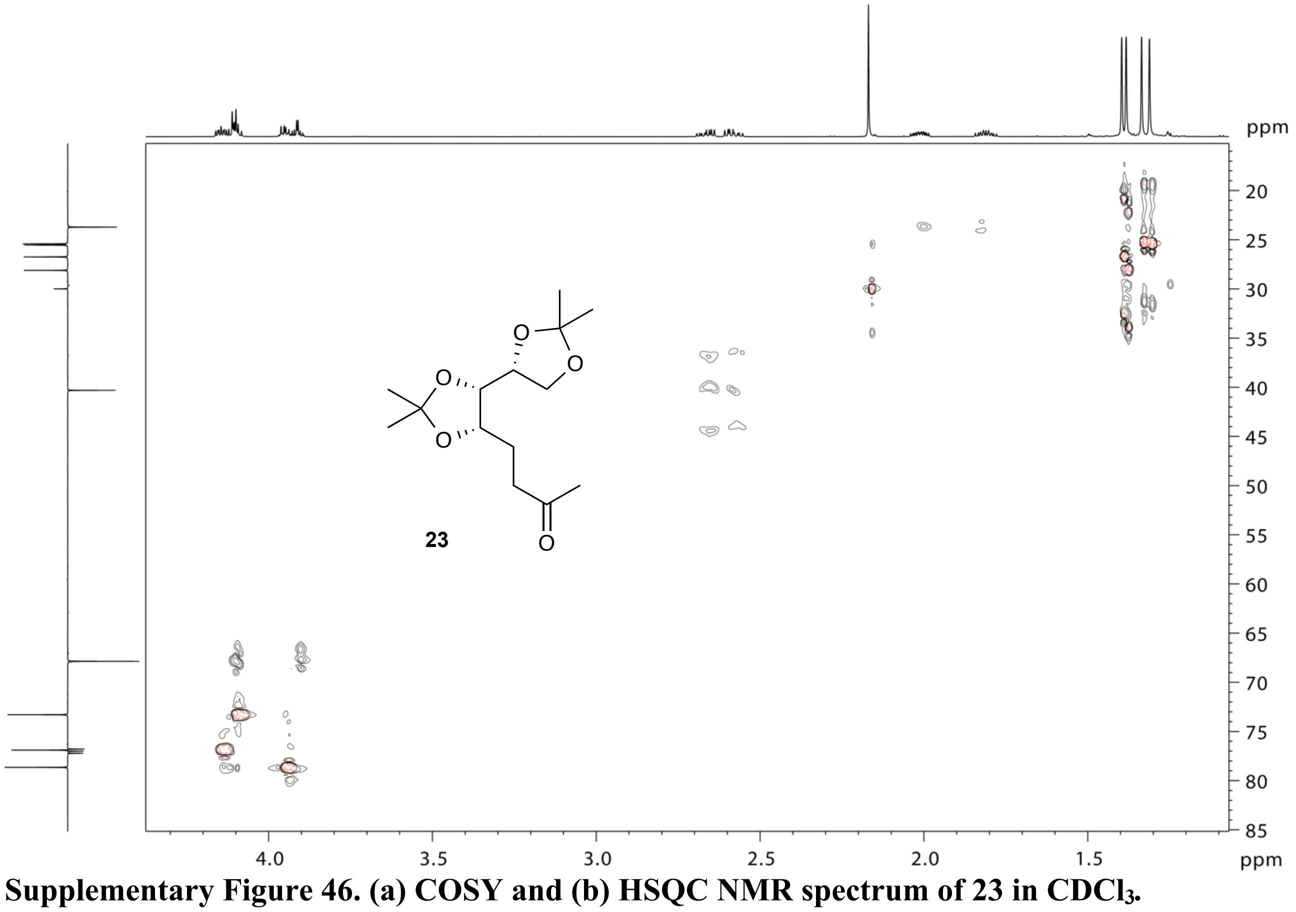

Supplementary Figure 46. (a) COSY and (b) HSQC NMR spectrum of 23 in $CDCl_3$.

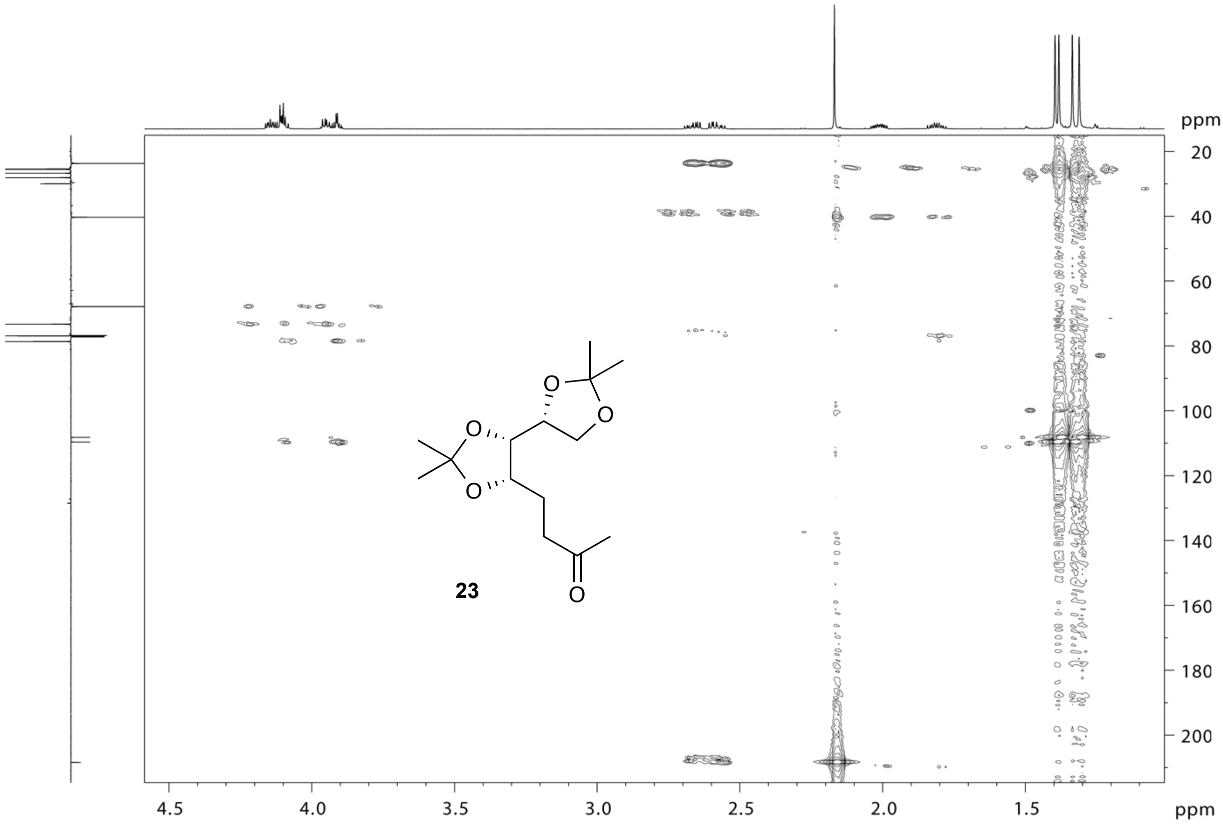

Supplementary Figure 47. HMBC NMR spectrum of 23 in $CDCl_3$.

a

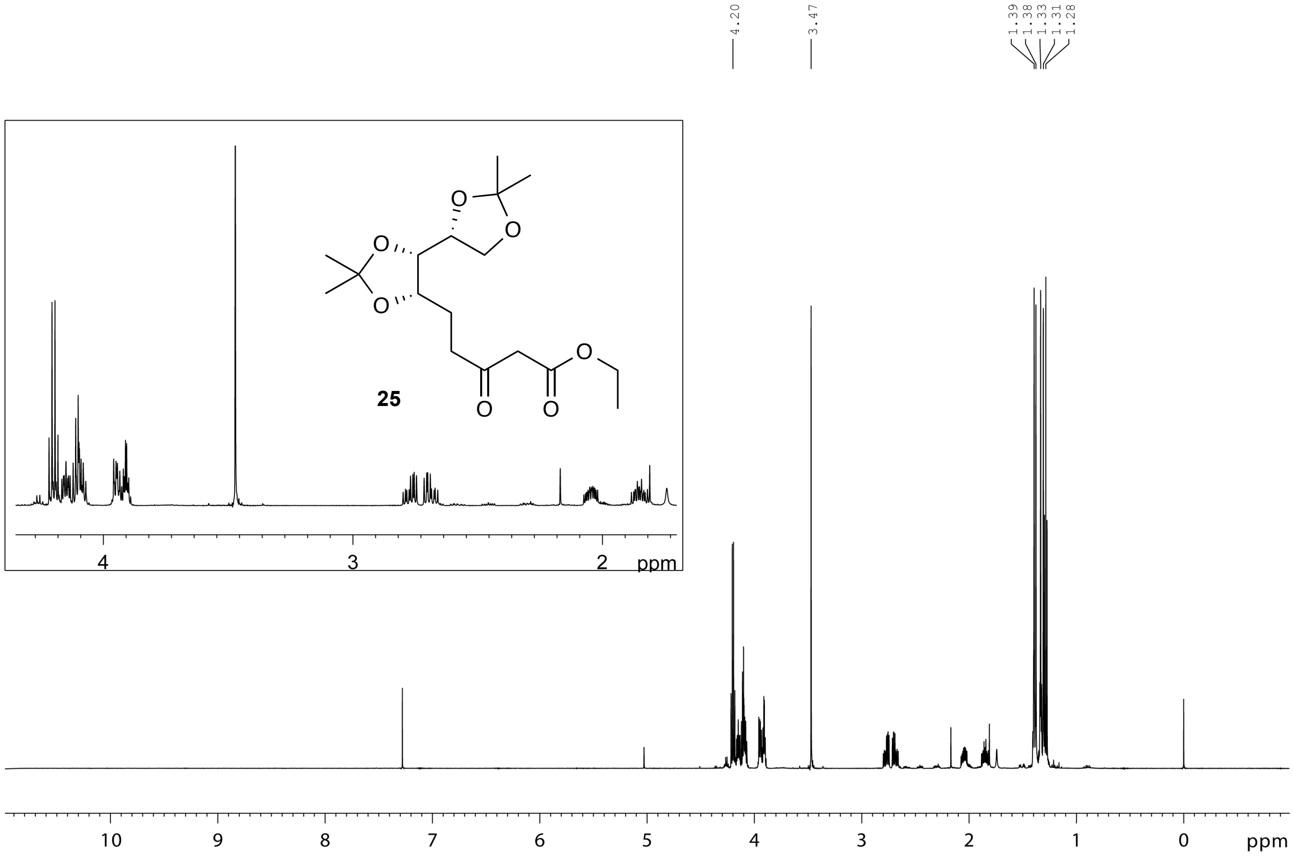

b

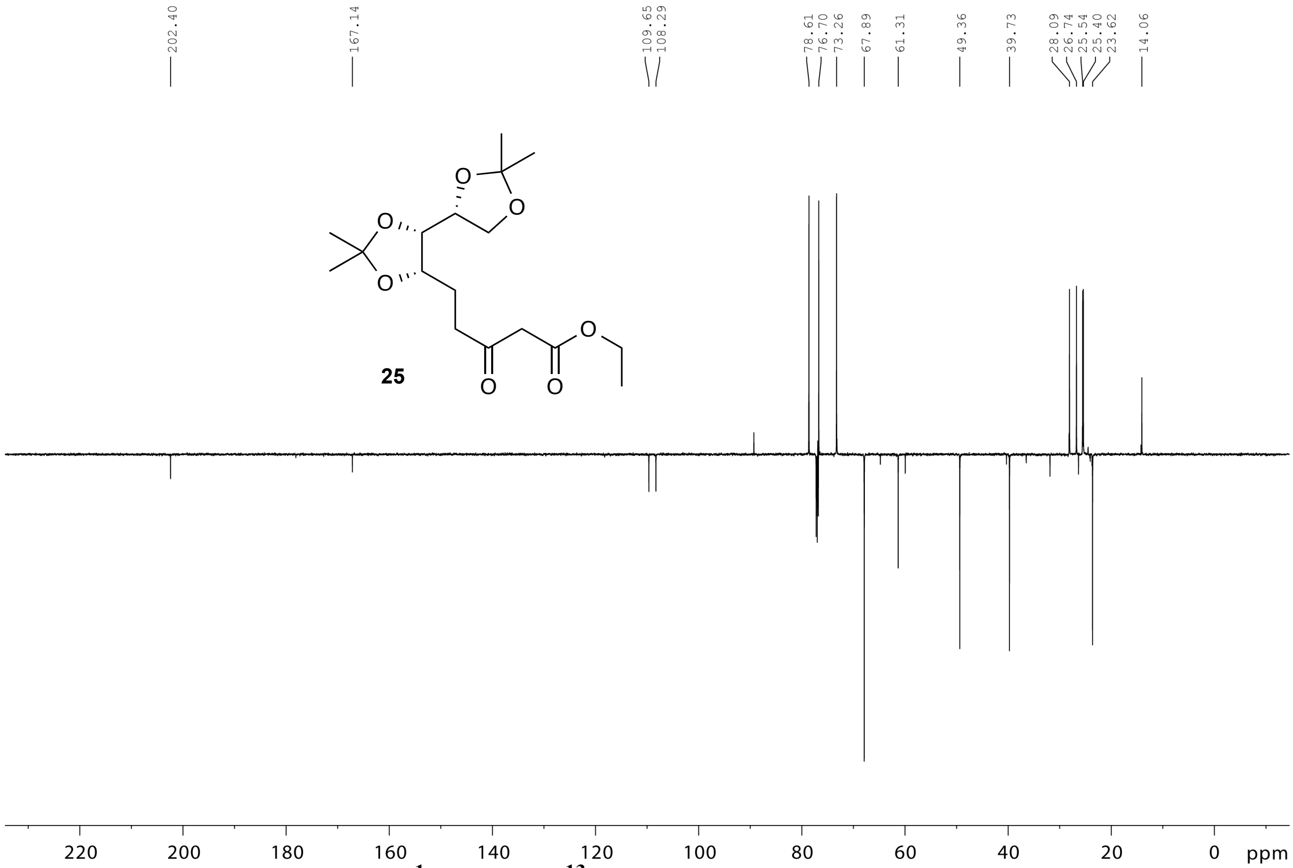

Supplementary Figure 48. (a) ^{1}H and (b) ^{13}C NMR spectrum of 25 in $CDCl_3$.

a

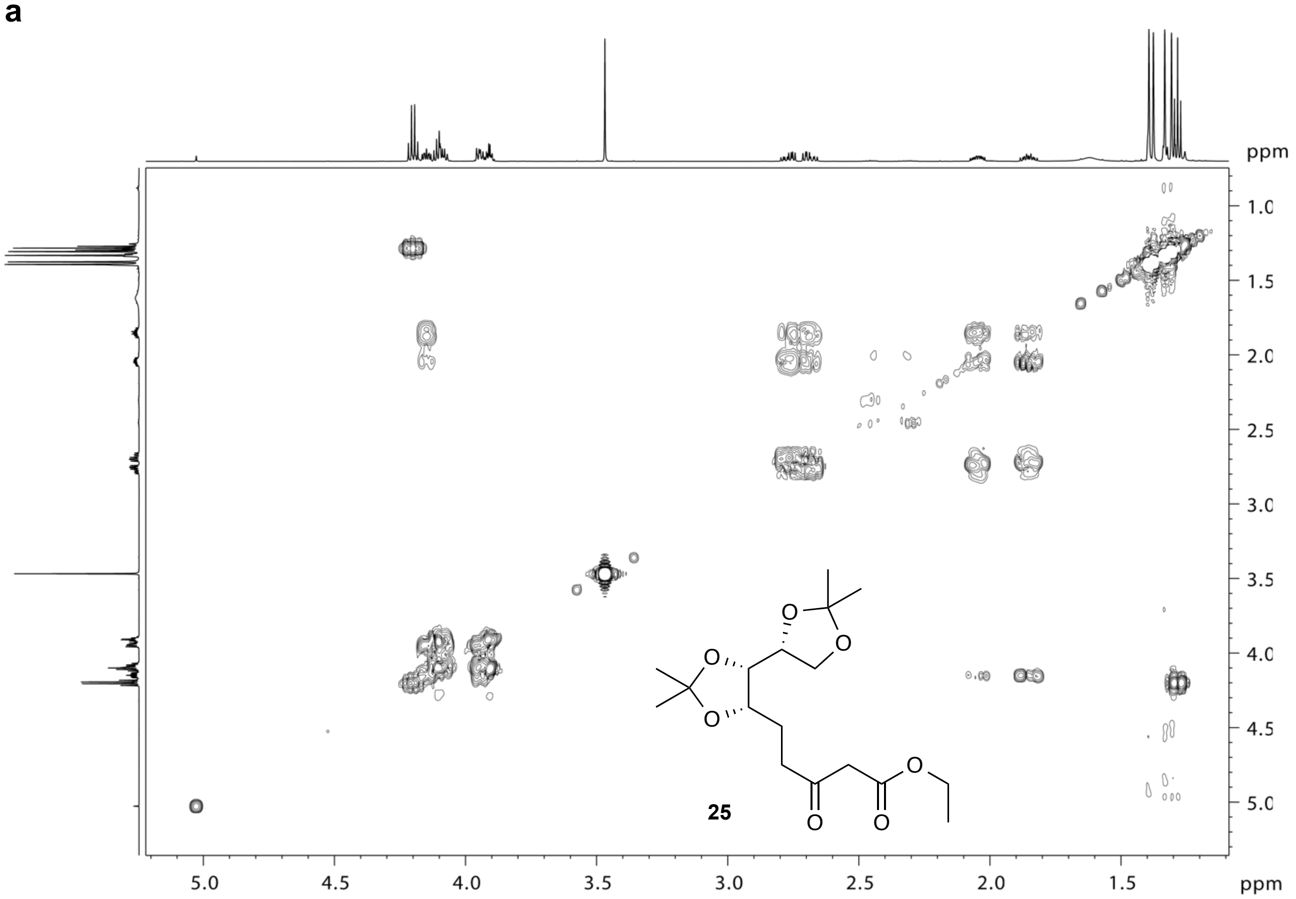

b

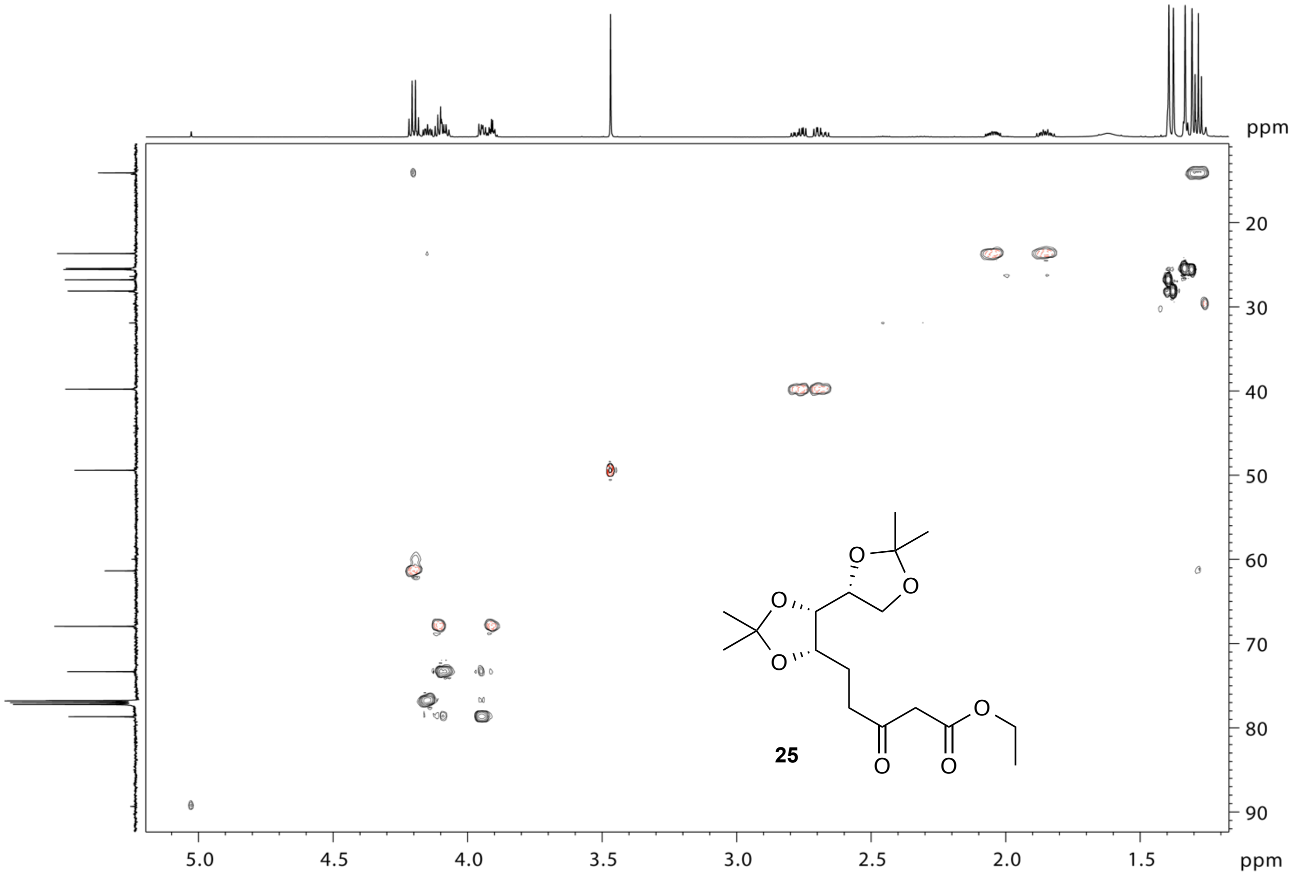

Supplementary Figure 49. (a) COSY and (b) HSQC NMR spectrum of 25 in $CDCl_3$.

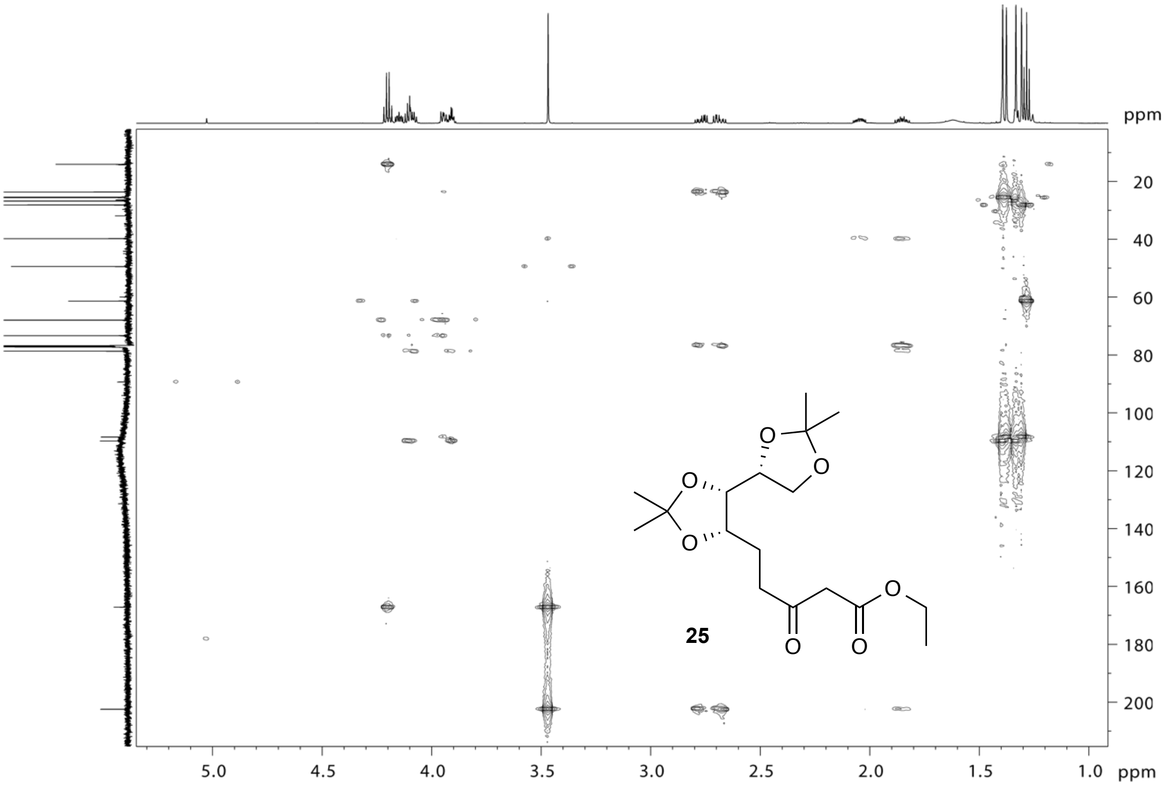

Supplementary Figure 50. HMBC NMR spectrum of 25 in $CDCl_3$.

a

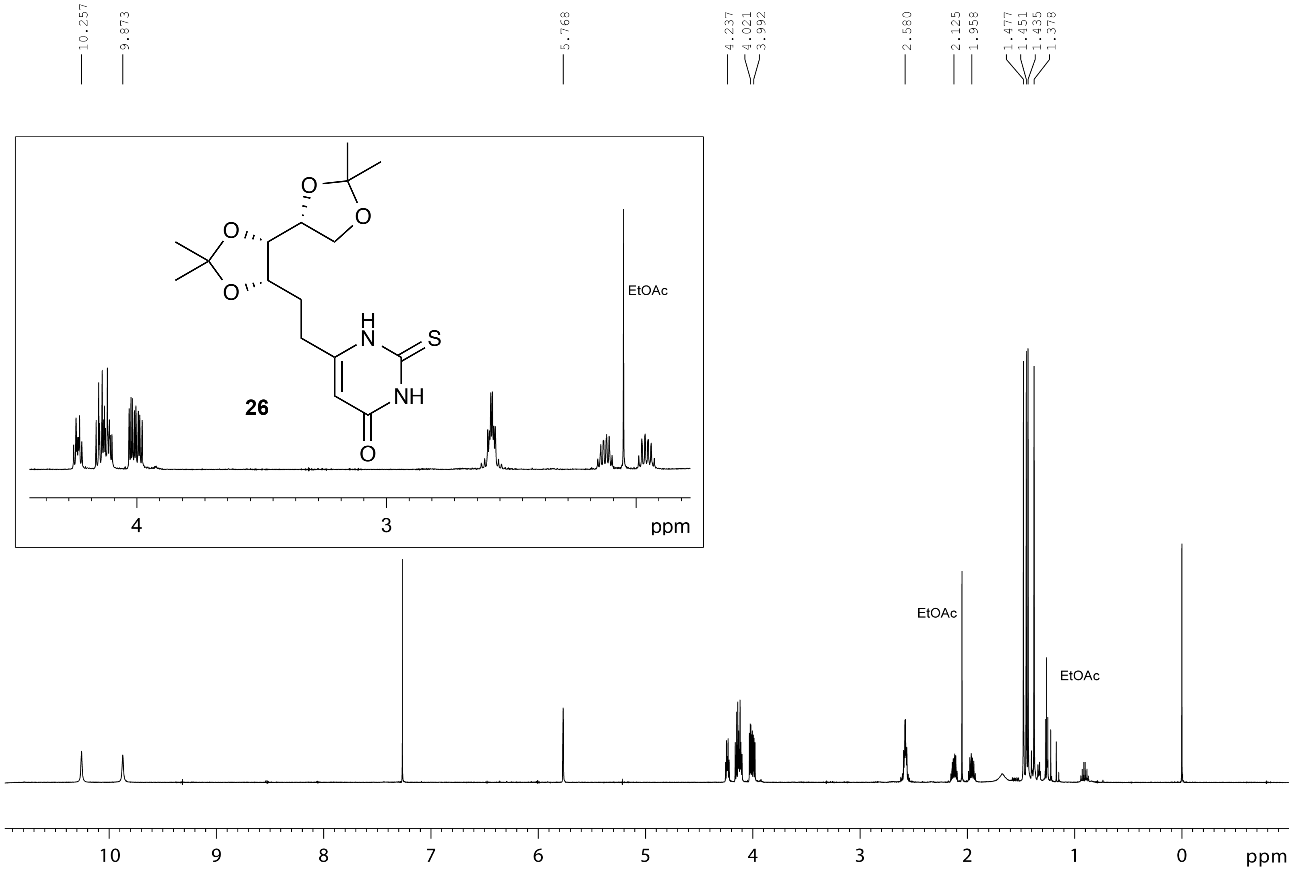

b

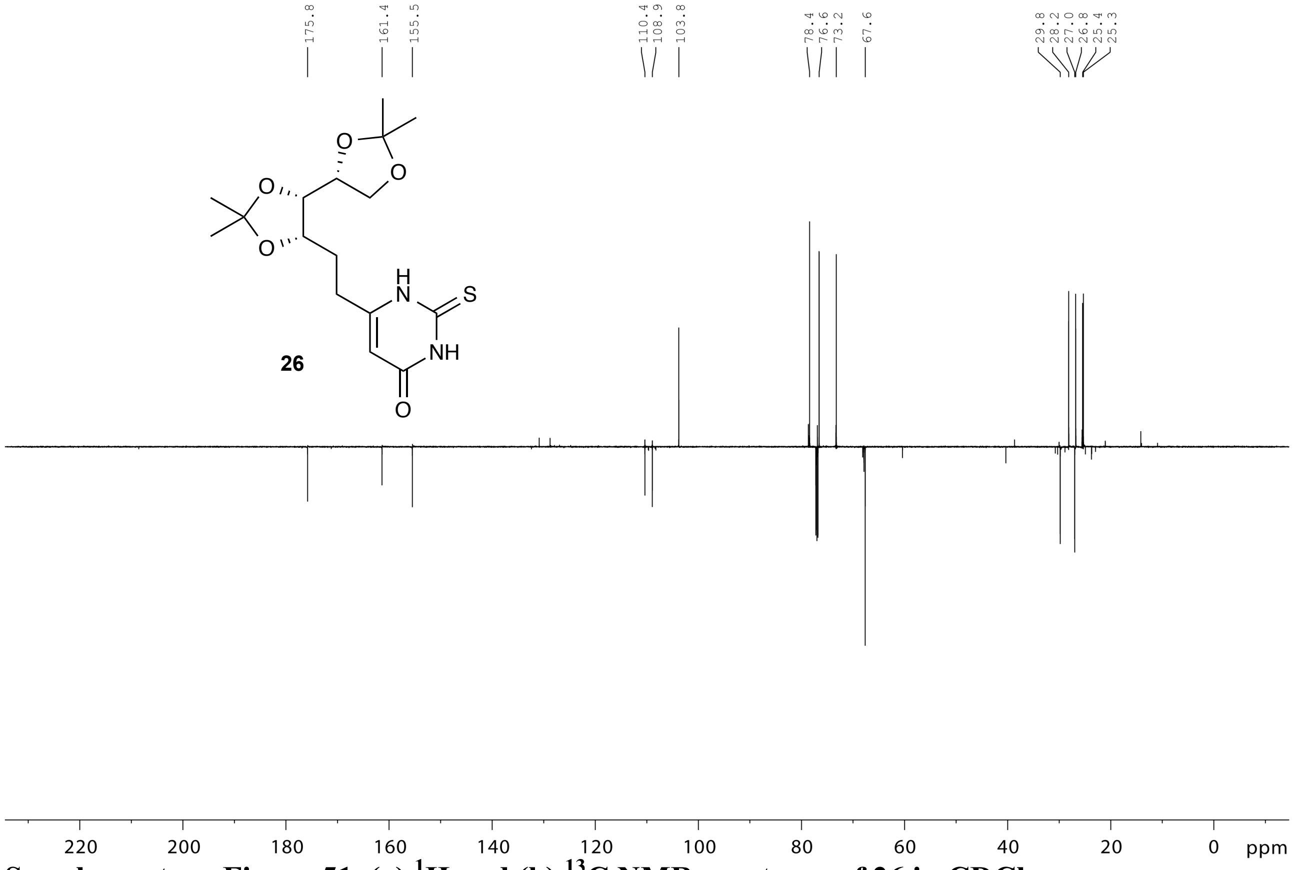

Supplementary Figure 51. (a) ^{1}H and (b) ^{13}C NMR spectrum of 26 in $CDCl_3$.

a

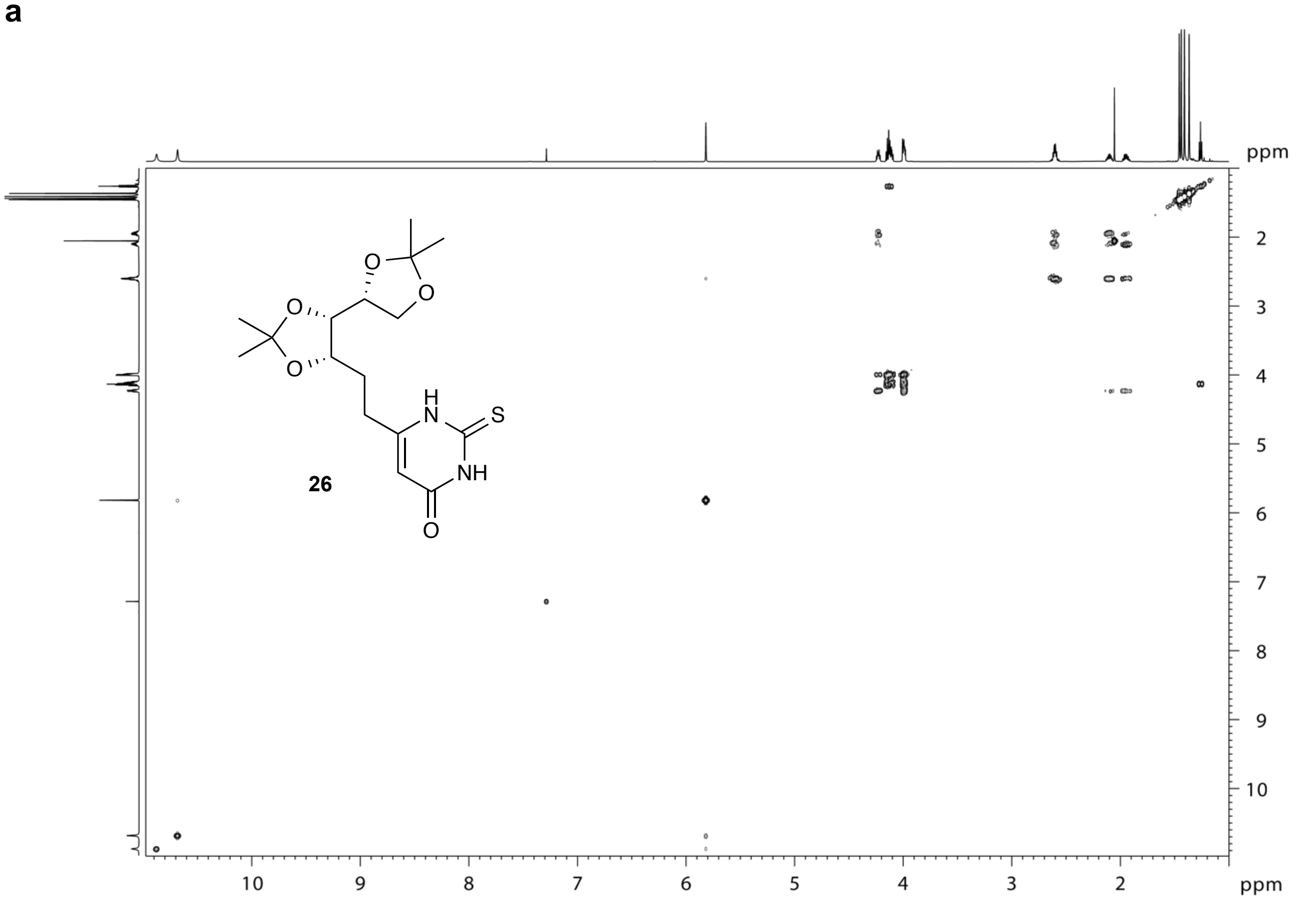

b

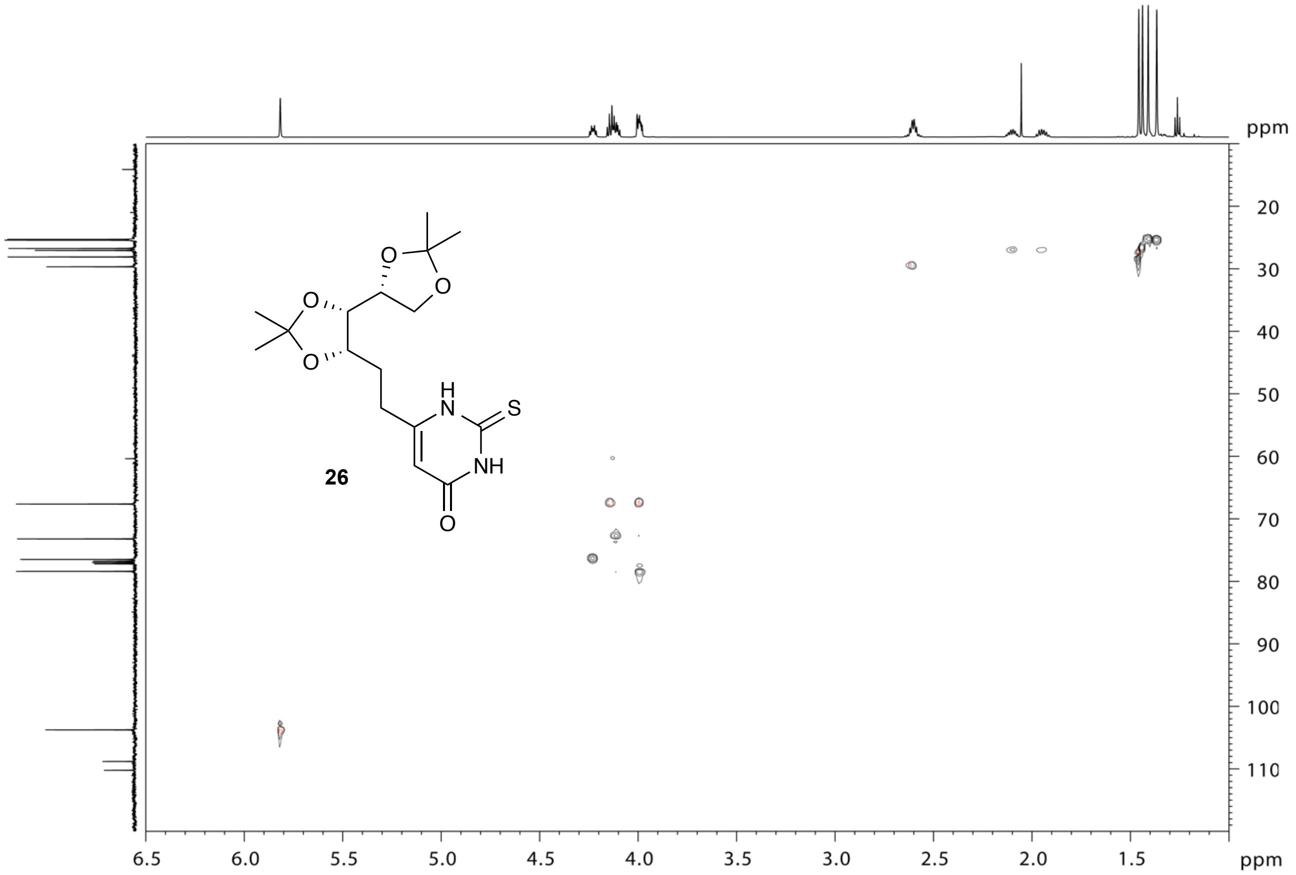

Supplementary Figure 52. (a) COSY and (b) HSQC NMR spectrum of 26 in $CDCl_3$.

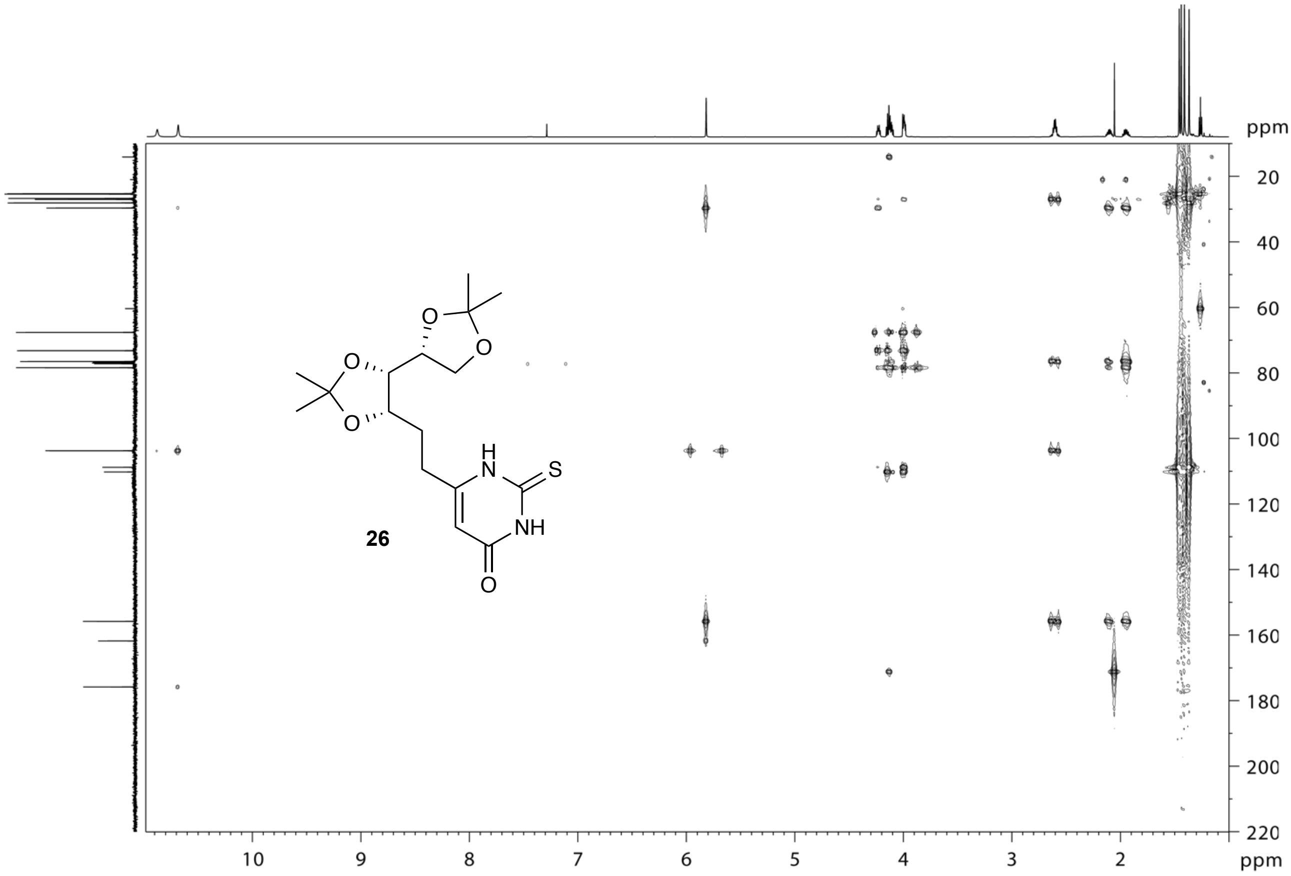

Supplementary Figure 53. HMBC NMR spectrum of 26 in $CDCl_3$.

a

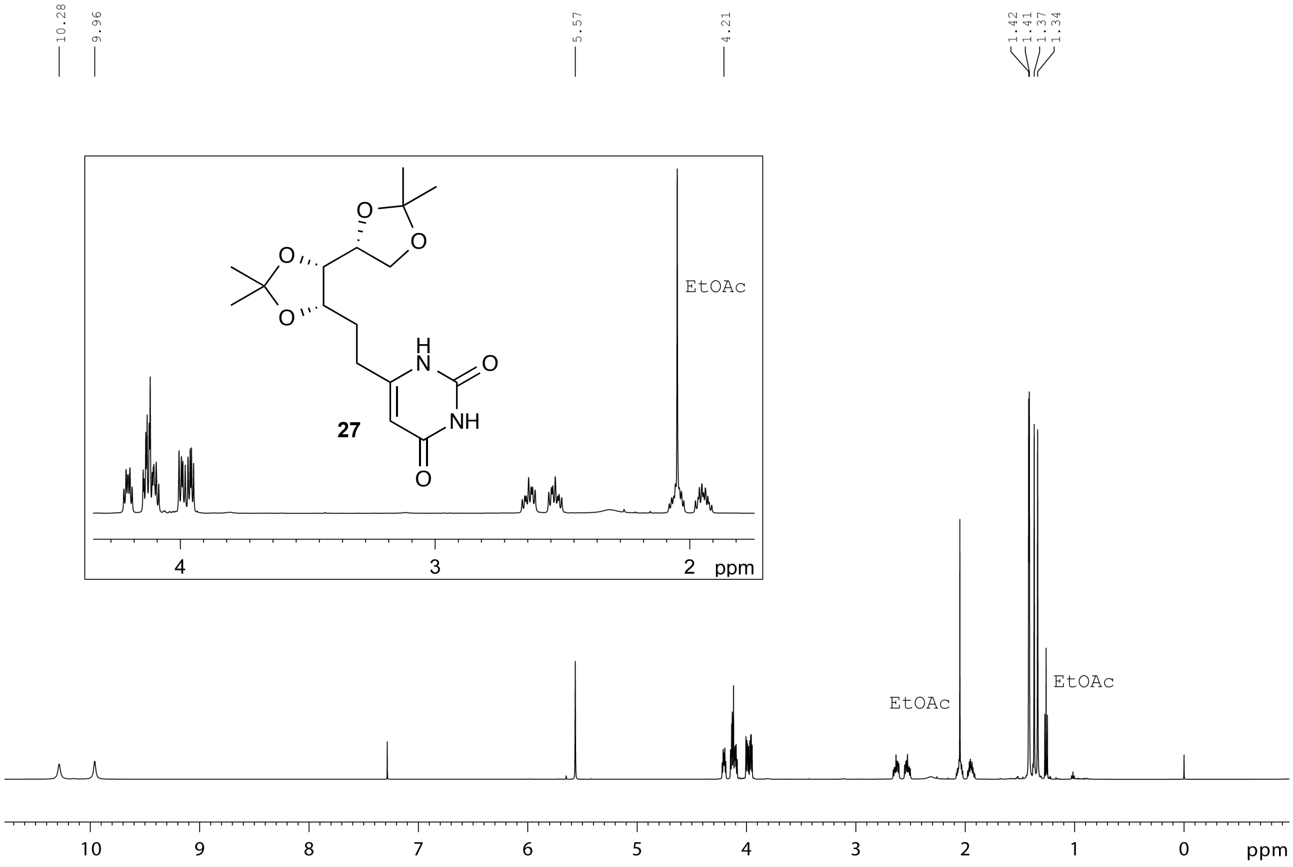

b

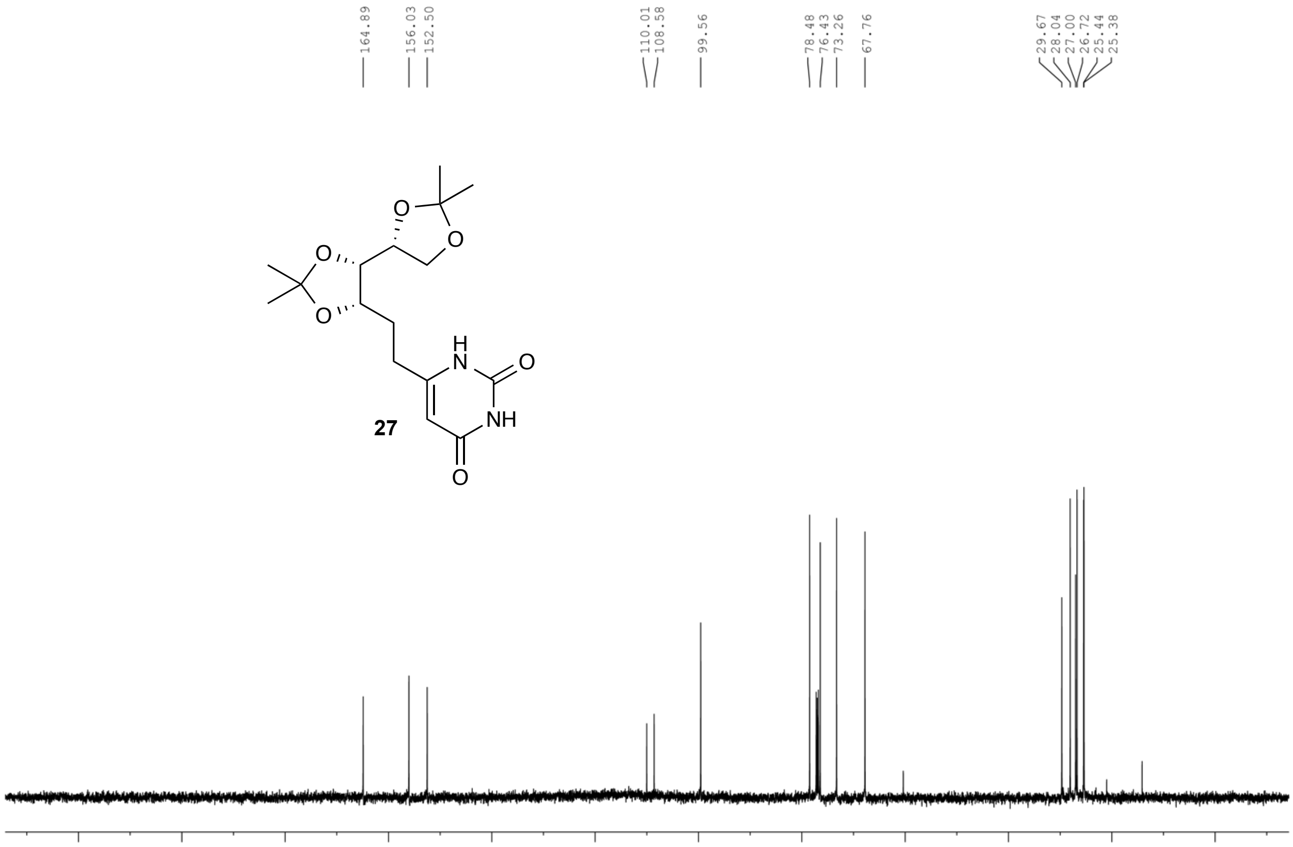

Supplementary Figure 54. (a) ^{1}H and (b) ^{13}C NMR spectrum of 27 in $CDCl_3$.

a

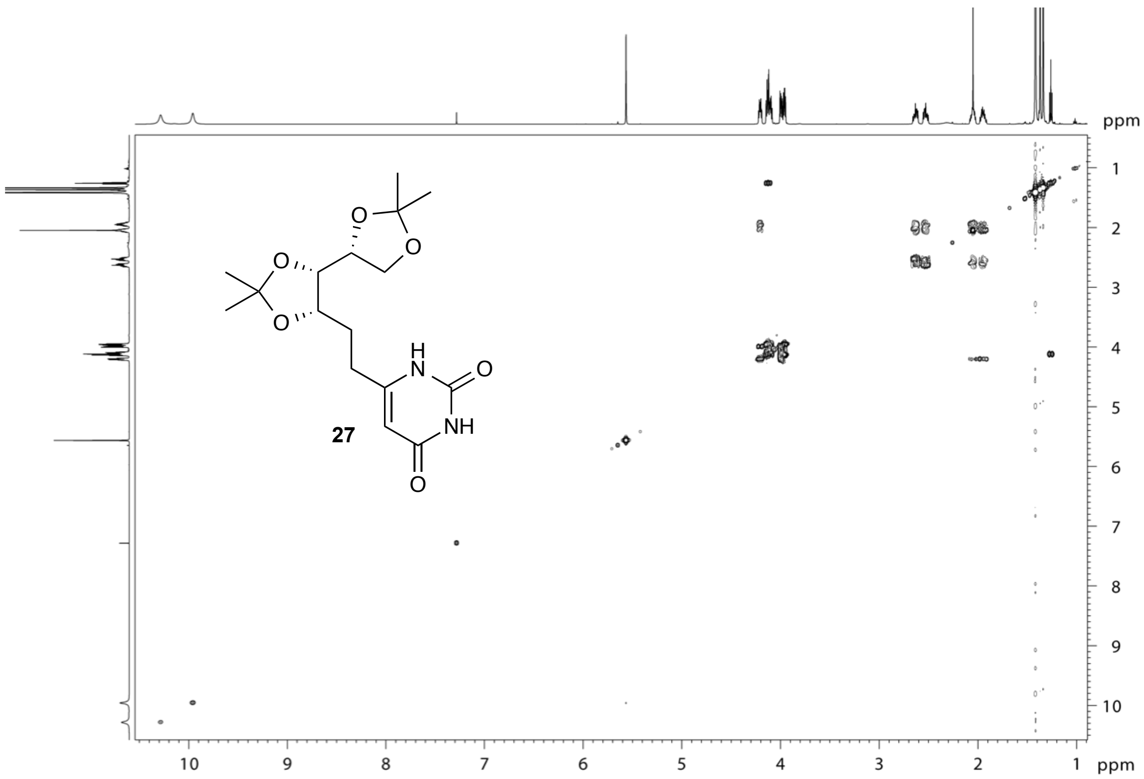

b

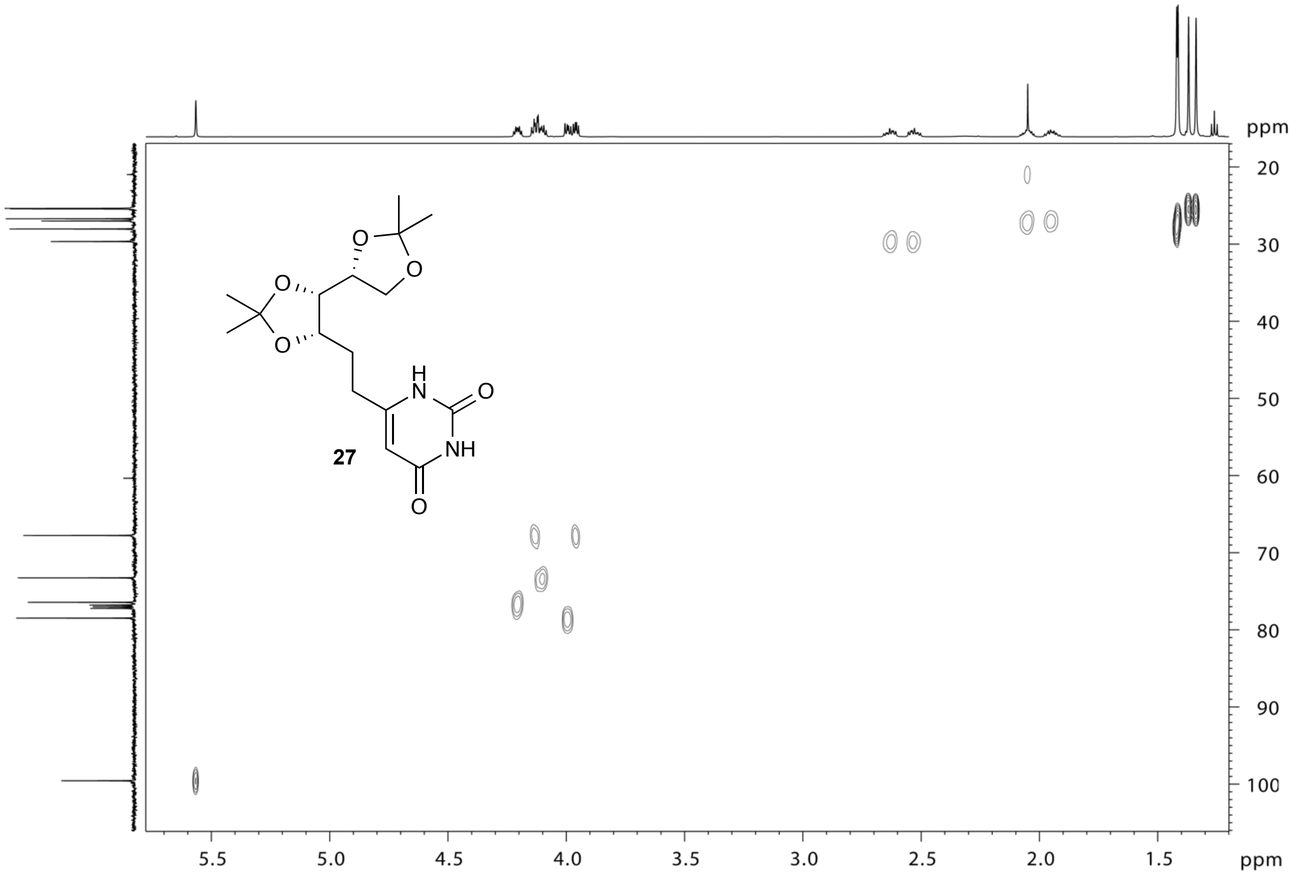

Supplementary Figure 55. (a) COSY and (b) HSQC NMR spectrum of 27 in $CDCl_3$.

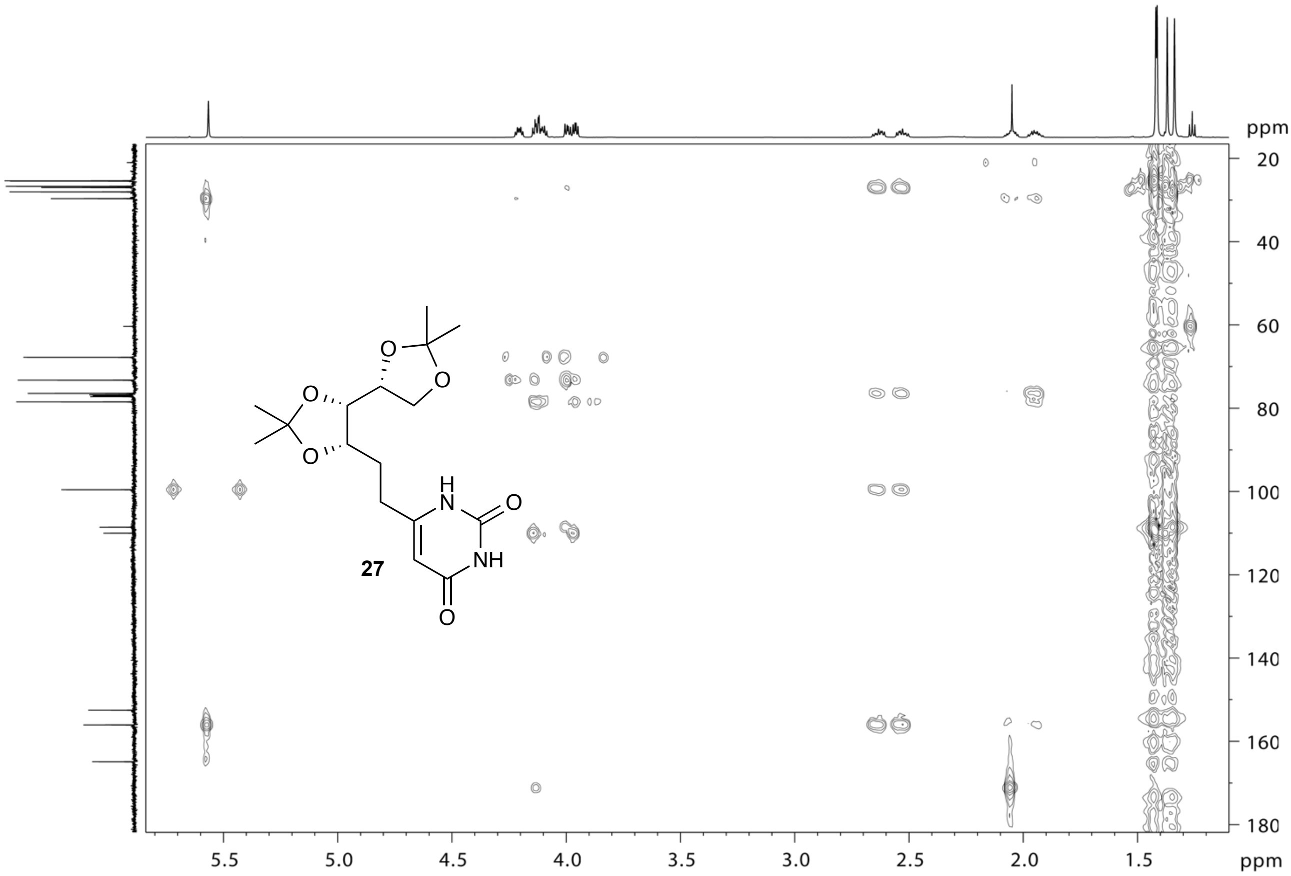

Supplementary Figure 56. HMBC NMR spectrum of 27 in $CDCl_3$.

a

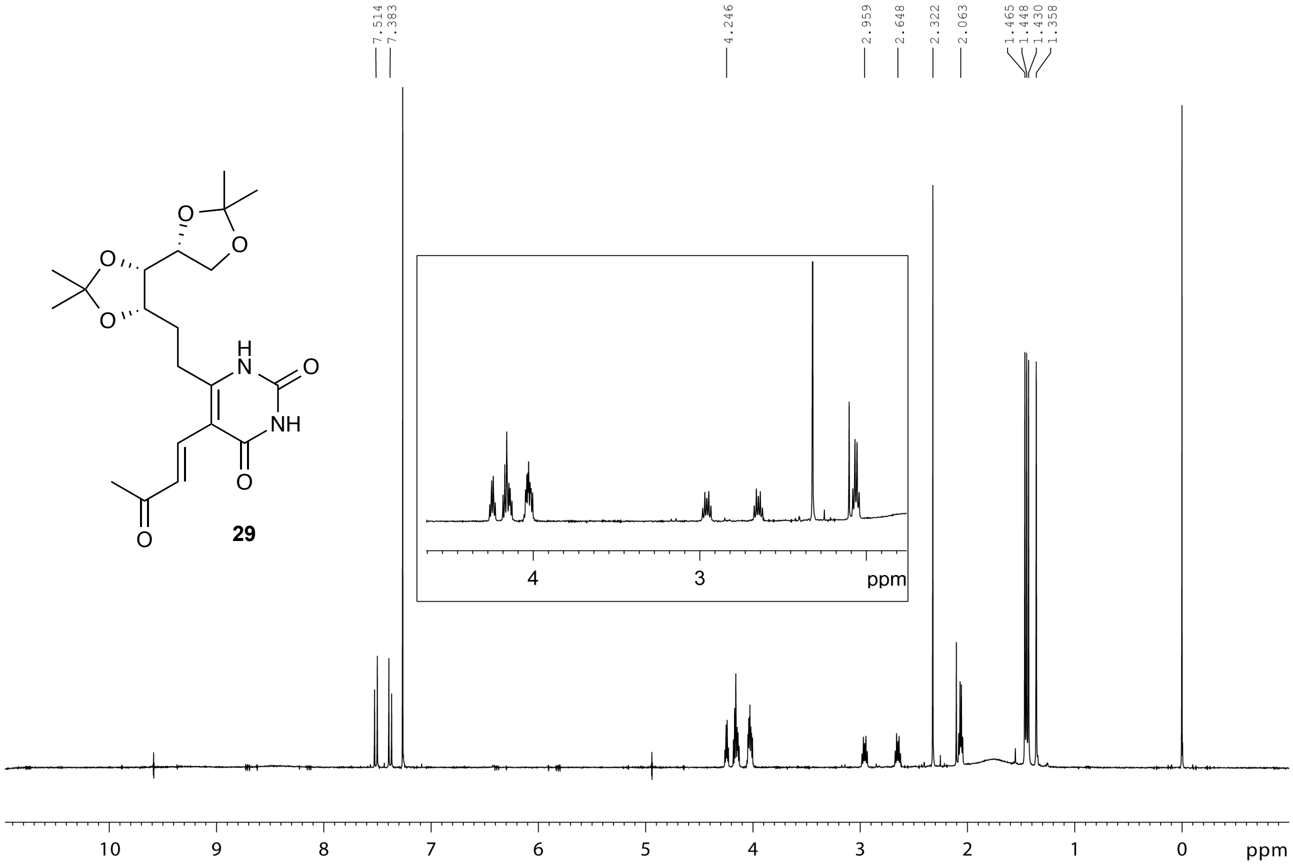

b

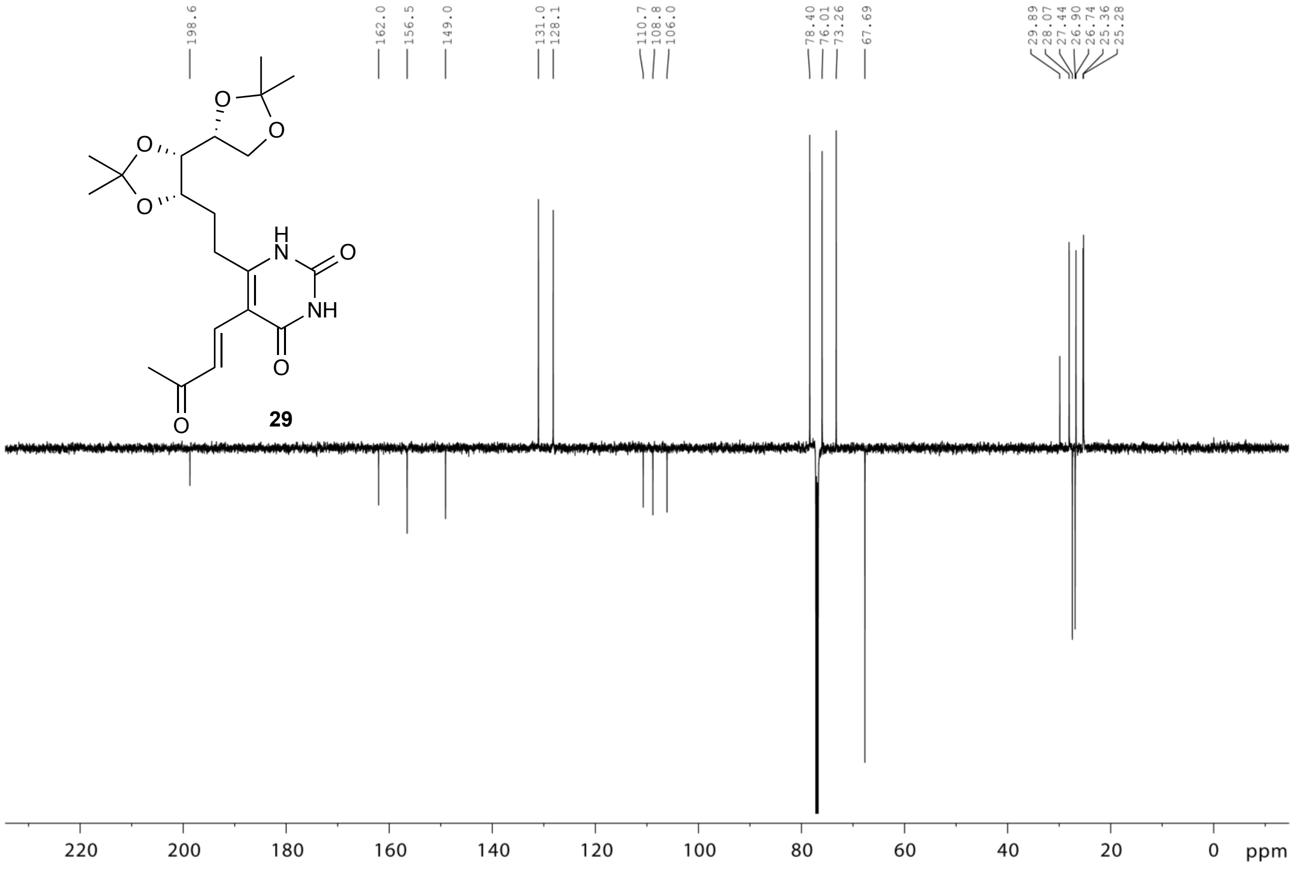

Supplementary Figure 57. (a) ^{1}H and (b) ^{13}C NMR spectrum of 29 in $CDCl_3$.

a

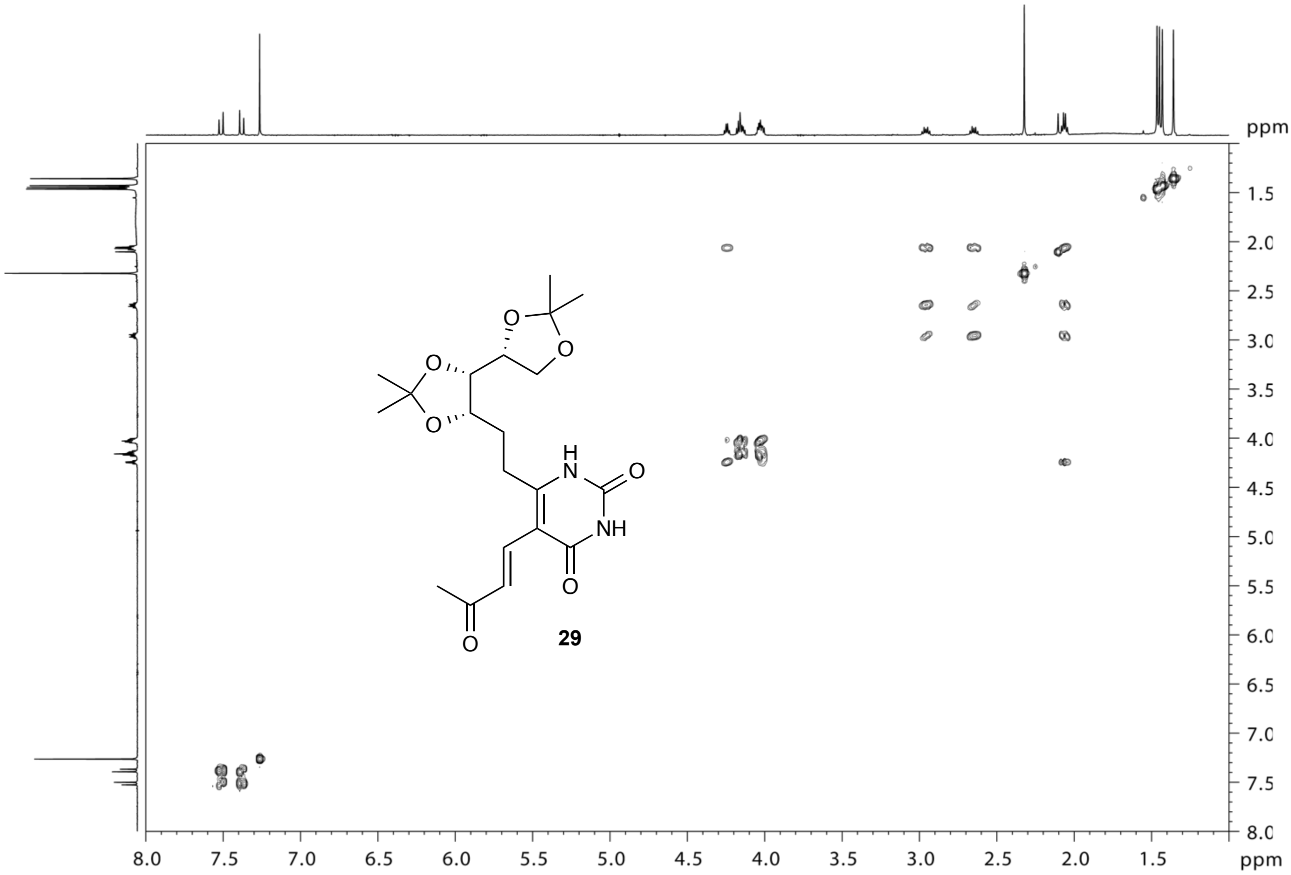

b

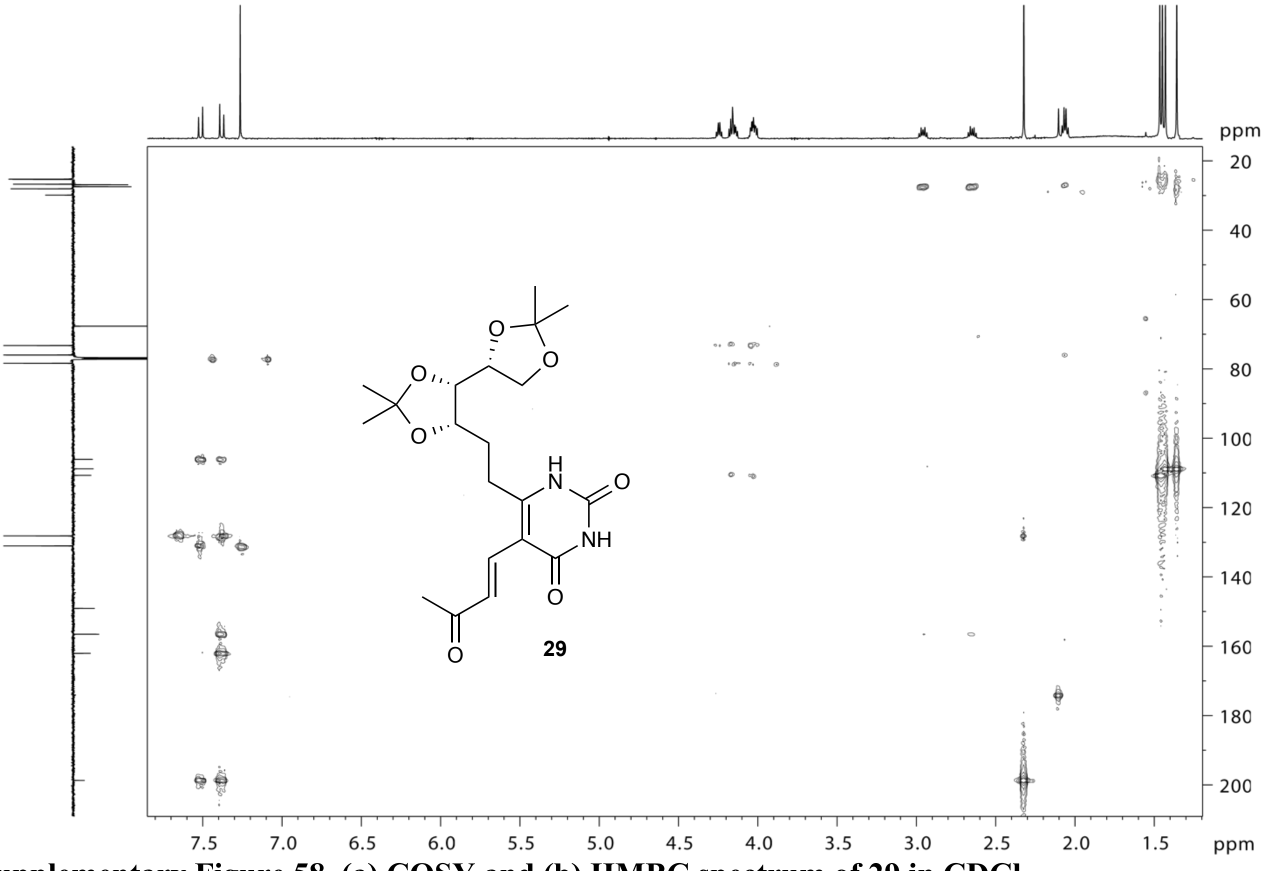

Supplementary Figure 58. (a) COSY and (b) HMBC spectrum of 29 in $CDCl_3$.

Abbreviations

5-A-RU: 5-amino-6-D-ribitylaminouracil

5-MOP-RU: 5-(1-methyl-2-oxopropylideneamino)-6-D-ribitylaminouracil

5-OE-RU: 5-(2-oxoethylideneamino)-6-D-ribitylaminouracil

5-OP-RU: 5-(2-oxopropylideneamino)-6-D-ribitylaminouracil

Ag: antigen

APC: antigen presenting cell

CD1: cluster of differentiation 1

CD3: cluster of differentiation 3, a T-cell marker

CD69: cluster of differentiation 69, a marker of T cell activation

HMBC: heteronuclear multiple bond correlation NMR spectroscopy

MR1: major histocompatibility complex class I-related protein

MAIT cell: mucosal associated invariant T cell

MHC: major histocompatibility complex

PBS: phosphate buffered saline

PE: phycoerythrin

rpHPLC, reversed phase high-performance liquid chromatography

TBS: TRIS buffered saline

TCR: T cell receptor

TRAV1-2: T cell receptor alpha variable 1-2

TRAJ33: T cell receptor alpha joining 33

TRBV6-1: T cell receptor beta variable 6-1

Supplementary References

1 Burchat, A. F., Chong, J. M. & Nielsen, N. Titration of alkyllithiums with a simple reagent to a blue endpoint. *J. Organomet. Chem.* **542**, 281-283 (1997).

2 Plaut, G. W. E. & Harvey, R. A. Enzymic synthesis of riboflavine. *Methods Enzymol.* **18B**, 515-538 (1971).

3 Piotto, M., Saudek, V. & Sklenar, V. Gradient-tailored excitation for single-quantum NMR-spectroscopy of aqueous-solutions. *J. Biomol. NMR* **2**, 661-665 (1992).

4 Corbett, A. J. *et al.* T-cell activation by transitory neo-antigens derived from distinct microbial pathways. *Nature* **509**, 361-365 (2014).

5 Beach, R. L. & Plaut, G. W. E. Investigations of structures of substituted lumazines by deuterium exchange and nuclear magnetic resonance spectroscopy. *Biochemistry* **9**, 760-770 (1970).

6 Zee-Cheng, K.-Y. & Cheng, C. C. Synthesis of 5,7-dioxo-3-methyl-5,6,7,8-tetrahydropyrimido[5,4-e]-as-triazine. *J. Med. Chem.* **11**, 1107-1108 (1968).

7 Chen, J. *et al.* Design, synthesis, and evaluation of acyclic C-nucleoside and N-methylated derivatives of the ribitylaminopyrimidine substrate of lumazine synthase as potential enzyme inhibitors and mechanistic probes. *J. Org. Chem.* **69**, 6996-7003 (2004).

8 Nencka, R. *et al.* Discovery of 5-substituted-6-chlorouracils as efficient inhibitors of human thymidine phosphorylase. *J. Med. Chem.* **50**, 6016-6023 (2007).

9 Kuroda, H., Hanaki, E., Izawa, H., Kano, M. & Itahashi, H. A convenient method for the preparation of α-vinylfurans by phosphine-initiated reactions of various substituted enynes bearing a carbonyl group with aldehydes. *Tetrahedron* **60**, 1913-1920 (2004).

10 Allevi, P., Ciuffreda, P., Tarocco, G. & Anastasia, M. The first synthesis of all possible stereoisomers of the (E)-4,5-dihydroxydec-2-enal, in homochiral form. *Tetrahedron: Asymmetry* **6**, 2357-2364 (1995).

11 Mander, L. N. & Sethi, S. P. Regioselective synthesis of β-ketoesters from lithium enolates and methyl cyanoformate. *Tetrahedron Lett.* **24**, 5425-5428 (1983).

12 Crepaldi, P. *et al.* 6-Amino-2-mercapto-3H-pyrimidin-4-one derivatives as new candidates for the antagonism at the P2Y12 receptors. *Bioorg. Med. Chem.* **17**, 4612-4621 (2009).

13 Novakov, I. A., Orlinson, B. S. & Navrotskii, M. B. Desulfurization of 2-thioxo-1,2,3,4-tetrahydropyrimidin-4-ones with oxiranes and 2-haloacetonitriles. *Russ. J. Org. Chem.* **41**, 607-609 (2005).

14 Dreier, L. & Wider, G. Concentration measurements by PULCON using X-filtered or 2D NMR spectra. *Magn. Reson. Chem.* **44**, S206-S212 (2006).

15 Gaussian 09 (Gaussian, Inc., Wallingford, CT, USA, 2009).

16 Kjer-Nielsen, L. *et al.* MR1 presents microbial vitamin B metabolites to MAIT cells. *Nature* **491**, 717-723 (2012).
